# Ynamide Carbopalladation: A Flexible Route to Mono-, Bi- and Tricyclic Azacycles

**DOI:** 10.1002/chem.201501710

**Published:** 2015-07-16

**Authors:** Craig D Campbell, Rebecca L Greenaway, Oliver T Holton, P Ross Walker, Helen A Chapman, C Adam Russell, Greg Carr, Amber L Thomson, Edward A Anderson

**Affiliations:** [a] Chemistry Research Laboratory, University of Oxford 12 Mansfield Road, Oxford, OX1 3TA (U.K.) edward E-mail: anderson@chem.ox.ac.uk; [b] Syngenta Ltd. Jealott's Hill International Research Centre Bracknell, Berkshire, RG42 6EY (U.K.); [c] AstraZeneca Alderley Park, Macclesfield, SK10 4TF (U.K.)

**Keywords:** domino reactions, hetero-Diels–Alder reactions, palladium catalysis, Suzuki coupling, ynamides

## Abstract

Bromoenynamides represent precursors to a diversity of azacycles by a cascade sequence of carbopalladation followed by cross-coupling/electrocyclization, or reduction processes. Full details of our investigations into intramolecular ynamide carbopalladation are disclosed, which include the first examples of carbopalladation/cross-coupling reactions using potassium organotrifluoroborate salts; and an understanding of factors influencing the success of these processes, including ring size, and the nature of the coupling partner. Additional mechanistic observations are reported, such as the isolation of triene intermediates for electrocyclization. A variety of hetero-Diels–Alder reactions using the product heterocycles are also described, which provide insight into Diels–Alder regioselectivity.

## Introduction

**Scheme 1:**
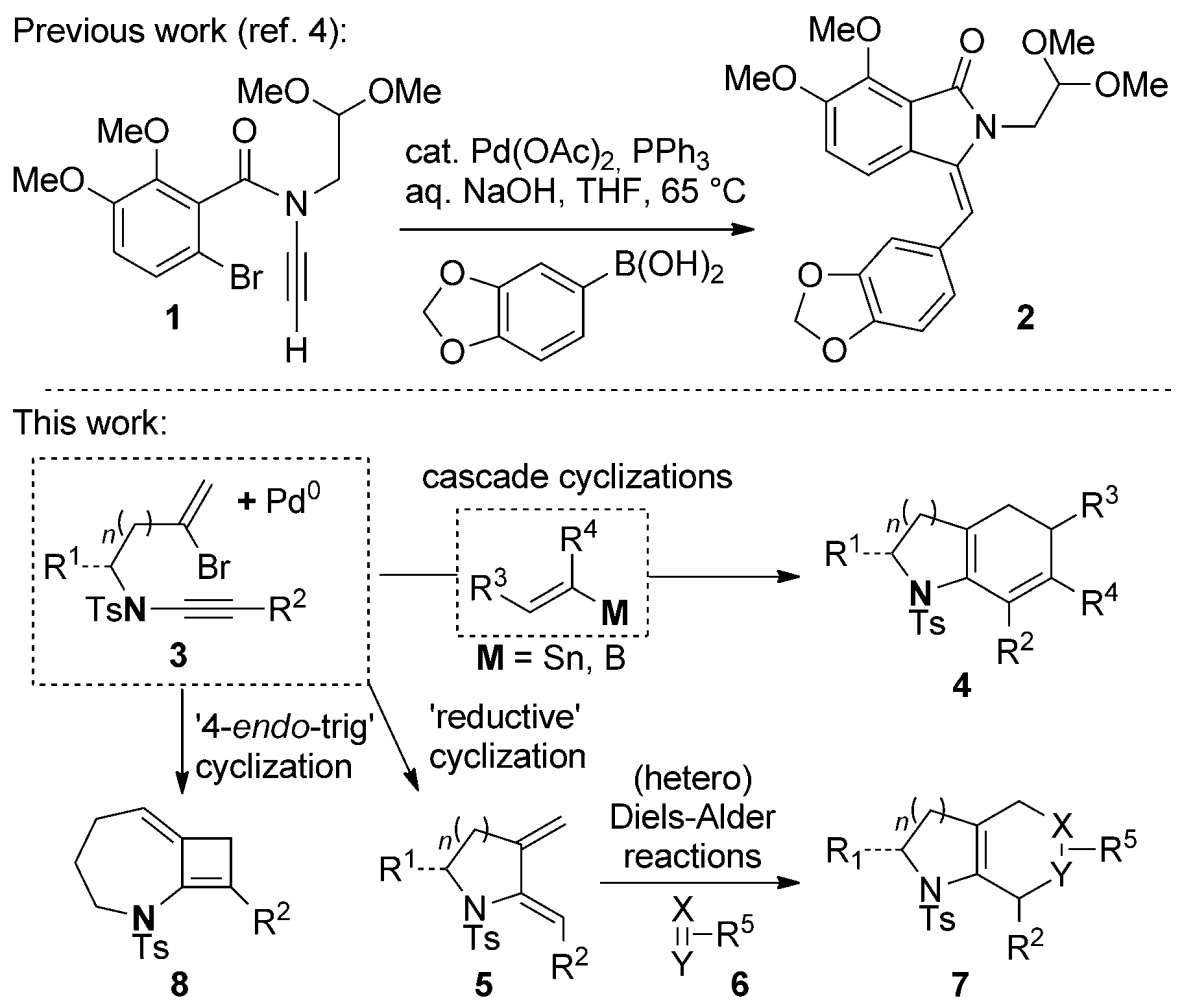
Ynamide carbopalladation.

Nitrogen-containing ring systems are of fundamental importance in chemistry and biology, and the development of efficient and general methods for their preparation remains a key challenge for organic chemists.[1] Ynamides offer an appealing entry to azacycles, as the intrinsic polarization of the ynamide can lead to heightened reactivity, as well as regio- and stereoselectivity, in cyclization reactions.[2] Notably, ynamide carbopalladation is relatively unexplored as a ring-forming tool, despite the rich history and indisputable value of this process in the cyclization of alkynes.[3] At the outset of our work, only a single study of ynamide carbopalladation had been disclosed,[4] which focused on the intramolecular carbopalladation of terminal ynamides by arylpalladium(II) complexes, terminating either through reduction of the inter mediate alkenylpalladium(II) species with ammonium formate, or Suzuki cross-coupling with an arylboronic acid, as employed in a total synthesis of the natural product lennoxamine (see **1**→**2**, Scheme 1). Applications of ynamide carbopalladation using alkenyl halides, or alkenylmetal coupling partners, would thus explore unchartered ynamide territory.

In this vein, we recently described a de novo synthesis of bicyclic azacycles from acyclic bromoenynamides using a carbopalladation/alkenyl Suzuki/6π-electrocyclization cascade (**3**→**4**, M=B(OR)_2_, Scheme 1).[5] This strategy for azabicycle synthesis complements traditional routes, which usually involve heterocycle annulation onto a preformed carbocycle; azabicycle syntheses in which both rings are formed in one step offer unique opportunities for structural variation.[6] We report here full details of the development of this process, from the first examples of Stille cross-coupling in this ynamide cascade (M=SnR_3_), to the evolution and subtleties of the Suzuki alternative, including the first use of potassium alkenyltrifluoroborate salts[7] in any carbopalladation/cross-coupling sequence (M=BF_3_K).[8] We also discuss the development of a reductive cyclization[9] that affords monocyclic amidodienes **5**—products which through [4+2] cycloadditions (including hetero-Diels–Alder reactions with dienophiles **6**) lead to heterobicyclic frameworks **7**, of potential use in medicinal chemistry. Finally, alongside some mechanistic observations and the exploration of equivalent reactions of ynhydrazides, the formation of an unprecedented 7,4-fused ring enamide **8** by a formal 4-*endo-*trig cyclization is described.[10]

## Results and Discussion

**Scheme 2:**
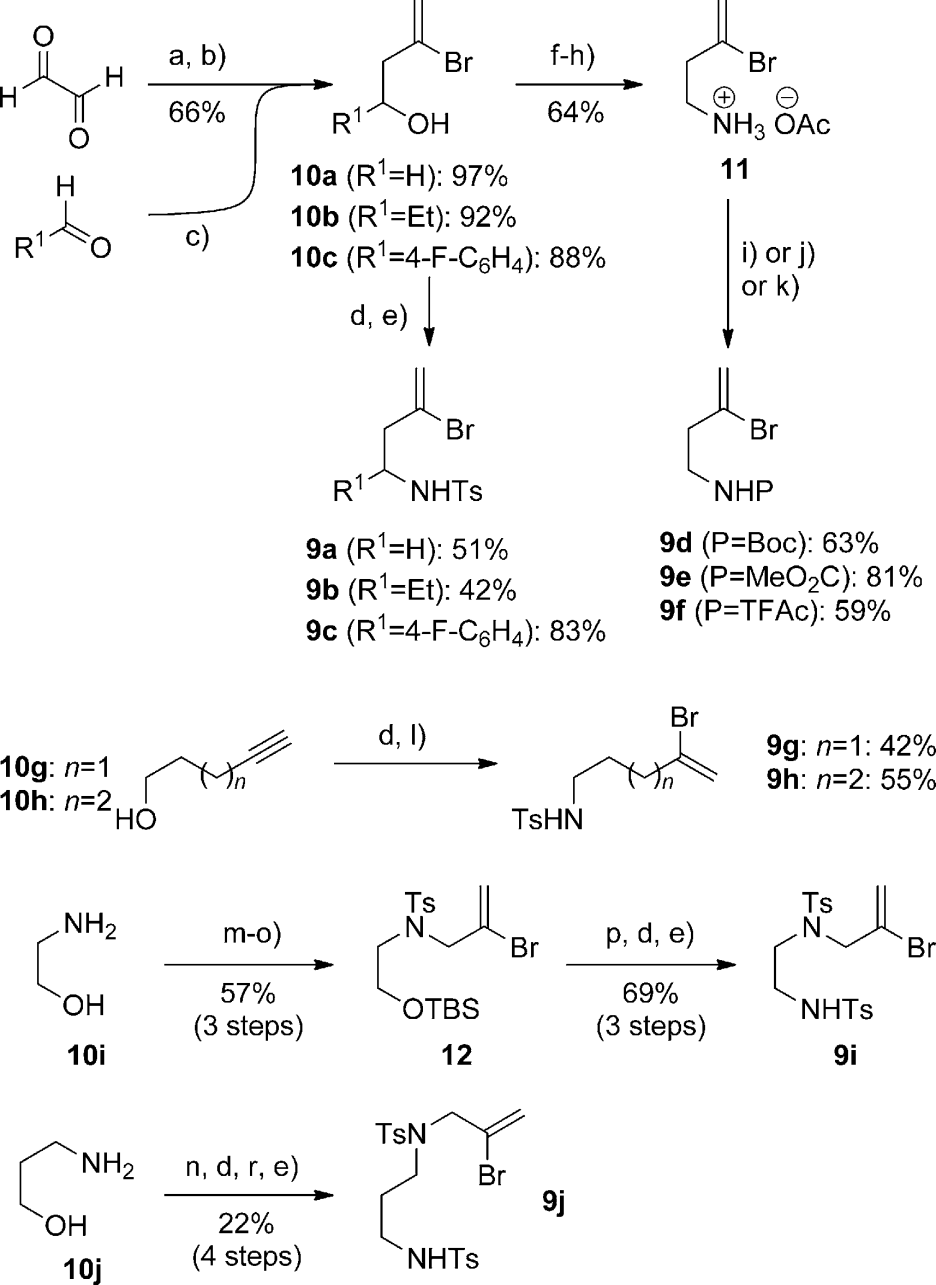
a) 2,3-dibromopropene (2.3 equiv), Sn (2.3 equiv), HBr (48 % aq., few drops), Et_2_O/H_2_O (1:1); b) NaIO_4_, MeOH/pH 7 buffer (5:1); NaBH_4_, 0 °C→RT; c) 2,3-dibromopropene (1.3 equiv), Sn (1.3 equiv), HBr (48 % aq., few drops), Et_2_O/H_2_O (1:1); d) TsBocNH, PPh_3_, DIAD, THF, 0 °C→RT; e) CF_3_CO_2_H, CH_2_Cl_2_, 0 °C; f) TsCl, py, CH_2_Cl_2_; g) NaN_3_, DMSO; h) H_2_S, pyridine/H_2_O (1:1); AcOH; i) Boc_2_O, Et_3_N, THF; j) MeOCOCl, py, CH_2_Cl_2_; k) TFAA, py, CH_2_Cl_2_; l) BBr_3_, CH_2_Cl_2_; AcOH; m) TBSCl, imid., CH_2_Cl_2_; n) TsCl, Et_3_N, CH_2_Cl_2_; o) 2,3-dibromopropene, 50 % NaOH (aq.), Bu_4_NHSO_4_ (0.1 equiv), tol/H_2_O (1:1); p) TBAF, THF, 0 °C; r) 2,3-dibromopropene, 25 % NaOH (aq.), Bu_4_NHSO_4_ (0.1 equiv), Bu_4_NI (0.1 equiv), tol/H_2_O (1:1). Procedures i)–k) were preceded by basic extraction of 11 using NaOH/CH_2_Cl_2_. DIAD=diisopropyl azodicarboxylate; TFAA=trifluoroacetic anhydride; TBAF=tetra-*n*-butylammonium fluoride.

A prerequisite for the realization of these processes was the synthesis of ynamides featuring a bromoalkene substituent. Among many methods for ynamide synthesis,[11] we were particularly attracted to Hsung’s robust copper-catalyzed coupling of amides and bromoalkynes, which has been shown to tolerate a bromoalkene.[11f] Our work thus began with the preparation of a range of bromoalkenyl amides (Scheme 2). To prepare the ‘parent’ bromoalkene sulfonamide **9 a**, we initially employed a two-directional allylation of aqueous glyoxal with 2,3-dibromopropene[12] using the procedure of Otera,[13] which gave an intermediate diol as an inconsequential mixture of diastereomers in quantitative yield. Periodate cleavage of this diol followed by in situ reduction provided alcohol **10 a**, a compound that proved somewhat unstable toward prolonged storage or distillation. A superior method for the preparation of **10 a** involved direct bromoallylation of aqueous formaldehyde, from which **10 a** was obtained in quantitative yield and sufficient purity to be employed directly in subsequent chemistry. Otera’s chemistry also proved suitable for the bromoallylation of other aldehydes, leading to the secondary alcohols **10 b**–**c**, which together with **10 a** were converted to the sulfonamides **9 a**–**c** by Mitsunobu reaction with TsBocNH, then *tert*-butoxycarbonyl (Boc) deprotection using trifluoroacetic acid. We also targeted the preparation of other amides, and after some optimization found that amine salt **11** could be prepared from **10 a** by tosylation, azide displacement, and Staudinger reduction. This was converted to the trifluoroacetyl, Boc, and methoxycarbonyl derivatives **9 d**–**f**.

With a view to the synthesis of six- and seven-membered azacycles such as tetrahydroquinolines and benzazepines, we prepared sulfonamides **9 g** and **9 h** in two steps from **10 g** and **10 h** by Mitsunobu amination, then bromoboration/protodeborylation (with in situ Boc deprotection). Sulfonamides **9 i** and **9 j** feature an additional nitrogen substituent in the tether, and thus represent precursors to diazepines and diazocanes; these were prepared from amino alcohols **10 i** and **10 j**, respectively, using equivalent chemistry. A notable feature of these latter sequences was the requirement for phase-transfer conditions to install the 2-bromoallyl group on the sulfonamides.

**Scheme 3:**
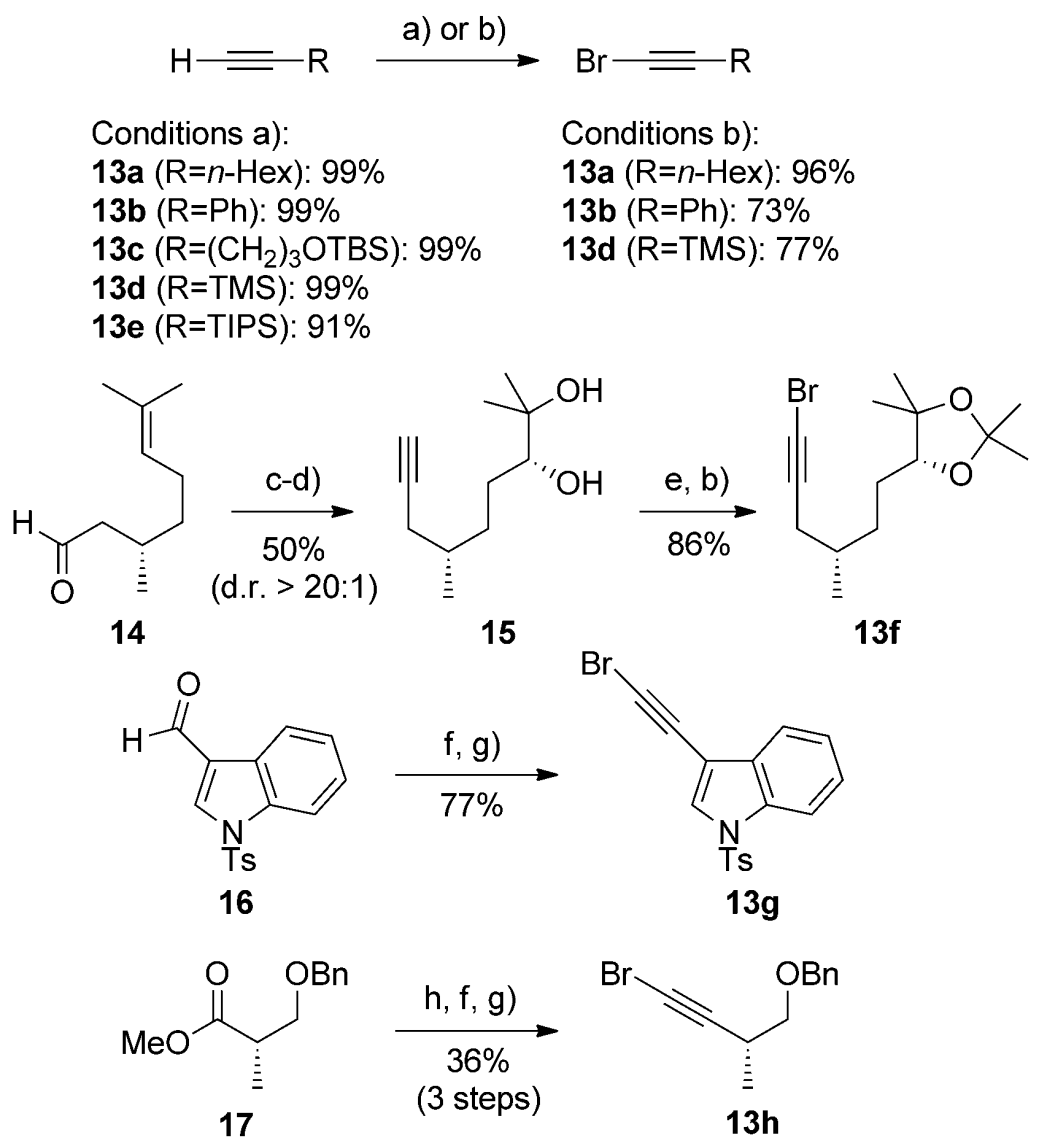
a) *n*BuLi, THF, −78 °C; Br_2_, −78 °C; b) NBS, AgNO_3_ (2.5 mol %), acetone; c) [Ph_3_PCHBr_2_]Br, KO*t*Bu, THF; 14; KO*t*Bu; d) AD-mix-β (0.4 mol %), *t*BuOH/H_2_O (1:1), 90 h; e) CH_2_(OMe)_2_, acetone, *p*-TsOH (cat.); f) CBr_4_, PPh_3_, CH_2_Cl_2_, 0 °C; g) KHMDS, THF, 0 °C; h) DIBALH, CH_2_Cl_2_, −78 °C. DIBALH=diisobutylaluminium hydride.

We next turned to the synthesis of bromoalkyne coupling partners for ynamide formation, which were prepared from the corresponding alkynes either by lithiation/bromine quench (**13 a**–**e**, Scheme 3), or preferably and more mildly using *N*-bromosuccinimide (NBS) and catalytic silver(I) nitrate (**13 a**–**f**).[14] The bromoalkynyl indole **13 g** was conveniently accessed from 3-formylindole **16**­ *via* vinyl dibromide formation,[15] then elimination of HBr using potassium hexamethyldisilazide (KHMDS), a strategy that also served well for the formation of the Roche ester-derived bromoalkyne **13 h**.

**Figure 1:**
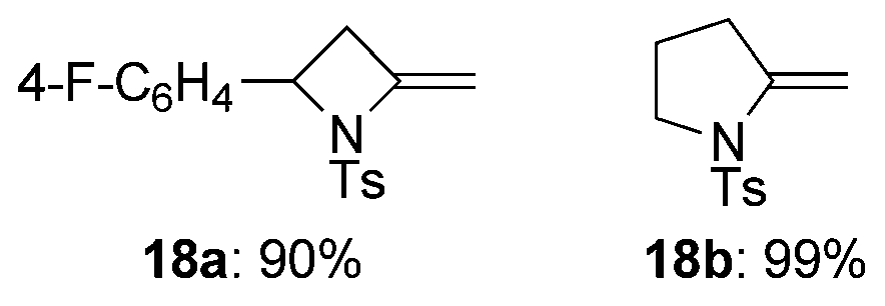
Copper-catalyzed azetidine and pyrrolidine enamide formation.

With a range of amides and bromoalkynes in hand, ynamide synthesis was addressed using the Hsung method (catalytic CuSO_4_/1,10-phenanthroline, K_3_PO_4_, toluene, 80 °C, Table 1). These conditions indeed proved successful for the formation of ynamides **3 a**–**h** from sulfonamide **9 a**, with the bromoalkene surviving unscathed; these ynamides were delivered in good to excellent yields in all cases, including the indole-substituted ynamide **3 f**, and more complex examples featuring additional functionality and stereogenic centres (e.g. **3 c**, **3 g**, **3 h**). An exception was TMS-ynamide **3 d**, where we experienced varying degrees of desilylation under the reaction conditions. Disappointingly, the Boc-protected ynamide **3 i** was obtained only in low yield even under extended reaction times, an observation consistent with the poor ynamide-forming reactivity generally observed with acyclic carbamates.[16] Similarly, no reaction could be achieved for amides **9 e**–**f** under any copper- or iron-catalyzed ynamide forming conditions.[11] In the case of α-branched sulfonamide **9 c**, copper catalysis afforded the exocyclic enamide azetidine **18 a**[17] (Figure 1) instead of the desired ynamide. Presumably, a modest Thorpe–Ingold effect (and/or steric hindrance) favours this cyclization to the total exclusion of ynamide formation; indeed the copper-catalyzed formation of such enamides is known.[17–18] Similarly, reaction of 3-bromoalkenyl sulfonamide **9 g** also led to cyclization to the pyrrolidine enamide **18 b**.[18] We were therefore rather surprised to find that sulfonamides **9 h**–**j** (featuring four- or five-atom tethers between the amide and bromoalkene) could be successfully converted to ynamides **3 j**–**m** under copper catalysis (Table 1); in none of these cases were piperidine or azepine enamide byproducts detected.

**Table 1:** Copper-catalyzed bromoalkenylynamide synthesis^[a]^

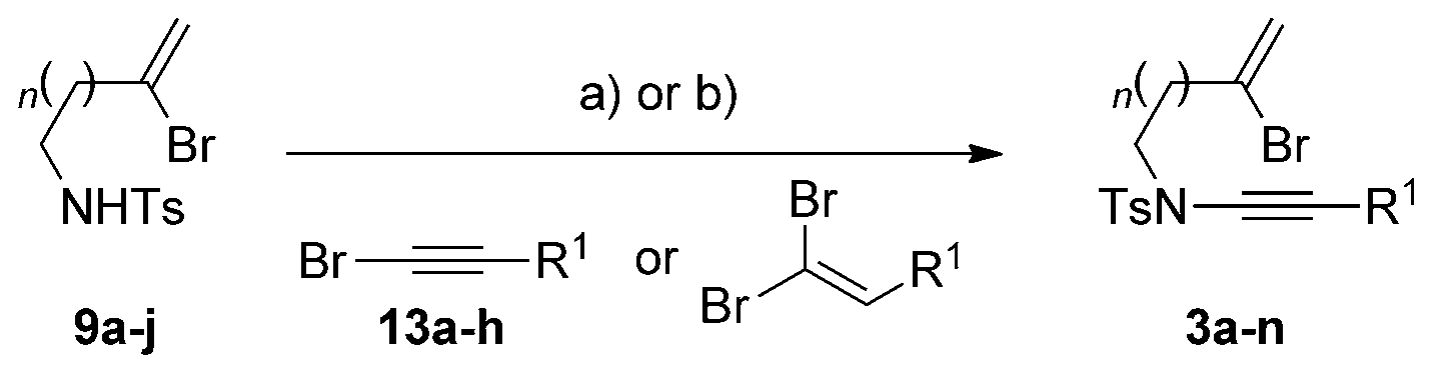
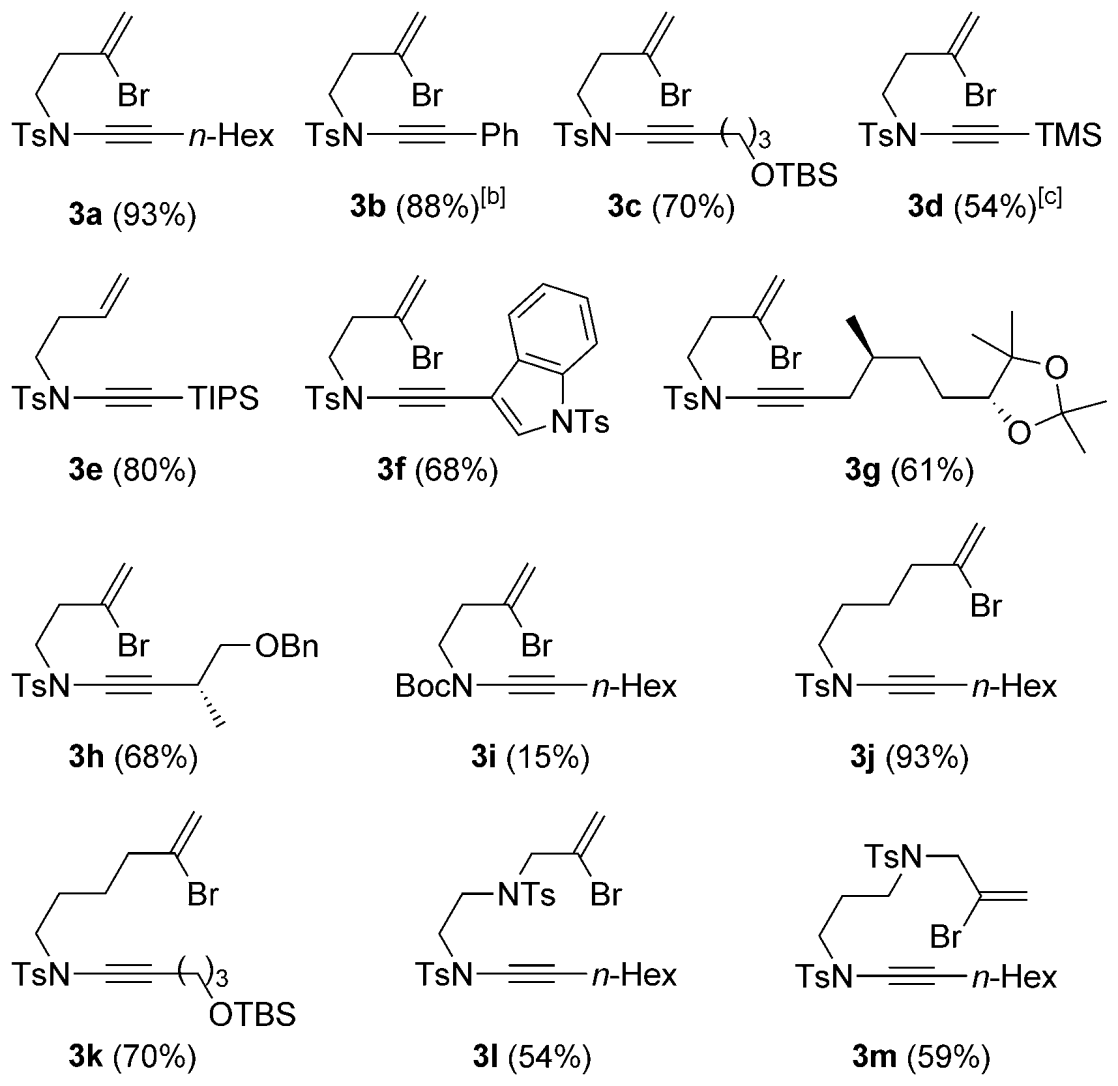

[a] All reactions were performed by using conditions (a) unless stated otherwise. Reaction conditions: a) amide (1.0 equiv), bromoalkyne (1.5 equiv), CuSO_4_**⋅**5 H_2_O (20 mol %), 1,10-phenanthroline (40 mol %), K_3_PO_4_ (2.0 equiv), toluene (0.25 m), 70 °C, 16 h; b) amide (1.0 equiv), dibromoalkene (1.5 equiv), CuI (12 mol %), *N*,*N′*-dimethylethylenediamine (18 mol %), Cs_2_CO_3_ (4.0 equiv), 1,4-dioxane (0.27 M), 60 °C, 16 h. [b] Performed using conditions (b). [c] Significant amounts of desilylation were observed.

To overcome the failure of metal-catalyzed syntheses of ynamides from sulfonamides **9 b**, **9 c** or **9 g**, we instead employed Witulksi’s alkynyliodonium triflate methodology (Table 2).^[11^ ^m]^ This chemistry, although widely applied in synthesis,[19] is well-suited only to the preparation of silylated ynamides. In our hands, the protocol gave modest yields of the corresponding TMS-substituted ynamides (40–47 %); subsequent desilylation and Sonogashira coupling afforded aryl-substituted ynamides **3 n**–**q**. As reported by Hsung,[20] ynamide Sonogashira coupling only proved successful using minimal amounts of copper(I) salts in order to avoid extensive ynamide homocoupling, and was nonetheless also only modest yielding (38–52 %).

**Table 2:** Alkynyliodonium triflate route to ynamides^[a]^

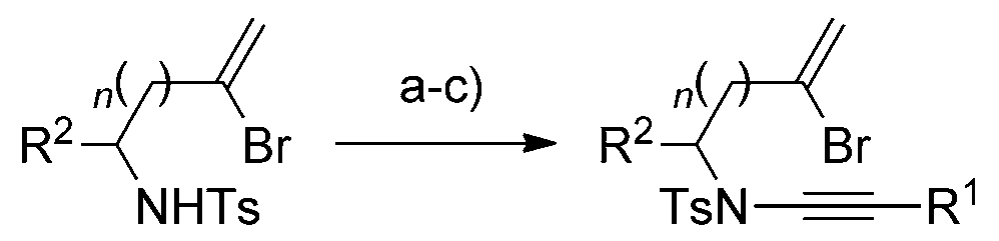
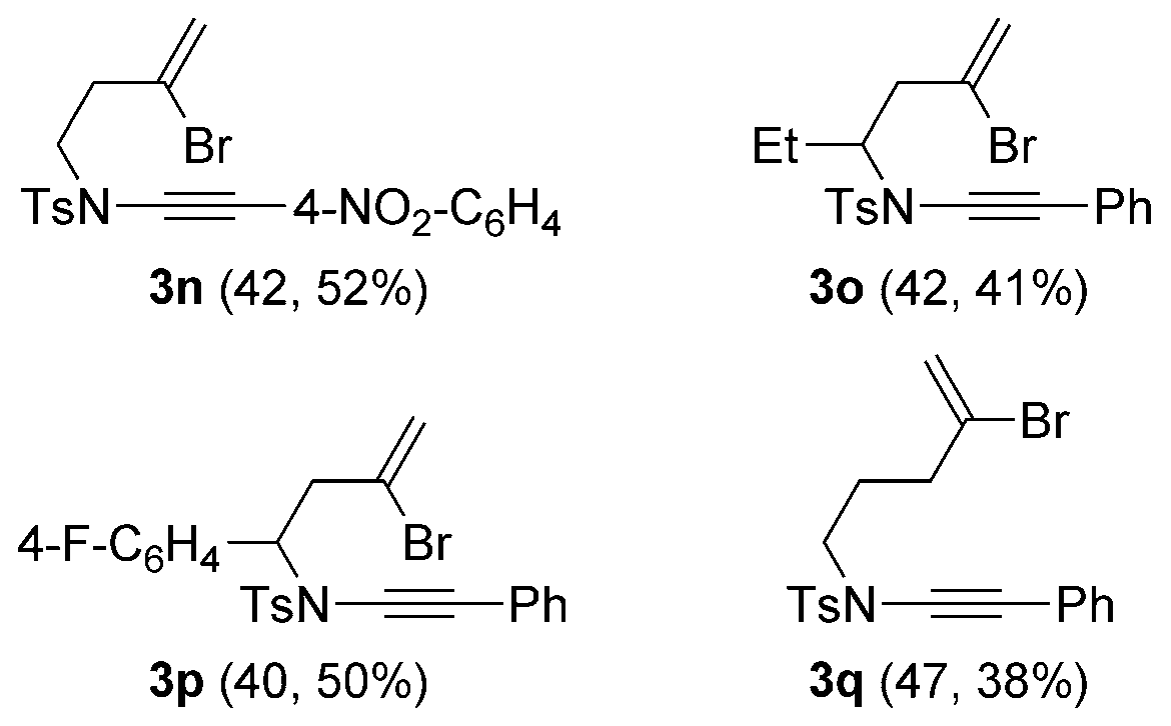

[a] Reaction conditions: a) Sulfonamide, KHMDS, TfOIPh(C≡C)TMS, toluene, −78 °C; b) TBAF (1 m in THF), THF; c) [Pd(PPh_3_)_4_] (5 mol %), CuI (<1 mol %), R^1^–I, toluene, 60 °C. The first yield is the yield for step a; the second yield is the combined yield for steps b and c.

With a wide selection of bromoenynamides in hand, attention turned to their cyclizations to bicyclic cyclohexadienamides. At the outset of this project, our initial focus was the use of Stille coupling, where the extensive investigations of the Suffert group on carbon-tethered bromoene- alkynes have shown the carbopalladation/Stille/electrocyclization manifold to be a rich source of reactivity and complex carbocycles.[21] Our own work on this carbon-tethered cascade mirrors Suffert’s findings, where in addition to developing a general approach to *n*,6- and *n*,8-fused bicyclic rings,[10, 22] we had employed this reaction to prepare the 7,8,5-CDE rings of lancifodilactone G.[23] We now hoped that this chemistry could be extended to the ynamide setting where, as mentioned, only reactions involving aryl halide and aryl boronate components had been explored prior to these studies.[4]

**Figure 2:**
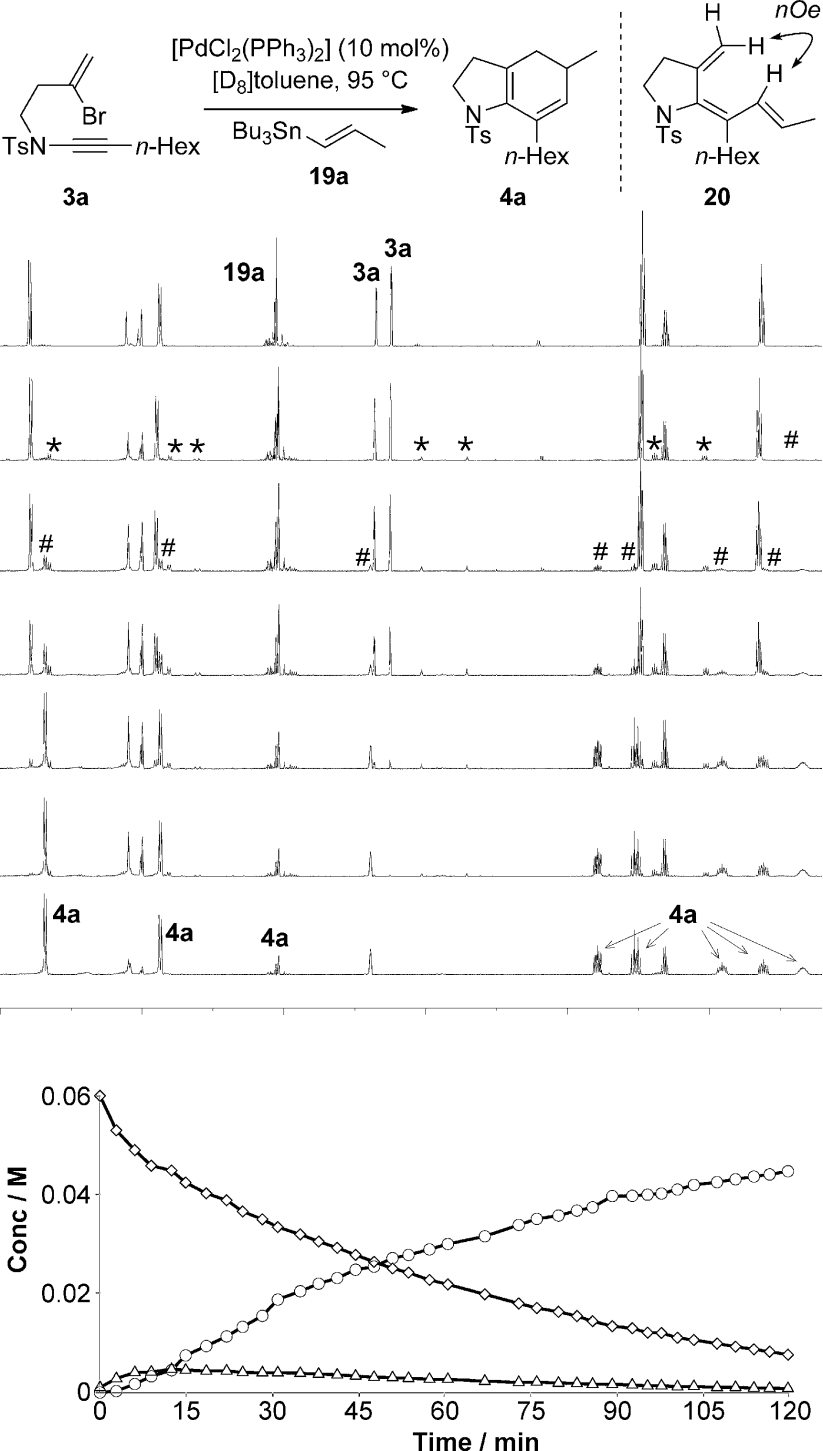
VT NMR spectroscopic studies of the ynamide carbopalladation/cross-coupling/electrocyclization sequence. ◊: 3 a, ○: 4 a, ▵: 20.

To investigate the conditions that would be required to effect ynamide cyclization, we subjected bromoenynamide **3 a** to coupling with stannane **19 a**, with [PdCl_2_(PPh_3_)_2_] as the catalyst (10 mol %), using variable temperature ^1^H NMR spectroscopy (VT NMR) to monitor reaction progress (Figure 2). The reaction initiated at 95 °C, as evidenced by the formation of several new peaks after 10–20 min at this temperature (Figure 2, “*” peaks: *δ*=6.63 (1 H, d, *J*=15.5 Hz), 5.30 (1 H, s), 4.70 (1 H, s), 3.39 (2 H, t, *J*=15.5 Hz), 3.04 ppm (2 H, t, *J*=15.5 Hz)). After 30 minutes, product formation was apparent (“#” peaks: *δ*=5.39 ppm (1 H, s)), which increased steadily in intensity over the subsequent two hours, by which time **3 a** (and the putative reaction intermediate observed initially) had been completely consumed.

**Scheme 4:**
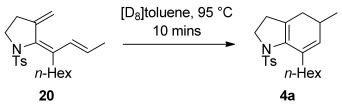
Thermal electrocyclization of triene 20 to azabicycle 4 a.

To determine the nature of this intermediate, the reaction was halted after ten minutes, and the intermediate was isolated as the predominant component of a 1:0.23:0.8 mixture (ratio of intermediate**/**ynamide **3 a/**product **4 a**) by careful column chromatography. This allowed its assignment as the triene **20**, the stereochemistry of which was determined by mutual ^1^H NMR nOe enhancements from the indicated propenyl proton to the *exo*-methylene (Figure 2). Support for the genuine intermediacy of this species was obtained by subjecting this purified triene-containing mixture to thermal cyclization conditions in the absence of catalyst ([D_8_]toluene, 95 °C, 10 min), which led to complete conversion of **20** to the product diene **4 a** (Scheme 4), suggesting that the electrocyclization step of the reaction operates independently of the catalyst. Whilst the formation of triene **20** is expected on the basis of the likely reaction mechanism, the observation of *syn*[24] trienes in cascade carbopalladation/electrocyclization is quite rare.[21a,b,i, 25] Indeed, in our previous work on bromoenyne cyclizations[22] and in the work of others,[21a,b,j, 26] isomeric *anti* trienes had been detected that we found could also be converted to product, but only in the presence of palladium(0) catalyst;[22] the present result thus reveals a subtle difference in behaviour between ynamides and ‘normal’ alkynes in carbopalladation processes.[27] To conclude this optimization, a brief survey of other catalysts (e.g. [Pd(PPh_3_)_4_], Pd(OAc)_2_) offered no improvement over the conditions used in the VT NMR spectroscopic experiment. We also found that the catalyst loading could be reduced to 1 mol %, and for the remainder of this work, catalyst loadings of 1 or 10 mol % were routinely employed.

With reaction conditions established, the cyclization was tested with a range of bromoenynamides and vinyl stannanes (Table 3). We were delighted to find that alkyl-, aryl-, and silyl-substituted ynamides **3 a**–**d** were all viable substrates, and that a variety of alkenylstannane partners featuring alkyl, aryl, and silyl groups (**19 a**–**d**) could be employed, giving a selection of disubstituted bicyclic cyclohexadienes **4 a**–**g**. Of particular note is the sterically-challenging cyclization with pyranyl stannane **19 e**, which afforded the tricyclic product **4 h** in good yield.

**Table 3:** Cyclizations of bromoenynamides under Stille conditions^[a]^

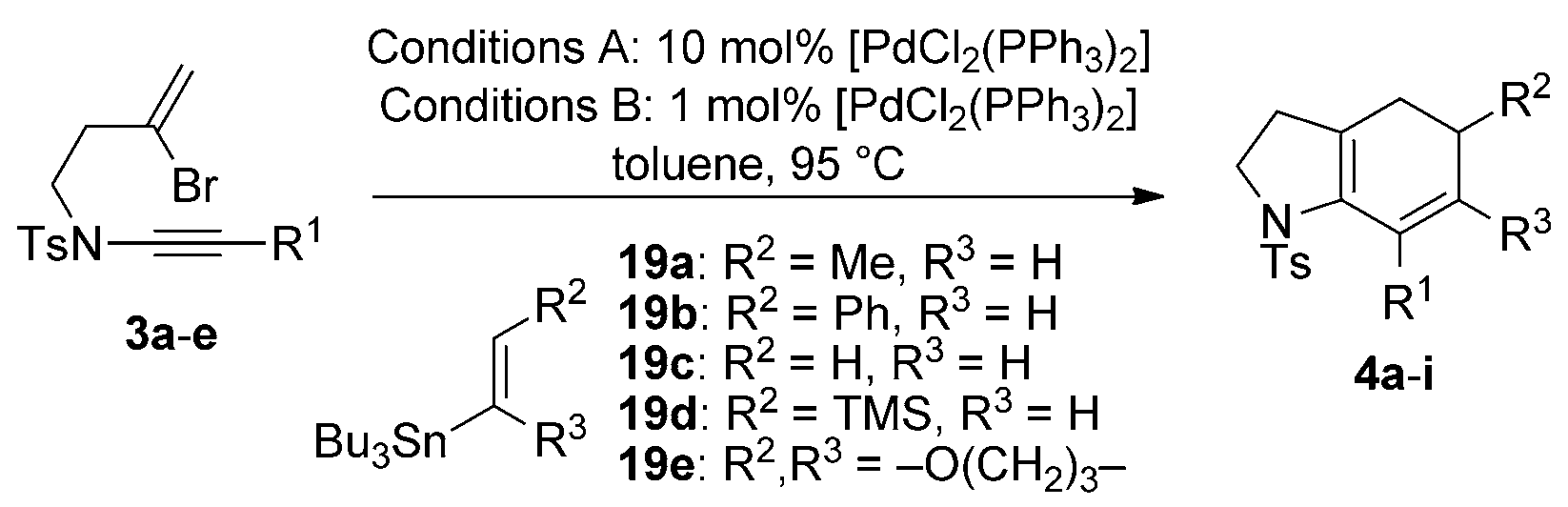
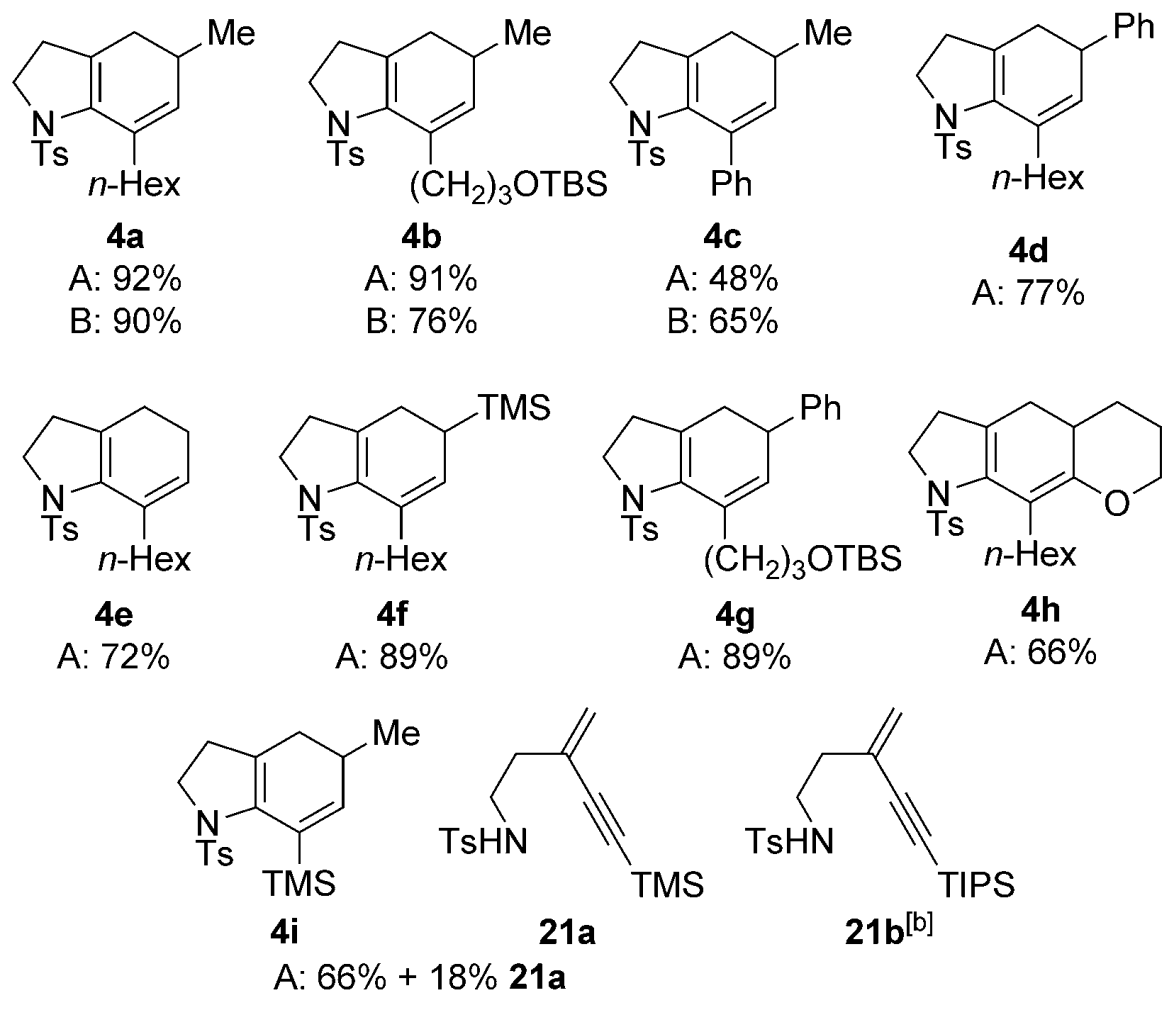

[a] Couplings were carried out using the bromoenynamide (1.0 equiv) and stannane (1.6 equiv) with the indicated catalyst loading, in toluene (0.17 m) at 95 °C. Yields are isolated yields. [b] Yield not determined; **21 b** was isolated as a byproduct from attempted cyclization of **3 e**.

**Scheme 5:**
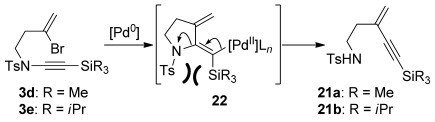
Possible mechanism for N-to-C alkyne transfer.

We were surprised by the failure of the TIPS-substituted ynamide **3 e** to undergo cyclization. This instead exclusively gave the N→C alkyne migration product **21 b**, a reaction that also occurred to an extent with the TMS-substituted ynamide **3 d**. The formation of these byproducts may proceed as shown in Scheme 5, in which the steric bulk of the silyl group leads to an intolerable increase in allylic strain upon carbopalladation (**22**); this may be relieved by an elimination of palladium and the amide leaving group (a retro-aminopalladation), with Pd^0^ regeneration by subsequent reaction of Pd^II^ with the stannane.

**Scheme 6:**
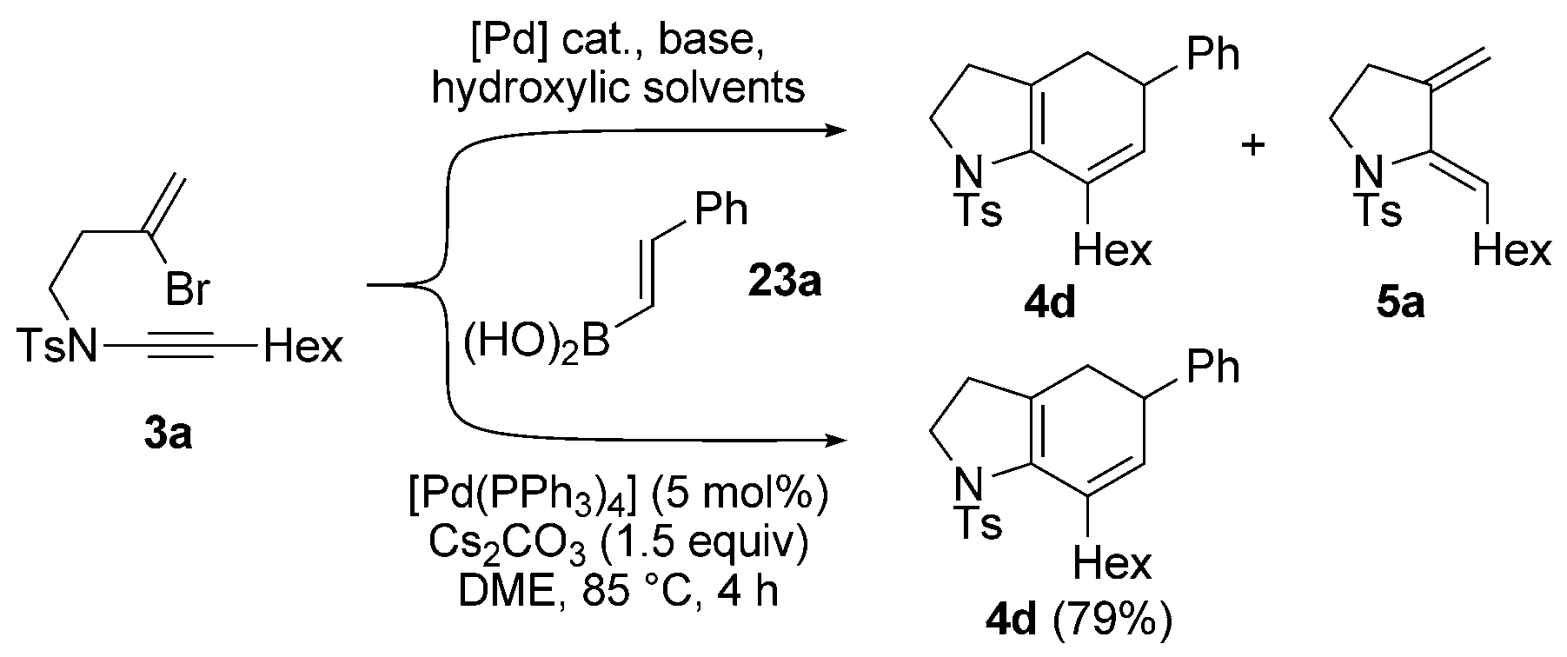
Solvent dependence of the Suzuki–Miyaura cascade cyclization.

Cascade cyclization by Stille cross-coupling is advantageous in that it employs air- and moisture-stable alkenylstannanes, and does not require an activator to promote transmetallation. However, due to the toxic nature of organotin compounds, we were eager to develop a Suzuki–Miyaura variant of this chemistry, which would hold significant appeal due to the low toxicity of vinylboronates and associated byproducts. On paper, this promised to be a straightforward exercise due to the related chemistry reported by Cossy, (aryl bromide carbopalladation onto a terminal ynamide/Suzuki coupling with an arylboronic acid, see Scheme 1). However, applying the conditions that had proven most effective in that work (5 mol % Pd(OAc)_2_, 10 mol % PPh_3_, THF/NaOH, reflux)[4a,c] to enynamide **3 a** and styrenylboronic acid **23 a** led, in addition to the desired product **4 d** (Scheme 6), to significant quantities of diene **5 a**, which seemed to have arisen by an unexpected reduction of the intermediate dienylpalladium(II) complex (see below).[28] Other conditions employed by Oh (10 mol % [Pd(PPh_3_)_4_], Cs_2_CO_3_, EtOH, 80 °C),[29] led to improved conversion, but did not suppress the formation of **5 a**. After some optimization, we found that the use of non-hydroxylic solvents avoided this problem,[28] with the combination of [Pd(PPh_3_)_4_] (5 mol %) and Cs_2_CO_3_ (1.5 equiv) in anhydrous DME at reflux affording **4 d** in 79 % yield.

Encouraged by these findings, the scope of this process was investigated with a wide range of ynamides and alkenylboronates (Table 4). What soon became apparent was that the efficiency of the cascade cyclization depended on the nature of the boronate. Firstly, the process was effective with 1,2-disubstituted alkenylboronates, but generally not with 1,1-disubstituted partners, which afforded mixtures of products. Secondly, alkyl-substituted alkenylboronic acids performed markedly better than the equivalent boronic esters; whereas an opposite trend was observed for aryl-substituted alkenylboronate derivatives. For instance, although alkyl-substituted alkenylboronic acids **23 b** and **23 c** afforded **4 j** and **4 k** in good yield from reaction with ynamide **3 a**, no reaction was observed using the equivalent pinacolboronic esters **24 b** or **24 c**; high yields were consistently obtained across a range of ynamides and alkyl-substituted alkenylboronic acids, including ynamides featuring more complex side chains (giving **4 n** and **4 o**). In contrast, reactions of aryl-substituted pinacolboronic esters outperformed the coupling of aryl-substituted alkenylboronic acids (**4 d**, **4 q**–**aa**); particularly notable is the successful formation of cyclopropyl derivative **4 q** from boronic ester **24 d**. Intriguingly, a catecholboronic ester proved a competent coupling partner for reaction of the trisubstituted boronic ester **25 e**, which gave **4 p** in respectable yield in spite of increased steric hindrance to transmetallation. We suspect that the contrasting substituent-dependent reactivity between alkenylboronic acids and esters depends on the ease of hydrolysis of the boronic ester: in the case of alkyl-substituted alkenylboronic esters, it would appear that slower hydrolysis prevents a productive reaction, potentially due to reduced electrophilicity of the boron atom compared to alkenylboronic esters with aryl substituents.

**Table 4:** Cyclizations of bromoenynamides under Suzuki–Miyaura conditions^[a]^

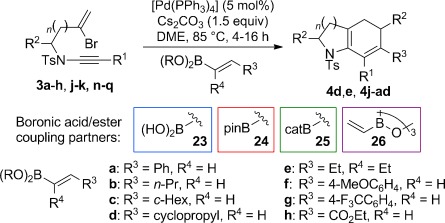
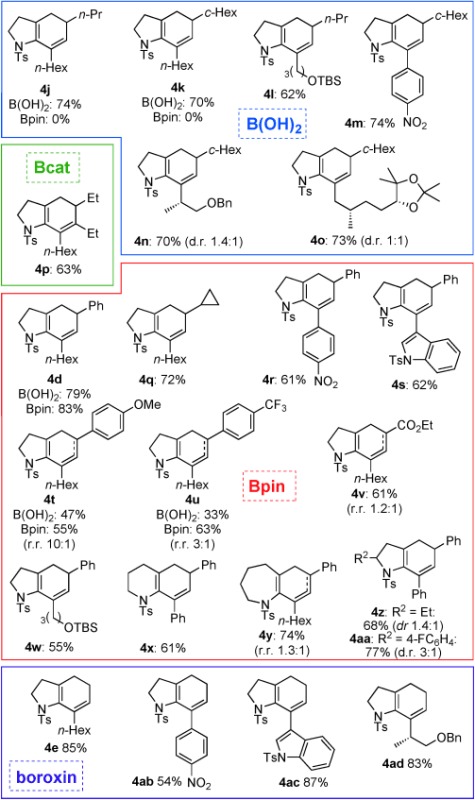

[a] Reactions were carried out using the bromoenynamide (1.0 equiv) and boronic acid derivative (1.5 equiv) in dimethoxyethane (0.17 m) at 85 °C. Yields are isolated yields.

In certain cases (**4 t**–**v**, **4 y**), a significant amount of regioisomeric 1,4-cyclohexadiene was observed along with the expected 1,3-cyclohexadiene product. Although in the case of **4 v** this isomerization could be rationalized through an ester enolization pathway, this seems unlikely for other products. An alternative might involve an isomerization mediated by a palladium(II) hydride intermediate, as is often observed in Heck reactions when reductive elimination of palladium(II) hydride complexes is slow. Such intermediates would be consistent with the observation of monocyclic dienes (**5**) encountered during reaction optimization (see below for further discussion of this process). Finally, the vinylboroxin trimer **26**, which serves as a stable precursor to vinylboronic acid itself, underwent efficient coupling to give the monosubstituted bicyclic cyclohexadiene products **4 e** and **4 ab**–**ad**. These reactions required an equivalent of water to assist boroxin hydrolysis.

Despite the demonstration of wide substrate scope for the Suzuki–Miyaura cascade with alkenylboronic acids and esters, it was clear that we might yet improve on several aspects of this reaction. This included relatively long reaction times, the variability in reaction efficiency between alkenylboronate derivatives[30] (depending on the nature of the other alkene substituents), the need for excess boronate coupling partner (typically 1.5 equivalents), and for anhydrous conditions to avoid formation of monocyclic dienamide products (**5**). More generally, boronic acids and esters can suffer from drawbacks such as problematic purification and/or oligomerization or decomposition on storage.[7] We were therefore attracted to the extensive work of the Molander[7a,c] and Lloyd–Jones groups[31] on the development of practical methods for the synthesis, and controlled hydrolysis/cross-coupling of bench-stable potassium alkenyltrifluoroborate salts, and decided to examine these increasingly popular reagents to improve the practicality of the cascade. Given that poor activation or slow transmetallation of the boronate could account at least in part for some of the reduced reactivity observed with certain boronate species (and indeed the overall timescale of the reaction), we felt that the more predictable hydrolysis pathways displayed by these trifluoroborate salts[31b] might improve the coupling sequence.

Surprisingly, as we had found with the alkenylboronic acid/ester cross-coupling cascade, most of the reported reaction conditions for sp^2^–sp^2^ coupling of trifluoroborate salts proved ineffective (Table 5).[7] For example, Molander’s conditions for alkene–alkene coupling[32] (Table 5, entry 1) using alkenylboronate **27 a** led to a complex mixture of products, including **5 a**. Conditions developed for vinylboronate-aryl halide coupling (entries 2, 3) were also unsuccessful.[33, 34] A further set of conditions that had been employed for this purpose[35] and for aryl–aryl coupling[31a] provided the first hints of success (entry 4): complete consumption of starting material was observed, but the desired product **4 j** was isolated as only the minor component of a 9:1 mixture with diene **5 a**. Reaction conditions we had employed for the Suzuki–Miyaura cascade (entries 5–7) did not offer improvement, giving only **5 a**. We also found that ‘ligand-free’ aryl–aryl coupling conditions proved ineffective (entry 8).[36]

**Table 5:** Optimization of the Suzuki-Molander cascade cyclization^[a]^

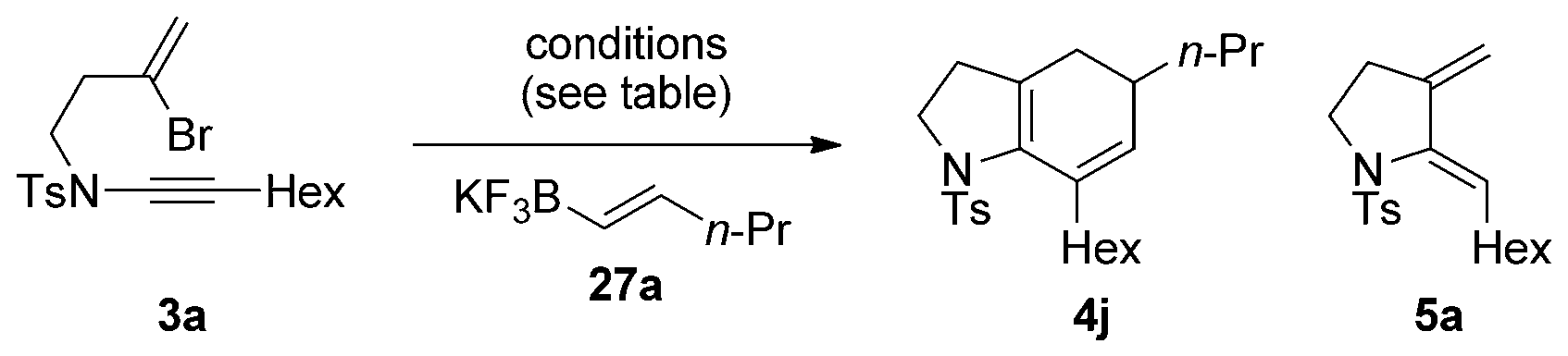					
Entry	Cat. [mol %]	Base [equiv]	Solvent,^[b]^ *T* [°C], *t* [h]	4 j/5 a(yield [%])^[c]^	ref.
1	Pd(OAc)_2_ (5) PPh_3_ (10)	Cs_2_CO_3_ (3)	THF/H_2_O, 85, 16	–^[d]^	[32]
2	[PdCl_2_(dppf)] (5)	Et_3_N (3)	*n*PrOH, 85, 16	–^[d]^	[33]
3	[PdCl_2_(dppf)] (5)	*t*BuNH_2_ (3)	*i*PrOH/H_2_O, 85, 16	–^[d]^	[34]
4	[PdCl_2_(PPh_3_)_2_] (10)	Cs_2_CO_3_ (3)	THF/H_2_O, 70, 16	1:9 (n.d.)	[31a, 35]
5	[Pd(PPh_3_)_4_] (10)	Cs_2_CO_3_ (2)	DME, 85, 2	0:1 (75)	[5a]
6	[Pd(PPh_3_)_4_] (10)	Cs_2_CO_3_ (2)	THF, 85, 2	0:1 (n.d.)	[5a]
7	[Pd(PPh_3_)_4_] (10)	Cs_2_CO_3_ (2)	THF, 3 equiv H_2_O, 85, 2	0:1 (n.d.)	[5a]
8	Pd(OAc)_2_ (5)	K_2_CO_3_ (3)	MeOH, 70, 16	–^[d]^	[36]
9	[Pd(PPh_3_)_4_] (10)	TMSCl/H_2_O (3)	MeCN, 85, 2	–^[e]^	[37]
10	[Pd(PPh_3_)_4_] (10)	LiOH (4)	DME/H_2_O, 85, 2	–^[e]^	[37]
11	[Pd(PPh_3_)_4_] (10)	LiOH (4)	DME/H_2_O, 85, 2	3:1 (79)^[f]^	
12	[Pd(PPh_3_)_4_] (10)	LiOH (4)	THF/H_2_O, 85, 2	3:1 (72)^[f]^	
13	[Pd(PPh_3_)_4_] (10)	LiOH (4)	MeCN/H_2_O, 85, 2	1:0 (80)^[e]^	
**14**	**[Pd(PPh_3_)_4_] (5)**	**LiOH (4)**	**MeCN/H_2_O, 85, 2**	**1:0 (88)**^[e]^	

[a] Reactions were carried out using the bromoenynamide (1.0 equiv) and potassium trifluoroborate salt (1.5 equiv) in the stated solvent (0.17 m). [b] Mixed solvent systems are a 10:1 ratio of solvent/H_2_O unless stated otherwise. [c] Yields are isolated yields; n.d.=not determined. [d] Complex mixture of products. [e] Complex mixture of products; the trifluoroborate salt and additive (TMSCl/H_2_O or LiOH) were premixed for 1 h before addition of substrate and catalyst. [f] Addition of LiOH to a solution of substrate, trifluoroborate salt, and catalyst. Isolated yield of mixture for entries 11 and 12. dppf=[1,1′-bis(diphenylphosphino)ferrocene].

Suspecting that inconsistent or insufficient rates of hydrolysis of the vinyl trifluoroborate salt might be contributing to these reactivity problems,[31b] conditions reported by Hutton to effect aryltrifluoroborate hydrolysis (to boronic acids) were examined (TMSCl/H_2_O, Table 5, entry 9, or LiOH in DME/H_2_O, entry 10).[37] Whilst pre-stirring the trifluoroborate salt and base, and then adding substrate and catalyst (as reported by Hutton, which presumably affords a reasonable proportion of the intermediate alkenylboronic acid before reaction initiation) again led to a complex mixture of products, we were delighted to find that modification of these conditions finally delivered a successful coupling: addition of LiOH to a mixture of substrate, trifluoroborate salt and catalyst (avoiding pre-mixing of base and boronate) effected rapid and complete conversion of **3 a** to **4 j** (entry 11). A brief survey of solvents (entries 11–13) showed that these reaction conditions were most effective in either DME/H_2_O or MeCN/H_2_O. Furthermore, the catalyst loading could be reduced to 5 mol %, with the reaction reaching completion within two hours. Although the reasons for this dramatic increase in reaction rate compared to boronic acids or esters remains unclear, it is notable that this protocol proved highly reproducible, and no longer required anhydrous, degassed solvents or an inert atmosphere. It is also notable that this process represents the first use of a trifluoroborate salt in any cascade carbopalladation/cross-coupling sequence,[8] and thus augments the synthetic opportunities of this rich field of chemistry.

Further optimization to these conditions revealed that only 1.1 equivalents of the trifluoroborate were required to effect the cascade process with high efficiency, and using these optimized conditions, several ynamides and alkenyltrifluoroborate coupling partners were examined in the Suzuki-Molander cascade (Table 6). Where DME/H_2_O proved a suitable solvent for coupling of the unsubstituted ‘parent’ vinylboronate **27 b**, appreciable levels of byproduct diene **5 a** were observed with alkyl-substituted alkenyltrifluoroborates. However, the use of MeCN/H_2_O as the solvent afforded the desired bicyclic products in all cases, giving a range of dihydroindolines in good yields; of additional note is the successful coupling of **3 k** to form dihydrobenzodiazepine **4 af**, which we could only effect under these Molander–Suzuki conditions, and not using organotin or boronic acid/ester coupling partners (with conditions as described above).

**Table 6:** Bromoenynamide cyclization under Suzuki-Molander conditions^[a]^

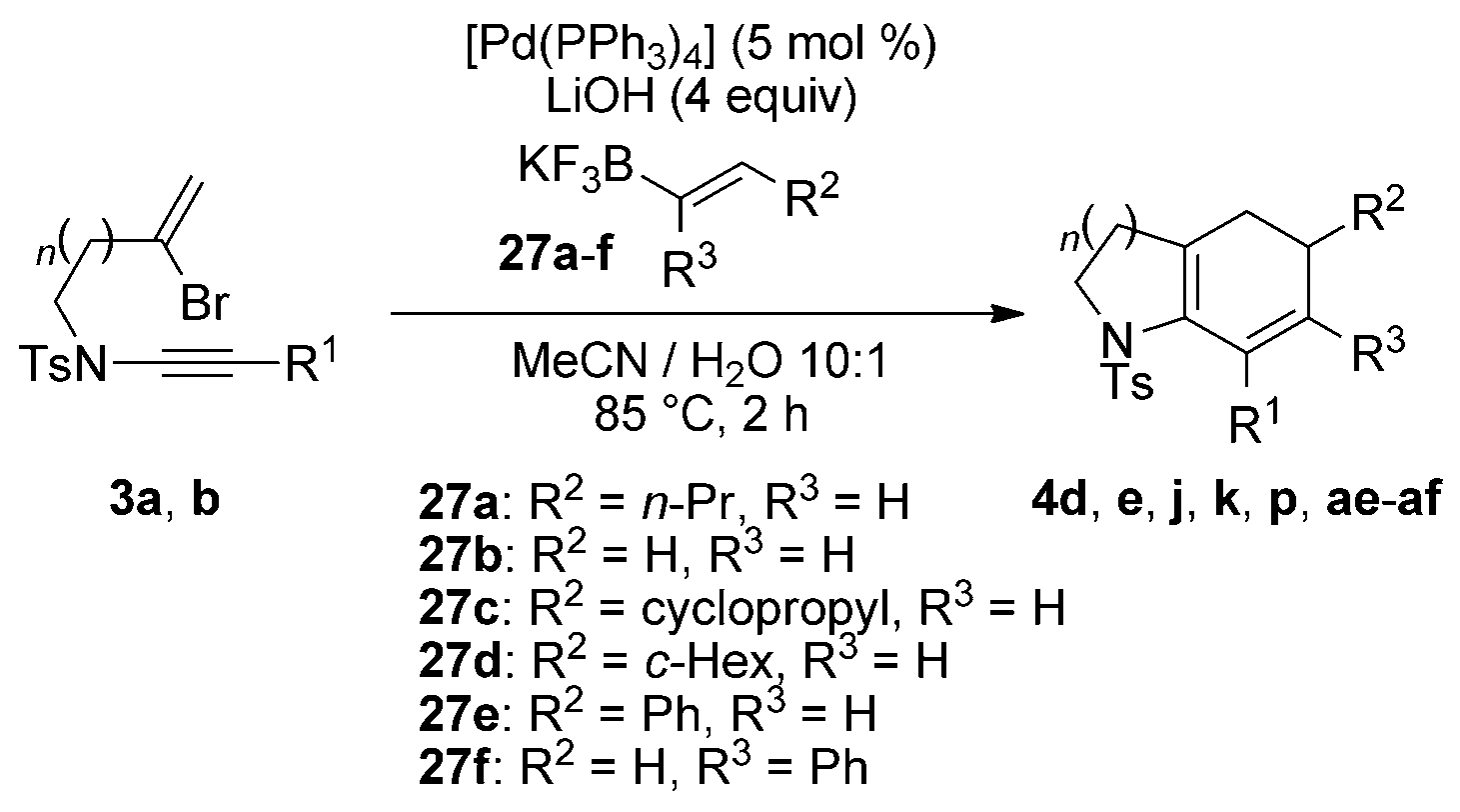
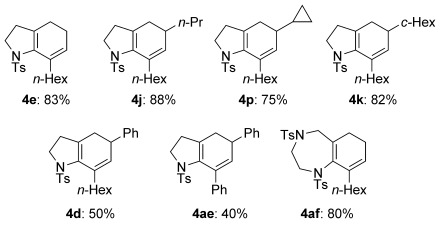

[a] Couplings were carried out using the bromoenynamide (1.0 equiv) potassium trifluoroborate salt (1.1 equiv) in MeCN/H_2_O (10:1, 0.25 m). Yields are isolated yields.

**Scheme 7:**
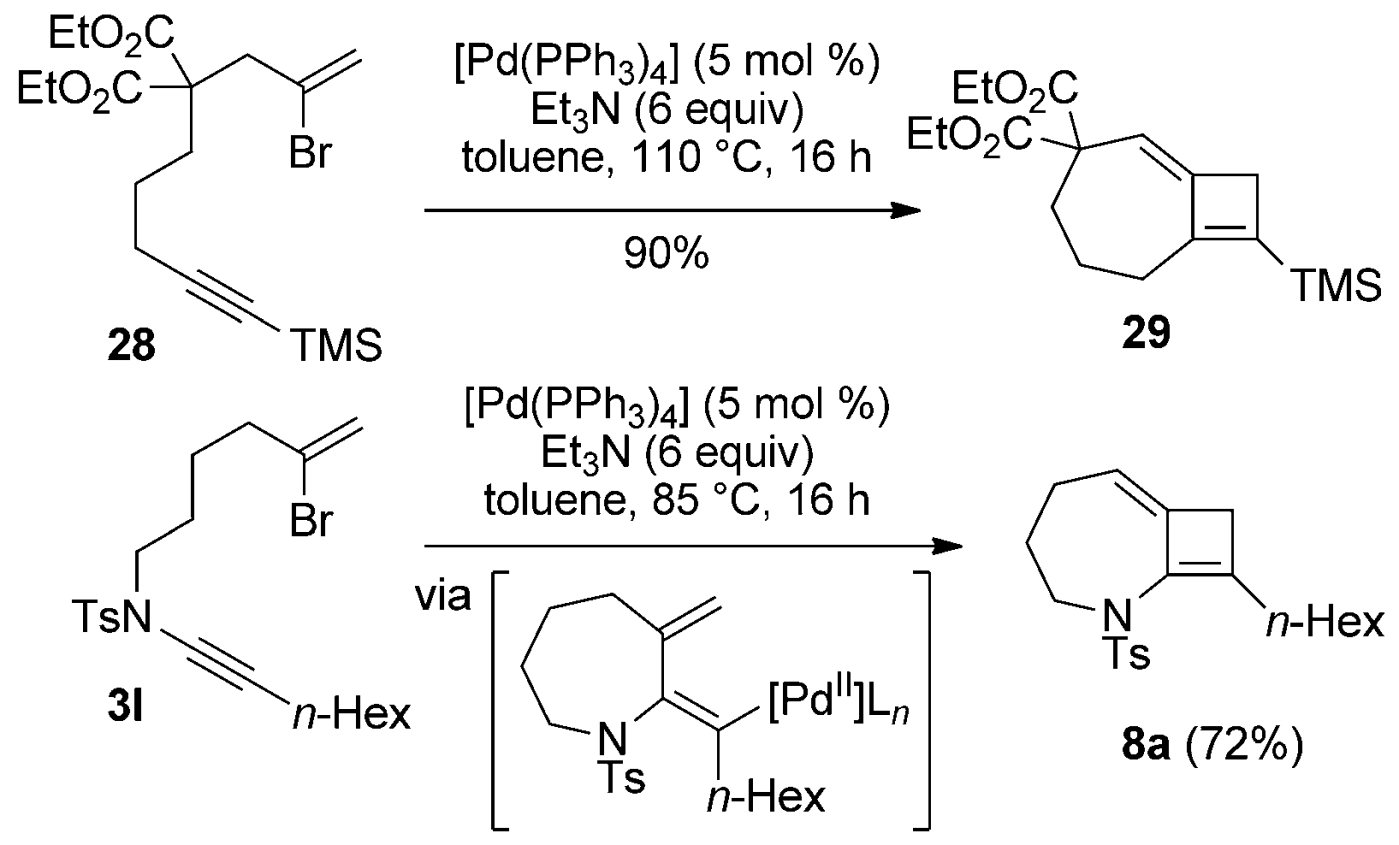
Formal 4-*endo*-*trig* cyclization of 3 l to 7,4-fused azabicycle 8 a.

As an aside to these collected cyclization efforts, we had noted that reactions of certain substrates (e.g. **3 l**) to give seven-membered ring products led to the formation of varying amounts of a byproduct featuring components of neither the boronate or stannane coupling partner, nor the characteristic alkene protons of the reductive cyclization product (**5**). We soon recognized that this byproduct bore a strong spectroscopic resemblance to similar compounds produced under the cyclization of related bromoenynes (e.g. **28→29**, Scheme 7).[10] The byproduct indeed turned out to be the 7,4-fused ring azabicycle **8 a**, which represents a rather unusual azacyclic scaffold. Compound **8 a** is the formal product of a 4-*endo*-*trig* carbopalladation, corresponding overall to a Heck reaction—pleasingly, application of the Heck-type conditions used in our previous bromoenyne work[10] to ynamide **3 l** gave an excellent yield of azacycle **8 a** (72 %), a product that further emphasises the structural diversity that can be achieved from palladium-catalyzed reactions of bromoenynamides.

Throughout the chemistry discussed above, a significant problem during our optimizations had been the formation of monocyclic dienamides (e.g. **5 a**). We recognized the potential of these as useful Diels–Alder substrates, and subsequently optimized this reductive cyclization, in which the alcohol solvent acts as a hydride source. Whilst details of the substrate scope of this reaction have been described,[9] a fuller discussion of the development of conditions for this reaction is included here, (e.g. dependence on the nature of the base, alcohol, and substrate, including different ring sizes), and applications to yne-hydrazides.

**Figure 3:**
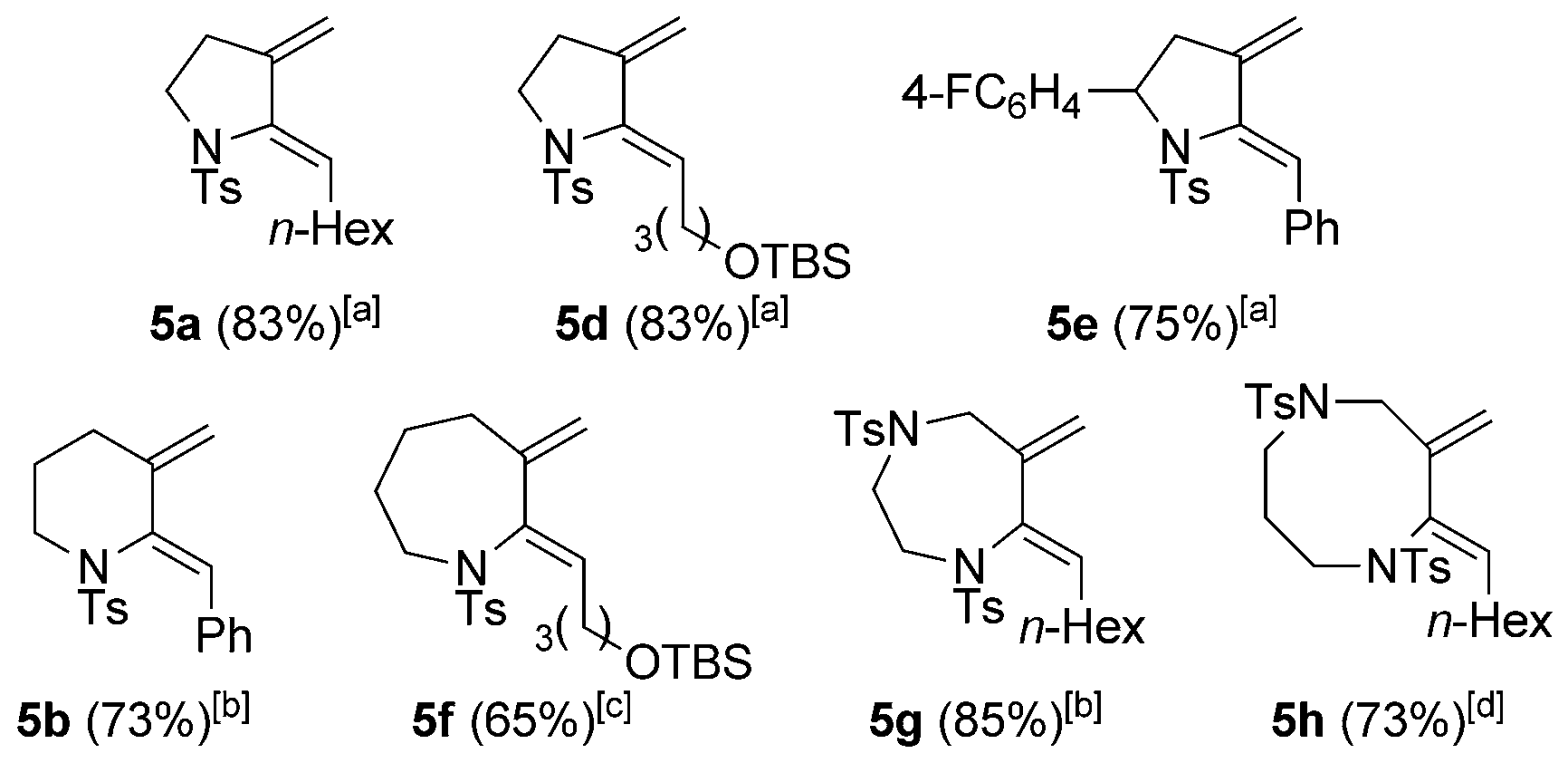
Representative examples of reductive cyclization products. [a] 2.5 mol % [Pd(PPh_3_)_4_], Cs_2_CO_3_, EtOH, 80 °C; [b] 2.5 mol % [Pd(PPh_3_)_4_], K_2_CO_3_, toluene/EtOH (1:1), 80 °C; [c] 10 mol % [Pd(PPh_3_)_4_], K_2_CO_3_, toluene/EtOH (10:1), 80 °C; [d] 10 mol % [Pd(PPh_3_)_4_], NaHCO_3_, EtOH, 80 °C.

A selection of conditions tested to effect this cyclization is shown in Table 7. Clearly, the success of the reaction depends crucially on the rate of carbopalladation (i.e. ring formation) versus alkoxide coordination/hydride transfer to palladium (and subsequent reductive elimination); premature hydride transfer to the metal would lead to direct reduction of the C–Br bond to give enynamides **30**. Initial reaction optimization showed that cesium carbonate in ethanol was highly effective for cyclizations to form five-membered rings (Table 7, entries 1–3), but that application to larger ring sizes (**3 p** and **3 k**) led to appreciable amounts of **30 b** or **30 c** (entries 4, 5). In an attempt to reduce the extent of direct reduction, a mixed toluene/ethanol solvent system was trialled, which pleasingly resulted in significantly less **30 c**, albeit at a cost of extended reaction time (entry 6). The nature of the alcohol might be expected to have an effect if alkoxide complexation and/or β-hydride elimination is rate-influencing, and indeed a significant variation in product ratio (**5 c**/**30 c**) was observed (entries 7–10), with methanol leading to enhanced formation of **30 c**, but cyclic alcohols reducing the formation of this side product. The nature of the base also proved significant (entries 11, 12), suggesting that alkoxide nucleophilicity is of importance in the reaction cycle; the use of potassium carbonate, or sodium bicarbonate, led to slower reactions, but much improved product ratios. Potassium carbonate in mixed toluene/ethanol solvent systems provided the ideal balance of basicity and reaction rate, and enabled the formation of six- and seven-membered rings with no direct reduction (entries 13–15). Using these optimized conditions, a wide selection of monocyclic dienes could be formed; some representative examples are shown in Figure 3.

**Table 7:** Summary of conditions for bromoenynamide reductive cyclization^[a]^

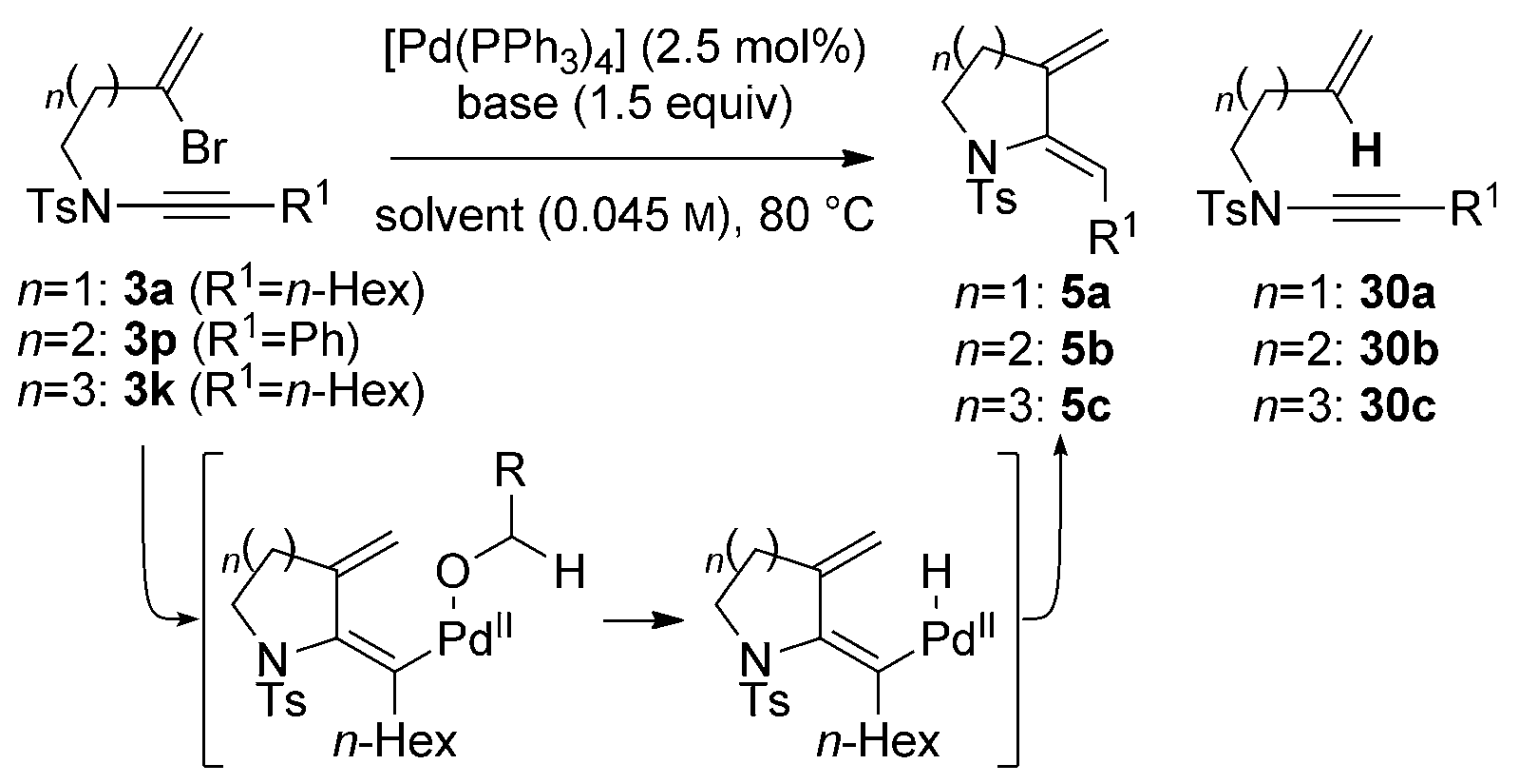						
Entry	Substrate	Base	Solvent	*t* [h]	Yield [%]	5/30^[b]^
1	**3 a**	Cs_2_CO_3_	EtOH^[a]^	1	83	1:0
2	**3 a**	Cs_2_CO_3_	EtOH	0.25	**83**	**1:0**
3	**3 a**	K_2_CO_3_	EtOH	1	62	1:0
4	**3 p**	Cs_2_CO_3_	EtOH	0.5	71	6:1
5	**3 k**	Cs_2_CO_3_	EtOH	1	60	1:3.3
6	**3 k**	Cs_2_CO_3_	Tol/EtOH (10:1)	2	45	1:0.4
7	**3 k**	Cs_2_CO_3_	Tol/*i*PrOH (10:1)	2	68	1:0.2
8	**3 k**	Cs_2_CO_3_	Tol/MeOH (10:1)	1	52	1:1
9	**3 k**	Cs_2_CO_3_	Tol/cyclopentanol (10:1)	2	68	1:0
10	**3 k**	Cs_2_CO_3_	Tol/cyclohexanol (10:1)	2	62	1:0
11	**3 k**	K_2_CO_3_	EtOH	1.75	68	1:0.15
12	**3 k**	NaHCO_3_^[c]^	EtOH	4	**40^[d]^**	**1:0**
13	**3 k**	K_2_CO_3_	Tol/EtOH (10:1)	5	**30^[d]^**	**1:0**
14	**3 k**	K_2_CO_3_	Tol/EtOH (1:1)	3.5	**69**	**1:0**
15	**3 p**	K_2_CO_3_	Tol/EtOH (1:1)	1	**73**	**1:0**

[a] 0.02 m. [b] Determined by ^1^H NMR spectroscopic analysis of the crude reaction mixture. [c] 10 mol % catalyst. [d] Conversion, as judged by ^1^H NMR spectroscopic analysis of the crude reaction mixture.

**Scheme 8:**
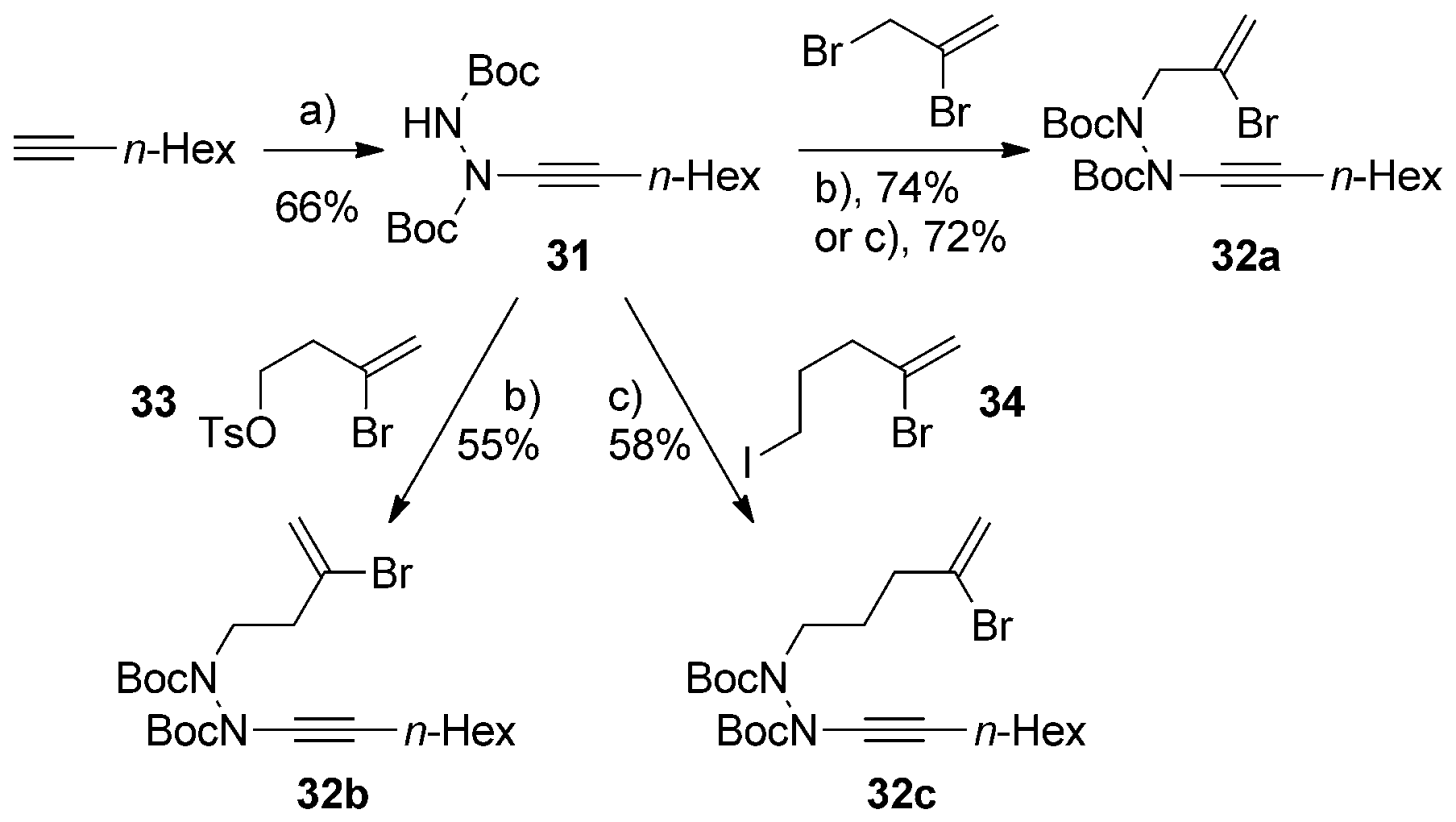
Synthesis of bromoenynhydrazides 32 a–c: a) *n*BuLi, THF, −78 °C; BocN=NBoc, −78 °C→RT; b) *n*Bu_4_NHSO_4_ (0.1 equiv), *n*Bu_4_NI (0.1 equiv), NaOH, K_2_CO_3_, toluene, 2 h; c) *n*Bu_4_NHSO_4_ (0.1 equiv), *n*Bu_4_NI (0.1 equiv), NaOH (25 % aq.), toluene, 1 h.

During our work, a report of yne-hydrazide synthesis from Beveridge and Batey[38] aroused our interest in the potential of these unusual alkyne derivatives to undergo equivalent cyclizations; indeed, our work on ynamide cycloisomerization[39] had shown that ynhydrazides can undergo productive cyclization processes. We recognized that reaction of an ynhydrazide (generated from addition of an alkyne to an azodicarboxylate, Scheme 8) with bromoalkene-containing electrophiles could permit a rapid synthesis of bromoenynhydrazide cyclization substrates.[40] Pleasingly, bromoallylation of ynhydrazide **31** (formed in 66 % yield from octyne and di-*tert-*butylazodicarboxylate) under phase-transfer conditions[41] gave a good yield of bromoenynhydrazide **32 a**. Use of the homologous electrophiles **33** and **34** under similar alkylation conditions led to the bromoenynhydrazides **32 b** and **32 c** respectively.

**Scheme 9:**
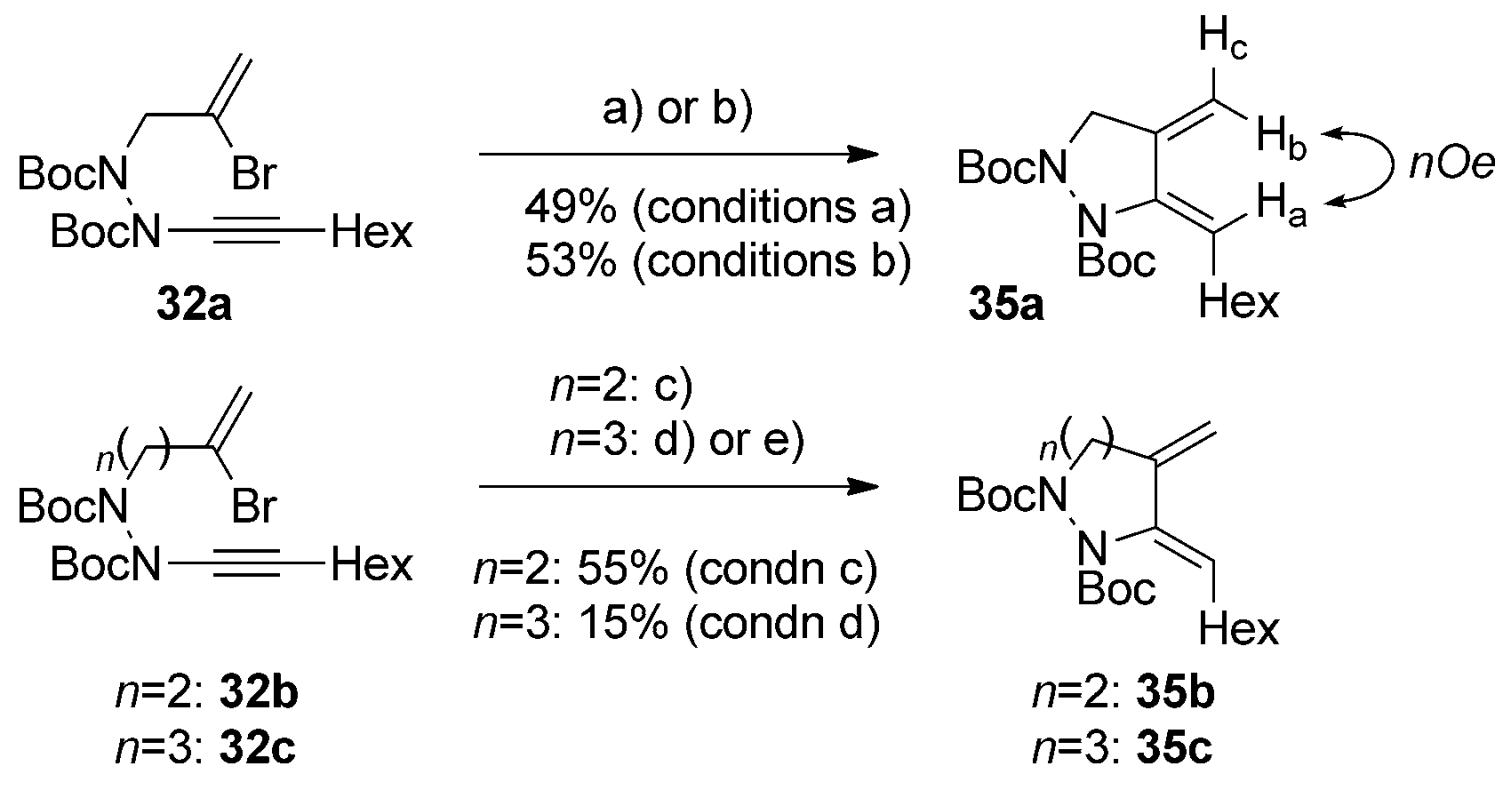
Reductive cyclizations of bromoenynhydrazides 32 a–c: a) [Pd(PPh_3_)_4_] (2.5 mol %), Cs_2_CO_3_, EtOH, 80 °C, 1 h; b) [Pd(PPh_3_)_4_] (2.5 mol %), K_2_CO_3_, toluene/EtOH (1:1), 80 °C, 22 h; c) as b), reaction time 0.5 h; d) as a), reaction time 1.5 h.

These ynhydrazides were tested with the range of cyclization conditions discussed above; however, success was met only with reductive cyclization to give dienes **35 a**–**c** in moderate yields (Scheme 9), with the stereochemistry assigned by ^1^H NMR nOe experiments. Cyclization to the seven-membered ring product **35 c** proved particularly challenging, and could only be realized in low yield using Cs_2_CO_3_ as a base. Despite the upfield chemical shifts of the alkene protons in dihydropyrazole diene **35 a** compared with 2-amidodiene **5 a**, and therefore implied increased electron density (*δ*_H_=(**35 a**, CDCl_3_) 5.61, 5.30 and 4.90 ppm for H_a_, H_b_ and H_c_; *δ*_H_=(**5 a**, CDCl_3_) 5.87, 5.20 and 4.64 ppm), these dienamides did not prove amenable to any subsequent Diels–Alder chemistry. Nonetheless, this concise route could yet provide a useful entry to substituted pyrazole frameworks.

In our previous work on the reductive cyclization (Table 7), we had explored the Diels–Alder reactivity of dienamides **5** using dienophiles such as α,β-unsaturated aldehydes and arynes.[9] We were keen to expand this study to hetero-Diels–Alder (HDA) reactions, which could give unusual heterobicyclic products, and would allow further study of the regioselectivity of cycloadditions of these 2-amidodienes. A range of heteroatomic dienophiles (**6 a**–**g**) were explored (Table 8), with all reactions proceeding at room temperature in dichloromethane; this represents a marked acceleration compared to the carbon-based dienophiles studied previously, which generally required prolonged heating (1–24 h) to achieve good levels of conversion.

**Table 8:** Dienamide Hetero-Diels–Alder reactions^[a]^

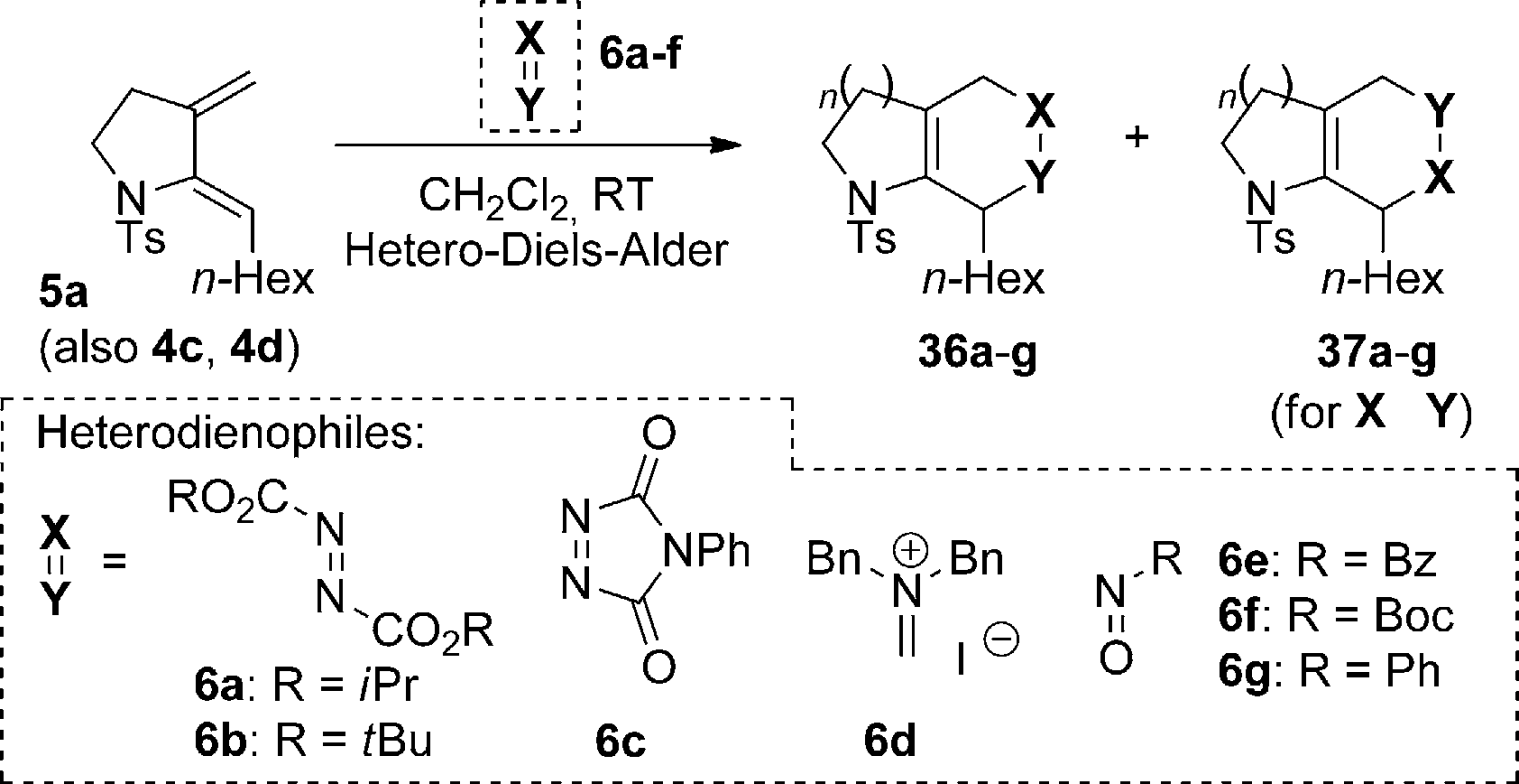					
Entry	Diene	Dienophile	Product	*t*	Yield [%] (36/37)
1 2	**5 a 5 a**	**6 a 6 b**	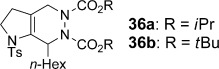	1.5 h 1.5 h	75 95
3	**5 a**	**6 c**	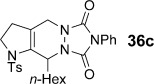	10 min	85
4	**5 a**	**6 d**	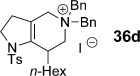	18 h	76
5	**5 a**	**6 e**	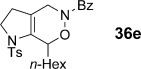	1 h	95
6	**5 a**	**6 f**	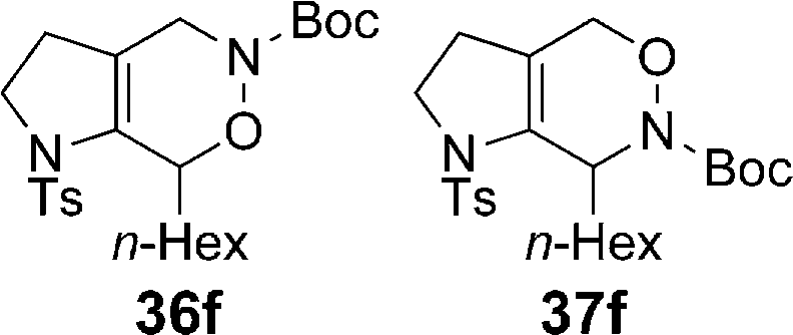	1 h	98 (1.8:1)
7	**5 a**	**6 g**	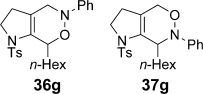	10 min	81 (3.7:1)
8	**4 a**	DDQ	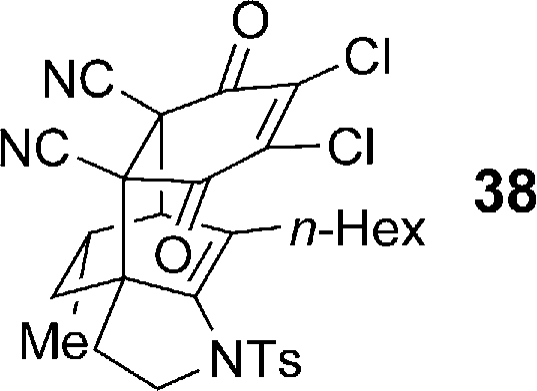	2 h^[b]^	62
9	**4 d**	**6 g**	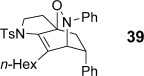	18 h	68
10	**5 a**	acrolein	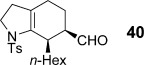	5 h^[c]^	69

[a] Reactions performed in CH_2_Cl_2_ at RT. [b] Reaction performed in toluene at RT. [c] Reaction performed in toluene at 80 °C.

We first examined symmetrical diimide dienophiles, and were pleased to find high-yielding, rapid reactivity for the formation of triazabicycles **36 a**–**c** on reaction with dienamide **5 a** (Table 8, entries 1–3). Of arguably greater interest was the reactivity and regioselectivity of non-symmetric dienophiles. This investigation began with the reaction of iminium ion **36 d** (entry 4), in which we were delighted to observe a high-yielding (if somewhat slower) cycloaddition to give the ammonium salt **36 d** as a single regioisomer. Nitroso compounds are an important heteroatomic dienophiles,[42] and here again we observed excellent reactivity with diene **5 a** by using *N*-Bz, *N*-Boc and *N*-Ph nitroso dienophiles **6 e**–**g** (entries 5–7, see regioselectivity discussion below).

**Figure 4:**
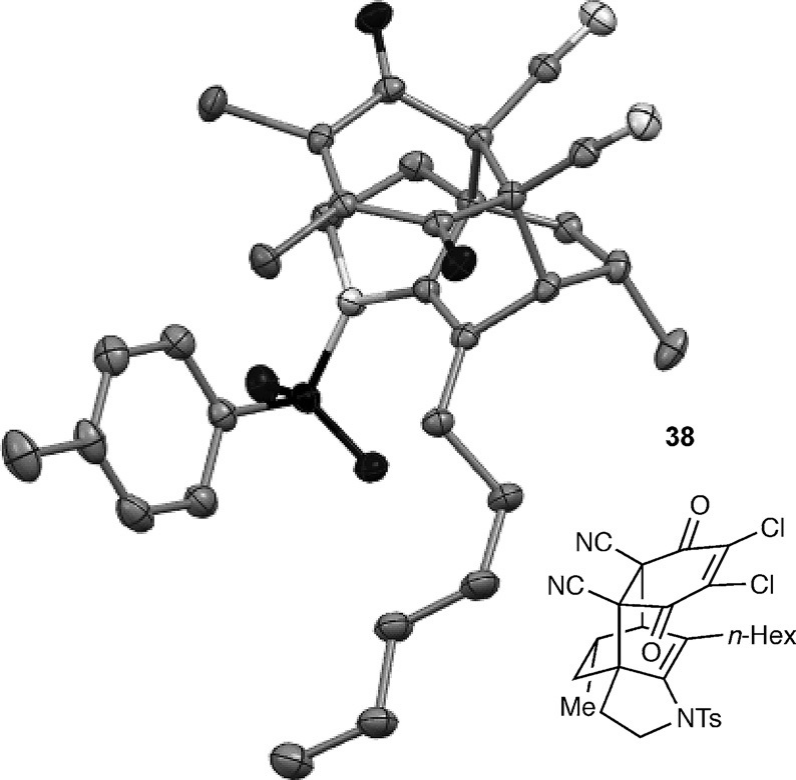
The structure of DDQ cycloadduct 38 as determined from single crystal X-ray diffraction. Displacement ellipsoids are drawn at 50 % probability and hydrogen atoms are omitted for clarity.

Bicyclic dienamide **4 d** was also examined as a substrate for this chemistry. Despite its increased steric hindrance, we suspected that a HDA reaction might be successful, as we had already observed a serendipitous cycloaddition between **4 a** and 2,3-dichloro-5,6-dicyano-1,4-benzoquinone (DDQ) during attempted oxidation of **4 a** to the indoline, giving product **38** as a single stereoisomer (Table 8, entry 8, structure determined by single-crystal X-ray diffraction, Figure 4). Accordingly, subjection of **4 d** to reaction with **6 g** indeed proved viable, affording a single regioisomer of cycloadduct **39** (entry 9).

**Figure 5:**
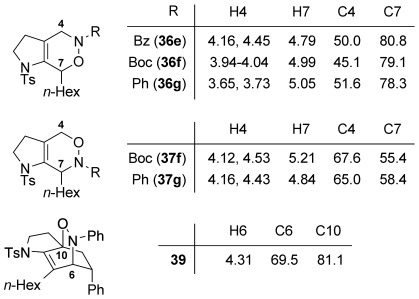
Comparison of ^1^H and ^13^C NMR spectroscopic chemical shifts (ppm, CDCl_3_) for nitroso-Diels–Alder cycloadducts.

The assignment of regio- and stereochemistry of these nitroso-Diels–Alder adducts was by no means trivial, but could be achieved by careful comparison of ^1^H and ^13^C chemical shifts between the two regioisomers (and spectroscopic comparisons with cycloadducts prepared in previous studies),[43] in combination with 2D NMR experiments (HMBC, HSQC). This revealed a general trend for the nitroso-Diels–Alder reactions whereby the proximal adduct **36** was favoured over the distal adduct **37**,[44] and indeed was formed as the sole product in the case of benzoyl nitroso cycloaddition (**36 e**, Table 8, entry 5). ^1^H and ^13^C NMR spectroscopic chemical shifts are shown in Figure 5 which support these assignments; the latter data is particularly consistent between the different cycloadducts.

This regioselectivity is as we might have expected on the basis of previous investigations,[42, 43] and is thought to arise from frontier molecular orbital effects (i.e., the HOMO coefficient of the diene **5 a** is largest on the *exo-*methylene carbon atom due to the influence of the *n*-hexyl and C3 alkyl substituents; bond formation has been shown to be advanced at this carbon atom in the cycloaddition transition state and favours formation of the proximal isomer **36**).[43b] Indeed, our previous experiments with acrolein and other dienophiles had shown high regioselectivity for the formation of the 1,2-disubstituted isomer **40** (Table 8, entry 10),[9] which mirrors this regioselectivity. It is notable that reaction of iminium ion **6 d**, which is the slowest of the cycloadditions, in fact shows a reversal of regioselectivity (based on dienophile polarization) compared to acrolein or the nitroso dienophiles: this might be rationalized by the positioning of the rather hindered nitrogen substituent in the sterically least demanding position, or perhaps by a more asynchronous mechanism in which the sulfonamide nitrogen atom plays a cation-stabilizing role.[45] In any case, it seems that a balance of electronic and steric factors governs the ratio of cycloadducts.

**Scheme 10:**
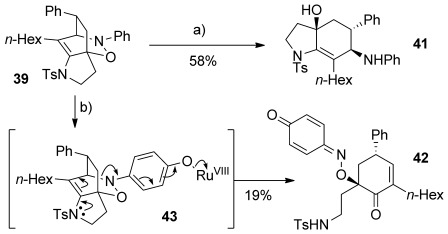
a) [Mo(CO)_6_], NaBH_4_, MeCN/H_2_O (7:1), 90 °C, 20 h; b) NaIO_4_, RuCl_3_ (2.5 mol %), EtOAc/MeCN/H_2_O (2:2:1), 2 h.

Finally, we were able to show that tricyclic product **39** could be further elaborated (Scheme 10). Treatment of **39** with [Mo(CO)_6_][46] led cleanly to the functionality-rich bicyclic product **41**. However, attempted C=C bond cleavage using ruthenium tetroxide,[47] which we expected to afford a spirocyclic product, instead gave cyclohexenone **42**. This surprising result could be rationalized by an oxidative fragmentation pathway (**43**, formed from *p*-oxidation of the aniline ring), perhaps driven by a relief of ring strain, or steric hindrance of the C=C bond towards oxidation.

## Conclusion

In conclusion, we have developed a number of routes for the preparation of bromoenynamides from simple, commercial starting materials. The palladium-catalyzed cascade cyclization of these ynamides successfully employs alkenyl stannane, boronic ester, boronic acid, boroxin and trifluoroborate derivatives, with subsequent in situ electrocyclization providing a plethora of bicyclic azacycle products. In addition to this structural diversity, mechanistic insight into the cyclization process has been established, including the formation of an unusual 7,4-fused azacycle scaffold. Further observations on the Diels–Alder reactivity of exocyclic amidodienes formed through a reductive cyclization reveals subtle electronic effects in HDA reactions, as well as affording novel azabicyclic frameworks. We anticipate that these collected methods could provide useful and general routes to such bicyclic systems.

## Experimental Section

### General methods

General procedures for the various cascade cyclizations are given. For specific experimental procedures for novel compounds, further experimental details, and characterization (including copies of ^1^H and ^13^C NMR spectra), see the Supporting Information.

### General procedures

**Stille cascade cyclisation of bromoenynamides**: A degassed (Ar bubbling, 15 min) solution of the bromoenynamide (1.0 equiv) and stannane coupling partner (1.6 equiv) in toluene (16.7 mL mmol^−1^) was added to a reaction vessel containing [PdCl_2_(PPh_3_)_2_] (1 or 10 mol %) under Ar. The reaction mixture was then heated to 95 °C under Ar until the reaction was judged complete by TLC analysis (4–24 h). The reaction was then cooled to RT and concentrated. Purification by flash chromatography (EtOAc/petroleum ether eluent) afforded the bicyclic dienamide product.

**Suzuki cascade cyclisation of bromoenynamides**: A degassed (Ar bubbling, 15 min) solution of the bromoenynamide (1.0 equiv) and vinylboronate (1.5 equiv) in DME (16.7 mL mmol^−1^) was added to a reaction vessel containing [Pd(PPh_3_)_4_] (5 mol %) and Cs_2_CO_3_ (1.5 equiv) under Ar. The reaction mixture was heated to reflux under Ar until the reaction was judged complete by TLC analysis. The reaction was then cooled to RT and concentrated; purification by flash chromatography afforded the bicyclic dienamide product.

**Reductive cyclization of bromoenynamides to pyrrolidines and piperidines (*n*=1, 2)**: [Pd(PPh_3_)_4_] (2.5 mol %) and Cs_2_CO_3_ (1.5 equiv) was added to a degassed (Ar bubbling, 15 min) solution of bromoenynamide (1.0 equiv) in EtOH (0.045 m). The reaction mixture was heated to 80 °C until judged complete by TLC analysis, then it was cooled to RT and filtered through a Celite plug (EtOAc eluent). The filtrate was concentrated, and the residue purified by column chromatography to afford the corresponding exocyclic diene product.

**Reductive cyclization of bromoenynamides to piperidines (*n*=2) and azepanes (*n*=3)**

*Method A*: [Pd(PPh_3_)_4_] (10 mol %) and K_2_CO_3_ (1.5 equiv) were added to a degassed (Ar bubbling, 15 min) solution of bromoenynamide (1.0 equiv) in toluene/EtOH (10:1, 0.045 m). The reaction mixture was heated to 80 °C until judged complete by TLC (3×TLC runs on the same plate in 20:1 petroleum ether/EtOAc, to give separation of SM and product). The reaction was then cooled to RT and filtered through a Celite® plug (EtOAc eluent). The filtrate was concentrated, and the residue purified by flash chromatography to afford the corresponding exocyclic diene.

*Method B*: [Pd(PPh_3_)_4_] (2.5 mol %) and K_2_CO_3_ (1.5 equiv) were added to a degassed (Ar bubbling, 15 min) solution of the appropriate bromoenynamide (1.0 equiv) in toluene/EtOH (1:1, 0.045 m). The reaction mixture was heated to 80 °C until the reaction was judged complete by TLC (3×TLC runs on the same plate in 20:1 petroleum ether/EtOAc to give separation). The reaction was then cooled to RT and filtered through a Celite plug (EtOAc eluent). The filtrate was concentrated, and the residue purified by flash chromatography to afford the corresponding exocyclic diene.

**Suzuki–Molander cascade cyclization of bromoenynamides**: A mixture of bromoenynamide (1 equiv), potassium trifluoroborate salt (1.1 equiv), LiOH (4 equiv) and [Pd(PPh_3_)_4_] (5 mol %) in MeCN/H_2_O (10:1) (5 mL mmol^−1^) was heated to 85 °C, and stirred rapidly for up to 3 h (until the reaction reached completion as judged by TLC). The mixture was then cooled to ambient temperature, diluted with Et_2_O (5 mL mmol^−1^) and NH_4_Cl (sat., aq.) was added. The product was extracted with Et_2_O (×2), dried (MgSO_4_) and concentrated, and the residue was purified by flash chromatography (Et_2_O/petroleum ether eluent) to afford the bicyclic dienamide product.

### Single-crystal X-ray diffraction

Raw frame data were collected at 150 K[47] with a Nonius K-CCD diffractometer and reduced using DENZO-SMN/SCALEPACK[48] as per the Supporting Information (CIF). The structure was solved with SIR92[49] and refined with CRYSTALS.[50] Full crystallographic data (in CIF format) are available as Supporting Information.

CCDC-1062627 http://www.ccdc.cam.ac.uk/cgi-bin/catreq.cgi(**38**) contains the supplementary crystallographic data for this paper. These data can be obtained free of charge from The Cambridge Crystallographic Data Centre via http://www.ccdc.cam.ac.uk/data_request/cif.

## Supporting Information

As a service to our authors and readers, this journal provides supporting information supplied by the authors. Such materials are peer reviewed and may be re-organized for online delivery, but are not copy-edited or typeset. Technical support issues arising from supporting information (other than missing files) should be addressed to the authors.

miscellaneous_information
