# Supplementary material for: Ynamide Carbopalladation: A Flexible Route to Mono-, Bi- and Tricyclic Azacycles
Source: Chemistry. 2015 Jul 16;21(36):12627–39. doi: 10.1002/chem.201501710 (PMC4557052; doi:10.1002/chem.201501710)
Supplement: Supplementary file 1 — miscellaneous_information [file chem0021-12627-sd1.pdf]

CHEMISTRY
A European Journal

Supporting Information

Ynamide Carbopalladation: A Flexible Route to Mono-, Bi- and Tricyclic Azacycles

Craig D. Campbell,[a] Rebecca L. Greenaway,[a] Oliver T. Holton ,[a] P. Ross Walker,[a] Helen A. Chapman,[b] C. Adam Russell,[b] Greg Carr,[c] Amber L. Thomson,[a] and Edward A. Anderson*[a]

chem_201501710_sm_miscellaneous_information.pdf

Ynamide carbopalladation: A flexible route to mono-, bi- and tricyclic azacycles

Craig D. Campbell,[a] Rebecca L. Greenaway,[a] Oliver T. Holton,[a] P. Ross Walker,[a] Helen A. Chapman,[b] C. Adam Russell,[b] Greg Carr,[c] Amber L. Thomson[a] and Edward A. Anderson*[a]

Chemistry Research Laboratory, 12 Mansfield Road, Oxford, OX1 3TA, UK

edward.anderson@chem.ox.ac.uk

SUPPORTING INFORMATION

1. General Experimental Considerations **2**

2. General Procedures **3**

3. Bromoenynamide Synthesis **7**

4. Stannane Synthesis **24**

5. Variable Temperature NMR Experiment (Stille coupling) **26**

6. Bromoenynamide Stille Cyclizations **28**

7. Bromoenynamide Suzuki-Molander Cyclizations **33**

8. Formal 4-endo-trig cyclization of 31 to 8a **36**

9. Bromoenynhydrazide Synthesis and Cyclizations **36**

10. Amidodiene Diels Alder reactions **43**

11. References **52**

12. Copies of ^{1}H and ^{13}C NMR spectra **53**

1. General experimental considerations

NMR Spectra: ^{1}H NMR spectra were recorded using an internal deuterium lock for the residual protons in $CDCl_3$ (δ 7.26) at ambient probe temperatures on the following instruments: Bruker AV400 (400 MHz), Bruker AMX 500 (500 MHz), Bruker DPX200 (200 MHz) or Bruker DPX250 (250 MHz). Data are presented as follows: chemical shift, peak multiplicity (s = singlet, d = doublet, t = triplet, q = quartet, quin = quintet, sex = sextet, m = multiplet, br = broad, app = apparent), coupling constants and interpretations. Chemical shifts are expressed in ppm on a δ scale relative to $SiMe_4$ (δ = 0 ppm) and coupling constants (*J*) are given in Hz. Assignments were made either on the basis of unambiguous chemical shift or coupling patterns, or by analogy to fully interpreted spectra for structurally related compounds. ^{13}C NMR spectra were recorded using an internal deuterium lock using solvents $CDCl_3$ (δ 77.0) at ambient probe temperatures on the following instruments: Bruker AV400 (100 MHz) or Bruker AMX 500 (125 MHz).

Infra-red spectra: Recorded on Perkin Elmer Paragon 1000 spectrometer with the sample prepared as a thin film between NaCl plates, or using a PIKE-Miracle Diamond /ZnSe ATR module.

Mass spectra: Accurate mass (HRMS) data was acquired using electrospray ionisation on a Bruker MicroTOF (resolution = 5000 FWHM) using tetraoctylammonium bromide as a lock-mass in both positive and negative ion modes, or via CI or FI on a Micromass GCT (resolution = 7000 FWHM) using isoamyl acetate as a lock-mass. High resolution values are calculated to 4 decimal places from the molecular formula, and all values are within a tolerance of 5 ppm.

Melting points: Obtained using a Reichert-Koffler block and are uncorrected.

Optical rotations: Performed on a Perkin Elmer 241 polarimeter using a Na lamp. [α] and c units are quoted as deg cm^3 g^{-1} dm^{-1} and g cm^{-3}, respectively. HPLC was carried out on an Agilent 1200 series running in normal phase under UV detection (λ = 254 and 220 nm) using a ZORBAX RX-SIL (150 mm × 4.6 mm ID) as the analytical column. Semi-preparative HPLC was carried out using a ZORBAX RX-SIL (250 mm × 9.4 mm ID).

Other techniques: Reactions were monitored by thin layer chromatography on pre-coated aluminium-backed plates (Merck Kieselgel 60 with fluorescent indicator UV254). Spots were visualized either by UV or by staining with $KMnO_4$ or vanillin solutions. Flash column chromatography was performed using silica gel 60 (0.040 – 0.063 mm) (MN Kieselgel 60M).

Materials: All reagents, obtained from Acros, Aldrich, Fluka, Lancaster, Strem and Fluorochem fine chemicals suppliers, were used directly as supplied or purified by the methods described by Amerago and Chai. All non-aqueous reactions were performed in oven dried apparatus under argon or nitrogen atmospheres, using distilled anhydrous solvents, at room temperature unless otherwise indicated.

2. General procedures

General Procedure A: *Barbier Allylation*

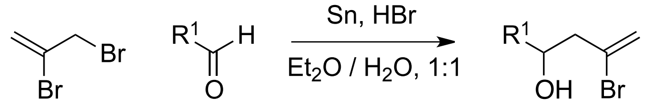

The procedure of Otera *et al.* was used.[1] To a mixture of tin powder (1.3 equiv.) and 2,3-dibromopropene (1.3 equiv.) in water / Et_2O (3 mL $mmol^{-1}$, 1:1) was added HBr (48% aq., several drops) and the appropriate aldehyde (1.0 equiv.). The mixture was stirred overnight at rt before being diluted with water and extracted with Et_2O. The combined organic layers were dried ($MgSO_4$) and concentrated to afford the alcohol product, which was used without further purification, or purified by flash chromatography (ether or EtOAc / petroleum ether) for the purposes of characterization.

General Procedure B: *Mitsunobu Coupling and N-Boc deprotection*

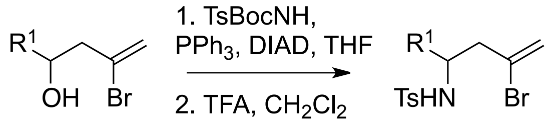

The procedure of Lu and Li was used.[2] Di-*iso*propylazodicarboxylate (1.5 equiv.) was added dropwise to a solution of triphenylphosphine (2.0 equiv.), TsNHBoc (1.3 equiv.) and the appropriate alcohol (1.0 equiv.) in THF (3 mL $mmol^{-1}$) at 0 °C. The reaction was stirred overnight at rt, before being concentrated. The residue was redissolved in Et_2O and then sonicated for 5 min; the resulting precipitate (triphenylphosphine oxide) was removed by filtration. To the filtrate was added hexanes, which was further sonicated for 5 min, and the resulting precipitate removed by filtration, before being concentrated. Purification of the residue by flash chromatography (typically petroleum ether / EtOAc, 20:1) afforded the *N*-Ts-*N*-Boc amide derivative. This was dissolved in CH_2Cl_2 (2 mL $mmol^{-1}$), then TFA (0.5 mL $mmol^{-1}$) was added dropwise at 0 °C. The reaction mixture was stirred for (up to) 12 h at rt before being cooled to 0 °C, and slowly quenched by the dropwise addition of $NaHCO_3$ (sat., aq.). The organic layer was separated, and the aqueous layer extracted with additional CH_2Cl_2. The combined organic layers were dried ($MgSO_4$) and concentrated. Purification *via* flash chromatography (typically petroleum ether / EtOAc, 10:1→5:1) afforded the sulfonamide product.

General procedure C: *Bromination of alkynes via deprotonation with n-BuLi / quench with Br_2*

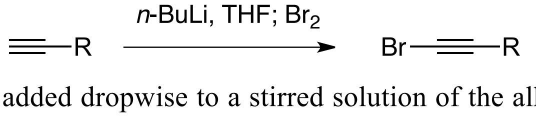

n-BuLi (2.5 M, 1.2 equiv.) was added dropwise to a stirred solution of the alkyne (1.0 equiv.) in THF (3 M) at −78 °C. The solution was stirred at −78 °C for 30 min, then bromine (1.4 equiv.) was added dropwise. The mixture was stirred for a further 15 min, before being quenched with $Na_2S_2O_3$ (sat., aq.) and then warmed to rt. The reaction was extracted with Et_2O, and the combined organic layers dried ($MgSO_4$) and concentrated to afford the corresponding bromoalkyne, which was generally used without further purification. In cases where volatile bromoalkynes were prepared, some residual THF remained in the concentrate.

General procedure D: *Silver-catalyzed bromination of alkynes with NBS*

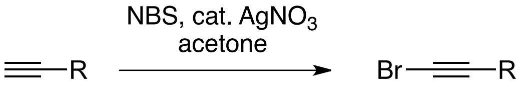

To a solution of terminal alkyne (1.0 equiv.) in acetone (1 mL $mmol^{-1}$) was added $AgNO_3$ (2.5 mol%). After stirring for 5 min, *N*-bromosuccinimide (1.1 equiv.) was added, and the mixture stirred for a further 4-15 h at rt until complete as judged by tlc. The mixture was then diluted with petroleum ether and filtered through a short silica pad (petroleum ether eluent). The resulting solution was concentrated, to obtain the corresponding bromoalkyne.

General Procedure E: *Preparation of bromoenynamides using the Hsung method*[3]

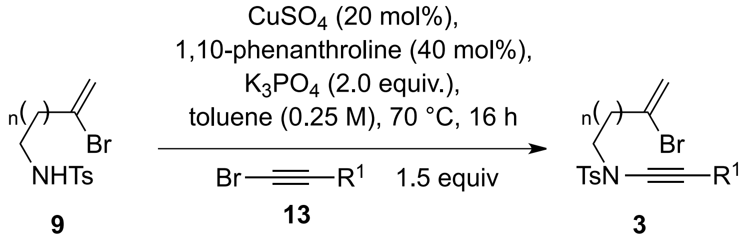

To a mixture of sulfonamide (1.0 equiv.), K_3PO_4 (2.0 equiv.), $CuSO_4.5H_2O$ (0.2 equiv.) and 1,10-phenanthroline (0.4 equiv.) was added a solution of bromoalkyne (1.5 equiv.) in toluene (4 mL $mmol^{-1}$). The mixture was heated to 70 °C for 16 h before being cooled to rt and concentrated. Purification by flash chromatography (EtOAc / petroleum ether) afforded the bromoenynamide.

General Procedure F: *Stille cascade cyclisation to amidodienes*

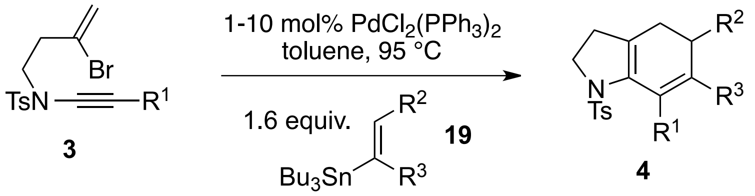

To a reaction vessel containing $PdCl_2(PPh_3)_2$ (1 or 10 mol%) under Ar was added a degassed (Ar bubbling, 15 min) solution of the bromoenynamide (1.0 equiv.) and stannane coupling partner (1.6 equiv) in toluene (16.7 mL $mmol^{-1}$). The reaction mixture was then heated to 95 °C under Ar until the reaction was judged complete by TLC (4-24 h). The reaction was then cooled to rt, and concentrated. Purification *via* flash chromatography (EtOAc / petroleum ether eluent) afforded the bicyclic dienamide product.

General Procedure G: *Suzuki cascade cyclisation to amidodienes*

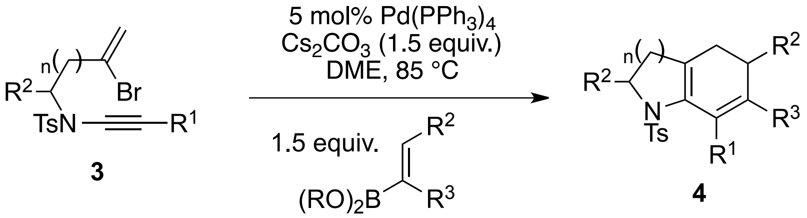

To a reaction vessel containing $Pd(PPh_3)_4$ (5 mol%) and Cs_2CO_3 (1.5 equiv.) under Ar was added a degassed (Ar bubbling, 15 min) solution of the bromoenynamide (1.0 equiv.) and vinylboronate (1.5 equiv.) in DME (16.7 mL.mmol^{-1}). The reaction mixture heated to reflux under Ar until the reaction was judged complete by TLC. The reaction was then cooled to rt and concentrated; purification *via* flash chromatography afforded the bicyclic dienamide product.

General Procedure H: *Reductive cyclization of bromoenynamides to pyrrolidines and piperidines* (*n = 1, 2)*

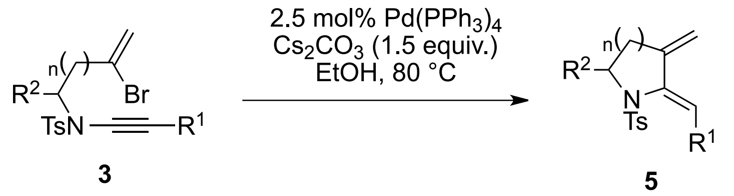

To a degassed (Ar bubbling, 15 min) solution of bromoenynamide (1.0 equiv.) in EtOH (0.045 M) was added $Pd(PPh_3)_4$ (2.5 mol%) and Cs_2CO_3 (1.5 equiv.). The reaction mixture was heated to 80 °C until judged complete by TLC, then it was cooled to rt and filtered through a Celite® plug (EtOAc eluent). The filtrate was concentrated, and the residue purified *via* column chromatography to afford the corresponding exocyclic diene product.

General Procedure I: *Reductive cyclization of bromoenynamides to piperidines (n = 2) and azepanes (n = 3)*

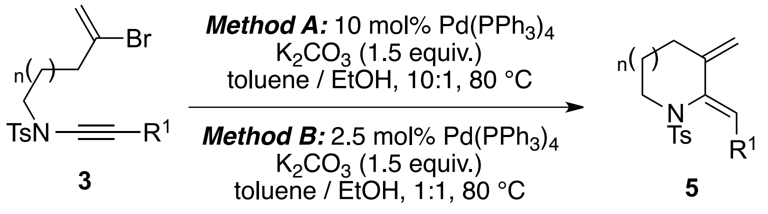

Method A: To a degassed (Ar bubbling, 15 min) solution of bromoenynamide (1.0 equiv.) in toluene / EtOH (10:1, 0.045 M) was added $Pd(PPh_3)_4$ (10 mol%) and K_2CO_3 (1.5 equiv.). The reaction mixture was heated to 80 °C until judged complete by TLC (3 x TLC runs on the same plate in 20:1 petroleum ether / EtOAc, to give separation of SM and product). The reaction was then cooled to rt and filtered through a Celite® plug (EtOAc eluent). The filtrate was concentrated, and the residue purified *via* flash chromatography to afford the corresponding exocyclic diene.

Method B: To a degassed (Ar bubbling, 15 min) solution of the appropriate bromoenynamide (1.0 equiv.) in toluene / EtOH (1:1, 0.045 M) was added $Pd(PPh_3)_4$ (2.5 mol%) and K_2CO_3 (1.5 equiv.). The reaction

mixture was heated to 80 °C until the reaction was judged complete by TLC (3 x TLC runs on the same plate in 20:1 petroleum ether / EtOAc to give separation). The reaction was tehn cooled to rt and filtered through a Celite® plug (EtOAc eluent). The filtrate was concentrated, and the residue purified *via* flash chromatography to afford the corresponding exocyclic diene.

General Procedure J: *Molander-Suzuki cascade cyclisation to amidodienes*

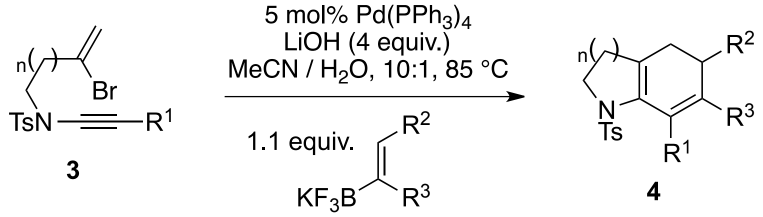

NB. No precautions of anhydrous, degassed solvent or inert atmosphere were employed.

To a mixture of ynamide (1 eq), potassium trifluoroborate (1.1 eq), LiOH (4 eq) and $Pd(PPh_3)_4$ (5 mol%) was added a solution of $MeCN:H_2O$ (10:1) (5 mL/mmol), the mixture immersed in an oil bath at 85 °C and stirred rapidly for up to 3 h. The mixture was cooled to ambient temperature, diluted with Et_2O (5 mL/mmol) and NH_4Cl(aq) added. The product was extracted with Et_2O (×2), dried ($MgSO_4$) and concentrated *in vacuo*, then purified *via* column chrotomagraphy (petroleum ether/Et_2O (9:1)).

3. Bromenynamide Synthesis

Preparation of alcohols 10a-c and ammonium salt 11:

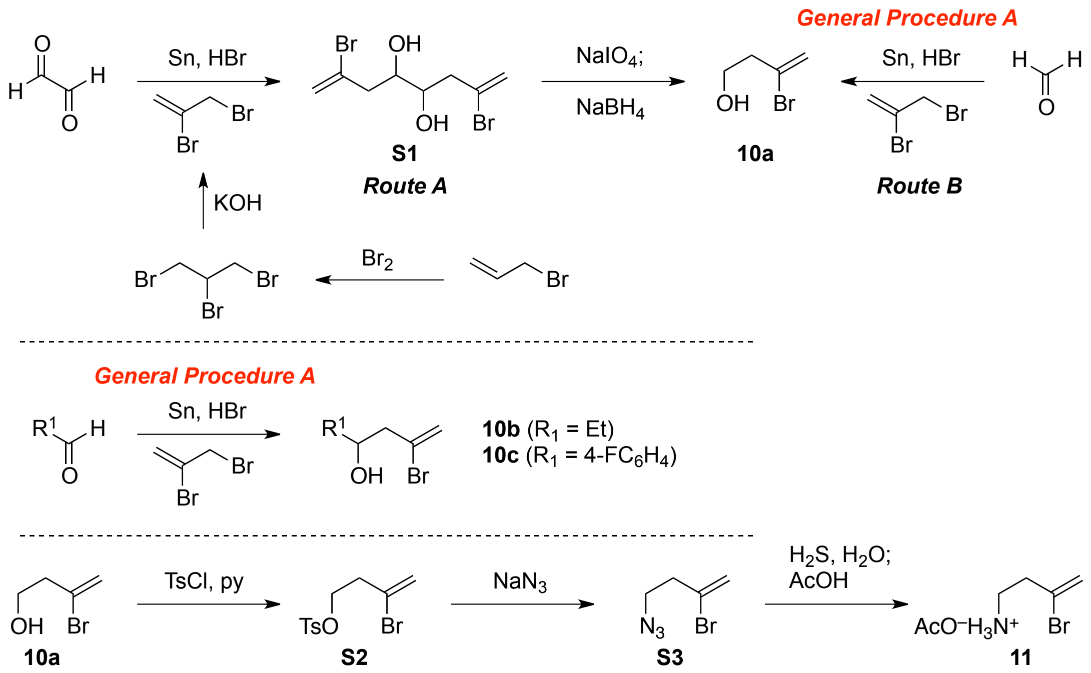

Large scale synthesis of 2,3-dibromoprop-1-ene:

1,2,3-Tribromopropane

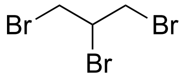

The procedure of Johnson and McEwen was used.[4] To a three-necked flask equipped with stirrer bar was added allyl bromide (181.5 g, 1.50 mol, 1.0 equiv.) and CCl_4 (250 mL). The flask was fitted with a dropping funnel, calcium chloride exit tube and a thermometer. To the dropping funnel was added bromine (255.0 g, 1.60 mol, 1.1 equiv.), pre-washed with an equal volume of conc. H_2SO_4. The bromine was added dropwise to the cooled (−5 °C) solution of allylbromide such that the temperature never exceeded 0 °C. After addition, the reaction mixture was allowed to warm to rt and stirred for a further 30 min. The dropping funnel and thermometer were removed and replaced with glass stoppers, and the drying tube replaced with distillation apparatus. The reaction mixture was heated to 120 °C at atmospheric pressure, and then slowly to 150 °C to distil off the remaining bromine, allyl bromide and carbon tetrachloride. The collecting flask was replaced, and the pressure reduced to 26 mbar to remove the remaining starting materials and solvent until the thermometer registered a sudden rise in temperature, at which point only the 1,2,3-tribromopropane remained in the distilling flask, as a pale yellow oil (419 g, 1.49 mmol, 99%, used without further purification); **R_f** 0.33 (petroleum ether / EtOAc (10:1)); **^{1}H NMR** (500 MHz, $CDCl_3$) δ_H 4.42 (1H, tt, J = 7.0, 4.0 Hz, H2), 3.99 (2H, dd, J = 7.0, 4.0 Hz, H1/H3), 3.90 (2H, dd, J = 7.0, 4.0 Hz, H1/H3); **^{13}C NMR** (125 MHz, $CDCl_3$) δ_C 48.7, 35.4. Data in accordance with literature values.[5]

2,3-Dibromoprop-1-ene

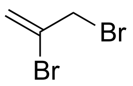

The procedure of Lespieau and Bourgeul was used for this reaction.[6] To a round bottomed flask, connected to efficient distillation equipment, was added 1,2,3-tribromopropane (419 g, 1.49 mol, 1.0 equiv.), water (22 mL) and NaOH pellets (104 g, 2.61 mol, 1.75 equiv.). An exotherm was observed, then the mixture was heated further using a heat gun, with stirring, until vigorous ebullition occurs, whereupon spontaneous distillation of the product took place; the heat source was immediately removed. When the reaction subsided, the flask was heated as before and the mass became solid as the volatile components were removed. Once all volatile components were removed, the distillate was isolated and washed with water (150 mL). The lower (organic) layer was then distilled *in vacuo* to yield 2,3-dibromopropene as a colorless oil (203 g, 1.19 mmol, 80%); **bp** 68 °C at 75 mbar; $\mathbf{R_f}$ 0.54 (petroleum ether / EtOAc (10:1)); **IR** (thin film) ν_{max} / cm^{-1} 2962, 1734, 1640, 1621, 1418, 1384, 1311, 1215, 1191, 1101, 938; ^{1}**H NMR** (400 MHz, $CDCl_3$) δ_H 6.03 (1H, dt, J = 2.0, 1.0 Hz, H1), 6.53 (1H, d, J = 2.0, H1'), 4.19 (2H, d, J = 1.0 Hz, H3); ^{13}**C NMR** (101 MHz, $CDCl_3$) 127.6, 121.2, 36.8; **HRMS** (CI) calc. for $C_3H_4Br_2$ 197.8680, found 197.8684.

3-Bromobut-3-en-1-ol, 10a

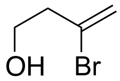

Route A: via 2,7-Dibromoocta-1,7-diene-4,5-diol S1. The procedure of Otera *et al.* was used for this reaction.[7] To a mixture of tin powder (29.7 g, 0.25 mol, 2.3 equiv.) and 2,3-dibromopropene (26.0 mL, 0.25 mol, 2.3 equiv.) in water / Et_2O (480 mL, 1:1) was added conc. aq. HBr (several drops) and aqeuous glyoxal (12.6 mL, 0.11 mol, 40% wt, 1.0 equiv.). The resulting mixture was stirred overnight at rt before being diluted with water and extracted with Et_2O. The combined organic layers were dried ($MgSO_4$) and the solvent removed *in vacuo* to afford diol **S1** as a coarse white powder (34.0 g, 0.25 mol, quant., unassigned mixture of diastereoisomers) which was used without further purification, but could be purified by flash chromatography (petroleum ether / ethyl acetate, 3:1) for the purposes of characterization; **mp** 85-86 °C; $\mathbf{R_f}$ 0.12 (petroleum ether / ethyl acetate, 3:1); **IR** (thin film) ν_{max} / cm^{-1} 3335, 2914, 1632, 1409, 1199, 1106, 898; ^{1}**H NMR** (400 MHz, $CDCl_3$) δ_H 5.76 (2H, app s, H1/H8), 5.59 (2H, t, J = 2.0 Hz, H1'/H8'), 4.05 (1H, m, H4/H5), 3.91 (1H, m, H4/H5), 2.77 (2H, m, H3/H6), 2.66 (2H, m, H3/H6); ^{13}**C NMR** (101 MHz, $CDCl_3$) δ_C 130.4, 130.3, 120.8, 120.6, 71.5, 70.9, 46.4, 44.4; **HRMS** (ES^-) calc. for $C_8H_{11}Br_2O_2$ $[M-H]^-$ 298.9144; found 298.9103.

To a solution of **S1** (7.60 g, 25.0 mmol, 1.0 equiv.) in MeOH / pH 7 buffer (108 mL, 5:1) was added $NaIO_4$ (8.13 g, 38.0 mmol, 1.5 equiv.). The reaction mixture was stirred at rt for 2 h, then cooled to 0 °C; to this was added $NaBH_4$ (1.93 g, 51.0 mmol, 2.0 equiv.) portionwise. Once the addition was complete, the reaction was allowed to warm to rt and stirred for 20 min, before being quenched with water. The mixture was extracted with CH_2Cl_2 and the combined organic layers dried ($MgSO_4$) and concentrated. The crude product was

purified by distillation to afford **10a** as a colorless liquid (5.12 g, 16.5 mmol, 66%); **bp** 74 °C @ 20 mbar; **R$_f$** 0.21 (petroleum ether / Et_2O, 1:1); **^{1}H NMR** (400 MHz, $CDCl_3$) δ_H 5.71 (1H, app s, H4), 5.53 (1H, app s, H4), 3.81 (2H, app q, J = 6.0 Hz, H1), 2.67 (2H, t, J = 6.0 Hz, H2), 1.87 (1H, t, J = 6.0 Hz, O*H*); **^{13}C NMR** (101 MHz, $CDCl_3$) δ_C 130.6, 119.5, 60.2, 44.5. Data in accordance with literature values.[8] *Note: the alcohol decomposes during distillation if attempted at higher temperatures than above.*

Route B: Prepared by General Procedure A using dibromopropene (44.0 mL, 0.43 mol) formaldehyde (19.4 mL, 0.33 mol, 37% in H_2O) to give **10a** as a pale yellow oil (48.8 g, 0.32 mol, 97%). Data as above.

5-Bromohex-5-en-3-ol, 10b

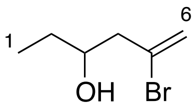

Prepared by General Procedure A using propionaldehyde (0.25 mL, 3.44 mmol) to afford **10b** as a colorless oil (572 mg, 3.16 mmol, 92%); **R$_f$** 0.23 (petroleum ether / EtOAc, 10:1); **^{1}H NMR** (500 MHz, $CDCl_3$) δ_H 5.70 (1H, app s, H6), 5.55 (1H, app s, H6), 3.88-3.84 (1H, m, H3), 2.58 (1H, dd, J = 14.5, 4.0 Hz, H4), 2.51 (1H, dd, J = 14.5, 8.5 Hz, H4), 1.75 (1H, br s, O*H*), 1.57-1.49 (2H, m, H2), 0.99 (3H, t, J = 7.5 Hz, H1); **^{13}C NMR** (125 MHz, $CDCl_3$) δ_C 130.9, 119.6, 70.4, 49.0, 29.3, 9.9. Data in accordance with literature values.[9]

3-Bromo-1-(4-fluorophenyl)but-3-en-1-ol, 10c

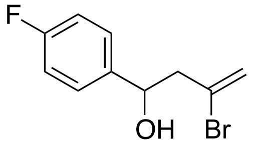

Prepared by General Procedure A using 4-fluorobenzaldehyde (5.20 mL, 48.2 mmol) to afford **10c** as a pale pink oil (7.63 g, 42.4 mmol, 88%); **R$_f$** 0.21 (petroleum ether / EtOAc, 10:1); **^{1}H NMR** (200 MHz, $CDCl_3$) δ_H 7.38-7.33 (2H, m, Ar*H*), 7.09-7.02 (2H, m, Ar*H*), 5.67 (1H, app s, H4), 5.55 (1H, d, J = 1.5 Hz, H4), 5.04 (1H, dd, J = 8.5, 4.5 Hz, H1), 2.84 (1H, ddd, J = 14.5, 8.5, 1.0 Hz, H2), 2.72 (1H, ddd, J = 14.5, 4.5, 1.0 Hz, H2); **^{13}C NMR** (101 MHz, $CDCl_3$) δ_C 162.3 (d, $J_{C\text{-}F}$ = 245.8 Hz), 138.6, 129.8, 127.5 (d, $J_{C\text{-}F}$ = 8.1 Hz), 120.2, 115.3 (d, $J_{C\text{-}F}$ = 21.4 Hz), 71.0, 51.2. Data in accordance with literature values.[10]

3-Bromobut-3-en-1-yl 4-methylbenzenesulfonate, S2

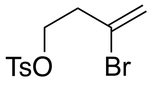

To an ice-cooled solution of alcohol **10a** (5.11 g, 33.8 mmol, 1.0 equiv.) in CH_2Cl_2 (2.6 mL) was added pyridine (5.5 mL, 67.7 mmol, 2.0 equiv.) and *p*-toluenesulfonyl chloride (9.68 g, 50.8 mmol, 1.5 equiv.). After addition, the reaction was warmed to rt and stirred for 3 h before being diluted with CH_2Cl_2. The organic layer was separated and washed with HCl (1M, aq.). The aqueous layer was extracted with CH_2Cl_2,

and the combined organics washed with $NaHCO_3$ (sat., aq.), dried ($MgSO_4$) and concentrated. Purification *via* flash chromatography (petroleum ether / Et_2O, 8:1) afforded tosylate **S2** as a colorless oil (10.3 g, 33.8 mmol, quant.); **R*f*** 0.21 (petroleum ether / Et_2O, 8:1); **^{1}H NMR** (400 MHz, $CDCl_3$) δ_H 7.79 (1H, d, *J* = 8.0 Hz, Ar*H*), 7.35 (2H, d, *J* = 8.0 Hz, Ar*H*), 5.65 (1H, app s, H4), 5.48 (1H, d, *J* = 2.0 Hz, H4), 4.19 (2H, t, *J* = 6.2 Hz, H1), 2.74 (2H, t, *J* = 6.2 Hz, H2), 2.45 (3H, s, TsC*H$_3$*); **^{13}C NMR** (101 MHz, $CDCl_3$) δ_C 145.0, 132.8, 129.9, 128.0, 127.6, 120.2, 67.2, 40.7, 21.7. Data in accordance with literature values.[11]

4-Azido-2-bromobut-1-ene, S3

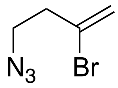

The procedure of Pillai was used for this reaction.[11] To sodium azide (1.46 g, 22.5 mmol, 2.0 equiv.) at rt was added a solution of tosylate **S2** (3.43 g, 11.2 mmol, 1.0 equiv.) in DMSO (12.7 mL). After stirring overnight, water was added and the mixture extracted with Et_2O. The combined organic extracts were dried ($MgSO_4$) and concentrated *in vacuo* to afford azide **S3** as a yellow oil (1.64 g, 9.33 mmol, 83%) which was used without further purification; **R*f*** 0.52 (petroleum ether / Et_2O, 8:1); **^{1}H NMR** (400 MHz, $CDCl_3$) δ_H 5.73 (1H, br s, H1), 5.55 (1H, br d, *J* = 1.0 Hz, H1), 3.50 (2H, t, *J* = 6.5 Hz, H4), 2.68 (2H, t, *J* = 6.5 Hz, H3); **^{13}C NMR** (101 MHz, $CDCl_3$) δ_C 129.7, 119.6, 49.0, 40.8. Data in accordance with literature values.[11]

3-Bromobut-3-en-1-aminium acetate, 11, and 3-bromobut-3-en-1-amine, S4

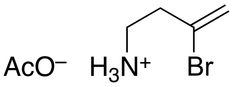

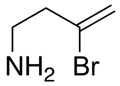

The procedure of Pillai was used for this reaction.[11] Hydrogen sulfide was bubbled through a solution of azide **S3** (1.94 g, 11.0 mmol) in pyridine / water (40 mL, 1:1). After 2 h, 2N acetic acid was added until the reaction was neutralized, and the mixture was then concentrated *in vacuo*, then azeotroped several times with EtOH. The resulting brown oil was dissolved in water, filtered, and concentrated *in vacuo*, co-evaporating several times with toluene, to give the amine salt **11** as a brown oil (1.79 g, 8.48 mmol, 77%); **^{1}H NMR** (400 MHz, D_2O) 5.70 (1H, app s, H4), 5.50 (1H, app s, H4), 4.67 (3H, br, *NH_3^+*), 3.07 (2H, t, *J* = 6.5 Hz, H1), 2.66 (2H, t, *J* = 6.5 Hz, H2), 1.78 (3H, s, OAc); **^{13}C NMR** (101 MHz, D_2O) δ_C 180.4, 127.9, 121.6, 38.7, 37.8, 23.0. Data in accordance with literature values.[11]

For subsequent reactions of the free amine (*vide infra*), **11** was partitioned between sat. *aq*. NaOH and CH_2Cl_2, and the organic layers combined, dried ($MgSO_4$) and concentrated *in vacuo* to afford the amine **S4**; **R*f*** 0.19 (petroleum ether / EtOAc, 1:1); **^{1}H NMR** (500 MHz, $CDCl_3$) δ_H 5.64 (1H, appt s, H4), 5.49 (1H, appt s, H4), 2.90 (2H, t, *J* = 6.1 Hz, H1), 2.53 (2H, t, *J* = 6.1 Hz, H2); **^{13}C NMR** (125 MHz, $CDCl_3$) δ_C 131.9, 118.6, 45.3, 39.8. Data in agreement with literature values.[12]

Preparation of amide derivatives 9a-h:

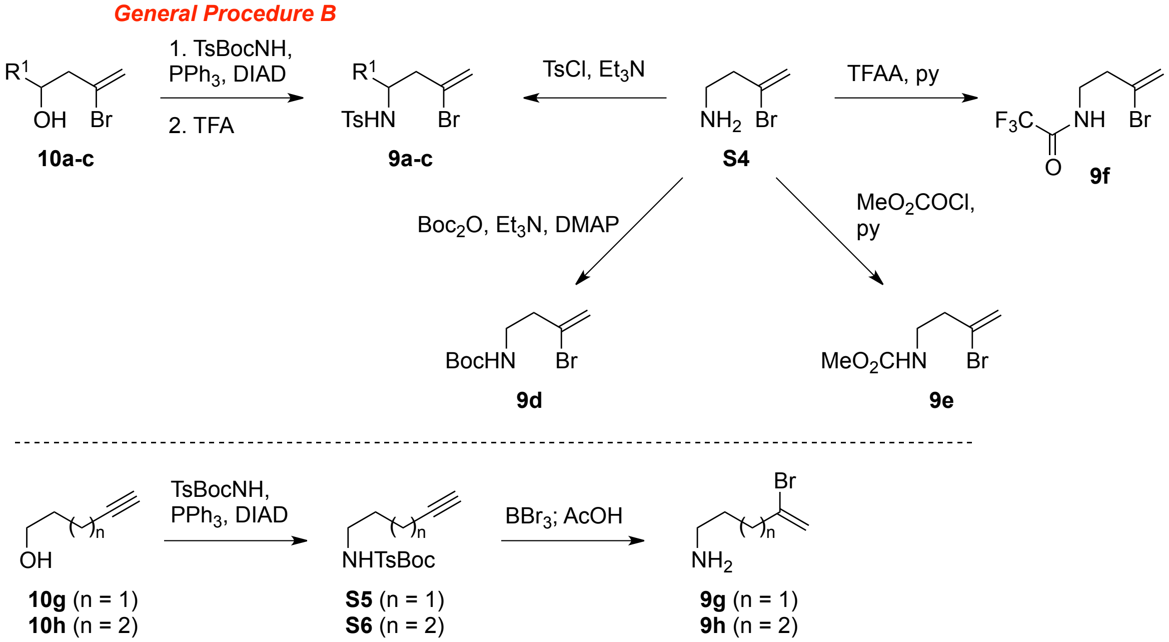

***N*-(3-Bromobut-3-en-1-yl)-4-methylbenzenesulfonamide, 9a**

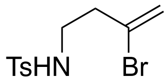

Method A: To a stirred solution of the free amine **S4** (derived from **11** as above, 200 mg, 1.33 mmol, 1.0 equiv.) and triethylamine (0.74 mL, 5.33 mmol, 4.0 equiv.) in CH_2Cl_2 (4 mL) at 0 °C, was added *p*-toluenesulfonyl chloride (253 mg, 1.33 mmol, 1.0 equiv.). Upon completion as judged by TLC, the reaction mixture was diluted with CH_2Cl_2 and washed with 1 M HCl. The aqueous layer was extracted with CH_2Cl_2 and the combined organic phases were washed with $NaHCO_3$ (sat., aq.), dried ($MgSO_4$) and concentrated to afford sulfonamide **9a** as a pale yellow oil (358 mg, 1.18 mmol, 89%) which was of a sufficient purity to be used without further purification.

Method B: Prepared by General Procedure B using 3-bromobut-3-en-1-ol **10a** (5.57 g, 36.9 mmol) to afford sulfonamide **9a** as a colorless oil, which solidified on storage to a cream colored solid (7.43 g, 24.0 mmol, 65%); **mp** 44-45 °C; $\mathbf{R}_f$ 0.13 (petroleum ether / EtOAc, 5:1); **IR** (thin film) ν_{max} / cm^{-1} 3261, 1629, 1598, 1423, 1317, 1163; ^{1}**H NMR** (400 MHz, $CDCl_3$) δ_H 7.76 (2H, d, *J* = 8.5 Hz, Ar*H*), 7.32 (2H, d, *J* = 8.5 Hz, Ar*H*), 5.60 (1H, app. s, H4), 5.48 (1H, d, *J* = 1.5 Hz, H4), 4.83 (1H, br t, *J* = 6.0 Hz, N*H*), 3.16 (2H, t, *J* = 6.5 Hz, H1), 2.57 (2H, t, *J* = 6.5 Hz, H2), 2.43 (3H, s, TsC*H*$_3$); ^{13}**C NMR** (101 MHz, $CDCl_3$) δ_C 143.6, 136.9, 129.8, 129.6, 127.1, 120.0, 41.3, 41.0, 21.5; **HRMS** (ES+) calc. for $C_{11}H_{15}{}^{81}BrNO_2S$ $[M+H]^+$ 305.9981; found 305.9965.

***N*-(5-Bromohex-5-en-3-yl)-4-methylbenzenesulfonamide, 9b**

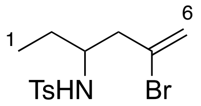

Prepared by General Procedure B using **10b** (572 mg, 3.01 mmol) to afford the sulfonamide **225b** as a colorless oil (425 mg, 1.28 mmol, 42%); **R*f*** 0.06 (petroleum ether / EtOAc, 10:1); **IR** (thin film) ν_{max} / cm^{-1} 3281, 1631, 1599, 1451, 1430, 1324, 1158, 1093, 1068, 893; **^{1}H NMR** (500 MHz, $CDCl_3$) δ_H 7.78 (2H, d, *J* = 8.0 Hz, Ar*H*), 7.31 (2H, d, *J* = 8.0 Hz, Ar*H*), 5.56 (1H, app s, H6), 5.40 (1H, app s, H6), 4.46 (1H, d, *J* = 8.0 Hz, N*H*), 3.47 (1H, app sextet, *J* = 6.5 Hz, H3), 2.52 (1H, dd, *J* = 14.5, 6.5 Hz, H4), 2.45 (1H, dd, *J* = 14.5, 7.0 Hz, H4), 2.44 (3H, s, TsC*H*$_3$), 1.64-1.61 (1H, m, H2), 1.42 (1H, app sept, *J* = 7.0 Hz, H2), 0.83 (3H, t, *J* = 7.5 Hz, H1); **^{13}C NMR** (125 MHz, $CDCl_3$) δ_C 143.4, 137.8, 129.7, 129.6, 127.3, 120.1, 53.6, 46.4, 26.9, 21.6, 9.5; **HRMS** (ES^+) calc. for $C_{13}H_{18}BrNNaO_2S$ $[M+Na]^+$ 354.0134; found 354.0122.

***N*-(3-Bromo-1-(4-fluorophenyl)but-3-en-1-yl)-4-methylbenzenesulfonamide, 9c**

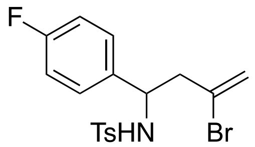

Prepared by General Procedure B using **10c** (2.00 g, 8.16 mmol) to afford sulfonamide **9c** as a pale yellow oil (1.99 g, 4.90 mmol, 60%); **R*f*** 0.29 (petroleum ether / EtOAc, 3:1); **IR** (thin film) ν_{max} / cm^{-1} 3373, 2911, 1629, 1509, 1223, 1157; **^{1}H NMR** (200 MHz, $CDCl_3$) δ_H 7.58 (2H, d, *J* = 8.5 Hz, Ts*H*), 7.18 (2H, d, *J* = 8.5 Hz, Ts*H*), 7.09-7.06 (2H, m, 4-FPh*H*), 6.88 (2H, t, *J* = 8.5 Hz, 4-FPh*H*), 5.51 (1H, dt, *J* = 2.0, 1.0 Hz, H4), 5.40 (1H, d, *J* = 2.0 Hz, H4), 5.06 (1H, d, *J* = 5.5 Hz, N*H*), 4.59 (1H, q, *J* = 6.5 Hz, H1), 2.82 (1H, ddd, *J* = 14.5, 8.0, 1.0 Hz, H2), 2.70 (1H, ddd, *J* = 14.5, 6.0, 1.0 Hz, H2), 2.40 (3H, s, TsC*H*$_3$); **^{13}C NMR** (101 MHz, $CDCl_3$) δ_C 162.2 (d, J_{C-F} = 246.6 Hz), 143.5, 136.8, 135.1 (d, J_{C-F} = 3.1 Hz), 129.4, 128.4, 128.4, 127.3, 120.9, 115.4 (d, J_{C-F} = 21.7 Hz), 55.6, 49.2, 21.5. **HRMS** (FI^+) calc. for $C_{10}H_{12}{}^{79}BrFN$ $[M+H]^+$ 244.0132; found 244.0121.

***tert*-Butyl (3-bromobut-3-en-1-yl)carbamate, 9d**

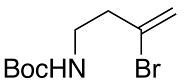

To a solution of free amine **S4** (derived from **11** as above, 200 mg, 1.33 mmol, 1.0 equiv.) in THF (5 mL) was added triethylamine (0.09 mL, 0.66 mmol, 0.5 equiv.) and di-*tert*-butyl dicarbonate (Boc_2O, 275 mg, 1.26 mmol, 0.95 equiv.). The reaction mixture was stirred at rt for 24 h before the addition of DMAP (16 mg, 0.13 mmol, 0.1 equiv.). After stirring at rt for a further 48 h the reaction was heated to reflux for 5 h. Upon completion as judged by TLC, the reaction was cooled to rt and quenched with 1 M HCl. The mixture was extracted with CH_2Cl_2, and the combined organic layers were dried ($MgSO_4$) and concentrated, to afford **9d** as a brown oil (210 mg, 0.84 mmol, 63%) which was used without further purification, but could be purified

by flash chromatography for the purposes of characterization: **R$_f$** 0.55 (petroleum ether / EtOAc, 10:1); **IR** (thin film) ν_{max} / cm^{-1} 3351, 2977, 1698, 1630, 1519, 1366, 1251; **^{1}H NMR** (400 MHz, $CDCl_3$) δ_H 5.64 (1H, app s, H4), 5.49 (1H, app s, H4), 4.68 (1H, br, N*H*), 3.33 (2H, d, *J* = 6.0 Hz, H1), 2.61 (2H, t, *J* = 6.0 Hz, H2), 1.43 (9H, s, *t*-Bu); **^{13}C NMR** (101 MHz, $CDCl_3$) δ_C 155.8, 131.0, 119.0, 79.4, 41.9, 38.6, 28.4; **HRMS** (ES+) calc. for $C_9H_{16}BrNNaO_2$ [M+Na]$^+$ 272.0257; found 272.0257.

Methyl (3-bromobut-3-en-1-yl)carbamate, 9e

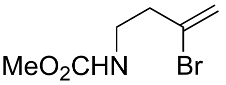

To a solution of free amine **S4** (50 mg, 0.33 mmol, 1.0 equiv.) in CH_2Cl_2 (1 mL) was added pyridine (54 µL, 0.66 mmol, 2.0 equiv.) then methyl chloroformate (31 µL, 0.40 mmol, 1.2 equiv.). The resulting reaction mixture was stirred at rt overnight, and then was diluted with CH_2Cl_2 and washed with 1 M HCl. The aqueous layer was extracted with CH_2Cl_2 and the combined organic phases washed with $NaHCO_3$ (sat. aq.), dried ($MgSO_4$) and concentrated to afford **9e** as a colorless oil (56 mg, 0.27 mmol, 81%), which was used without further purification, but could be purified by flash chromatography for the purposes of characterization: **R$_f$** 0.57 (petroleum ether / EtOAc, 2:1); **^{1}H NMR** (400 MHz, $CDCl_3$) δ_H 5.65 (1H, app s, H4), 5.50 (1H, app s, H4), 4.87 (1H, br s, N*H*), 3.66 (3H, s, CO_2CH_3), 3.39 (2H, q, *J* = 6.0 Hz, H1), 2.62 (2H, t, *J* 6.0 Hz, H2); **^{13}C NMR** (101 MHz, $CDCl_3$) δ_C 156.9, 130.8, 119.2, 52.1, 41.5, 38.9; **HRMS** (FI$^+$) calc. for $C_6H_{10}BrNO_2$ [M]$^+$ 206.9895, found 206.9889.

***N*-(3-Bromobut-3-en-1-yl)-2,2,2-trifluoroacetamide, 9f**

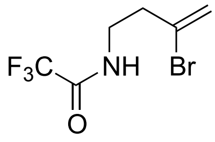

To a solution of free amine **S4** (145 mg, 0.97 mmol, 1.0 equiv.) in CH_2Cl_2 (2 mL) at 0 °C was added pyridine (0.16 mL, 1.94 mmol, 2.0 equiv.) and then trifluoroacetic anhydride (0.16 mL, 1.16 mmol, 1.2 equiv.). The resulting mixture was warmed to rt and stirred overnight before being diluted with CH_2Cl_2 and washed with 1 M HCl. The aqueous layer was extracted with CH_2Cl_2 and the combined organics washed with $NaHCO_3$ (sat. aq.), dried ($MgSO_4$) and concentrated to afford **9f** as a brown oil (140 mg, 0.57 mmol, 59%), which was of sufficient purity to be used without further purification, but could be purified by flash chromatography for the purposes of characterization; **R$_f$** 0.69 (petroleum ether / EtOAc, 2:1); **IR** (thin film) ν_{max} / cm^{-1} 3313, 3104, 2951, 1708, 1632, 1560, 1432, 1185; **^{1}H NMR** (400 MHz, $CDCl_3$) δ_H 6.60 (1H, br s, N*H*), 5.69 (1H, app s, H4), 5.56 (1H, d, *J* = 2.0 Hz, H4), 3.60 (2H, q, *J* = 6.5 Hz, H1), 2.72 (2H, t, *J* = 6.5 Hz, H2); **^{13}C NMR** (101 MHz, $CDCl_3$) δ_C 157.7, 129.6, 120.2, 114.3 (q, *J* = 287 Hz), 40.2, 37.9; **HRMS** (ES$^-$) calc. for $C_6H_6BrF_3NO$ [M-H]$^-$ 243.9590; found 243.9589.

***tert*-Butyl pent-4-yn-1-yl(tosyl)carbamate (S5)**

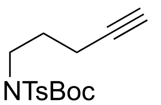

Di-*iso*propylazodicarboxylate (0.72 g, 3.57 mmol, 1.5 equiv.) was added dropwise to a stirred solution of PPh_3 (1.25 g, 4.77 mmol, 2.0 equiv.), TsNHBoc (0.84 g, 3.9 mmol, 1.3 equiv.) and 4-pentyn-1-ol (0.22 mL, 2.38 mmol, 1.0 equiv.) in THF (7 mL) at 0 °C. The reaction was warmed to rt, and stirred overnight, then concentrated. The residue was purified by flash chromatography (petroleum ether / EtOAc, 10:1) to give *N*-Boc-*N*-Ts amide **S5** as a white solid (768 mg, 2.28 mmol, 95%); **mp** 97.4-100.6 °C; $\mathbf{R}_f$ 0.27 (petroleum ether / EtOAc, 5:1); **IR** (thin film) ν_{max} / cm^{-1} 3278, 3007, 2984, 2960, 2929, 1726, 1597, 1443, 1369, 1346, 1320, 1257, 1118; **^{1}H NMR** (400 MHz, $CDCl_3$) δ_H 7.78 (2H, d, *J* = 8.5 Hz, Ts*H*), 7.30 (2H, d, *J* = 8.0 Hz, Ts*H*), 3.92 (2H, app dd, *J* = 8.0, 7.0 Hz, H1), 2.44 (3H, s TsCH_3), 2.28 (2H, td, *J* = 7.0, 2.5 Hz, H3), 1.99 (2H, app quin, *J* = 7.0 Hz, H2), 1.98 (1H, t, *J* = 2.5 Hz, H5), 1.34 (9H, s, *t*-Bu); **^{13}C NMR** (101 MHz, $CDCl_3$) δ_c 144.3, 137.5, 129.4, 128.0, 84.4, 83.2, 69.1, 46.4, 29.0, 28.0, 21.8, 16.2; **HRMS** (ES^+) calc. for $C_{17}H_{23}NNaO_4S^+$ $[M+Na]^+$ 360.1240; found 360.1237.

***N*-(4-Bromopent-4-en-1-yl)-4-methylbenzenesulfonamide, 9g**

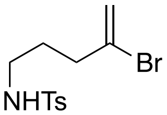

A modified procedure of Moran and Morken was used.[13] To an oven-dried flask equipped with a stirrer bar was added BBr_3 (9.05 mL, 9.05 mmol, 1 M in CH_2Cl_2, 0.5 equiv.). The solution was cooled to −78 °C, before the dropwise addition of a solution of alkyne **S5** (6.11 g, 18.1 mmol, 1.0 equiv.) in CH_2Cl_2 (30 mL). The reaction mixture was allowed to warm to rt over 4.5 h, then glacial AcOH (19 mL) was added. After stirring for a further 1 h, the reaction was quenched by the addition of water and then extracted with CH_2Cl_2 (x 3). The combined organic layers were dried ($MgSO_4$), filtered and concentrated. Purification *via* flash chromatography (petroleum ether / EtOAc, 10:1→3:1) afforded **9g** as a light brown oil (2.57 g, 7.96 mmol, 44%); $\mathbf{R}_f$ 0.13 (petroleum ether / EtOAc + 1% NEt_3, 10:1); **^{1}H NMR** (200 MHz, $CDCl_3$) δ_H 7.75 (2H, d, *J* = 8.5 Hz, Ts*H*), 7.30 (2H, d, *J* = 8.5 Hz, Ts*H*), 5.54 (1H, m, H5), 5.36 (1H, d, *J* = 2.0 Hz, H5), 5.03 (1H, br s, N*H*), 2.94 (2H, q, *J* = 6.5 Hz, H1), 2.42 (5H, m, TsCH_3 and H3), 1.72 (2H, qu, *J* = 7.0 Hz, H2). Data in accordance with literature values.[14]

***tert*-Butyl *N*-hex-5-ynyl-*N*-(*p*-tolylsulfonyl)carbamate (S6)**

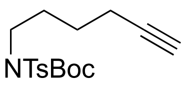

Di-*iso*propylazodicarboxylate (3.09 g, 15.3 mmol, 1.5 equiv.) was added dropwise to a stirred solution of PPh_3 (5.34 g, 20.4 mmol, 2.0 equiv.), TsNHBoc (3.59 g, 13.2 mmol, 1.3 equiv.) and 5-hexyn-1-ol (1.0 g, 10.2 mmol, 1.0 equiv.) in THF (7 mL) at 0 °C. The reaction was warmed to rt and stirred overnight, then

concentrated. The residue was purified by flash chromatography (petroleum ether→petroleum ether / EtOAc, 4:1) to give *N*-Boc-*N*-Ts amide **S6** as a white solid (3.31 g, 9.43 mmol, 93%); **mp** 65.3-67.9 °C; $\mathbf{R_f}$ 0.41 (petroleum ether / EtOAc, 5:1); **IR** (thin film) ν_{max} / cm^{-1} 3286, 2979, 1724, 1598, 1456, 1352, 1289, 1256, 1184, 1087; **^{1}H NMR** (400 MHz, $CDCl_3$) δ_H 7.78 (2H, d, *J* = 8.0 Hz, Ts*H*), 7.30 (2H, d, *J* = 8.0 Hz, Ts*H*), 3.85 (2H, t, *J* = 7.5 Hz, H1), 2.44 (3H, s, TsCH_3), 2.26 (2H, td, *J* = 7.0, 2.5 Hz, H4), 1.97 (1H, t, *J* = 2.5 Hz, H6), 1.88 (2H, qu, *J* = 7.5 Hz, H2), 1.60 (2H, app quin, *J* = 7.5 Hz, H3), 1.34 (9H, s, *t*-Bu); **^{13}C NMR** (101 MHz, $CDCl_3$) δ_C 151.0, 144.1, 137.4, 129.2, 127.8, 84.2, 84.0, 68.7, 46.6, 29.3, 27.9, 25.6, 21.6, 18.1; **HRMS** (ES+) calc. for $C_{18}H_{25}NNaO_4S$ $[M+Na]^+$ 374.1397; found 374.1391.

N-(5-Bromohex-5-en-1-yl)-4-methylbenzenesulfonamide, 9h

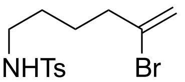

A modified procedure of Moran and Morken was used.[13] To an oven-dried flask equipped with a stirrer bar was added BBr_3 (0.14 mL, 0.14 mmol, 1 M in CH_2Cl_2, 0.5 equiv.). The solution was cooled to −78 °C, before the dropwise addition of a solution of alkyne **S6** (100 mg, 0.28 mmol, 1.0 equiv) in CH_2Cl_2 (0.5 mL). The reaction mixture was allowed to warm to rt over 4.5 h, then glacial AcOH (0.3 mL) was added. After stirring for a further 1 h, the reaction was quenched by the addition of water and then extracted with CH_2Cl_2 (x 3). The combined organic layers were dried ($MgSO_4$), filtered and concentrated. Purification *via* flash chromatography (petroleum ether / EtOAc, 10:1→3:1) afforded **9h** as a colorless oil (37 mg, 0.086 mmol, 30%); $\mathbf{R}_\mathit{f}$ 0.10 (petroleum ether / EtOAc, 10:1); **IR** (thin film) ν_{max} / cm^{-1} 3279, 2941, 2867, 1630, 1599, 1433, 1156, 1093, 887; **^{1}H NMR** (500 MHz, $CDCl_3$) δ_H 7.75 (2H, d, *J* = 8.0 Hz, Ts*H*), 7.32 (2H, d, *J* = 8.0 Hz, Ts*H*), 5.52 (1H, app s, H6), 5.37 (1H, app s, H6), 4.60 (1H, t, *J* = 6.0 Hz, N*H*), 2.96 (2H, app q, *J* = 6.5 Hz, H1), 2.44 (3H, s, TsCH_3), 2.37 (2H, t, *J* = 7.0 Hz, H4), 1.55-1.46 (4H, m, H2 and H3); **^{13}C NMR** (125 MHz, $CDCl_3$) δ_C 143.5, 137.0, 133.8, 129.8, 127.1, 117.0, 42.9, 40.7, 28.3, 24.8, 21.6; **HRMS** (ES+) calc. for $C_{13}H_{18}BrNNaO_2S$ $[M+Na]^+$ 354.0134, found 354.0127. (This compound was made in higher yield by Mitsunobu reaction / deprotection on 5-bromohex-5-en-1-ol; Mitsunobu yield 59%; TFA deprotection 88%), according to General Procedure B.

Preparation and use of *N*-(2-bromoallyl)-4-methyl-*N*-(2-(4-methylphenylsulfonamido)ethyl)benzene sulfonamide, 9i

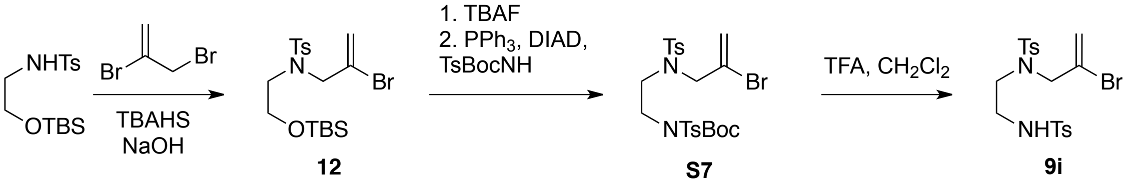

To a rapidly stirring solution of *N*-(2-((*tert*-butyldimethylsilyl)oxy)ethyl)-4-methylbenzenesulfonamide[15] (2.63 g, 7.98 mmol) in PhMe (80 mL) was added tetrabutylammonium hydrogensulfate (271 mg, 0.798 mmol), followed by a solution of NaOH (80 mL of a 25% w/v aq. solution). The mixture was stirred

rapidly for 45 min, then the layers were separated and the organic phase was washed with NH_4Cl (30 mL, sat., aq.). The organic phase was dried ($MgSO_4$) and concentrated to afford the crude product as a pale yellow semi-solid, which was purified by rapid flash chromatography (7%→12% EtOAc / petroleum ether) to afford intermediate **12** as a colorless oil (3.38 g, 7.54 mmol, 94%) that was used immediately in the subsequent steps. *Note: **12** decomposes on standing when neat.* To a solution of *N*-(2-bromoallyl)-*N*-(2-((*tert*-butyldimethylsilyl)oxy)ethyl)-4-methylbenzenesulfonamide **12** (3.00 g, 6.69 mmol) in THF (75 mL) at 0 °C was added TBAF (6.8 mL of a 1 M solution in THF, 6.8 mmol), and the reaction was stirred for 1 h. The reaction was then quenched with NH_4Cl (30 mL, sat., aq.) and extracted with Et_2O (2 × 30 mL). The combined organic fractions were dried (Na_2SO_4), filtered and concentrated to afford the crude alcohol *N*-(2-bromoallyl)-*N*-(2-hydroxyethyl)-4-methylbenzenesulfonamide as a colorless oil (2.07 g, ~93%) which was used immediately in the subsequent reaction. To a solution of PPh_3 (1.70 g, 6.48 mmol) in THF (30 mL) at 0 °C was added DIAD (1.12 mL, 5.69 mmol) steadily, and the mixture stirred for 20 min. A solution of crude deprotected alcohol (1.80 g, 5.39 mmol) in THF (10 mL) was added dropwise, then the mixture was stirred for 10 min. BocNHTs (1.47 g, 5.42 mmol) was added in one portion and the mixture warmed to rt over 16 h. The mixture was concentrated, then redissolved in a minimal volume of $CHCl_3$ and then filtered rapidly through a pad of silica, eluting with 1.6% v/v acetone / $CHCl_3$, then concentrated *in vacuo* to afford the *tert*-butyl (2-((*N*-(2-bromoallyl)-4-methylphenyl)sulfonamido)ethyl)(tosyl)carbamate **S7** (2.45 g) as an unstable colorless oil; $\mathbf{R}_f$ 0.31 (1.6% v/v acetone / $CHCl_3$); **^{1}H NMR** (400 MHz, $CDCl_3$) δ_H 7.79 (2H, d, *J* = 8.5 Hz, Ts*H*), 7.78 (2H, d, *J* = 8.5 Hz, Ts*H*), 7.33 (2H, d, *J* = 8.5 Hz, Ts*H*), 7.32 (2H, d, *J* = 8.5 Hz, Ts*H*), 5.93-5.89 (1H, m, C=C*H*H), 5.65 (1H, br d, *J* = 2.0 Hz, C=CH*H*), 4.16 (2H, s, TsNCH_2C(Br)=CH_2), 4.01-3.95 (2H, m, TsBocNCH_2), 3.49-3.44 (2H, m, TsBocNCH_2CH_2), 2.45 (3H, s, Ts*Me*), 2.44 (3H, s, Ts*Me*), 1.39 (9H, s, OC(CH_3)$_3$). This intermediate **S7** (2.45 g) was immediately redissolved in CH_2Cl_2 (37 mL) and treated with TFA (37 mL). After 1 h, the organic fraction was washed successively with H_2O (50 mL) and $NaHCO_3$(aq) (30 mL), then dried (Na_2SO_4), filtered and concentrated *in vacuo* to afford the crude sulfonamide product **9i** as a colorless oil (1.97 g, ~75% over three steps). *NOTE: Throughout this procedure, concentration in vacuo must be carried out with bath temperature <20 °C to minimize decomposition.* Sulfonamide **9i** undergoes ready decomposition upon concentration and in neutral solution, so must be stored below –20 °C or used immediately.

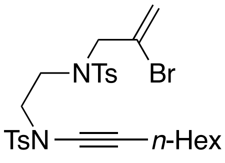

3l

Formation of ynamide **3l**: To a mixture of crude tosylamide **9i** (1.00 g, 2.05 mmol), K_3PO_4 (870 mg, 4.10 mmol), 1,10-phenanthroline (296 mg, 1.64 mmol), $CuSO_4$•$5H_2O$ (205 mg, 0.821 mmol) and 1-bromo-1-octyne (545 mg, 2.88 mmol) was added anhydrous PhMe (10 mL) and the mixture heated to 70 °C for 16 h. The mixture was cooled to rt and passed through cotton wool, eluting with Et_2O, then organic concentrated. Purification by flash chromatography (petroleum ether / EtOAc (4:1)) afforded the ynamide **3l**

as a colorless oil (663 mg, 54%); **R*f*** 0.38 (petroleum ether / EtOAc (7:3)); **^{1}H NMR** (400 MHz, $CDCl_3$) δ_H 7.76 (2H, d, *J* = 8.0 Hz, Ts*H*), 7.72 (2H, d, *J* = 8.0 Hz, Ts*H*), 7.35 (2H, d, *J* = 8.0 Hz, Ts*H*), 7.32 (2H, d, *J* = 8.0 Hz, Ts*H*), 5.84 (1H, d, *J* = 2.0 Hz, H5), 5.62 (1H, d, *J* = 2.0 Hz, H5′), 4.07 (2H, s, H3), 3.49-3.46 (2H, m, H1), 3.41-3.37 (2H, m, H2), 2.46 (3H, s, TsCH_3), 2.44 (3H, s, TsCH_3), 2.24 (2H, t, *J* = 7.0 Hz, H8), 1.52-1.43 (2H, m, H9), 1.36-1.23 (6H, m, H10, H11, H12), 0.89 (3H, t, *J* = 7.0 Hz, H13); **^{13}C NMR** (101 MHz, $CDCl_3$) δ_C 144.7, 143.9, 136.1, 134.1, 129.8, 129.8, 127.8, 127.6, 127.4, 120.5, 72.8, 70.5, 56.6, 50.6, 45.9, 31.3, 28.8, 28.5, 22.6, 21.7, 21.6, 18.4, 14.1; Data as reported previously.[16]

Preparation and use of *N*-(2-bromoallyl)-4-methyl-*N*-(3-((4-methylphenyl)sulfonamido)propyl) benzenesulfonamide, 9j

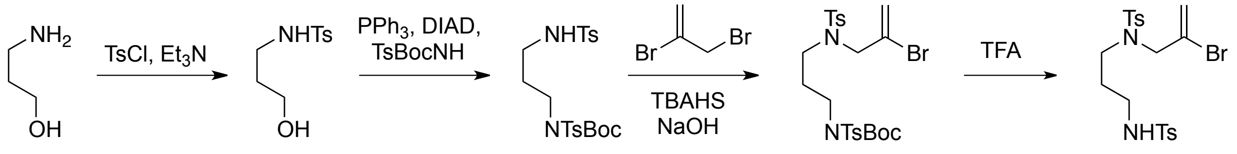

9j

To a solution of 1-aminopropanol (5.00 g, 66.0 mmol) in $CHCl_3$ (10 mL) at 0 °C was added Et_3N (25.6 mL, 133 mmol), followed by a solution of tosyl chloride (13.8 g, 72.6 mmol) in $CHCl_3$ (15 mL) over a period of 10 min. The mixture was warmed slowly to rt, then quenched with $NaHCO_3$ (25 mL of a 5% w/v aq. solution), and EtOAc (50 mL) was added. The layers were separated and the organic phase was washed with citric acid (2 × 20 mL, 5% w/v aq. solution) then brine (20 mL). The organic phase was dried ($MgSO_4$) and concentrated to afford the crude tosylated product as a pale yellow semi-solid (13.4 g, ~88%).

To a solution of PPh_3 (2.06 g, 7.85 mmol) in THF (30 mL) at 0 °C was added DIAD (1.35 mL, 6.87 mmol) steadily, and the mixture stirred for 15 min. A solution of crude tosylated product (1.50 g, 6.54 mmol) in THF (30 mL) was added dropwise, then the mixture stirred for 10 min. BocNHTs (1.95 g, 7.20 mmol) was added in one portion, and the mixture warmed to rt over 16 h. The mixture was concentrated, then redissolved in a minimal volume of CH_2Cl_2, and then filtered through a pad of silica, eluting with CH_2Cl_2. This organic phase was then washed successively with H_2O_2 (8 mL of a 15% v/v aq. solution), Na_2SO_3 (8 mL, sat., aq.) and H_2O (8 mL), then dried (Na_2SO_4), filtered and concentrated to afford the crude carbamate (2.34 g, 4.84 mmol, ~74%) as a colorless oil, which was used immediately to prevent spontaneous cyclisation to 1,3-ditosyltetrahydropyrimidin-2(1*H*)-one on standing and in solution.

To a rapidly stirring solution of this crude sulfonamide (200 mg, 0.414 mmol) in PhMe (4.1 mL) was added tetrabutylammonium hydrogensulfate (14.1 mg, 41.4 μmol) followed by 2,3-dibromopropene (108 mg, 0.539 mmol). NaOH (4.1 mL of a 25% w/v aq. solution) was added, and the mixture was stirred for 15 min, until starting material was consumed by tlc; then the layers were separated and extracted with Et_2O (2 × 5 mL). The combined organic fractions were dried (Na_2SO_4), filtered and concentrated *in vacuo*,* then redissolved in CH_2Cl_2 (3 mL) and cooled to 0 °C. TFA (1 mL) was added, and the reaction was stirred for 3 h, then diluted with H_2O (5 mL) and CH_2Cl_2 (3 mL) and the layers separated. The organic phase was dried

(Na_2SO_4), filtered and concentrated *in vacuo**, and the deprotected sulfonamide (68 mg, 0.135 mmol) was used immediately in the ynamide formation step (~21% yield over four steps).

** A water bath temperature of <20 °C was required during rotovap concentration to prevent decomposition. The intermediate must be used immediately or stored below –20 °C.*

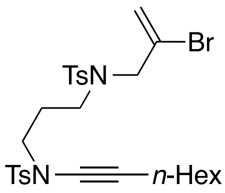

3m

To a mixture of crude sulfonamide (68 mg, 0.136 mmol), K_3PO_4 (58 mg, 0.271 mmol), 1,10-phenanthroline (20 mg, 0.109 mmol), $CuSO_4•5H_2O$ (14 mg, 0.821 mmol) and 1-bromo-1-octyne (34 mg, 0.271 mmol) was added anhydrous PhMe (0.70 mL) and the mixture heated to 85 °C for 16 h. The mixture was cooled to rt and passed through cotton wool, eluting with Et_2O, then the organic fraction was concentrated *in vacuo*. Purification by flash chromatography (petroleum ether / EtOAc, 9:1) afforded the ynamide **3m** as a colorless oil (42 mg, 0.080 mmol, 59%); $\mathbf{R}_f$ 0.38 (petroleum ether / EtOAc (7:3)); **^{1}H NMR** (400 MHz, $CDCl_3$) δ_H 7.76 (2H, d, *J* = 8.0 Hz, Ts*H*), 7.72 (2H, d, *J* = 8.0 Hz, Ts*H*), 7.35 (2H, d, *J* = 8.0 Hz, Ts*H*), 7.32 (2H, d, *J* = 8.0 Hz, Ts*H*), 5.84 (1H, d, *J* = 2.0 Hz, H5), 5.62 (1H, d, *J* = 2.0 Hz, H5′), 4.07 (2H, s, H3), 3.49-3.46 (2H, m, H1), 3.41-3.37 (2H, m, H2), 2.46 (3H, s, TsCH_3), 2.44 (3H, s, TsCH_3), 2.24 (2H, t, *J* = 7.0 Hz, H8), 1.52-1.43 (2H, m, H9), 1.36-1.23 (6H, m, H10, H11, H12), 0.89 (3H, t, *J* = 7.0 Hz, H13); **^{13}C NMR** (101 MHz, $CDCl_3$) δ_C 144.7, 143.9, 136.1, 134.1, 129.8, 129.8, 127.8, 127.6, 127.4, 120.5, 72.8, 70.5, 56.6, 50.6, 45.9, 31.3, 28.8, 28.5, 22.6, 21.7, 21.6, 18.4, 14.1. Data as reported previously.[16]

Synthesis of bromoalkynes 13a-e

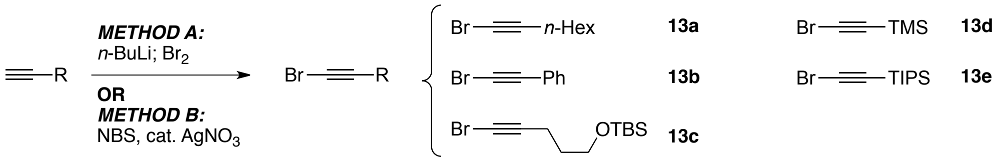

1-Bromooct-1-yne, 13a

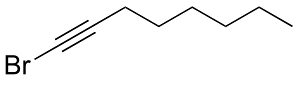

Preparation by General Procedure C: Using 1-octyne (6.69 mL, 45.4 mmol). Concentration *in vacuo* (care due to product volatility!) gave **13a** as a pale yellow oil (8.32 g, 44.0 mmol, 96%), which was used without further purification; $\mathbf{R}_f$ 0.70 (petroleum ether / EtOAc, 10:1); **^{1}H NMR** (400 MHz, $CDCl_3$) δ_H 2.19 (2H, t, *J* = 7.2 Hz, H3), 1.50 (2H, app quin, *J* = 7.2 Hz, H4), 1.43-1.20 (6H, m, H5-H7), 0.89 (3H, t, *J* = 7.2 Hz, H8); **^{13}C NMR** (101 MHz, $CDCl_3$) δ_C 80.4, 37.4, 31.3, 28.5, 28.3, 22.5, 19.7, 14.0. Data in accordance with literature values.[17]

Preparation by General Procedure D: Synthesised from oct-1-yne (15.0 mL, 102 mmol). The resulting crude material was purified by column chromatography (petroleum ether) to give **13a** as a colorless oil (18.4 g, 97.5 mmol, 96%); Data in accordance with the above.

(Bromoethynyl)benzene, 13b

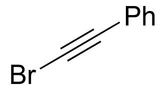

Preparation by General Procedure C: Using phenylacetylene (5.0 mL, 45.5 mmol). Concentration *in vacuo* gave **13b** as a pale yellow oil (8.45 g, 45.5 mmol, 99%), which was used without further purification; $\mathbf{R_f}$ = 0.68 (5:1, petroleum ether:EtOAc); **^{1}H NMR** (400 MHz, $CDCl_3$) δ_H 7.48 (2H, dd, *J* = 7.7, 1.9 Hz, Ar*H*), 7.38-7.29 (3H, m, Ar*H*); **^{13}C NMR** (101 MHz, $CDCl_3$) δ_C 132.0, 128.6, 128.3, 122.7, 80.0, 49.7. Data in accordance with literature values.[18]

Preparation by General Procedure D: Synthesised from phenylacetylene (5.00 mL, 45.5 mmol). The crude material was purified by column chromatography (petroleum ether) to give **13b** as a yellow oil (5.99 g, 33.1 mmol, 73%). Data in accordance with the above.

((5-Bromopent-4-yn-1-yl)oxy)(*tert*-butyl)dimethylsilane, 13c

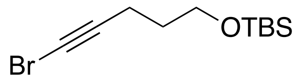

To a stirred suspension of NaH (60% in mineral oil, 1.43 g, 35.5 mmol, 1.08 equiv.) in THF (50 mL) at rt was added 4-pentyn-1-ol (2.77 g, 32.9 mmol, 1 equiv.), and this was left to stir for 30 min. Then TBSCl (5.38 g, 35.7 mmol, 1.08 eq.) was added portionwise. Following completion of the reaction as judged by tlc, the reaction was quenched with K_2CO_3 (sat., aq.), then diluted with water and extracted with ether. The combined organic layers were dried ($MgSO_4$) and concentrated to afford *tert*-butyldimethyl(pent-4-yn-1-yloxy)silane as a colorless oil (5.84 g, 29.4 mmol, 89%); **^{1}H NMR** (400 MHz, $CDCl_3$) δ_H 3.70 (2H, t, *J* = 6.0 Hz, H1), 2.28 (2H, dt, *J* = 7.1, 2.3 Hz, H3), 1.94 (1H, *J* = 2.3 Hz, H5), 1.73 (2H, m, H2), 0.90 (9H, s, SiMe$_2$*t-Bu*), 0.06 (6H, s, Si*Me*$_2$*t*-Bu). Data in agreement with literature values.[19]

This product was converted to the bromalkyne **13c** using General Procedure C: Using 7.86 g (39.6 mmol) of the alkyne, **13c** was obtained as a brown liquid (10.0 g, 36.0 mmol, 91%); **^{1}H NMR** (400 MHz, $CDCl_3$) δ_H 3.68 (2H, t, *J* = 6.0 Hz, H1), 2.29 (2H, t, *J* = 7.0 Hz, H3), 2.29 (2H, tt, *J* = 7.0, 6.0 Hz, H2), 0.89 (9H, s, SiMe$_2$*t-Bu*), 0.05 (6H, s, Si*Me*$_2$*t*-Bu); **^{13}C NMR** (101 MHz, $CDCl_3$) δ_C 79.9, 61.3, 37.7, 31.2, 25.9, 18.3, 16.1, 5.4. Data in agreement with literature values.[19]

This bromoalkyne could also be prepared using General Procedure D, according to the procedure of Sabitha.[19]

(Bromoethynyl)trimethylsilane, 13d

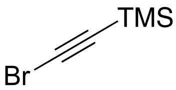

Preparation by General Procedure C: Using TMS acetylene (2.98 g, 30.2 mmol). This gave **13d** as a pale yellow liquid (5.32 g, 30.0 mmol, 99%); **^{1}H NMR** (400 MHz, $CDCl_3$) δ_H 0.18 (9H, s, TMS); **^{13}C NMR** (101 MHz, $CDCl_3$) δ_C 87.0, 61.4, 0.3. Data in agreement with literature values.[20]

Preparation by General Procedure D: Using TMS acetylene (5.00 mL, 35.4 mmol). Purification by flash chromatography (petroleum ether) have **13d** as a yellow oil (4.83 g, 27.3 mmol, 77%).

(Bromoethynyl)triisopropylsilane, 13e

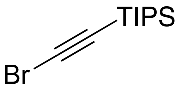

Preparation by General Procedure C: Using TIPS acetylene (0.81 g, 4.46 mmol). This gave **13e** as a light brown liquid (1.06 g, 4.06 mmol, 91%); **^{1}H NMR** (400 MHz, $CDCl_3$) δ_H 1.09 (21H, s, TIPS); **^{13}C NMR** (101 MHz, $CDCl_3$) δ_C 83.4, 61.7, 18.4, 11.2. Data in agreement with literature values.[21]

Bromoalkyne **13f** was prepared according to the following scheme:

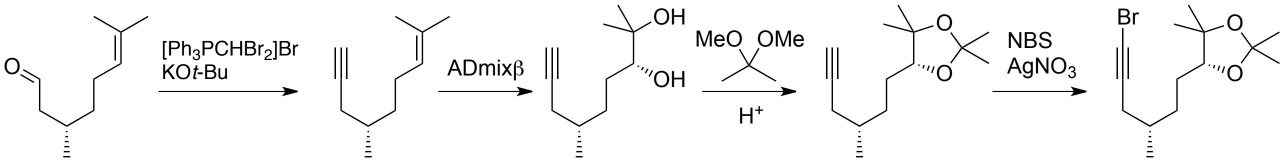

(*S*)-4,8-Dimethylnon-7-en-1-yne, S8

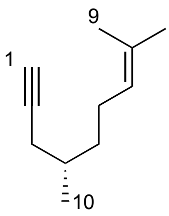

To a mixture of $[Ph_3PCHBr_2]Br$ (5.71 g, 11.1 mmol) in THF (50 mL) at rt was added KO*t*-Bu (1.18 g, 10.5 mmol) in a single portion, and the mixture was stirred for 2 min. (*S*)-Citronellal (855 mg, 5.54 mmol) was added and the mixture was stirred for 10 min, before addition of further KO*t*-Bu (3.11 g, 27.7 mmol). After 10 min, the reaction was quenched with brine (50 mL), concentrated *in vacuo** to remove the THF, then extracted with Et_2O (50 mL). The mixture was concentrated *in vacuo** and the product was triturated with petroleum ether (30 mL), again concentrated *in vacuo**, and then purified by column chromatography (petroleum ether eluent) to obtain the alkyne **S8** as a volatile colorless oil (557 mg, 67%); **R_f** 0.87 (petroleum ether); **IR** (thin film) ν_{max} / cm^{-1} 3311, 2995, 2918, 2063, 1455, 1378, 1110, 911, 736, 630; **^{1}H NMR** (400 MHz, $CDCl_3$) δ_H 5.11 (1H, br t, J = 7.0 Hz, H7), 2.19 (1H, ddd, J = 16.5, 5.5, 2.5 Hz, H3), 2.09 (1H, ddd, J = 16.5, 8.0, 2.5 Hz, H3′), 2.04-1.97 (2H, m, H6), 1.96 (1H, t, J = 2.5 Hz, H1), 1.75-1.64 (1H, m, H4), 1.69 (3H,

s, CH_3), 1.62 (3H, s, CH_3), 1.51-1.39 (1H, m, H5), 1.30-1.20 (1H, m, H5′), 1.00 (3H, d, J = 6.5 Hz, H10); **^{13}C NMR** (101 MHz, $CDCl_3$) δ_C 83.3, 79.0, 73.3, 69.4, 66.0, 33.2, 32.5, 29.2, 26.6, 25.7, 23.3, 19.6, 15.4; **HRMS** (ES$^+$) calc. for $C_{11}H_{21}O_2$ $[M+H]^+$ 185.1536, found 185.1531.

*NB *Due to the volatility of the alkyne, careful concentration in vacuo must be observed.*

(3*R*,6*S*)-2,6-Dimethylnon-8-yne-2,3-diol, 15

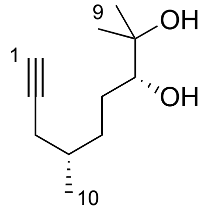

To a solution of (*S*)-4,8-dimethylnon-7-en-1-yne **S8** (294 mg, 1.96 mmol) in *t*-BuOH / H_2O (18 mL (1:1)) was added methanesulfonamide (222 mg, 1.96 mmol) followed by AD-mix-β (2.66 g). The mixture was stirred at rt for 72 h then Et_2O (20 mL) was added, and the layers separated. The organic fraction was washed with H_2O (20 mL), dried (Na_2SO_4), filtered and concentrated to afford the diol **15** as a colorless oil (314 mg, 87%); **R*f*** 0.37 (Et_2O); **IR** (thin film) ν_{max} / cm^{-1} 3452 (br), 3309, 2957, 2112, 1460, 1380, 1160, 1065, 963; **^{1}H NMR** (400 MHz, $CDCl_3$) δ_H 3.35-3.29 (1H, m, *H*7), 2.48-2.41 (1H, br d, J = 4.0 Hz, O*H*), 2.25-2.22 (1H, br s, O*H*), 2.19 (1H, ddd, J = 16.5, 5.5, 2.5 Hz, H6), 2.09 (1H, ddd, J = 16.5, 6.5, 2.5 Hz, H6′), 1.96 (1H, t, J = 2.5 Hz, H1), 1.75-1.64 (2H, m, H3), 1.55-1.45 (1H, m, H4) 1.32-1.23 (2H, m, H5), 1.20 (3H, s, H9), 1.15 (3H, s, H9′), 1.01 (3H, d, J = 6.5 Hz, H10; **^{13}C NMR** (101 MHz, $CDCl_3$) δ_C 83.3, 79.0, 73.3, 69.4, 66.0, 33.2, 32.5, 29.2, 26.6, 25.7, 23.3, 19.6, 15.4; **HRMS** (ES$^+$) calc. for $C_{11}H_{21}O_2$ $[M+H]^+$ 185.1536, found 185.1531; **$[\alpha]_D^{20}$** +10.2 (c 0.5, $CHCl_3$).

(*R*)-2,2,4,4-Tetramethyl-5-((*S*)-3-methylhex-5-yn-1-yl)-1,3-dioxolane, S9

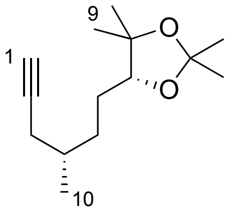

To a solution of diol **15** (352 mg, 1.57 mmol) in acetone (6.3 mL) was added dimethoxypropane (2.75 mL, 22.2 mmol) followed by *p*TSA.H_2O (12.9 mg, 0.0676 mmol), and the mixture was stirred for 1 h. The reaction was quenched with $NaHCO_3$ (10 mL, sat., aq.) and extracted with Et_2O (2 × 10 mL). The combined organic extracts were dried (Na_2SO_4), filtered and concentrated to afford the crude acetonide as a colorless oil. Purification by silica chromatography (petroleum ether / Et_2O, 9:1) gave the purified acetonide **S9** as a colorless oil (369 mg, 86%); **R*f*** 0.41 (petroleum ether/Et_2O (9:1)); **IR** (thin film) ν_{max} / cm^{-1} 3311, 2979, 2912, 2117, 1458, 1369, 1273, 1217, 1199, 1116, 998, 854, 629; **^{1}H NMR** (400 MHz, $CDCl_3$) δ_H 3.66 (1H, dd, J = 8.5, 4.5 Hz, *H*7), 2.21 (1H, ddd, J = 16.5, 5.5, 3.0 Hz, H6), 2.10 (1H, ddd, J = 16.5, 7.0, 3.0 Hz, H6′), 1.97

(1H, t, J = 2.5 Hz, H1), 1.78-1.61 (2H, m, H3), 1.56-1.43 (1H, m, H4) 1.42 (3H, s, CH_3), 1.33 (3H, s, CH_3), 1.25 (3H, s, CH_3), 1.10 (3H, s, CH_3), 1.02 (3H, d, J = 7.0 Hz, H10; **^{13}C NMR** (101 MHz, $CDCl_3$) δ_C 106.6, 83.7, 83.2, 80.3, 69.4, 33.2, 32.6,28.7, 27.0, 26.3, 25.9, 23.0, 19.3; **HRMS** (ES^+) calc. for $C_{14}H_{25}O_2$ $[M+H]^+$ 225.1855, found 185.1849; **$[\alpha]_D^{20}$** –16.2 (c 1, $CHCl_3$).

(*R*)-5-((*S*)-6-Bromo-3-methylhex-5-yn-1-yl)-2,2,4,4-tetramethyl-1,3-dioxolane, 13f

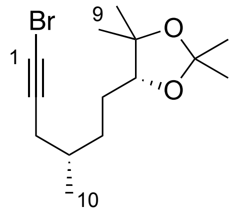

To a solution of terminal acetylene **S9** (306 mg, 1.37 mmol) in acetone (2.7 mL) was added $AgNO_3$ (23.3 mg, 0.137 mmol), and the mixture was stirred for 5 min before addition of *N*-bromosuccinimide (255 mg, 1.44 mmol). After 2 h, the mixture was diluted with Et_2O (10 mL) and filtered through celite, eluting with Et_2O (10 mL), then the mixture was concentrated. Rapid purification by flash chromatography (petroleum ether / Et_2O, 9:1 eluent) gave the bromoalkyne **13f** as a colorless oil (369 mg, 86%), which was used immediately as the product underwent decomposition on standing and in the freezer at –20 °C; **R$_f$** 0.59 (petroleum ether/Et_2O (4:1)); **^{1}H NMR** (400 MHz, $CDCl_3$) δ_H 3.61 (1H, dd, J = 8.5, 4.0 Hz, H7), 2.18 (1H, dd, J = 16.5, 5.5 Hz, H6), 2.07 (1H, dd, J = 16.5, 7.0, H6′), 1.71-1.51 (2H, m, H3), 1.51-1.40 (1H, m, H4) 1.37 (3H, s, CH_3), 1.29 (3H, s, CH_3), 1.21 (3H, s, CH_3), 1.05 (3H, s, CH_3), 0.96 (3H, d, J = 7.0 Hz, H10).

Bromoalkyne **13g** was prepared according to the following scheme:

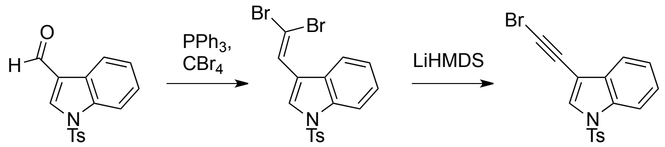

3-(2,2-Dibromoethenyl)-1-(phenylsulfonyl)indole, S10

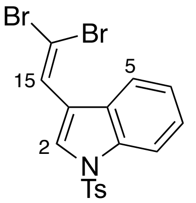

To a solution of PPh_3 (8.57 g, 32.6 mmol) in CH_2Cl_2 (35 mL) at –20 °C was added CBr_4 (5.41 g, 16.3 mmol) in a single portion. The mixture was stirred for 10 min, then cooled to –78 °C before dropwise addition of a solution of *N*-tosylindole-3-carboxaldehyde (1.63 g, 5.44 mmol) in CH_2Cl_2 (10 mL). Stirring was continued for 5 min, then the mixture was warmed to rt before addition of hexane (40 mL). The precipitate was filtered, concentrated *in vacuo*, redissolved in $CHCl_3$ (20 mL) and reconcentrated, then purified *via* rapid

chromatography through a pad of silica (petroleuem ether / EtOAc, 1:3) to afford the vinyl dibromide **S10** as a pale yellow solid (2.03 g, 82%); **mp** 113–118 °C; **R*f*** 0.42 (petroleum ether/EtOAc (1:3)); **^{1}H NMR** (400 MHz, $CDCl_3$) δ_H 8.30 (1H, d, *J* = 1.0 Hz, H15), 8.00 (1H, dt, *J* = 8.5, 1.0 Hz, H5), 7.80 (2H, dt, *J* = 8.5, 2.0 Hz, TsH), 7.55 (1H, d, *J* = 0.5 Hz, H2), 7.53 (1H, dt, *J* = 7.5, 1.0 Hz, H8), 7.37 (1H, ddd, *J* = 8.5, 7.5, 1.0 Hz, H7), 7.31-7.28 (1H, m, H6), 7.26-7.23 (2H, m, TsH), 2.35 (3H, s, TsC*H*$_3$); **^{13}C NMR** (101 MHz, $CDCl_3$) δ_C 145.5, 135.0, 134.2, 130.1, 129.5, 127.0, 125.6, 125.1, 123.7, 118.9, 117.5, 113.8, 90.4, 21.8; **LRMS** (ESI$^+$) $C_{16}H_{11}NO_2SBr_2$ 441.0 (100, [M+H]$^+$). Data are in agreement with the literature.[22]

3-(Bromoethynyl)-1-tosyl-1*H*-indole, 13g

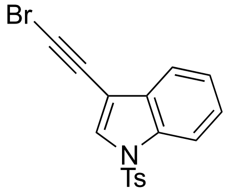

To a solution of vinyl dibromide (900 mg, 1.98 mmol) in THF (15 mL) at –78 °C was added LHMDS (2.37 mL of a 1 M solution in THF, 2.37 mmol) dropwise and the mixture was stirred for 20 min, before being quenched with NH_4Cl (10 mL, sat., aq.). The product was extracted with EtOAc (2 × 10 mL), then the organic fractions were combined, dried ($MgSO_4$), filtered and concentrated, giving the bromoalkyne as a colorless oil (696 mg, 94%) which was used without further purification. **IR** (thin film) ν_{max} / cm^{-1} 3033, 2954, 2201, 1597, 1446, 1375, 1175; **^{1}H NMR** (400 MHz, $CDCl_3$) δ_H 7.97 (1H, dt, *J* = 8.5, 1.0 Hz, H5), 7.81-7.74 (3H, m, H2, H11), 7.64 (1H, app dq, *J* = 8.0, 0.5 Hz, H8), 7.36 (1H, ddd, *J* = 8.5, 7.0, 1.0 Hz, H7), 7.32-7.28 (1H, m, H6), 7.26-7.23 (2H, m, H12), 2.36 (3H, s, TsC*H*$_3$); **^{13}C NMR** (101 MHz, $CDCl_3$) δ_C 145.6, 134.9, 134.1, 130.8, 130.2, 130.0, 127.1, 125.7, 124.0, 120.6, 113.7, 104.8, 71.6, 53.6, 21.8; **HRMS** (ES$^+$) calc. for $C_{17}H_{12}BrNNaO_2S^+$ ([M+Na]$^+$) 395.9664, found 395.9674.

Bromoalkyne **13h** was prepared according to the following scheme:

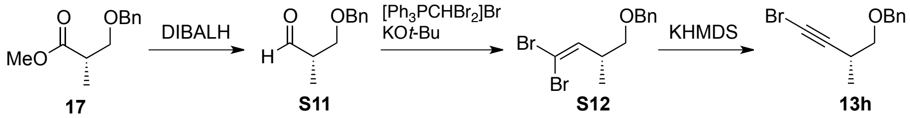

(*R*)-(((4-Bromo-2-methylbut-3-yn-1-yl)oxy)methyl)benzene, 13h

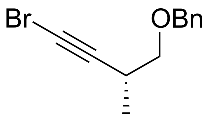

To a solution of methyl (*R*)-3-(benzyloxy)-2-methylpropanoate (1.00 g, 4.80 mmol) in CH_2Cl_2 (13 mL) at –78 °C was added dropwise a solution of DIBAL (4.80 mL of a 1 M solution in CH_2Cl_2, 4.80 mmol). The mixture was maintained at –78 °C for 1 h, then quenched carefully with MeOH at this temperature. Sodium potassium tartrate (10 mL, sat., aq.) was added, and the mixture was stirred at rt for 2 h. The layers were separated and the product was extracted with CH_2Cl_2 (2 × 10 mL). The organics extracts were combined,

dried (Na_2SO_4), filtered and concentrated *in vacuo* (20 °C) to obtain the crude aldehyde **S11**, which was used immediately to prevent racemisation (assumed quantitative). To a mixture of [Ph_3PCHBr_2]Br (4.95 g, 9.61 mmol) in THF (30 mL) at rt was added KO*t*-Bu (1.03 g, 9.18 mmol), and the mixture was stirred for 20 min. To the generated ylide was added crude (*R*)-3-(benzyloxy)-2-methylpropanal **S11** (856 mg, 4.80 mmol). The mixture was stirred for 10 min at rt, then diluted with petroleum ether (100 mL), and the precipitate removed by filtration, washing with petroleum ether (100 mL). Following concentration, rapid purification through a pad of silica (petroleum ether / Et_2O, 19:1 eluent) gave the vinyl dibromide **S12** as a colorless oil (615 mg), which was used directly in the subsequent step.

To a solution of crude vinyl dibromide **S12** (524 mg, 1.57 mmol) in THF (7 mL) –78 °C was added KHMDS (3.34 mL of a 0.47 M solution in toluene, 1.57 mmol) dropwise. The mixture was stirred at –78 °C for 30 min, then warmed to –20 °C for 30 min, then quenched at this temperatre with NH_4Cl (5 mL, sat., aq.). The product was extracted with Et_2O (3 × 10 mL), washed with brine (10 mL) then dried ($MgSO_4$), filtered and concentrated *in vacuo*.* The product was purified by flash chromatography (petroleum ether / Et_2O, 19:1 eluent) to afford the **13h** as a colorless oil (354 mg, 89%).

* *NB Water bath temperature of <20 °C was used to minimise decomposition.* Product was used immediately as underwent significant decomposition at ambient temperature and in solution.

R_f 0.61 (petroleum ether/Et_2O (9:1)); **^{1}H NMR** (400 MHz, $CDCl_3$) δ_H 7.45-7.28 (5H, m, Ph*H*), 4.57 (2H, br s, PhCH_2), 3.52 (1H, dd, *J* = 9.0, 6.5 Hz, OCH_AH_B), 3.38 (1H, dd, *J* = 9.0, 7.0 Hz, OCH_AH_B), 2.78 (1H, sxt, *J* = 7.0 Hz, C*H*Me), 1.23 (3H, d, *J* = 7.0 Hz, CH_3); **^{13}C NMR** (101 MHz, $CDCl_3$) δ_C 138.2, 128.5, 127.7, 127.7, 82.2, 73.1, 73.6, 39.4, 28.0, 17.5; **LRMS** (ESI$^+$) $C_{12}H_{13}BrNaO$ [M+Na]$^+$ 275.0; **$[\alpha]_D^{20}$** –8.5 (c 1, $CHCl_3$).

Bromoenynamide formation: Ynamide formation was generally achieved using either the Hsung (or Evano) coupling of bromoalkynes (or dibromoalkenes) with the relevant amides, see General Procedure E. The characterization of ynamides **3a-m** has been reported in our earlier communications.[16, 23] For ynamides **3n-q**, problems encountered using these copper-catalyzed methods necessitated ynamide synthesis using the Witulski method of alkynyl iodonium triflate salts;[24] characterization data for these compounds has also been described previously. [16, 23]

4. Stannane Synthesis

All stannanes employed in this work are known compounds. These were prepared according to the following procedures:

Tributylpropenylstannane (19a): Prepared according to the procedure of Moloney et al.[25]

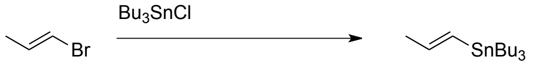

19a

Tributylstyrenylstannane (19b): Prepared as **19a**.

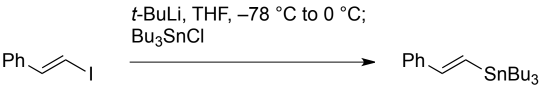

19b

Tributylvinylstannane (19c): Prepared according to the procedure of Seyferth and Stone.[26]

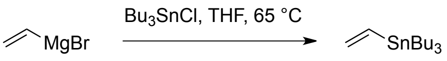

19c

Tributyl(3,4-dihydro-2*H*-pyran-6-yl)stannane (19e): Prepared according to the procedure of Quayle et al.[27]

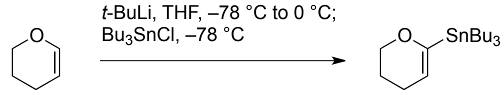

19e

5. Ynamide carbopalladation / Stille coupling – VT NMR experiments

VT ^{1}H NMR experiment (Main paper, Figure 2) to determine reaction initiation temperature and the evolution of product and intermediate

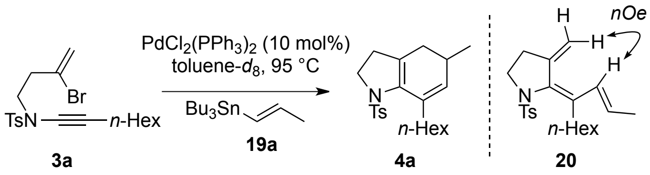

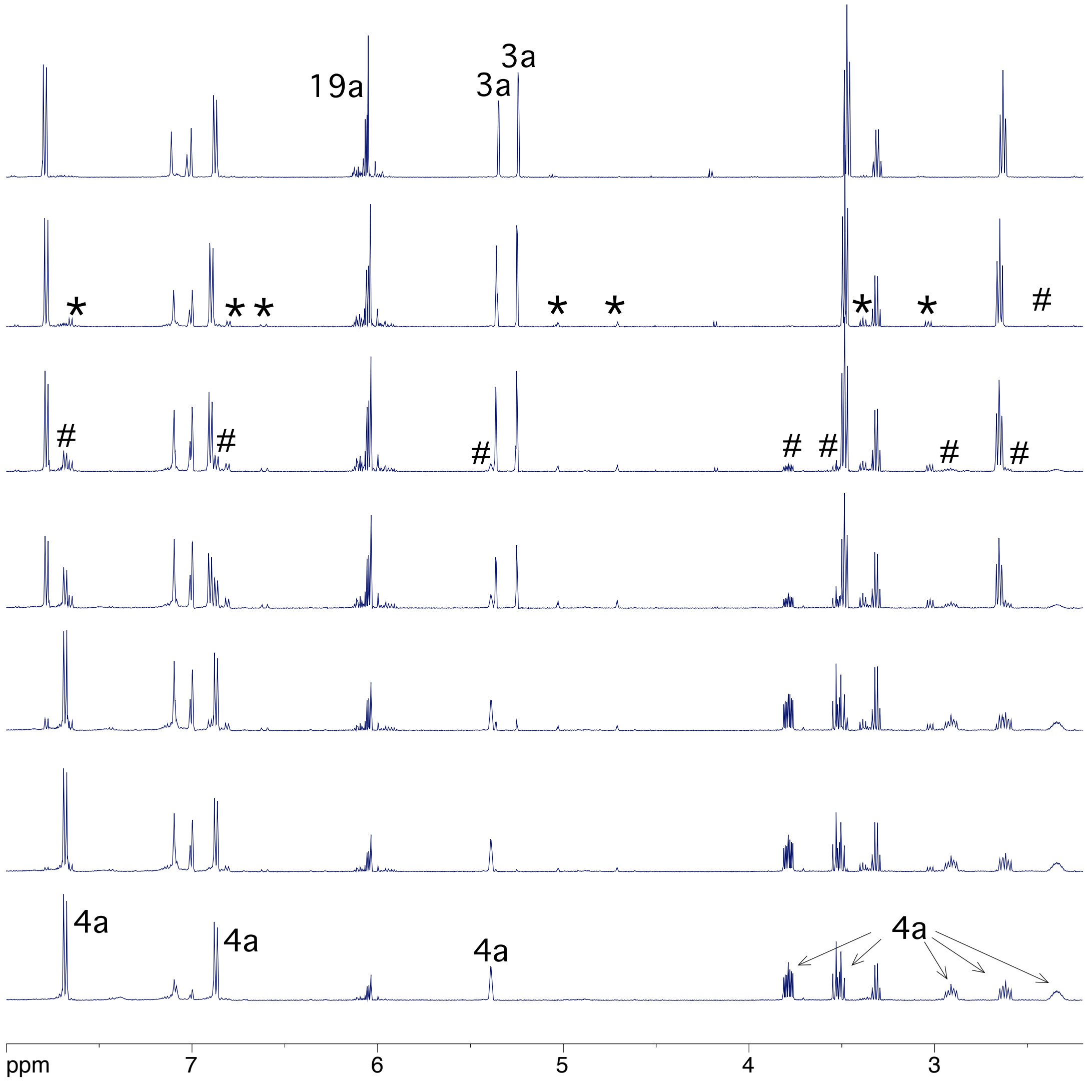

Procedure: To an NMR tube containing $PdCl_2(PPh_3)_2$ (3.9 mg, 0.0057 mmol, 10 mol%) under Ar was added a degassed (Ar bubbling) solution of stannane **19a** (30 mg, 0.091 mmol) and bromoenynamide **3a** (23.5 mg, 0.057 mmol) in d_8-toluene (0.8 mL). The NMR tube was placed in a 500 MHz spectrometer at rt, then gradually heated to 95 °C in 5 °C increments until intiation of reaction was observed. The reaction was held at 95 °C for ~45 min until complete consumption of starting material and intermediate.

Isolation of (*E*)-2-((*E*)-dec-2-en-*4*-ylidene)-3-methylene-1-tosylpyrrolidine (20):

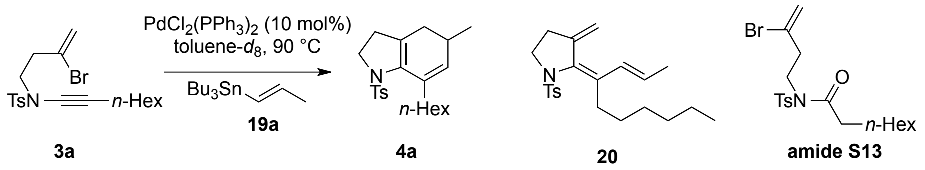

Isolation of (*E*)-2-((E)-dec-2-en-*4*-ylidene)-3-methylene-1-tosylpyrrolidine (**20**): To an NMR tube containing $PdCl_2(PPh_3)_2$ (3.4 mg, 0.0048 mmol, 10 mol%) under Ar was added a degassed (Ar bubbling, 15 min) solution of the bromoenynamide **3a** (20 mg, 0.048 mmol) and stannane **19a** (26 mg, 0.078 mmol) in d_8-toluene (0.8 mL). Alongside this, an equivalent sample was made up containing bromoenynamide and stannane in d_8-toluene, but no catalyst. A room temperature ^{1}H NMR spectrum of both samples was taken to acquire a spectrum at t=0; then, the control sample (no catalyst) was heated to 90 °C for the purpose of retuning and shimming of the probe and sample. The reaction sample was then inserted into the pre-heated spectrometer, and monitored by ^{1}H NMR with acquisitions at 2 minute intervals. The reaction reached a peak of intermediate production at 12-13 minutes. After cooling to rt (i.e. reaction was halted), the sample was concentrated, and purified by flash chromatography (2% EtOAc in petroleum ether). This afforded 3.1 mg of a fraction containing **20**, **3a**, **4a**, and the amide **S13** in a **1 : 0.23 : 0.8 : 0.5** ratio. The amide **S13** is believed to arise from partial hydrolysis of the starting material in the course of exposure to SiO_2 during purification by slow column chromatography. This procedure was performed three times with equivalent results.

The stereochemistry of **20** was proven by nOe enhancement between protons H2 and H3; the assignment of structure was made on the basis of ^{1}H NMR chemical shifts for these protons and the associated methyl group, and by analogy to trienes prepared and isolated in earlier work from our group.

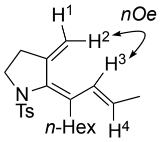

20

This triene was then subjected to a further VT NMR experiment, which confirmed complete conversion to the dienamide **4a**, in the absence of catalyst. Notably, consumption of this intermediate was also observed in all standard VT NMR reactions (i.e. in the presence of the catalyst), according to our optimization experiments and as shown in Figure 2 of the paper.

6. Ynamide carbopalladation / Stille coupling / electrocyclization cascades

These were carried out using **General Procedure F**.

7-Hexyl-5-methyl-1-tosyl-2,3,4,5-tetrahydro-1*H*-indole, 4a

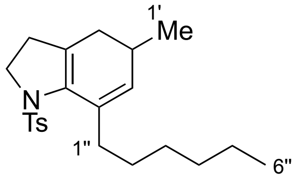

Synthesised from ynamide **3a** (100 mg, 0.243 mmol), stannane **19a** (129 mg, 0.388 mmol), and $PdCl_2(PPh_3)_2$ (17.0 mg, 0.024 mmol, **10 mol%**) using General Procedure F, to afford **4a** as a yellow oil (84.0 mg, 0.225 mmol, 92%); **R*f*** 0.30 (petroleum ether / ether, 3:1); **IR** (thin film) ν_{max} / cm^{-1} 2926, 2855, 1598, 1493, 1352, 1164, 1132; **^{1}H NMR** (500 MHz, $CDCl_3$) δ_H 7.68 (2H, d, *J* = 8.2 Hz, Ts*H*), 7.27 (2H, d, *J* = 8.0 Hz, Ts*H*), 5.38 (1H, s, H6), 3.87 (1H, ddd, *J* = 13.0, 8.0, 3.3 Hz, H2), 3.66 (1H, dt, *J* = 13.1, 9.4 Hz, H2), 2.65-2.59 (1H, m, H5), 2.44 (3H, s, Ts*Me*), 2.42-2.32 (2H, m, H4), 1.93 (1H, dd, *J* = 17.4, 7.8 Hz, H3), 1.74 (1H, dd, *J* = 17.5, 2.3 Hz, H3), 1.69-1.24 (10H, m, H1''-5''), 1.07 (3H, d, *J* = 6.5 Hz, H1'), 0.88 (3H, t, *J* = 6.6 Hz, H6''); **^{13}C NMR** δ_C; 143.7, 138.5, 134.1, 133.1, 132.4, 129.1, 128.4, 128.2, 51.5, 32.0, 31.8, 31.7, 30.5, 29.5, 28.9, 28.8, 22.6, 21.6, 20.2, 14.1; **HRMS** (ES^+) calc. for $C_{22}H_{31}NNaO_2S$ $[M+Na]^+$ 396.1968; found 396.1961. Also prepared by General Procedure **F** using ynamide **3a** (50 mg, 0.12 mmol), stannane **19a** (63 mg, 0.19 mmol), and $PdCl_2(PPh_3)_2$ (~1.0 mg, 0.0012 mmol, **1 mol%)**, with heating for 18 h. Purification *via* flash chromatography (petroleum ether + 1% NEt_3 → petroleum ether / EtOAc (10:1) + 1% NEt_3) afforded amidodiene **4a** as a pale yellow oil (40.7 mg, 0.109 mmol, 90%).

7-(3-(*tert*-Butyldimethylsilyloxy)propyl)-5-methyl-1-tosyl-2,3,4,5-tetrahydro-1*H*-indole, 4b

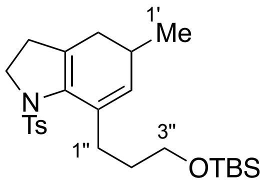

Synthesised from ynamide **3c** (100 mg, 0.20 mmol), stannane **19a** (106 mg, 0.32 mmol) and $PdCl_2(PPh_3)_2$ (14.0 mg, 0.020 mmol, **10 mol%**) using General Procedure **F** to afford **4b** as a yellow oil (84.0 mg, 0.182 mmol, 91%); **R*f*** 0.38 (petroleum ether / EtOAc, 3:1); **IR** (thin film) ν_{max} / cm^{-1} 2957, 2738, 1597, 1493, 1353, 1254, 1165; **^{1}H NMR** (400 MHz, $CDCl_3$) δ_H 7.66 (2H, d, *J* = 8.3 Hz, Ts*H*), 7.26 (2H, d, *J* = 8.3 Hz, Ts*H*), 5.39 (1H, s, H6), 3.90-3.84 (1H, m, H2), 3.70-3.63 (1H, m, H2), 3.67 (2H, t, *J* = 7.4 Hz, H3''), 3.62 (2H, t, *J* = 7.4 Hz, H1''), 2.71-2.63 (1H, m, H5), 2.43 (3H, s, Ts*Me*), 2.42-2.35 (2H, m, H4), 1.94 (1H, dd, *J* = 17.5, 8.0 Hz, H3), 1.83-1.50 (3H, m, H2'', H3), 1.06 (3H, d, *J* = 7.1 Hz, H1'), 0.90 (9H, s, $OSiMe_2$*t-Bu*), 0.05 (6H, s, OSi*Me*$_2$*t*-Bu); **^{13}C NMR** (101 MHz, $CDCl_3$) δ_C 143.7, 138.3, 133.3, 133.0, 132.8 ,129.2, 128.7, 128.4, 62.9, 51.5, 31.8, 30.5, 29.5, 28.2, 26.0, 21.6, 20.2, 18.4, 13.5, –5.2; **HRMS** (ES^+) calc. for $C_{25}H_{39}NNaO_3SSi$ $[M+Na]^+$ 484.2312, found 484.2304.

Also prepared by General Procedure **F** using ynamide **3c** (50 mg, 0.10 mmol), stannane **19a** (53 mg, 0.16 mmol) and $PdCl_2(PPh_3)_2$ (~0.9 mg, 0.001 mmol, **1 mol%)**, with heating for 18 h. Purification *via* column chromatography (petroleum ether + 1% NEt_3→petroleum ether / EtOAc (10:1) + 1% NEt_3) afforded amidodiene **4b** as a pale yellow oil (34 mg, 0.076 mmol, 76%).

5-Methyl-7-phenyl-1-tosyl-2,3,4,5-tetrahydro-1*H*-indole, 4c

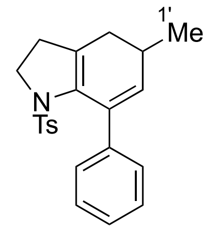

Synthesised from ynamide **3b** (50 mg, 0.124 mmol), stannane **19a** (65 mg, 0.198 mmol), and $PdCl_2(PPh_3)_2$ (8.7 mg, 0.012 mmol, **10 mol%**) using General Procedure F to afford **4c** as a pale brown oil (21.9 mg, 0.060 mmol, 48%); **R*f*** 0.27 (petroleum ether / ether, 3:1); **IR** (thin film) ν_{max} / cm^{-1} 2925, 1598, 1493, 1446, 1353, 1164, 1090; **^{1}H NMR** (400 MHz, $CDCl_3$) δ_H 7.62 (2H, d, *J* = 8.2 Hz, Ts*H*), 7.43 (2H, d, *J* = 7.5 Hz, Ph*H*), 7.34 (2H, t, *J* = 7.5 Hz, Ph*H*), 7.25 (3H, d, *J* = 8.0 Hz, Ph*H* and Ts*H*), 5.82 (1H, d, *J* = 3.2 Hz, H6), 3.95 (1H, ddd, *J* = 12.6, 8.6, 3.7 Hz, H2), 3.82 (1H, dt, *J* = 12.9, 9.5 Hz, H2), 2.70-2.58 (1H, m, H5), 2.44 (3H, s, Ts*Me*), 2.09 (1H, dd, *J* = 17.5, 8.0 Hz, H4), 2.02-1.80 (3H, m, H4 and 2 x H3), 1.18 (3H, d, *J* = 7.1 Hz, H1'); **^{13}C NMR** (101 MHz, $CDCl_3$) δ_C 143.6, 139.2, 136.3, 134.1, 133.5, 133.2, 132.2, 129.2, 128.2, 127.8, 126.9, 126.7, 50.7, 31.4, 31.1, 30.0, 21.6, 20.0; **HRMS** (ES^+) calc. for $C_{22}H_{23}NNaO_2S$ $[M+H]^+$ 388.1342, found 388.1332.

Also prepared by General Procedure F using ynamide **3b** (50 mg, 0.124 mmol), stannane **19a** (63 mg, 0.19 mmol) and $PdCl_2(PPh_3)_2$ (0.8 mg, 0.0012 mmol, **1 mol%**) with heating for 18 h. Purification *via* column chromatography (petroleum ether + 1% NEt_3 → petroleum ether / EtOAc (10:1) + 1% NEt_3) afforded amidodiene **4c** as a yellow oil (29.5 mg, 0.081 mmol, 65%).

7-Hexyl-5-phenyl-1-tosyl-2,3,4,5-tetrahydro-1*H*-indole, 4d

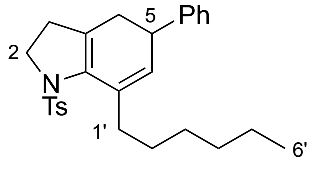

Synthesised from ynamide **3a** (100 mg, 0.24 mmol), stannane **19b** (153 mg, 0.39 mmol), and $PdCl_2(PPh_3)_2$ (17.0 mg, 0.024 mmol, **10 mol%**) using General Procedure F to afford 4**d** as a yellow oil (81.6 mg, 0.18 mmol, 77%); **R*f*** 0.23 (petroleum ether / ether, 3:1); **^{1}H NMR** (500 MHz, $CDCl_3$) δ_H 7.69 (2H, d, *J* = 8.2 Hz, Ts*H*), 7.39-7.28 (5H, m, Ar*H*), 7.22 (2H, d, *J* = 8.2 Hz, Ts*H*), 5.62 (1H, s, H6), 3.96-3.88 (2H, m, H2), 3.64-3.57 (1H, m, H5), 2.77-2.70 (1H, m, H1'), 2.50-2.40 (1H, m, H1'), 2.44 (3H, s, $TsCH_3$), 2.37-2.16 (2H, m, H4), 1.69-1.59 (2H, m, H3), 1.57-1.27 (8H, m, H1'-H5'), 0.90 (3H, t, J = 7.0 Hz, H6'; **HRMS** (ES+) calc. for

$C_{27}H_{34}NO_2S$ $[M+H]^+$ 436.2305, found 436.2299. The data for this compound are as previously reported.[16]

7-Hexyl-1-tosyl-2,3,4,5-tetrahydro-1*H*-indole, 4e

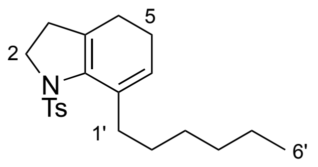

Synthesised from ynamide **3a** (100 mg, 0.24 mmol), stannane **19c** (123 mg, 0.39 mmol), and $PdCl_2(PPh_3)_2$ (17.0 mg, 0.024 mmol, **10 mol%**) using General Procedure F to afford **4e** as a yellow oil (63 mg, 0.175 mmol, 72%); **R*f*** 0.44 (petroleum ether / EtOAc, 3:1); **IR** (thin film) ν_{max} / cm^{-1} = 2927, 1727, 1598, 1463, 1378, 1288, 1163, 1073; **^{1}H NMR** (500 MHz, $CDCl_3$) δ_H 7.67 (2H, d, *J* = 8.0 Hz, Ts*H*), 7.26 (2H, d, *J* = 8.0 Hz, Ts*H*), 5.61 (1H, t, *J* = 4.5 Hz, H6), 3.76 (2H, t, *J* = 7.5 Hz, H2), 2.56-2.49 (2H, m, H5), 2.44 (3H, s, Ts*Me*), 2.16-2.06 (2H, m, H6), 1.99-1.91 (2H, m, H4), 1.63 (2H, t, *J* = 7.5 Hz, H3), 1.50-1.39 (2H, m, H1′), 1.45-1.25 (2H, m, H2′); 1.36-1.25 (6H, m, H3′-5′), 0.88 (3H, t, *J* = 7.0 Hz, H6′); **^{13}C NMR** (125 MHz, $CDCl_3$) δ_C 143.7, 138.7, 135.1, 132.9, 132.4, 129.1, 128.3, 120.8, 51.2, 32.1, 31.8, 30.5, 28.9, 28.8, 23.7, 23.0, 21.6, 14.1, 13.6; **HRMS** (ES+) calc. for $C_{21}H_{29}NNaO_2S$ $[M+Na]^+$ 382.1811, found 382.1807. The data for this compound are as previously reported.[16]

7-Hexyl-1-tosyl-5-(trimethylsilyl)-2,3,4,5-tetrahydro-1*H*-indole, 4f

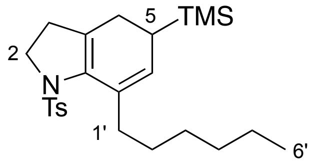

Synthesised from ynamide **3a** (100 mg, 0.24 mmol), stannane **19d** (151 mg, 0.39 mmol), and $PdCl_2(PPh_3)_2$ (17.0 mg, 0.024 mmol, **10 mol%**) using General Procedure F to afford **4f** as a yellow oil (93 mg, 0.215 mmol, 89%); **R*f*** 0.52 (petroleum ether / EtOAc. 3:1); **IR** (thin film) ν_{max} / cm^{-1} = 2926, 1597, 1464, 1352, 1249, 1164, 838; **^{1}H NMR** (400 MHz, $CDCl_3$) δ_H 7.69 (2H, d, *J* = 8.2 Hz, Ts*H*), 7.27 (2H, d, *J* = 8.2 Hz, Ts*H*), 5.45 (1H, s, H6), 3.86 (1H, ddd, *J* = 12.9, 7.9, 2.4 Hz, H2), 3.63 (1H, ddd, *J* = 12.9, 10.5, 9.1 Hz, H2), 2.81-2.75 (1H, m, H5), 2.45 (3H, s, Ts*Me*), 2.32-2.22 (1H, m, H3), 2.01-1.73 (2H, m, H4), 1.79 (1H, dd, *J* = 16.6, 8.8 Hz, H1'), 1.69-1.23 (9H, m, H1'-5'), 0.93 (3H, t, *J* = 7.3 Hz, H6'), 0.05 (9H, s, TMS); **^{13}C NMR** (101 MHz, $CDCl_3$) δ_C 143.7, 138.6, 134.8, 133.4, 132.4, 129.1, 128.3, 122.5, 51.5, 32.5, 31.8, 30.5, 29.2, 28.8, 24.5, 22.7, 22.7, 21.6, 14.1, -3.2; **HRMS** (ES+) calc. for $C_{24}H_{37}NNaO_2SSi$ $[M+Na]^+$ 454.2206, found 454.2198.

7-(3-(*tert*-Butyldimethylsilyloxy)propyl)-5-phenyl-1-tosyl-2,3,4,5-tetrahydro-1*H*-indole, 4g

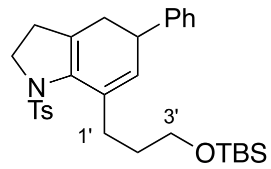

Synthesised from ynamide **3c** (100 mg, 0.20 mmol), stannane **19b** (106 mg, 0.32 mmol), and $PdCl_2(PPh_3)_2$ (14.0 mg, 0.020 mmol, **10 mol%**) using General Procedure F to afford **4g** as a pale yellow oil (92 mg, 0.175 mmol, 89%); **R*f*** 0.39 (petroleum ether / EtOAc, 3:1); **IR** (thin film) ν_{max} / cm^{-1} = 2929, 2857, 1598, 1493, 1352, 1251, 1164; **^{1}H NMR** (400 MHz, $CDCl_3$) δ_H 7.68 (2H, d, *J* = 8.1 Hz, Ts*H*), 7.38-7.25 (5H, m, Ar*H*), 7.22 (2H, d, *J* = 8.1 Hz, Ts*H*), 5.65 (1H, s, H6), 3.95-3.55 (2H, m, H2), 3.68 (2H, t, *J* = 6.7Hz, H3'), 3.60 (2H, m, *J* = 7.7 Hz, H1'), 2.77 (1H, m, H5), 2.59-2.46 (2H, m, H4), 2.44 (3H, s, Ts*Me*), 2.36-2.17 (2H, m, H3), 1.85-1.61 (2H, m, H2'), 0.91 (9H, s, $OSiMe_2$*t-Bu*), 0.08 (6H, s, OSi*Me*$_2$*t*-Bu); **^{13}C NMR** (101 MHz, $CDCl_3$) δ_C 144.9, 143.8, 138.6, 134.6, 133.0, 132.2, 129.2, 128.5, 128.4, 127.5, 126.5, 125.8, 63.0, 51.5, 41.1, 32.4, 31.9, 30.4, 28.3, 26.0, 21.7, 18.4, -5.2; **HRMS** (ES^+) calc. for $C_{30}H_{41}NNaO_3SSi$ $[M+Na]^+$ 546.2469, found 546.2472.

9-Hexyl-8-tosyl-2,3,4,4a,5,6,7,8-octahydropyrano[3,2-*f*]indole, 4h

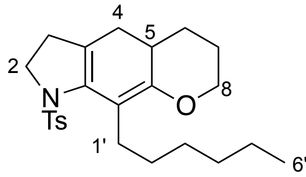

Prepared from bromoenynamide **3a** (50 mg, 0.12 mmol), stannane **19e**[27] (71 mg, 0.19 mmol) and $PdCl_2(PPh_3)_2$ (8.0 mg, 0.012 mmol, **10 mol%**) using General Procedure F with heating for 15 h. Purification *via* column chromatography (petroleum ether→petroleum ether / EtOAc, 20:1) afforded **4h** as a pale yellow oil (32 mg, 0.077 mmol, 64%); **R*f*** 0.22 (petroleum ether / EtOAc, 10:1); **IR** (thin film, ν_{max} / cm^{-1}) 2954, 2926, 2857, 2255, 1677, 1627, 1598, 1454, 1351, 1162, 1119, 1089, 910; **^{1}H NMR** (500 MHz, $CDCl_3$) δ_H 7.70 (2H, d, *J* = 8.5 Hz, Ts*H*), 7.26 (2H, d, *J* = 8.0 Hz, Ts*H*), 3.96 (2H, dt, *J* = 7.0, 3.5 Hz, H8), 3.68 (1H, ddd, *J* = 12.5, 8.5, 6.0 Hz, H2), 3.55 (1H, ddd, *J* = 12.5, 9.0, 6.5 Hz, H2), 2.81 (1H, ddd, *J* = 13.5, 9.0, 7.0 Hz, H1'), 2.42 (3H, s, TsC*H*$_3$), 2.38-2.31 (1H, m, H1'), 2.24-2.12 (1H, m, H5), 2.10-2.01 (1H, m, H4/6), 1.94-1.84 (3H, m, H4, H6 and H7), 1.85-1.76 (2H, m, H4/6 and H7), 1.48-1.40 (2H, m, H3 and H2'), 1.37-1.22 (7H, m, H2' and H3'-5'), 1.05 (1H, dddd, *J* = 13.0, 8.5, 7.0, 6.0 Hz, H3), 0.87 (3H, t, *J* = 7.0 Hz, H6'); **^{13}C NMR** (125 MHz, $CDCl_3$) δ_c 147.8, 143.8, 136.0, 135.5, 129.7, 127.9, 126.4, 103.3, 66.0, 51.3, 38.0, 34.5, 31.9, 29.2, 28.6, 27.8, 27.2, 25.2, 23.0, 22.9, 21.7, 14.3; **HRMS** (ES^+) calc. for $C_{24}H_{34}NO_3S^+$ $[M+H]^+$ 416.2254, found 416.2242.

5-Methyl-1-tosyl-7-(trimethylsilyl)-2,3,4,5-tetrahydro-1*H*-indole, 4i

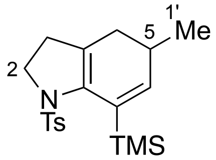

Synthesised from ynamide **3d** (110 mg, 0.275 mmol) stannane **19a** (146 mg, 0.440 mmol) and $PdCl_2(PPh_3)_2$ (19.3 mg, 0.0275 mmol, **10 mol%**) using General Procedure F, to afford **4i** as a colorless crystalline solid (69 mg, 0.191 mmol, 69%); **mp** 111 ˚C; **R*f*** 0.53 (petroleum ether / EtOAc, 10:1); **IR** (thin film, ν_{max} / cm^{-1}) 2954, 1350, 1246, 1163, 1090, 901, 841; **^{1}H NMR** (400 MHz, $CDCl_3$) δ_H 7.63 (2H, d, *J* = 8.2 Hz, Ts*H*), 7.25 (2H, d, *J* = 8.2 Hz, Ts*H*), 5.92 (1H, d, *J* = 2.3 Hz, H6), 3.81 (1H, ddd, *J* = 12.5, 8.0, 3.0 Hz, H2), 3.62 (1H, dt, *J* = 13.0, 9.5 Hz, H2), 2.43 (3H, s, Ts*Me*), 2.40-2.29 (1H, m, H5), 1.89 (1H, dd, *J* = 17.5, 8.0 Hz, H4), 1.79-1.65 (2H, m, H3 and H4), 1.56 (1H, dt, *J* = 17.0, 6.0 Hz, H3), 1.08 (3H, d, *J* = 7.0 Hz, H1'), 0.26 (9H, s, TMS); **^{13}C NMR** (101 MHz, $CDCl_3$) δ_C 143.6, 143.5, 139.9, 134.6, 133.0, 129.5, 129.0, 128.3, 50.4, 31.3, 30.3, 30.2, 21.6, 19.8, 0.9; **HRMS** (ES+) calc. for $C_{19}H_{28}NO_2SSi$ $[M+H]^+$ 362.1605, found 362.1597.

4-Methyl-*N*-(3-methylene-5-(trimethylsilyl)pent-4-yn-1-yl)benzenesulfonamide, 21a

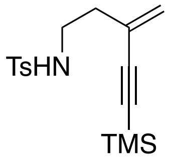

Isolated as a side-product from cascade cyclization reactions of **3d**, as a yellow oil (3 mg, 9.3 μmol, 18%); **R$_f$** 0.23 (petroleum ether / EtOAc, 10:1); **IR** (thin film, ν_{max} / cm^{-1}) 2959, 1599, 1437, 1332, 1250, 1161, 1094, 909; **^{1}H NMR** (500 MHz, $CDCl_3$) δ_H 7.75 (2H, d, *J* = 8.0 Hz, Ts*H*), 7.31 (2H, d, *J* = 8.0 Hz, Ts*H*), 5.43 (1H, d, *J* = 1.0 Hz, =C*H*$_2$), 5.23 (1H, d, *J* = 1.5 Hz, =C*H*$_2$), 4.52 (1H, t, *J* = 6.0 Hz, N*H*), 3.17 (2H, app q, *J* = 6.5 Hz, H1), 2.43 (3H, s, TsC*H*$_3$), 2.28 (2H, t, *J* = 6.5 Hz, H2), 0.14 (9H, s, Si(*CH*$_3$)$_3$); **^{13}C NMR** (125 MHz, $CDCl_3$) δ_c 143.6, 137.2, 129.9, 127.7, 127.3, 125.2, 104.0, 96.0, 41.6, 37.0, 21.7, 0.0; **HRMS** (ES^+) calc. for $C_{16}H_{23}NNaO_2SSi^+$ $[M+Na]^+$ 344.1111, found 344.1110.

4-Methyl-*N*-(3-methylene-5-(triisopropylsilyl)pent-4-ynyl)benzenesulfonamide, 21b

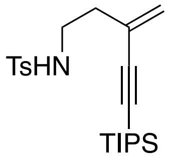

Isolated product from attempted Suzuki cascade cyclisation reactions with TIPS-substituted ynamide **3e**; **R*f*** 0.14 (petroleum ether / ether, 3:1); **^{1}H NMR** (500 MHz, $CDCl_3$) δ_H 7.74 (2H, d, *J* = 8.1 Hz, Ts*H*), 7.30 (2H, d, *J* = 8.1 Hz, Ts*H*), 5.44 (1H, d, *J* = 1.5 Hz, =C*H*$_2$), 5.24 (1H, d, *J* = 1.5 Hz, =C*H*$_2$), 4.47 (1H, t, *J* = 6.2 Hz, N*H*), 3.20 (2H, app q, *J* = 6.4 Hz, H1), 2.43 (3H, s, TsC*H*$_3$), 2.30 (2H, t, *J* = 6.4 Hz, H2), 1.03 (21H, app s, Si*i*-*Pr*$_3$); **^{13}C NMR** (125 MHz, $CDCl_3$) δ_C 143.4, 137.0, 129.7, 127.6, 127.1, 124.8, 105.7, 92.1, 41.3, 37.2, 21.5, 18.5, 11.1; **HRMS** (ES^+) calc. for $C_{22}H_{36}NO_2SSi$ $[M+H]^+$ 406.2231, found 406.2230.

7. Ynamide carbopalladation / Molander-Suzuki coupling / electrocyclization cascades

These were performed using general procedure J.

7-Hexyl-5-phenyl-1-tosyl-2,3,4,5-tetrahydro-1*H*-indole, 4d

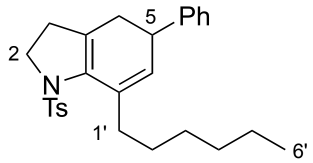

Following General Procedure J, using ynamide **3a** (20.0 mg, 0.0485 mmol), potassium (*E*)-styrenyl trifluoroborate (11.2 mg, 0.0534 mmol), $Pd(PPh_3)_4$ (2.8 mg, 2.4 µmol, **5 mol%**), LiOH (4.7 mg, 0.194 mmol), in MeCN / H_2O (10:1, 0.25 mL), with heating to 85 °C for 2 h. Purification by column chromatography (Et_2O / petroleum ether, 9:1) gave amidodiene **4d** as a pale yellow oil (10.6 mg, 50%). The data for this compound are as previously reported.[16]

7-Hexyl-1-tosyl-2,3,4,5-tetrahydro-1*H*-indole, 4e

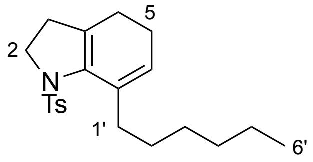

Following General Procedure J, using ynamide **3a** (20.0 mg, 0.0485 mmol), potassium vinyltrifluoroborate (7.2 mg, 0.0534 mmol), $Pd(PPh_3)_4$ (2.8 mg, 2.4 µmol, **5 mol%**), LiOH (4.7 mg, 0.194 mmol) in MeCN / H_2O (10:1, 0.25 mL), with heating to 85 °C for 2 h. Purification by column chromatography (Et_2O / petroleum ether, 9:1) gave the amidodiene **4p** as a pale yellow oil (14.5 mg, 83%). **R_f** 0.44 (petroleum ether / EtOAc, 3:1); **^{1}H NMR** (400 MHz, $CDCl_3$) δ_H 7.67 (2H, d, J = 8.0 Hz, Ts*H*), 7.26 (2H, d, J = 8.0 Hz, Ts*H*), 5.61 (1H, t, J = 4.5 Hz, H6), 3.76 (2H, t, J = 7.5 Hz, H2), 2.56-2.49 (2H, m, H5), 2.44 (3H, s, Ts*Me*), 2.16-2.06 (2H, m, H6), 1.99-1.91 (2H, m, H4), 1.63 (2H, t, J = 7.5 Hz, H3), 1.50-1.39 (2H, m, H1′), 1.45-1.25 (2H, m, H2′); 1.36-1.25 (6H, m, H3′-5′), 0.88 (3H, t, J = 7.0 Hz, H6′). The data for this compound are as previously reported.[16]

7-Hexyl-5-propyl-1-tosyl-2,3,4,5-tetrahydro-1*H*-indole, 4j

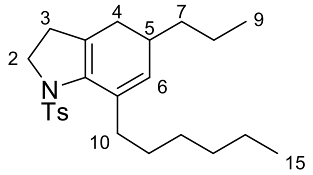

Following General Procedure J, using ynamide **3a** (20.0 mg, 0.0485 mmol), potassium (*E*)-trifluoro(pent-1-en-1-yl)-borate (9.4 mg, 0.0534 mmol), $Pd(PPh_3)_4$ (2.8 mg, 2.4 µmol, **5 mol%**), LiOH (4.7 mg, 0.194 mmol) in MeCN / H_2O (10:1, 0.25 mL), with heating to 85 °C for 2 h. Purification by flash chromatography (Et_2O /

petroleum ether, 9:1) gave the amidodiene **4j** as a pale yellow oil (17.2 mg, 88%); **R*f*** 0.19 (petroleum ether / EtOAc (9:1)); ^{1}**H NMR** (400 MHz, $CDCl_3$) δ_H 7.68 (2H, d, *J* = 7.5 Hz, Ts*H*), 7.26 (2H, d, *J* = 7.5 Hz, Ts*H*), 5.43 (1H, br s, H6), 3.87 (1H, ddd, *J* = 13.0, 8.0 and 3.5, H2), 3.68 (1H, dt, *J* = 13.0, 9.3 Hz, H2′), 2.69-2.58 (1H, m, H10), 2.44 (3H, s, TsCH_3), 2.41-2.22 (2H, m, H10′ and H5), 1.94 (1H, dd, *J* = 17.5 and 8.0, H4), 1.83-1.65 (2H, m, H4′ and H3), 1.63-1.52 (1H, m, H3′), 1.50-1.25 (12H, m, H7, H8, H11-H14), 0.94 (3H, t, *J* = 7.0 Hz, H15) and 0.91-0.85 (3H, m, H9). The data for this compound are as previously reported.[16]

5-Cyclohexyl-7-hexyl-1-tosyl-2,3,4,5-tetrahydro-1*H*-indole, 4k

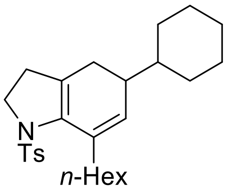

Following General Procedure J, using ynamide **3a** (20.0 mg, 0.0485 mmol), potassium (*E*)-(2-cyclohexylvinyl)trifluoroborate (11.5 mg, 0.0534 mmol), Pd(PPh_3)$_4$ (2.8 mg, 2.4 μmol, **5 mol%**), LiOH (4.7 mg, 0.194 mmol) in MeCN / H_2O (10:1, 0.25 mL), with heating to 85 °C for 2 h. Purification by flash chromatography gave the amidodiene **4k** as a pale yellow oil (17.6 mg, 82%). **R*f*** 0.51 (petroleum ether / Et_2O (7:3)); ^{1}**H NMR** (400 MHz, $CDCl_3$) 7.70-7.66 (2H, m, Ts*H*), 7.30-7.22 (2H, m, Ts*H*), 5.47 (1H, br s, H6), 3.89 (1H, ddd, *J* = 13.0, 7.5, 3.5 Hz, H2), 3.66 (1H, dt, *J* = 13.0, 9.5 Hz, H2′), 2.70-2.60 (1H, m, H14), 2.45 (3H, s, Ts*Me*), 2.40-2.27 (1H, m, H14′), 2.22-2.04 (1H, m, H5), 1.92 (1H, t, *J* = 17.0 Hz, H4), 1.83 (1H, dd, *J* = 17.0, 8.5 Hz, H4′), 1.80-1.62 (4H, m, H3 and H15), 1.52-1.12 (11H, m, alkyl CH_2 and H10), 1.06-0.86 (2H, m, H13), 0.89-0.85 (3H, m, H19); The data for this compound are as previously reported.[16]

5-Cyclopropyl-7-hexyl-1-tosyl-2,3,4,5-tetrahydro-1*H*-indole, 4p

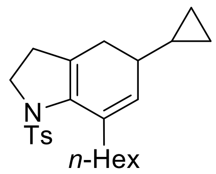

Following General Procedure J, using ynamide **3a** (20.0 mg, 0.0485 mmol), potassium (*E*)-(2-cyclopropylvinyl)trifluoroborate (9.3 mg, 0.0534 mmol), Pd(PPh_3)$_4$ (2.8 mg, 2.4 μmol, **5 mol%**), LiOH (4.7 mg, 0.194 mmol), MeCN:H_2O (10:1) (0.25 mL), heating to 85 °C for 2 h, gave after purification by column chromatography (Et_2O:petroleum ether (9:1)), the amidodiene **4p** as a pale yellow oil (14.5 mg, 75%). **R*f*** 0.55 (petroleum ether / Et_2O (7:3)); ^{1}**H NMR** (400 MHz, $CDCl_3$) δ_H 7.69-7.64 (2H, m, Ts*H*), 7.25-7.22 (2H, m, Ts*H*), 5.56 (1H, br d, *J* = 1.5 Hz, H6), 3.86 (1H, ddd, *J* = 13.0, 8.0 and 3.0, H2), 3.68 (1H, dt, *J* = 13.0, 9.5 Hz, H2′), 2.69-2.58 (1H, m, H12), 2.44 (3H, s, Ts*Me*), 2.41-2.33 (1H, m, H12′), 2.09-1.92 (2H, m, H4), 1.78-1.68 (1H, m, H3), 1.63-1.39 (4H, m, H3′, H5, H13), 1.37-1.25 (6H, m, H14-H16), 0.93-0.86 (3H, m, H17), 0.81-0.72 (1H, m, $H10_a$), 0.55-0.40 (2H, m, $H10_b$), 0.23-0.16 (1H, m, $H10'_a$), 0.11-0.03 (1H, m, $H10'_b$); The data for this compound are as previously reported.[16]

5,7-Diphenyl-1-tosyl-2,3,4,5-tetrahydro-1*H*-indole, 4ae:

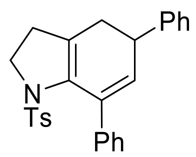

Following General Procedure J, using ynamide **3b** (20.0 mg, 0.0495 mmol), potassium (*E*)-styrenyl trifluoroborate (11.4 mg, 0.0544 mmol), $Pd(PPh_3)_4$ (2.9 mg, 2.5 μmol, **5 mol%**), LiOH (4.7 mg, 0.198 mmol), in MeCN / H_2O (10:1, 0.25 mL), with heating to 85 °C for 2 h. Purification by flash chromatography gave the amidodiene **4ae** as a pale yellow oil (8.5 mg, 40%). **R*f*** 0.40 (petroleum ether / EtOAc (10:1)); **IR** (thin film, ν_{max} / cm^{-1}) 3059, 3028, 2926, 2892, 2845, 2253, 1598, 1493, 1447, 1353, 1164, 1090; **^{1}H NMR** (400 MHz, $CDCl_3$) δ_H 7.62 (2H, d, *J* = 8.0 Hz, Ts*H*), 7.48 (2H, dd, *J* = 8.0, 1.0 Hz, Ph*H*), 7.37-7.34 (6H, m, Ph*H*), 7.31-7.26 (2H, m, Ph*H*), 7.23 (2H, d, *J* = 8.0 Hz, Ts*H*), 6.06 (1H, d, *J* = 3.5 Hz, H6), 4.04-3.99 (1H, m, H2), 3.86-3.80 (1H, dt, *J* = 13.0, 9.0 Hz, H2′), 3.80-3.76 (1H, m, H5), 2.52-2.31 (2H, m, H4), 2.45 (3H, s, Ts*Me*), 1.97 (2H, app t, *J* = 9.0 Hz, H3). The data for this compound are as previously reported.[16]

NB. Compound undergoes partial aerobic oxidation to the indoline.[16]

9-Hexyl-1,4-ditosyl-2,3,4,5,6,7-hexahydro-1*H*-benzo[*e*][1,4]diazepine, 4af

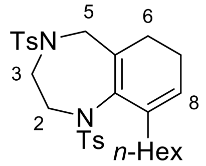

Following General Procedure J, using ynamide **3l** (30.0 mg, 0.0504 mmol), potassium vinyltrifluoroborate (7.4 mg, 0.0554 mmol), $Pd(PPh_3)_4$ (2.9 mg, 2.5 μmol, **5 mol%**), LiOH (4.8 mg, 0.201 mmol), MeCN:H_2O (10:1) (0.25 mL), heating to 85 °C for 3 h, gave after purification by column chromatography, the amidodiene **4af** as a tacky colorless oil (18.4 mg, 80%). **R*f*** 0.40 (petroleum ether / EtOAc (4:1)); **IR** (thin film, ν_{max} / cm^{-1}) 2927, 2857, 1597, 1493, 1451, 1346, 1250, 1211, 1161, 1091, 1044, 962, 877, 815, 670; **^{1}H NMR** (500 MHz, C_6D_6) δ_H 7.66-7.62 (2H, m, Ts*H*), 7.50-7.45 (2H, dm, Ts*H*), 7.37-7.34 (6H, m, Ph*H*), 6.75 (2H, d, *J* = 8.0 Hz, Ts*H*), 6.64 (2H, d, *J* = 8.0 Hz, Ts*H*), 5.60 (1H, br app d, *J* = 6.5 Hz, H8), 4.09 (1H, dt, *J* = 15.0, 3.0 Hz, H5), 3.68 (1H, dd, *J* = 15.0, 1.0 Hz, H3), 3.44 (1H, br d, *J* = 13.0 Hz, H2), 3.07-3.01 (1H, m, H3′), 2.87-2.77 (2H, m, H2′, H5′), 2.51-2.39 (1H, td, *J* = 18.5, 7.5 Hz, H6), 2.38-2.21 (2H, m, $CH_3(CH_2)_4CH_2$), 2.10-1.98 (1H, m, H7), 1.91 (3H, s, Ts*Me*), 1.82 (3H, s, Ts*Me*), 1.85-1.72 (2H, m, H6′, H7′), 1.38-1.22 (8H, m, $(CH_2)_4$), 0.91 (3H, t, *J* = 7.5 Hz, CH_2CH_3); **^{13}C NMR** (125 MHz, $CDCl_3$) δ_C 143.5, 143.2, 139.4, 138.9, 137.7, 137.0, 136.7, 129.9, 129.9, 128.7, 128.1, 127.8, 122.5, 51.5, 49.6, 49.0, 32.6, 32.5, 30.6, 30.0, 29.5, 23.4, 22.8, 21.5, 21.5, 14.7; **HRMS** (ES+) calc. for $C_{29}H_{39}O_4N_2S_2$ $[M+H]^+$ 543.2346, found 543.2347.

NB. Compound readily undergoes oxidation and decomposition.

8. Formal 4-*endo*-trig Heck cyclization of 3l to 8a

9-Hexyl-2-tosyl-2-azabicyclo[5.2.0]nona-1(9),6-diene, 8a

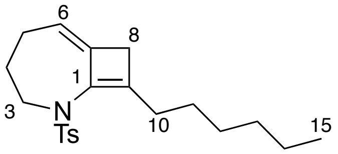

To a solution of ynamide **3k** (30.0 mg, 0.0681 mmol) in PhMe (0.43 mL) was added Et_3N (57 μL, 0.409 mmol) and $Pd(PPh_3)_4$ (3.9 mg, 3.4 μmol, 5 mol%) and the mixture was heated to 85 °C for 16 h. The mixture was then cooled to rt and concentrated, and then purified by flash chromatography (petroleum ether / Et_2O, 9:1) to afford **8a** as a colorless oil (17.6 mg, 72%). $\mathbf{R}_f$ 0.56 (petroleum ether / Et_2O (7:3)); **IR** (thin film, ν_{max} / cm^{-1}) 3029, 2925, 2855, 1624, 1598, 1400, 1370, 1089, 681; ^{1}**H NMR** (500 MHz, $CDCl_3$) δ_H 7.71-7.67 (2H, m, Ts*H*), 7.27 (2H, d, *J* = 8.0 Hz, Ph*H*), 5.51 (1H, t, *J* = 7.5 Hz, H6), 3.61-3.58 (2H, m, H3), 2.63 (2H, br d, *J* = 1.5 Hz, H8), 2.43 (3H, s, TsC*H*3), 2.11-2.07 (2H, m, H10), 2.04 (2H, q, *J* = 7.5 Hz, H5), 1.44-1.36 (6H, m, H4, H11, H12), 1.35-1.28 (4H, m, H13, H14), 0.90 (3H, t, *J* = 7.0 Hz, H15); ^{13}**C NMR** (125 MHz, $CDCl_3$) δ_C 143.4, 138.5, 137.5, 134.7, 134.6, 129.6, 127.3, 114.7, 52.3, 33.2, 31.7, 29.9, 28.6, 28.4, 26.0, 22.7, 21.7, 14.3; **HRMS** (ES+) calc. for $C_{21}H_{30}NO_2S$ $[M+H]^+$ 360.1992, found 360.1897.

9. Synthesis and Reductive Cyclizations of Bromoenynhydrazides 34a-c

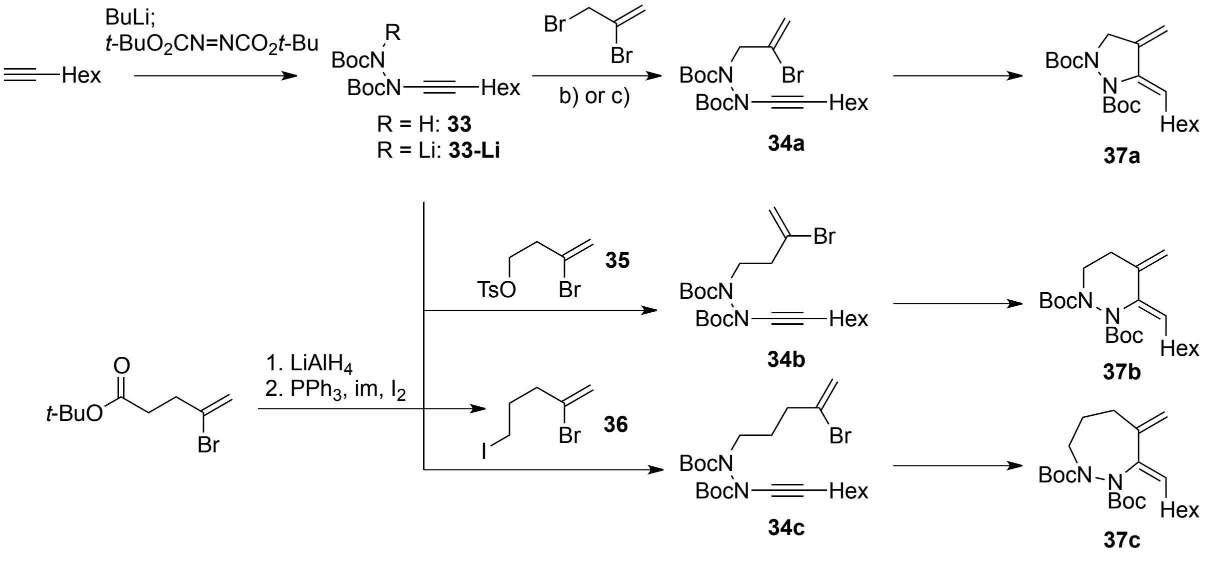

Di-*tert*-butyl 1-(oct-1-yn-1-yl)hydrazine-1,2-dicarboxylate, 31

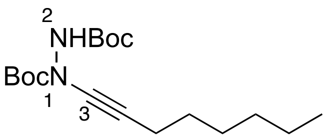

The procedure of Beveridge *et al.* was used for this reaction.[28] *n*-BuLi (1.44 mL, 3.60 mmol, 1.2 equiv., 2.5 M in THF) was added dropwise to a stirred solution of oct-1-yne (0.44 mL, 3.00 mmol, 1.0 equiv.) in THF

(15 mL) at −78 °C over 1-2 min. The mixture was stirred for 15 min at −78 °C, then a solution of di-*tert*-butylazodicarboxylate (1.03 g, 4.50 mmol, 1.5 equiv.) in THF (9 mL) was added dropwise over 1-2 min, after which the cooling bath was removed and the reaction warmed to rt and stirred for a further 30 min. The reaction was quenched by the addition of NH_4Cl (15 mL, sat. aq.) and then diluted with EtOAc and water. The organic layer was separated, dried ($MgSO_4$), filtered through a short silica plug topped with Celite (EtOAc eluent), and concentrated. Purification *via* flash chromatography (petroleum ether / EtOAc, 20:1→10:1) afforded ynhydrazide **31** as a colorless oil (678 mg, 1.98 mmol, 66%); **R_f** 0.34 (petroleum ether / EtOAc, 10:1)); **^{1}H NMR** (500 MHz, $CDCl_3$) δ_H 2.29 (2H, t, J = 7.0 Hz, H5), 1.49 (9H, s, CO_2tBu), 1.48 (9H, s, CO_2tBu), 1.43-1.32 (4H, m, H6 and H7), 1.32-1.22 (4H, m, H8 and H9), 0.88 (3H, t, J = 7.0 Hz, H10); **^{13}C NMR** (125 MHz, $CDCl_3$) δ_C 154.2, 153.4, 83.8, 82.2, 73.4, 71.2, 31.5, 28.9, 28.6, 28.3, 28.1, 22.7, 18.6, 14.2. Data in accordance with literature values.[28]

Di-*tert*-butyl 1-(2-bromoallyl)-2-(oct-1-yn-1-yl)hydrazine-1,2-dicarboxylate, 32a

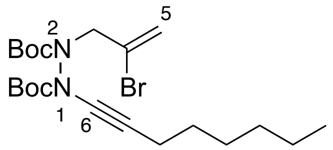

Method A: The procedure of Tšupova *et al.* was used for this preparation.[29] To a vial containing K_2CO_3 (40 mg, 0.293 mmol, 2.0 equiv.), ground NaOH (20 mg, 0.511 mmol, 3.5 equiv.), *n*-Bu_4NHSO_4 (5 mg, 0.0146 mmol, 0.1 equiv.) and *n*-Bu_4NI (5 mg, 0.0146 mmol, 0.1 equiv.) was added a solution of ynhydrazide **31** (50 mg, 0.146 mmol, 1.0 equiv.) and 2,3-dibromopropene (29 µL, 0.293 mmol, 2.0 equiv.) in toluene (0.15 mL, 1 mL $mmol^{-1}$). The resulting mixture was stirred for 2 h at rt before being quenched with NH_4Cl (sat. aq.), diluted with EtOAc and water, and the layers separated. The organic layer was dried ($MgSO_4$) and concentrated. Purification *via* flash chromatography (petroleum ether / EtOAc, 20:1) afforded **32a** as a colorless oil (50 mg, 0.108 mmol, 74%).

Method B: To a vial containing *n*-Bu_4NHSO_4 (5 mg, 0.0146 mmol, 0.1 equiv.) and *n*-Bu_4NI (5 mg, 0.0146 mmol, 0.1 equiv.) was added a solution of ynhydrazide **31** (50 mg, 0.146 mmol, 1.0 equiv.) and 2,3-dibromopropene (29 µL, 0.293 mmol, 2.0 equiv.) in toluene (1.5 mL, 10 mL $mmol^{-1}$). To this was added *aq.* NaOH (1.5 mL, 10 mL $mmol^{-1}$, 25% w/v) and the biphasic mixture was stirred rapidly for 1 h at rt before being quenched with NH_4Cl (sat. aq.), diluted with EtOAc and water, and the layers separated. The organic layer was dried ($MgSO_4$) and concentrated. Purification *via* flash chromatography (petroleum ether / EtOAc, 20:1) afforded **32a** as a colorless oil (48.3 mg, 0.105 mmol, 72%); **R_f** 0.65 (petroleum ether / EtOAc, 10:1); **IR** (thin film) ν_{max} / cm^{-1} 2959, 2933, 1730, 1369, 1301, 1256, 1150; **^{1}H NMR** *Broad signals due to rotameric forms of carbonates* (500 MHz, $CDCl_3$) δ_H 5.98 (1H, br s, H5) and 5.91 (1H, br s, H5), 5.61 (1H, s, H5), 4.65-4.34 (1H, m, H3), 4.26-4.00 (1H, m, H3), 2.31 (2H, br s, H8), 1.63-1.44 (20H, m, H9 and 2 x CO_2tBu), 1.44-1.33 (2H, m, H10), 1.34-1.22 (4H, m, H11 and H12), 0.89 (3H, t, J = 7.0 Hz, H13); **^{13}C NMR** *Broad signals due to rotameric forms of carbonates* (125 MHz, $CDCl_3$) δ_C 154.0, 153.6, 152.9, 152.6, 127.4, 127.0, 120.2, 119.2, 83.8, 82.4, 72.3, 56.8, 56.5, 31.5, 28.9, 28.6, 28.2, 28.0, 22.7, 18.5, 14.2; **HRMS** (ES^+)

calc. for $C_{21}H_{35}BrN_2NaO_4$ [M+Na]$^+$ 481.1672, found 481.1661.

3-Bromobut-3-en-1-yl 4-methylbenzenesulfonate, 33 (prepared as S2, above)

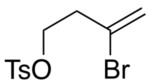

To an ice-cooled solution of alcohol **10a** (5.11 g, 33.8 mmol, 1.0 equiv.) in CH_2Cl_2 (2.6 mL) was added pyridine (5.5 mL, 67.7 mmol, 2.0 equiv.) and *p*-toluenesulfonyl chloride (9.68 g, 50.8 mmol, 1.5 equiv.). After addition, the reaction was warmed to rt and stirred for 3 h before being diluted with CH_2Cl_2. The organic layer was separated and washed with HCl (1M, aq.). The aqueous layer was extracted with CH_2Cl_2, and the combined organics washed with $NaHCO_3$ (sat., aq.), dried ($MgSO_4$) and concentrated. Purification *via* flash chromatography (petroleum ether / Et_2O, 8:1) afforded tosylate **S2** as a colorless oil (10.3 g, 33.8 mmol, quant.); $\mathbf{R_f}$ 0.21 (petroleum ether / Et_2O, 8:1); **^{1}H NMR** (400 MHz, $CDCl_3$) δ_H 7.79 (1H, d, *J* = 8.0 Hz, Ar*H*), 7.35 (2H, d, *J* = 8.0 Hz, Ar*H*), 5.65 (1H, app s, H4), 5.48 (1H, d, *J* = 2.0 Hz, H4), 4.19 (2H, t, *J* = 6.2 Hz, H1), 2.74 (2H, t, *J* = 6.2 Hz, H2), 2.45 (3H, s, TsCH_3); **^{13}C NMR** (101 MHz, $CDCl_3$) δ_C 145.0, 132.8, 129.9, 128.0, 127.6, 120.2, 67.2, 40.7, 21.7. Data in accordance with literature values.[11]

Di-*tert*-butyl 1-(3-bromobut-3-en-1-yl)-2-(oct-1-yn-1-yl)hydrazine-1,2-dicarboxylate, 32b

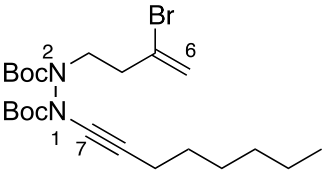

The procedure of Tšupova *et al.* was used for this preparation.[29] To a vial containing K_2CO_3 (40 mg, 0.293 mmol, 2.0 equiv.), ground NaOH (20 mg, 0.511 mmol, 3.5 equiv.), Bu_4NHSO_4 (5 mg, 0.0146 mmol, 0.1 equiv.) and *n*-Bu_4NI (5 mg, 0.0146 mmol, 0.1 equiv.) was added a solution of ynhydrazide **31** (50 mg, 0.146 mmol, 1.0 equiv.) and tosylate **33** (89 mg, 0.293 mmol, 2.0 equiv.) in toluene (0.15 mL, 1 mL mmol^{-1}). The resulting mixture was stirred for 22 h at rt before being quenched with NH_4Cl (sat. aq.), diluted with EtOAc and water, and the layers separated. The organic layer was dried ($MgSO_4$) and concentrated. Purification *via* flash chromatography (petroleum ether / EtOAc, 20:1) afforded **32b** as a colorless oil (38 mg, 0.080 mmol, 55%); $\mathbf{R_f}$ 0.54 (petroleum ether / EtOAc, 10:1); **IR** (thin film) ν_{max} / cm^{-1} 2979, 2932, 1719, 1368, 1300, 1254, 1147; **^{1}H NMR** *Broad signals due to rotameric forms of both carbonates* (500 MHz, $CDCl_3$) δ_H 5.68 (1H, s, H6), 5.46 (1H, s, H6), 3.81 (1H, br s, H3), 3.61 (1H, br s, H3), 2.76 (2H, br d, *J* = 6.0 Hz, H4), 2.30 (2H, br s, H9), 1.58-1.33 (22H, m, H10, H11 and 2 x CO_2tBu), 1.28 (4H, br s, H12 and H13), 0.88 (3H, t, *J* = 6.5 Hz, H14); **^{13}C NMR** *Broad and multiple signals due to rotameric forms of both carbonates* (125 MHz, $CDCl_3$) δ_C 154.0, 153.7, 153.1, 152.8, 130.7, 130.4, 118.9, 118.4, 83.7, 81.9, 73.1, 72.8, 71.6, 48.7, 47.2, 40.2, 39.4, 31.5, 28.9, 28.6, 28.5, 28.3, 28.1, 22.7, 18.6, 14.2; **HRMS** (ES$^+$) calc. for $C_{22}H_{37}BrN_2NaO_4$ [M+Na]$^+$ 495.1829; found 495.1820.

The preparation of iodide **34** was achieved according to the following Scheme:[30]

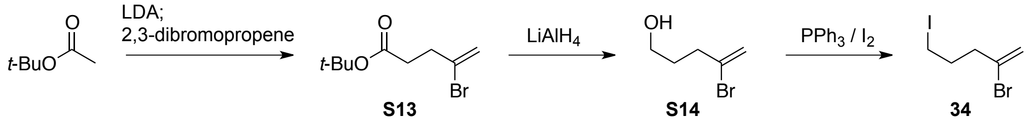

***tert*-Butyl-4-bromopent-4-enoate, S13**

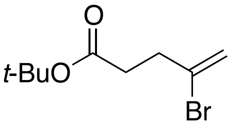

The procedure of Baldwin *et al.* was used.[30] To a freshly prepared 1 M solution of LDA at −78 °C (17.2 mL, 17.2 mmol, 1.5 equiv., prepared from addition of *n*-BuLi (8.0 mL, 2.5 M) to a solution of *i*-Pr_2NH (2.8 mL) in THF (9.2 mL) at 0 °C), was added a solution of *tert*-butyl acetate (2.30 mL, 17.2 mmol, 1.5 equiv.) in THF (36 mL) dropwise. The reaction was stirred for 30 min at −78 °C, then 2,3-dibromopropene (1.2 mL, 11.5 mmol, 1.0 equiv.) was added dropwise. After stirring for 2 h −78 °C, the reaction was quenched with NH_4Cl (sat. aq.) and allowed to warm to rt, before being extracted with Et_2O. The organic layers were combined, dried ($MgSO_4$) and concentrated. Purification *via* flash chromatography (petroleum ether / EtOAc, 10:1) afforded **S13** as a colorless oil (2.15 g, 9.20 mmol, 80%); **R_f** 0.54 (petroleum ether / EtOAc, 10:1); **^{1}H NMR** (500 MHz, $CDCl_3$) δ_H 5.63 (1H, d, *J* = 1.5 Hz, H5), 5.43 (1H, d, *J* = 1.5 Hz, H5), 2.72 (2H, t, *J* = 7.5 Hz, H2), 2.49 (2H, td, *J* = 7.5 Hz, H3), 1.45 (9H, s, C(CH_3)$_3$); **^{13}C NMR** (101 MHz, $CDCl_3$) δ_C 171.2, 132.5, 117.3, 80.7, 36.8, 34.1, 28.1. Data in accordance with literature values.[30]

4-Bromopent-4-en-1-ol, S14

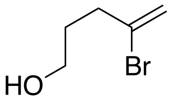

The procedure of Baldwin *et al.* was used.[30] To a solution of $LiAlH_4$ (382 mg, 10.07 mmol, 1.1 equiv.) in Et_2O (18 mL) at 0 °C was added a solution of *tert*-butyl 4-bromopent-4-enoate **S13** (2.15 g, 9.15 mmol, 1.0 equiv.) in Et_2O (18 mL). The reaction mixture was then stirred for 2 h at rt before being quenched with NaOH (sat. *aq*.). The aqueous layer was extracted with Et_2O and the combined organic layers were dried ($MgSO_4$) and concentrated *in vacuo* to afford **S14** as a pale yellow oil (0.85 g, 5.12 mmol, 56%); **R_f** 0.58 (petroleum ether / EtOAc, 1:1); **^{1}H NMR** (500 MHz, $CDCl_3$) δ_H 5.62 (1H, d, *J* = 1.5 Hz, H5), 5.43 (1H, d, *J* = 1.5 Hz, H5), 3.69 (2H, t, *J* = 6.0 Hz, H1), 2.55 (2H, t, *J* = 7.5 Hz, H3), 1.84 (2H, quin, *J* = 7.5 Hz, H2), 1.39 (1H, br s, O*H*); **^{13}C NMR** (125 MHz, $CDCl_3$) δ_C 133.9, 117.0, 61.4, 37.7, 30.8. Data in accordance with literature values.[30]

2-Bromo-5-iodopent-1-ene, 34

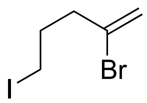

The procedure of Baldwin *et al.* was used for this reaction.[30] To a solution of alcohol **S14** (1.00 g, 6.06 mmol, 1.0 equiv.) in Et_2O:MeCN (38 mL, 3:1) was added PPh_3 (1.75 g, 6.66 mmol, 1.1 equiv.) and imidazole (453 mg, 6.66 mmol, 1.1 equiv.). The resultant solution was cooled to 0 °C, then iodine (1.69 g, 6.66 mmol, 1.1 equiv.) was added portionwise. After complete addition the reaction was warmed to rt and stirred for 1 h, before being filtered under reduced pressure, the solid being thoroughly washed with cold Et_2O, then the solvent was removed *in vacuo*. The crude mixture was redissolved in Et_2O and washed successively with 10% *aq.* $Na_2S_2O_3$ and brine before being dried ($MgSO_4$) and concentrated. Petroleum ether was then added to the crude residue and the reaction was filtered through a silica plug; concentration of the filtrate afforded iodide **34** as a colorless oil (1.29 g, 4.72 mmol, 78%); **R*f*** 0.70 (petroleum ether / EtOAc, 10:1); **^{1}H NMR** (400 MHz, $CDCl_3$) δ_H 5.68 (1H, d, J = 1.0 Hz, H1), 5.47 (1H, d, J = 1.5 Hz, H1), 3.19 (2H, t, J = 6.5 Hz, H5), 2.56 (2H, t, J = 7.0 Hz, H3), 2.06 (2H, quin, J = 7.0 Hz, H4); **^{13}C NMR** (101 MHz, $CDCl_3$) δ_C 132.2, 118.4, 41.7, 31.0, 5.2. Data in accordance with literature values.[30]

Di-*tert*-butyl 1-(4-bromopent-4-en-1-yl)-2-(oct-1-yn-1-yl)hydrazine-1,2-dicarboxylate, 32c

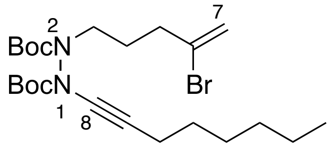

To a vial containing *n*-Bu_4NHSO_4 (5.0 mg, 0.0146 mmol, 0.1 equiv.) was added a solution of ynhydrazide **31** (50 mg, 0.146 mmol, 1.0 equiv.) and iodide **34** (80 mg, 0.293 mmol, 2.0 equiv.) in toluene (1.5 mL, 10 mL mmol^{-1}). To this was added NaOH (aq., 1.5 mL, 10 mL mmol^{-1}, 25% w/v) and the biphasic mixture was stirred vigorously at rt for 21 h, before being quenched with NH_4Cl (sat. aq.), diluted with EtOAc and water, and the layers separated. The organic layer was dried ($MgSO_4$) and concentrated. Purification *via* flash chromatography (petroleum ether / EtOAc, 10:1) afforded bromoenynhydrazide **32c** as a colorless oil (41.2 mg, 0.84 mmol, 58%); **R*f*** 0.49 (petroleum ether / EtOAc, 10:1); **IR** (thin film) ν_{max} / cm^{-1} 2980, 2933, 1740, 1716, 1393, 1303, 1264, 1148; **^{1}H NMR** *Broad signals due to rotameric forms of both carbonates* (500 MHz, $CDCl_3$) δ_H 5.60 (1H, d, J = 1.5 Hz, H7), 5.42 (1H, br s, H7), 3.67-3.49 (1H, m, H3), 3.49-3.34 (1H, m, H3), 2.51 (2H, t, J = 6.5 Hz, H5), 2.28 (2H, t, J = 10.5 Hz, H10), 1.86 (2H, dt, J = 13.5, 7.0 Hz, H4), 1.57-1.33 (22H, m, H11, H12 and 2 x CO_2t-Bu), 1.33-1.07 (4H, m, H13 and H14), 0.87 (3H, t, J = 6.5 Hz, H15); **^{13}C NMR** *Broad and multiple signals due to rotameric forms of both carbonates* (125 MHz, $CDCl_3$) δ_C 154.4, 154.1, 153.3, 152.8, 133.9, 133.6, 117.1, 83.6, 83.4, 81.7, 81.6, 73.1, 72.7, 71.6, 46.9, 38.8, 31.5, 29.0, 28.6, 28.5, 28.3, 28.1, 25.9, 22.7, 18.6, 14.2; **HRMS** (ES+) calc. for $C_{23}H_{39}BrN_2NaO_4$ [M+Na]$^+$ 509.1985, found 509.1978.

(*Z*)-Di-*tert*-butyl 3-heptylidene-4-methylenepyrazolidine-1,2-dicarboxylate, 35a

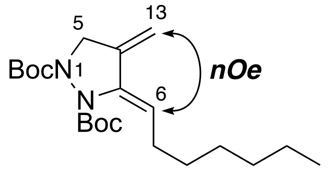

Prepared by General Procedure H using bromoenynamide **32a** (90 mg, 0.195 mmol) with heating for 1 h or by General Procedure I Method B using bromoenynamide **32a** (90 mg, 0.195 mmol) with heating for 22 h. Purification *via* column chromatography (petroleum ether / EtOAc, 20:1) afforded the exocyclic diene **35a** as a pale yellow oil (36.5 mg, 0.096 mmol, 49% from Procedure H, and 39.5 mg, 0.103 mmol, 53% from Procedure IB); $\mathbf{R}_f$ 0.44 (petroleum ether / EtOAc, 10:1); **IR** (thin film) ν_{max} / cm^{-1} 2978, 2929, 1712, 1456, 1392, 1368, 1326, 1151; **^{1}H NMR** (500 MHz, $CDCl_3$) δ_H 5.64-5.57 (1H, m, H6), 5.30 (1H, t, *J* = 2.5 Hz, H13), 4.90 (1H, s, H13), 4.51 (1H, d, *J* = 15.0 Hz, H5), 3.87 (1H, d, *J* = 15.0 Hz, H5), 2.26-2.18 (2H, m, H7), 1.56-1.33 (22H, m, H8, H9 and 2 × CO_2tBu), 1.35-1.09 (4H, m, H10 and H11), 0.86 (3H, t, *J* = 7.0 Hz, H12); **^{13}C NMR** (125 MHz, $CDCl_3$) δ_C 156.0, 154.3, 141.5, 134.5, 115.9, 102.5, 82.0, 81.3, 50.7, 31.8, 29.5, 29.2, 29.1, 28.3, 28.3, 22.7, 14.2; **HRMS** (ES^+) calc. for $C_{21}H_{36}N_2NaO_4$ $[M+Na]^+$ 403.2567; found 403.2567. The stereochemistry of **35a** was assigned through ^{1}H NMR nOe experiments. A strong enhancement was seen between H6 and *cis*-H13. The stereochemistry of the dienes **35b** and **35c** was assigned by analogy.

(*Z*)-Di-*tert*-butyl 3-heptylidene-4-methylenetetrahydropyridazine-1,2-dicarboxylate, 35b

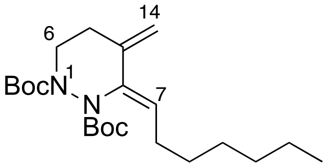

Prepared by General Procedure I Method B using bromoenynamide **32b** (10 mg, 0.021 mmol) with heating for 30 min. Purification *via* column chromatography (petroleum ether → petroleum ether / EtOAc, 9:1) afforded the exocyclic diene **35b** as a colorless oil (4.3 mg, 0.011 mmol, 52%); $\mathbf{R}_f$ 0.43 (petroleum ether / EtOAc, 10:1); **IR** (thin film) ν_{max} / cm^{-1} 2929, 1710, 1456, 1393, 1368, 1255, 1151; **^{1}H NMR** *Broad signals due to rotameric forms of carbonates* (500 MHz, $CDCl_3$) δ_H 5.72 (1H, s, H7), 5.22 (1H, s, H14), 4.83 (1H, s, H14), 3.99-3.85 (1H, m, H6), 3.45-3.29 (1H, m, H6), 2.52-2.41 (1H, m, H5), 2.34-2.18 (2H, m, H5 and H8), 2.16-2.06 (1H, m, H8), 1.46 (18H, s, 2 x CO_2t-Bu), 1.36-1.22 (8H, m, H9-H12), 0.87 (3H, t, *J* = 7.0 Hz, H13); **^{13}C NMR** *Broad signals due to rotameric forms of carbonates* (125 MHz, $CDCl_3$) δ_C 154.5, 154.0, 133.2, 128.8, 123.5, 110.1, 96.3, 81.5, 80.7, 44.5, 32.0, 30.8, 29.9, 29.4, 29.1, 28.5, 28.3, 28.1, 22.8, 14.2; **HRMS** (ES^+) calc. for $C_{22}H_{38}N_2NaO_4$ $[M+Na]^+$ 417.2724; found 417.2706.

(*Z*)-Di-*tert*-butyl 3-heptylidene-4-methylene-1,2-diazepane-1,2-dicarboxylate, 35c

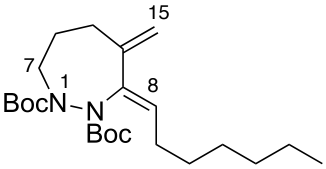

Prepared by General Procedure H using bromoenynamide **32c** (40 mg, 0.082 mmol) with heating for 1.5 h. Purification *via* column chromatography (petroleum ether → petroleum ether / EtOAc, 9:1) afforded the exocyclic diene **35c** as a colorless oil (5.2 mg, 0.012 mmol, 15%); **R*f*** 0.43 (petroleum ether / EtOAc, 10:1); **IR** (thin film) ν_{max} / cm^{-1} 2930, 1715, 1455, 1392, 1366, 1323, 1152; **^{1}H NMR** *Broad / doubled signals due to rotameric forms of carbonates* (500 MHz, $CDCl_3$) δ_H 5.64 (1H, dd, *J* = 8.5, 5.5 Hz, H8), 5.22 (1H, s, H15), 4.84 (1H, s, H15), 4.23 (1H, d, *J* = 14.0 Hz, H7), 2.96-2.83 (1H, m, H7), 2.49-2.40 (1H, m, H5), 2.15-1.98 (3H, m, H5 and H9), 1.85-1.76 (1H, m, H6), 1.71-1.59 (1H, m, H6), 1.51-1.42 (20H, m, 2 × CO_2*t*-Bu and H10), 1.37-1.22 (6H, m, H11-H13), 0.92-0.84 (3H, m, H14); **^{13}C NMR** *Broad signals due to rotameric forms of carbonates* (125MHz, $CDCl_3$) δ_C 155.3, 151.4, 147.4, 137.4, 123.0, 113.1, 81.3, 81.0, 47.9, 34.3, 32.0, 30.8, 29.4, 29.0, 28.9, 28.5, 28.4, 22.8, 14.2; **HRMS** (ES^+) calc. for $C_{23}H_{40}N_2NaO_4$ $[M+Na]^+$ 431.2880; found 431.2875.

10. (Hetero-) Diels-Alder Cycloadditions of Amidodienes 5a, 4c and 4d

Diisopropyl 7-hexyl-1-tosyl-2,3-dihydro-1*H*-pyrrolo[2,3-*d*]pyridazine-5,6(4*H*,7*H*)-dicarboxylate, 36a

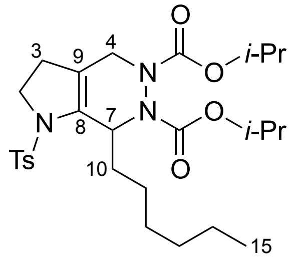

Di-*iso*-propylazodicarboxylate (19.5 µL, 0.099 mmol, 1.1 equiv.) was added dropwise to a stirred solution of diene **5a** (30 mg, 0.090 mmol, 1.0 equiv.) in CH_2Cl_2 (1.0 mL). The reaction mixture was stirred for 3 h at rt, then diluted with CH_2Cl_2 (10 mL) and washed with $NaHCO_3$ (10 mL, sat., aq.). The organic layer was dried ($MgSO_4$), filtered and concentrated. The resulting crude material was purified by flash chromatography (petroleum ether / EtOAc, 5:1) to give **36a** as a colorless oil (36 mg, 0.067 mmol, 75%); $\mathbf{R}_f$ 0.41 (petroleum ether / EtOAc, 3:1); **IR** (thin film) ν_{max} / cm^{-1} 2928, 1720, 1381, 1168, 1105; **^{1}H NMR** (500 MHz, d_8-toluene, 363 K) δ_H 7.80 (2H d, J = 7.7 Hz, Ts*H*), 6.95 (2H, d, J = 7.7 Hz, *m*-TsH), 5.76-5.62 (1H, br m, H7), 5.07-4.94 (2H, m, 2 x C*H*(CH_3)$_2$), 4.28 (1H, d, J = 16.5 Hz, H4), 3.53 (1H, d, J = 16.5 Hz, H4), 3.51-3.40 (2H, m, H2), 2.21 (1H, m, H10), 2.00 (3H, s, ArC*H*$_3$), 1.84-1.71 (2H, m, H11) 1.70-1.59 (2H, m, H10, H3), 1.60-1.49 (1H, m, H3), 1.43-1.25 (6H, m, H12-14), 1.25-1.05 (12H, m, 2 x CH(C*H*$_3$)$_2$), 0.99-0.83 (3H, m, H15); **^{13}C NMR** (125 MHz, d_8-toluene, 363 K) δ_C 156.5, 155.5, 143.7, 139.1, 135.8, 130.0, 128.8, 118.6, 70.6, 70.0, 56.5, 49.9, 43.8, 32.8, 32.2, 30.5, 29.5, 29.4, 27.6, 23.3, 22.5, 22.4, 22.3, 22.2, 21.3, 14.4; HRMS *m/z* (ES^+) calc. for $C_{27}H_{41}N_3NaO_6S$ $[M+Na]^+$ 558.2608; found 558.2602.

Di-*tert*-butyl 7-hexyl-1-tosyl-2,3-dihydro-1*H*-pyrrolo[2,3-*d*]pyridazine-5,6(4*H*,7*H*)-dicarboxylate, 36b

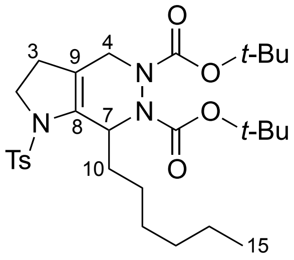

Di-*tert*-butylazodicarboxylate (23 mg, 0.099 mmol, 1.1 equiv.) was added dropwise to a solution of **5a** (30 mg, 0.090 mmol, 1.0 equiv.) in CH_2Cl_2 (1.0 mL). The reaction mixture was stirred at rt for 15 h, then diluted with CH_2Cl_2 (10 mL) and washed with $NaHCO_3$ (10 mL, sat. aq.). The organic layer was dried ($MgSO_4$), filtered and concentrated. The resulting crude material was purified by column chromatography (10%→20% EtOAc in petroleum ether) to give **36b** as a colorless oil (48 mg, 0.085 mmol, 95%); the ^{1}H NMR spectrum of this compound is broadened due to the highly rotameric nature of the product. Due to the thermal instability of the compound, variable temperature analysis could not be attempted, and as such ^{13}C NMR spectroscopic analysis is not reported as signal coalescence could not be obtained. $\mathbf{R}_f$ 0.38 (petroleum ether /

EtOAc, 4:1); **^{1}H NMR** (500 MHz, $CDCl_3$) δ_H 7.83-7.62 (2H, br m, Ts*H*), 7.32-7.20 (2H, br m, Ts*H*), 5.44-5.19 (1H, br m, H9), 4.25 (1H, d, *J* = 16.7 Hz, H4), 3.83-3.74 (1H, m, H2), 3.68-3.48 (2H, br m, H2, H4), 2.46-2.37 (3H, br m, ArC*H*$_3$), 2.15-2.05 (2H, m, H3), 2.00-1.90 (1H, m, H10), 1.68-1.22 (9H, m, H10, H12-14), 0.93-0.83 (3H, br m, H15); **IR** (thin film) ν_{max} / cm^{-1} 2928, 1703, 1393, 1367, 1164; HRMS *m/z* (ES$^+$) calc. for $C_{29}H_{45}N_3NaO_6S$ $[M+Na]^+$ 586.2921; found 586.2930.

10-Hexyl-7-phenyl-1-tosyl-2,3,7,10-tetrahydropyrrolo[2,3-*d*][1,2,4]triazolo[1,2-*a*]pyridazine-6,8(1*H*,4*H*)-dione, 36c.

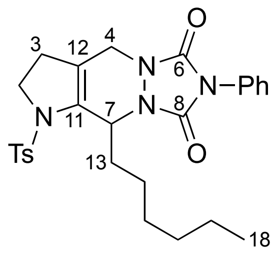

To a solution of **5a** (30 mg, 0.090 mmol, 1.0 equiv.) in CH_2Cl_2 (0.5 mL) was added a solution of 4-phenyl-3*H*-1,2,4-triazole-3,5(4*H*)-dione **6c** (18 mg, 0.099 mmol, 1.1 equiv.) in CH_2Cl_2 (0.5 mL). The reaction mixture was stirred at rt for 10 min. The reaction mixture was added to CH_2Cl_2 (10 mL), washed with sat. aq. $NaHCO_3$ (10 mL), dried ($MgSO_4$), filtered and concentrated *in vacuo*. The resulting crude material was purified by column chromatography (petroleum ether / EtOAc, 3:2) to give **36c** as a colorless oil (39 mg, 0.077 mmol, 85%); **R*f*** 0.29 (3:2, petroleum ether:EtOAc); **^{1}H NMR** (500 MHz, $CDCl_3$) δ_H 7.69 (2H, d, *J* = 8.2 Hz, Ts*H*), 7.57-7.53 (2H, m, Ph*H*), 7.49 (2H, app t, *J* = 7.5 Hz, Ph*H*), 7.39 (1H, t, *J* = 7.5 Hz, Ph*H*), 7.35 (2H, d, *J* = 8.2 Hz, Ts*H*), 5.47 (1H, br s, H10), 4.25 (1H, dd, *J* = 16.1, 0.6 Hz, H4), 3.98-3.81 (3H, m, H4, 2 x H2), 2.45 (3H, s, ArC*H*$_3$), 2.29-2.20 (1H, m, H3), 2.20-2.09 (2H, m, H3, H13), 2.06-1.96 (1H, m, H13), 1.48-1.21 (8H, m, H14-17), 0.87 (3H, t, *J* = 6.7 Hz, H18); **^{13}C NMR** (125 MHz, $CDCl_3$) δ_C 153.0, 150.6, 144.5, 136.0, 133.2, 131.2, 130.0, 129.2, 128.2, 127.6, 125.3, 118.6, 54.2, 50.2, 44.3, 32.1, 31.6, 29.2, 29.2, 24.9, 22.6, 21.6, 14.0; **IR** (thin film) ν_{max} / cm^{-1} 2927, 1777, 1715, 1354, 1164; **HRMS** *m/z* (ES+) calc. for $C_{27}H_{32}N_4NaO_4S$ $[M+Na]^+$ 531.2036; found 531.2049.

***N*,*N*,*N*',*N*'-Tetrabenzylmethanediamine**

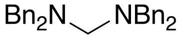

According to the modified procedure of Heaney et al.[31] Dibenzylamine (500 mg, 97%, 2.46 mmol, 2.0 equiv.) was added dropwise to a mixture of formaldehyde (94 µL, 37 wt% in H_2O, 1.23 mmol, 1.0 equiv.) in H_2O (1.5 mL) and Et_2O (0.5 mL) at 0 °C. The reaction mixture was stirred at rt overnight and then saturated with solid KOH. The aqueous layer was extracted with Et_2O (2 × 15 mL). The combined organic layers were dried ($MgSO_4$), filtered and concentrated *in vacuo*. The resulting crude material was purified by column chromatography (5:1, petroleum ether:EtOAc) to give the title compound as a colorless solid (385 mg, 0.95 mmol, 77%); m.p. 94-98 °C; R*f* = 0.50 (5:1, petroleum ether:EtOAc); δ_H (400 MHz, $CDCl_3$) 7.17-7.34 (20H, m, Ar*H*), 3.60 (8H, C*H*$_2$Ph), 3.08 (2H, N-C*H*$_2$-N); δ_C (100 MHz, $CDCl_3$) 139.7, 129.0, 128.1,

126.7, 72.3, 56.1. Data consistent with literature values.[32]

5,5-Dibenzyl-7-hexyl-1-tosyl-2,3,4,5,6,7-hexahydro-1*H*-pyrrolo[3,2-*c*]pyridin-5-ium iodide, 39d.

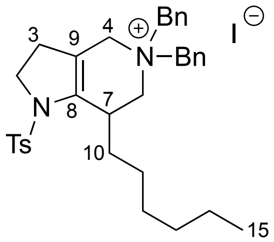

A solution of *N,N,N',N'*-tetrabenzylmethanediamine (200 mg, 0.49 mmol) in Et_2O (0.5 mL) was added dropwise to a solution of iodotrimethylsilane (73 μL, 0.49 mmol) in Et_2O (0.5 mL) at 0 °C. The reaction was warmed to rt and stirred for 20 min, and the resultant precipitate was collected by filtration, washed with Et_2O, and dried *in vacuo* to give *N*-benzyl-*N*-methylene-1-phenylmethanaminium iodide **6d** (135 mg, 0.40 mmol, 82%).

To a solution of **5a** (30 mg, 0.090 mmol, 1.0 equiv.) in CH_2Cl_2 (1.0 mL) was added *N*-benzyl-*N*-methylene-1-phenylmethanaminium iodide **6d** (33 mg, 0.099 mmol, 1.1 equiv.). The reaction mixture was stirred for 18 h at rt, and then concentrated. The resulting crude material was purified by flash chromatography (4.5% MeOH in CH_2Cl_2) to give **36d** as a yellow semi-solid (46 mg, 0.069 mmol, 76%); **R*f*** 0.53 (5.0% MeOH in DCM); **IR** (thin film) ν_{max} / cm^{-1} 2927, 1352, 1162; **^{1}H NMR** (500 MHz, $CDCl_3$) δ_H 7.81 (2H, d, *J* = 8.2 Hz, Ts*H*), 7.60 (2H, d, *J* = 6.9 Hz, Ph*H*), 7.58-7.52 (1H, m, Ph*H*), 7.51-7.40 (5H, m, ArH), 7.29-7.23 (4H, m, Ts*H*, ArH), 5.48 (1H, d, *J* = 12.9 Hz, C*H*H'Ph), 5.32 (1H, d, *J* = 12.6 Hz, CH*H*'Ph), 4.62-4.49 (3H, m, C*H*H'Ph, CH*H*'Ph, H6), 4.25 (1H, ddd, *J* = 12.9, 8.8, 2.2 Hz, H2), 3.90-3.71 (3H, m, H4, H2), 3.37 (1H, br s, H7), 2.76 (1H, dd, *J* = 12.2, 11.4 Hz, H6), 2.38-2.14 (3H, m, H3, H10), 2.28 (3H, s, ArC*H*$_3$) 1.49-1.39 (1H, m, H10), 1.37-1.14 (8H, m, H11-14), 0.89 (3H, t, *J* = 6.9 Hz, H15); **^{13}C NMR** (125 MHz, $CDCl_3$) δ_C 145.3, 137.6, 134.6, 133.8, 133.1, 131.1, 131.0, 130.6, 129.5, 129.4, 127.3, 126.4, 125.9, 118.4, 64.2, 60.3, 58.4, 52.2, 50.3, 31.8, 31.3, 30.6, 29.7, 29.0, 25.3, 22.5, 21.5, 14.0; **HRMS** *m/z* (ESI) calc. for $C_{34}H_{43}N_2O_2S$ $[M]^+$ 543.3040; found 543.3033.

(7-Hexyl-1-tosyl-2,3-dihydropyrrolo[2,3-*d*][1,2]oxazin-6(1*H*,4*H*,7*H*)-yl)(phenyl)methanone, 36e

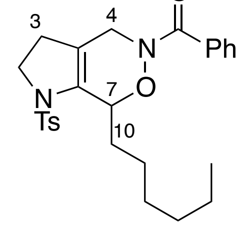

To a solution of **5a** (30 mg, 0.090 mmol, 1.0 equiv.) in CH_2Cl_2 (1.0 mL) was added Bu_4NIO_4 (90 mg, 0.207 mmol, 2.3 equiv.). Benzohydroxamic acid (27 mg, 0.198 mmol, 2.2 equiv.) was then added portionwise over 1 h. After consumption of starting material as judged by tlc, the reaction mixture was then partitioned between CH_2Cl_2 (5 mL) and $NaHCO_3$ (10 mL, sat. aq.). The aqueous layer was extracted with

further CH_2Cl_2 (2 x 5 mL). The combined organic layers were dried ($MgSO_4$), filtered and concentrated. The resulting crude material was purified by flash chromatography (30%→40% EtOAc / petroleum ether) to give **36e** as a yellow oil (as a single regioisomer) (40 mg, 0.085 mmol, 95%); **R*f*** 0.50 (petroleum ether / EtOAc, 3:2); **IR** (thin film) ν_{max} / cm^{-1} 2928, 1642, 1354, 1164; **^{1}H NMR** (500 MHz, $CDCl_3$) δ_H 7.78 (2H, d, *J* = 6.9 Hz, Ph*H*), 7.60 (2H, d, *J* = 8.2 Hz, Ts*H*), 7.54-7.48 (1H, m, Ph*H*), 7.28-7.52 (2H, m, Ph*H*), 7.36 (2H, d, *J* = 8.2 Hz, Ts*H*), 4.79 (1H, br d, *J* = 6.9 Hz, H7), 4.45 (1H, d, *J* = 17.2 Hz, H4), 4.16 (1H, d, *J* = 17.2 Hz, H4), 3.82-3.67 (2H, m, H2), 2.47 (3H, s, ArCH_3), 2.09 (1H, dd, *J* = 15.4, 9.5 Hz, H3), 2.04-1.97 (1H, m, H10), 1.85-1.74 (1H, m, H3), 1.58-1.50 (1H, m, H10), 1.27-1.05 (8H, m, H11-14), 0.85 (3H, t, *J* = 7.3 Hz, H15); **^{13}C NMR** (100 MHz, $CDCl_3$) δ_C 170.2, 144.5, 136.9, 133.3, 132.5, 131.2, 129.9, 128.8, 128.0, 127.6, 123.9, 80.8 (C7), 50.0 (C4), 43.4, 31.7, 31.1, 29.4, 28.8, 24.9, 22.5, 21.7, 14.1; **HRMS** *m/z* (ES^+) calc. for $C_{26}H_{32}N_2NaO_4S$ $[M+Na]^+$ 491.1975; found 491.1974.

Atom	δ (**36e**)
H4	4.45, 4.16 (CH_2N)
H7	4.79 (CHO)
C4	50.0
C7	80.8

Note: An HSQC spectrum was used to correlate these proton and carbon chemical shifts and thus assign regioselectivity.

***tert*-Butyl 7-hexyl-1-tosyl-2,3,4,7-tetrahydropyrrolo[3,2-*d*][1,2]oxazine-5(1*H*)-carboxylate 36f, and *tert*-Butyl 7-hexyl-1-tosyl-2,3,4,7-tetrahydropyrrolo[2,3-*d*][1,2]oxazine-6(1*H*)-carboxylate, 37f**

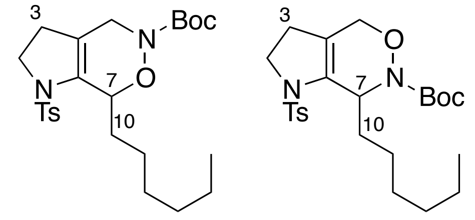

36f **37f**

To a solution of **5a** (30 mg, 0.090 mmol, 1.0 equiv.) in CH_2Cl_2 (1.0 mL) was added Bu_4NIO_4 (90 mg, 0.207 mmol, 2.3 equiv.). *N*-Boc-hydroxylamine (26 mg, 0.198 mmol, 2.2 equiv.) was then added portionwise over 1 h. After completion of the reaction as judged by tlc, the reaction mixture was partitioned between CH_2Cl_2 (5 mL) and $NaHCO_3$ (10 mL, sat. aq.). The aqueous layer was extracted with further CH_2Cl_2 (2 x 5 mL). The combined organic layers were dried ($MgSO_4$), filtered and concentrated. The resulting crude material was purified by flash chromatography (15%→25% EtOAc / petroleum ether) to give regioisomeric products **36f** and **37f**, in a 1.8:1 ratio, as a pale yellow oil (41 mg, 0.088 mmol, 98%); For the purposes of characterization, 3 mg of each regioisomer was separated by semi-preparative HPLC.

Product **36f**: **HPLC** (10% *i*-PrOH in hexane, 6.0 mL/min, t_R = 3.27 min); **R*f*** 0.73 (petroleum ether / EtOAc,

5:1); **IR** (thin film) ν_{max} / cm^{-1} 2928, 1702, 1355, 1164; **^{1}H NMR** (500 MHz, $CDCl_3$) δ_H 7.61 (2H, d, *J* = 8.2 Hz, Ts*H*), 7.30 (2H, d, *J* = 8.2 Hz, Ts*H*), 4.99 (1H, br d, *J* = 8.8 Hz, H7), 4.00 (1H, d, *J* = 17.0 Hz, H4), 3.94 (1H, d, *J* = 17.0 Hz, H4), 3.76-3.70 (2H, m, H2), 2.44 (3H, s, ArCH_3), 2.12-2.02 (2H, m, H10, H3), 1.91-1.79 (1H, H3), 1.78-1.68 (1H, m, H10), 1.68-1.59 (1H, m, H11) 1.55 (9H, s, *t*-Bu), 1.55-1.46 (1H, m, H11), 1.44-1.23 (6H, m H12-14), 0.89 (3H, t, *J* = 6.9 Hz, H15); **^{13}C NMR** (125 MHz, $CDCl_3$) δ_C 155.0, 144.3, 137.6, 132.8, 129.7, 127.6, 122.5, 81.8, 79.1 (C7), 50.0, 45.1 (C4), 31.8, 31.2, 29.3, 29.0, 28.3, 25.4, 22.7, 21.6, 14.1; **HRMS** *m/z* (ES^+) calc. for $C_{24}H_{36}N_2NaO_5S$ $[M+Na]^+$ 487.2237; found 487.2236.

Product **37f: HPLC** (10% *i*-PrOH in hexane, 6.0 mL/min, t_R = 4.10 min); **R_f** 0.62 (petroleum ether / EtOAc, 5:1); **IR** (thin film) ν_{max} / cm^{-1} 2927, 1704, 1364, 1165; **^{1}H NMR** (500 MHz, $CDCl_3$) δ_H 7.72 (2H, d, *J* = 8.2 Hz, Ts*H*), 7.28 (2H, d, *J* = 8.2 Hz, Ts*H*), 5.21 (1H, br d, *J* = 9.1 Hz, H7), 4.53 (1H, d, *J* = 14.8 Hz, H4), 4.12 (1H, d, *J* = 14.8 Hz, H4), 3.82 (1H, ddd, *J* = 12.0, 10.4, 9.1 Hz, H2), 3.63 (1H, ddd, *J* = 12.0, 10.1, 5.0 Hz, H2), 2.43 (3H, s, ArCH_3), 2.18-2.10 (1H, m, H3), 2.09-1.96 (2H, m, H3, H10), 1.78-1.67 (1H, m, H10), 1.56 (9H, s, *t*-Bu), 1.52-1.45 (2H, m, H11), 1.44-1.27 (6H, m, H12-14), 0.88 (3H, t, *J* = 6.7 Hz, H15); **^{13}C NMR** (125 MHz, $CDCl_3$) δ_C 155.0, 143.9, 136.7, 133.1, 129.7, 127.8, 119.3, 81.8, 67.6 (C4), 55.4 (C7), 49.3, 31.8, 31.3, 28.7, 28.6, 28.4, 26.3, 22.6, 21.6, 14.2; **HRMS** *m/z* (ES^+) calc. for $C_{24}H_{36}N_2NaO_5S$ $[M+Na]^+$ 487.2237; found 487.2232.

Assignment of regioselectivity was made on the basis of ^{1}H and ^{13}C NMR chemical shifts, which show increased deshielding at the position adjacent to the oxygen atom. An HSQC spectrum was also used to correlate proton and carbon chemical shifts and thus assign regioselectivity.

Atom	δ /ppm (**36f**)	δ /ppm (**37f**)
H4	4.00 (CH_2N)	4.53, 4.12 (CH_2O)
H7	4.99 (CHO)	5.21 (CHN)
C4	45.1 (CN)	67.6 (CO)
C7	79.1 (CO)	55.4 (CN)

7-Hexyl-5-phenyl-1-tosyl-1,2,3,4,5,7-hexahydropyrrolo[3,2-*d*][1,2]oxazine, 36g, and 7-Hexyl-6-phenyl-1-tosyl-1,2,3,4,6,7-hexahydropyrrolo[2,3-*d*][1,2]oxazine, 37g

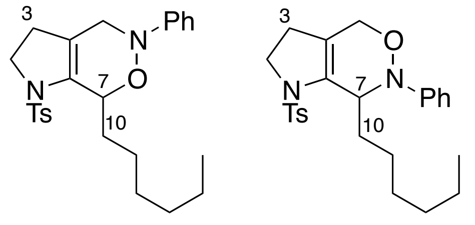

36g **37g**

To a solution of **5a** (30 mg, 0.090 mmol, 1.0 equiv.) in CH_2Cl_2 (1.0 mL) was added nitrosobenzene (11 mg, 0.099 mmol, 1.1 equiv.). The reaction mixture was stirred for 10 min at rt. The reaction mixture was added

to CH_2Cl_2 (10 mL), washed with sat. aq. $NaHCO_3$ (10 mL), dried ($MgSO_4$), filtered and concentrated *in vacuo*. The resulting crude material was purified by column chromatography (5:1, petroleum ether:EtOAc) to give regioisomeric products **36g** and **37g** in a 3.7:1 ratio as a yellow oil (32 mg, 0.073 mmol, 81%); For the purposes of characterisation, 2 mg of each regioisomer was separated by semi-preparative HPLC.

Product **36g: HPLC** (1.5% *i*-PrOH in hexane, 6.5 mL/min, t_R = 5.68 min); $\mathbf{R_f}$ 0.31 (petroleum ether / EtOAc, 5:1); **IR** (thin film) ν_{max} / cm^{-1} 2926, 1354, 1163; **^{1}H NMR** (700 MHz, $CDCl_3$) δ_H 7.67 (2H, d, *J* = 8.1 Hz, Ts*H*), 7.32 (2H, dd, *J* = 8.3, 7.4 Hz Ph*H*), 7.29 (2H, d, *J* = 8.1 Hz, Ts*H*), 7.09 (2H, d, *J* = 8.3 Hz, Ph*H*), 7.00 (1H, t, *J* = 7.4 Hz, Ph*H*), 5.05 (1H, br d, *J* = 8.5 Hz, H7), 3.84-3.73 (3H, m, 2 x H2, H4), 3.65 (1H, d, *J* = 15.0 Hz, H4), 2.43 (3H, s, ArC*H*$_3$), 2.20-2.09 (2H, m, H3, H10), 1.98-1.90 (1H, m, H3), 1.90-1.82 (1H, m, H10), 1.44-1.19 (8H, m, H11-14), 0.91-0.87 (3H, m, H15); **^{13}C NMR** (125 MHz, $CDCl_3$) δ_C 150.2, 144.1, 139.3, 133.2, 129.8, 129.1, 128.0, 122.2, 115.6, 78.5 (C7), 51.8 (C4), 50.3, 32.0, 31.6, 29.7, 29.5, 29.3, 22.8, 21.8, 14.3; **HRMS** *m/z* (ES^+) calc. for $C_{25}H_{32}N_2NaO_3S$ $[M+Na]^+$ 463.2026; found 463.2034.

Product **37g**: **HPLC** (1.5% *i*-PrOH in hexane, 6.5 mL/min, t_R = 5.30 min); $\mathbf{R_f}$ 0.27 (petroleum ether / EtOAc, 5:1); **IR** (thin film) ν_{max} / cm^{-1} 2925, 1353, 1163; **^{1}H NMR** (700 MHz, $CDCl_3$) δ_H 7.70 (2H, d, *J* = 8.3 Hz, Ts*H*), 7.31 (2H, dd, *J* = 8.4, 7.5 Hz, Ph*H*), 7.24 (2H, d, *J* = 8.3 Hz, Ts*H*), 7.09 (2H, d, *J* = 8.4 Hz, Ph*H*), 6.97 (1H, t, *J* = 7.5 Hz, Ph*H*), 4.84 (1H, br s, H7), 4.43 (1H, d, *J* = 15.2 Hz, H4), 4.16 (1H, d, *J* = 15.2 Hz, H4), 3.84-3.71 (2H, m, H2), 2.41 (3H, s, ArC*H*$_3$), 2.12-2.05 (1H, m, H3), 1.97-1.90 (1H, m, H3), 1.90-1.83 (2H, m, H10), 1.51-1.42 (2H, m, H11), 1.32-1.18 (6H, m, H12-14), 0.92-0.79 (3H, m, H15); **^{13}C NMR** (125 MHz, $CDCl_3$) δ_C 148.1, 138.0, 134.2, 129.8, 129.2, 127.8, 121.5, 115.9, 65.0 (C4), 58.4 (C7), 50.0, 31.9, 31.3, 28.7, 26.7, 26.0, 22.8, 21.8, 14.2; **HRMS** *m/z* (ES^+) calc. for $C_{25}H_{32}N_2NaO_3S$ $[M+Na]^+$ 463.2026; found: 463.2030.

The assignment of regioselectivity was made on the basis of ^{1}H and ^{13}C NMR chemical shifts, as follows, which show increased deshielding at the position adjacent to the oxygen atom:

Atom	δ /ppm (**36g**)	δ /ppm (**37g**)
H4	3.80, 3.65 (CH_2N)	4.43, 4.16 (CH_2O)
H7	5.05 (CHO)	4.84 (CHN)
C4	51.6 (CN)	65.0 (CO)
C7	78.3 (CO)	58.4 (CN)

3a,8-(8'-Methyl)ethano-(4a*R*,7a*S*)-5,6-dichloro-9-hexyl-1-tosyl-2,3,3a,4a,7a,8-hexahydro-1*H*-benzoquino[*e*]indole-4a,7a-dicarbonitrile, 38

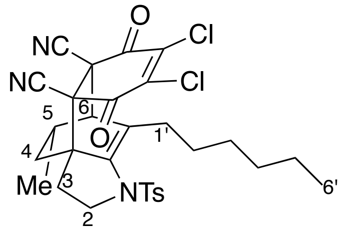

To a stirred solution of dienamide **4c** (80 mg, 0.214 mmol, 1.0 equiv.) in toluene (3.0 mL) at rt was added DDQ (58 mg, 0.256 mmol, 1.2 equiv.). After 2 h, the reaction was quenched with $NaHCO_3$ (5 mL, sat., aq.) and extracted with Et_2O. The combined organic layers were dried ($MgSO_4$) and concentrated. Flash chromatography (petroleum ether / EtOAc, 10:1) afforded **38** as an orange solid (80 mg, 0.133 mmol, 62%); $\mathbf{R}_f$ 0.44 (petroleum ether / EtOAc, 3:1); **IR** (thin film) ν_{max} / cm $^{-1}$ 2930, 1704, 1579, 1454, 1359, 1244, 1168; **^{1}H NMR** (500 MHz, $CDCl_3$) δ_H 7.67 (2H, d, *J* = 8.3 Hz, ArH), 7.33 (2H, d, *J* = 8.0 Hz, ArH), 3.88 (1H, ddd, *J* = 13.4, 8.4, 2.1 Hz, H2), 3.20 (1H, d, *J* = 1.3 Hz, H6), 3.02 (1H, ddd, *J* = 13.4, 11.0, 6.5 Hz, H2), 2.65-2.60 (3H, m, H1', H4 and H5), 2.46 (3H, s, TsMe), 2.07 (1H, ddd, *J* = 14.4, 6.5, 2.1 Hz, H3), 1.50 (1H, ddd, *J* = 12.3, 3.8 Hz, H1'), 1.52-1.25 (9H, m, H3, H2'-H5'), 1.00 (3H, d, *J* = 6.3 Hz, Me), 0.90 (t, *J* = 6.9 Hz, H6'), 0.53 (app q, *J* = 8.9 Hz, H4); **^{13}C NMR** (125 MHz, $CDCl_3$) δ_C 178.9, 178.0, 147.2, 145.8, 145.2, 136.4, 135.5, 130.1, 127.4, 123.9, 115.2, 114.2, 58.2, 56.1, 55.5, 54.9, 51.1, 41.1, 33.0, 31.5, 29.7, 29.5, 29.4, 27.5, 22.6, 21.7, 21.4, 14.1; **HRMS** (ES^+) calc. for $C_{31}H_{35}Cl_2N_3NaO_5S$ $[M+MeOH+Na]^+$654.1574; found 654.1567. For X-ray crystallography data, see end of SI.

Of note: The anisotropic effects of the nitriles lead to remarkable deshielding at H4β and H5 (2.6 ppm); and shielding at H4α (0.5 ppm).

7-Hexyl-5,8-diphenyl-1-tosyl-1,2,3,4,5,6-hexahydro-3a,6-(epoxyimino)indole, 39

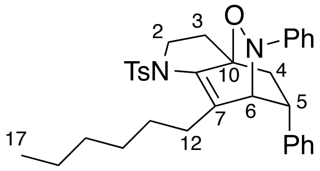

To a solution of **4d** (76 mg, 0.174 mmol, 1.0 equiv.) in CH_2Cl_2 (2.0 mL) was added nitrosobenzene (21 mg, 0.192 mmol, 1.1 equiv.). The reaction mixture was stirred at rt for 18 h, then diluted with CH_2Cl_2 (10 mL) and washed with $NaHCO_3$ (10 mL, sat. aq.). The organic layer was dried ($MgSO_4$), filtered and concentrated. The resulting crude material was purified by column chromatography (petroleum ether / EtOAc, 5:1) to give **39** as a yellow oil (71 mg, 0.131 mmol, 75%); $\mathbf{R}_f$ 0.28 (petroleum ether / EtOAc, 5:1); **IR** (thin film) ν_{max} / cm^{-1} 2927, 1597, 1488, 1355, 1165; **^{1}H NMR** (500 MHz, $CDCl_3$) δ_H 7.72 (2H, d, *J* = 8.2 Hz, Ts*H*), 7.38-7.32 (2H, m, Ar*H*) 7.29-7.17 (7H, m, Ar*H*), 6.97-6.93 (3H, m, Ar*H*), 4.31 (1H, d, *J* = 2.8 Hz, H6), 4.08 (1H, ddd, *J* = 12.3, 7.9, 2.2 Hz, H2), 3.84 (1H, ddd, *J* = 12.3, 10.7, 6.7 Hz, H2), 3.78 (1H, ddd, *J* = 9.4, 5.3, 2.9 Hz, H5), 2.51 (1H, dd, *J* = 13.0, 9.6 Hz, H4), 2.41 (3H, s, ArC*H*$_3$), 2.06 (1H, ddd, *J* = 14.0, 6.8, 2.2 Hz, H3),

1.93 (1H, ddd, J = 14.1, 11.9, 5.0 Hz, H12), 1.76 (1H, ddd, J = 14.1, 11.9, 5.0 Hz, H12), 1.71 (1H, ddd, J = 14.0, 10.9, 7.9 Hz, H3), 1.34 (1H, dd, J = 13.0, 5.4 Hz, H4), 1.06 (2H, app quin, J = 7.3 Hz, H16), 0.96-0.82 (4H, m, H14, H15), 0.77 (3H, t, J = 7.4 Hz, H17), 0.60-0.42 (2H, m, H13); δ_C (125 MHz, $CDCl_3$) 151.1, 144.1, 142.1, 136.3, 135.7, 129.8, 128.5, 128.4, 128.1, 127.5, 126.9, 125.6, 122.7, 117.8, 81.1 (C10), 69.5 (C6), 51.4 (C2), 42.8 (C5), 37.8 (C4), 32.4 (C12), 31.9 (C3), 31.4, 29.4, 25.7, 22.3, 21.6, 14.0; HRMS *m/z* (ES^+) calc. for $C_{33}H_{38}N_2NaO_3S$ $[M+Na]^+$ 565.2495; found 565.2490.

The regiochemical assignment of this cycloadduct is made, as previously, on the basis of ^1H and ^{13}C NMR chemical shift analysis:

Atom	δ /ppm (**39**)
H6	4.31 (CHN)
C6	69.5 (CN)
C10	81.1 (CO)

7-Hexyl-5-phenyl-6-(phenylamino)-1-tosyl-2,3,3a,4,5,6-hexahydro-1*H*-indol-3a-ol, 41.

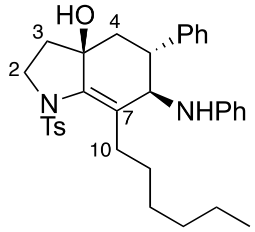

To a solution of **39** (24 mg, 0.044 mmol, 1.0 equiv.) in MeCN / H_2O (0.7 mL / 0.1 mL) was added $Mo(CO)_6$ (17 mg, 0.066 mmol, 1.6 equiv.). The reaction mixture was stirred for 10 min at rt, then $NaBH_4$ (5.0 mg, 0.13 mmol, 3.0 equiv.) added. The reaction mixture was heated to 90 °C for 20 h, then cooled to rt and filtered through Celite, eluting with CH_2Cl_2. The solution was dried ($MgSO_4$), filtered and concentrated. The resulting crude material was purified by column chromatography (petroleum ether / EtOAc, 5:1) to give **41** as a yellow oil (14.0 mg, 0.026 mmol, 58%); $\mathbf{R_f}$ 0.27 (petroleum ether / EtOAc, 5:1); ^{1}**H NMR** (500 MHz, $CDCl_3$) δ_H 7.87 (2H, d, J = 8.2 Hz, Ts*H*), 7.37 (2H, d, J = 8.2 Hz, Ts*H*), 7.28-7.22 (2H, m, Ph*H*), 7.22-7.17 (1H, m, Ph*H*), 7.15 (2H, d, J = 7.3 Hz, Ph*H*), 7.06 (2H, app t, J = 7.9 Hz, NPh*H*), 6.65 (1H, t, J = 7.3 Hz, NPh*H*), 6.48 (2H, d, J = 7.9 Hz, NPh*H*), 3.91 (1H, d, J = 9.7 Hz, H6), 3.80 (1H, td, J = 10.1, 7.9 Hz, H2), 3.64 (1H, br s, NH), 3.44 (1H, td, J = 9.9, 1.5 Hz, H2), 3.35 (1H, ddd, J = 13.1, 10.0, 2.9 Hz, H5), 2.72 (1H, ddd, J = 14.5, 9.2, 7.1 Hz, H10), 2.45 (3H, s, ArC*H*$_3$), 2.23-2.17 (1H, m, H10), 2.18 (dd, J = 13.3, 2.7 Hz, H4) 2.12 (1H, ddd, J = 13.0, 7.8, 1.7 Hz, H3), 2.03 (1H, br s, OH), 1.87 (1H, app t, J = 13.4 Hz, H4), 1.72 (1H, dt, J = 13.0, 9.6 Hz, H3), 1.46-1.04 (8H, m, H11-14), 0.83 (3H, t, J = 6.9 Hz, H15); ^{13}**C NMR** (125 MHz, $CDCl_3$) δ_C 147.5, 144.3, 143.2, 136.5, 135.5, 135.0, 129.7, 129.0, 128.4, 128.2, 127.9, 126.7, 117.6, 114.4, 74.7 (C-OH), 60.9 (C6), 47.6 (C2), 42.1 (C5), 40.4 (C4), 38.0 (C3), 31.5, 29.6, 28.8, 27.4, 22.6, 21.6, 14.1; **IR** (thin film) ν_{max} / cm^{-1} 3504, 3396, 2926, 1601, 1497, 1325, 1159; **HRMS** *m/z* (ES^+) calc. for

$C_{33}H_{40}N_2NaO_3S$ $[M+Na]^+$ 567.2652; found 567.2652.

The assignment of regio- and stereochemistry of the cycloaddition is supported by this compound; ^1H–^1H NMR J values imply the following conformation due to large axial-axial coupling around H4-H6:

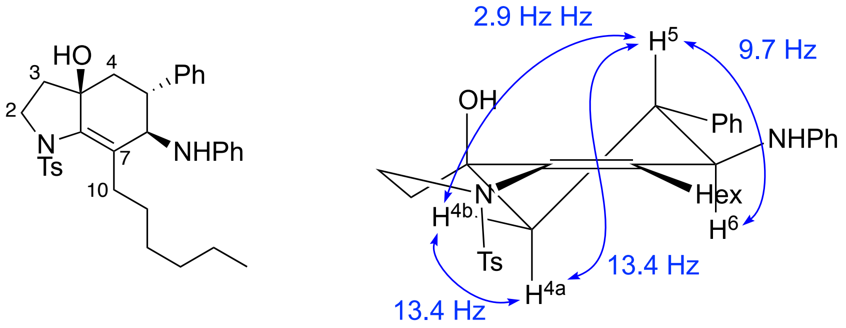

***N*-(2-((1*S*,3*R*)-5-Hexyl-4-oxo-3-(((4-oxocyclohexa-2,5-dien-1-ylidene)amino)oxy)-1,2,3,4-tetrahydro-[1,1'-biphenyl]-3-yl)ethyl)-4-methylbenzenesulfonamide, 43**

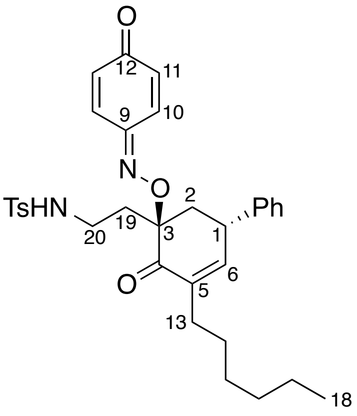

According to the procedure of Miller and co-workers.[33] To a solution of **41** (35 mg, 0.064 mmol, 1.0 equiv.) in EtOAc / MeCN (1 mL, 1:1) was added a solution of $NaIO_4$ (62 mg, 0.290 mmol, 4.5 equiv.) in H_2O (0.5 mL), followed by $RuCl_3$ (0.5 mg, 0.0016 mmol, 0.025 equiv.). The reaction mixture was stirred at rt, open to air, for 2 h. The mixture was then partitioned between EtOAc (5 mL) and brine (5 mL). The aqueous layer was extracted with EtOAc (3 x 5 mL), then the combined organic extracts were dried ($MgSO_4$), filtered and concentrated. The resulting crude material was purified by flash chromatography (petroleum ether / EtOAc, 4:1) to give **43** as a yellow oil (7.0 mg, 0.012 mmol, 19%); $\mathbf{R}_f$ 0.25 (petroleum ether / EtOAc, 4:1); **^{1}H NMR** (500 MHz, $CDCl_3$) δ_H 7.69 (2H, d, J = 8.2 Hz, Ts*H*), 7.61 (1H, dd, J = 10.4, 2.5 Hz, H10), 7.40-7.34 (2H, m, Ph*H*), 7.33-7.28 (1H, m, Ph*H*), 7.26 (2H, d, J = 8.2 Hz, Ts*H*), 7.17 (2H, d, J = 7.3 Hz, Ph*H*), 7.10 (1H, dd, J = 10.1, 2.5 Hz, H10), 6.62 (1H, br s, H6), 6.54-6.47 (2H, m, H11), 4.72 (1H, t, J = 6.0 Hz, NH), 3.87-3.80 (1H, m, H1), 3.07-3.00 (2H, m, H20), 2.75 (1H, ddd, J = 14.7, 4.9, 1.6 Hz, H2), 2.43-2.35 (1H, m, H13), 2.40 (3H, s, ArC*H*$_3$), 2.20-2.11 (3H, m, H19, H13), 2.09-2.02 (1H, m, H2), 1.42-1.19 (8H, m, H14-17), 0.88-0.82 (3H, m, H18); **^{13}C NMR** (125 MHz, $CDCl_3$) δ_C 194.8 (C4), 187.1 (C12), 150.0 (C9), 147.3 (C6), 143.6, 143.0, 138.4 (C5), 136.9, 136.8, 133.0, 130.7, 129.8, 129.2, 127.68, 127.51, 127.2, 124.1, 85.3 (C3), 41.6 (C2), 39.9 (C1), 38.3 (C20), 33.7 (C19), 31.8, 30.2, 29.1, 28.4, 22.8, 21.7, 14.2; **IR** (thin film) ν_{max} / cm^{-1} 3279, 2925, 1685, 1641, 1329, 1159, 1094, 988; **HRMS** *m/z* (ES^+) calc. for $C_{33}H_{38}N_2NaO_5S$ $[M+Na]^+$ 597.2394; found 597.2401.

11. References

[1] T. Mandai, J. Nokami, T. Yano, Y. Yoshinaga, J. Otera, *J. Org. Chem.* **1984**, *49*, 172.

[2] H. Lu, C. Li, *Org. Lett.* **2006**, *8*, 5365.

[3] X. Zhang, Y. Zhang, J. Huang, R. P. Hsung, K. C. M. Kurtz, J. Oppenheimer, M. E. Petersen, I. K. Sagamanova, L. Shen, M. R. Tracey, *J. Org. Chem.* **2006**, *71*, 4170.

[4] J. R. Johnson, W. L. McEwen, *Org. Synth.* **1941**, *Coll. Vol. 1*, 521.

[5] G. K. Dewkar, S. V. Narina, A. Sudalai, *Org. Lett.* **2003**, *5*, 4501.

[6] R. Lespieau, M. Bourgel, *Org. Synth.* **1941**, *Coll. Vol. 1*, 209.

[7] T. Mandai, J. Nokami, T. Yano, Y. Yoshinaga, J. Otera, *J. Org. Chem.* **1984**, *49*, 172.

[8] P. Magnus, D. Quagliato, *J. Org. Chem.* **1985**, *50*, 1621.

[9] T. Kauffmann, D. Stach, *Chem. Ber.* **1992**, *125*, 913.

[10] D. Beruben, I. Marek, J. F. Normant, N. Platzer, *J. Org. Chem.* **1995**, *60*, 2488.

[11] J. Pillai, *D. Phil. Thesis, University of Oxford*, **1998**.

[12] A. Padwa, A. G. Waterson, *Tetrahedron* **2000**, *56*, 10159.

[13] W. J. Moran, J. P. Morken, *Org. Lett.* **2006**, *8*, 2413.

[14] H. Lu, Q. Chen, C. Li, *J. Org. Chem.* **2007**, *72*, 2564.

[15] K. R. Law, C. S. P. McErlean, *Chem. Eur. J.* **2013**, *19*, 15852.

[16] R. L. Greenaway, C. D. Campbell, O. T. Holton, C. A. Russell, E. A. Anderson, *Chem. Eur. J.* **2011**, *17*, 14366.

[17] Y. Fukudome, H. Naito, T. Hata, H. Urabe, *J. Am. Chem. Soc.* **2008**, *130*, 1820.

[18] D. L. Usanov, H. Yamamoto, *J. Am. Chem. Soc.* **2011**, *133*, 1286.

[19] G. Sabitha, C. S. Reddy, P. Srihari, J. S. Yadav, *Synthesis* **2003**, *2003*, 2699.

[20] X. Zhang, Y. Zhang, J. Huang, R. P. Hsung, K. C. M. Kurtz, J. Oppenheimer, M. E. Petersen, I. K. Sagamanova, L. Shen, M. R. Tracey, *J. Org. Chem.* **2006**, *71*, 4170.

[21] M. X.-W. Jiang, M. Rawat, W. D. Wulff, *J. Am. Chem. Soc.* **2004**, *126*, 5970.

[22] S. Roy, G. W. Gribble, *Synth. Commun.* **2007**, *37*, 829.

[23] R. L. Greenaway, C. D. Campbell, H. A. Chapman, E. A. Anderson, *Adv. Synth. Catal.* **2012**, *354*, 3187.

[24] For pioneering work using alkynyliodonium salts, see: B. Witulski, T. Stengel, *Angew. Chem.* **1998**, *110*, 495; *Angew. Chem. Int. Ed.* **1998**, *37*, 489.

[25] M. G. Moloney, J. T. Pinhey, M. J. Stoermer, *J. Chem. Soc., Perkin Trans. 1* **1990**, 2645.

[26] D. Seyferth, F. G. A. Stone, *J. Am. Chem. Soc.* **1957**, *79*, 515.

[27] J. C. Conway, P. Quayle, A. C. Regan, C. J. Urch, *Tetrahedron* **2005**, *61*, 11910.

[28] R. E. Beveridge, R. A. Batey, *Org. Lett.* **2012**, *14*, 540.

[29] S. Tšupova, O. Lebedev, U. Mäeorg, *Tetrahedron* **2012**, *68*, 1011.

[30] J. E. Baldwin, R. M. Adlington, R. Singh, *Tetrahedron* **1992**, *48*, 3385.

[31] H. Heaney, G. Papageorgiou, R. F. Wilkins, *Tetrahedron* **1997**, *53*, 2941.

[32] N. Millot, C. Piazza, S. Avolio, P. Knochel, *Synthesis* **2000**, *2000*, 941.

[33] For an example of oxidative alkene cleavage of a nitroso-Diels-Alder cycloadduct, see: B. T. Shireman, M. J. Miller, M. Jonas, O. Wiest, *J. Org. Chem.* **2001**, *66*, 6046.

12. Copies of ^{1}H and ^{13}C NMR spectra for novel or key compounds.

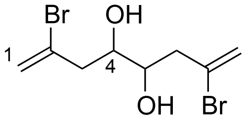

S1

^{1}H: 500 MHz, $CDCl_3$

^{13}C: 125 MHz, $CDCl_3$

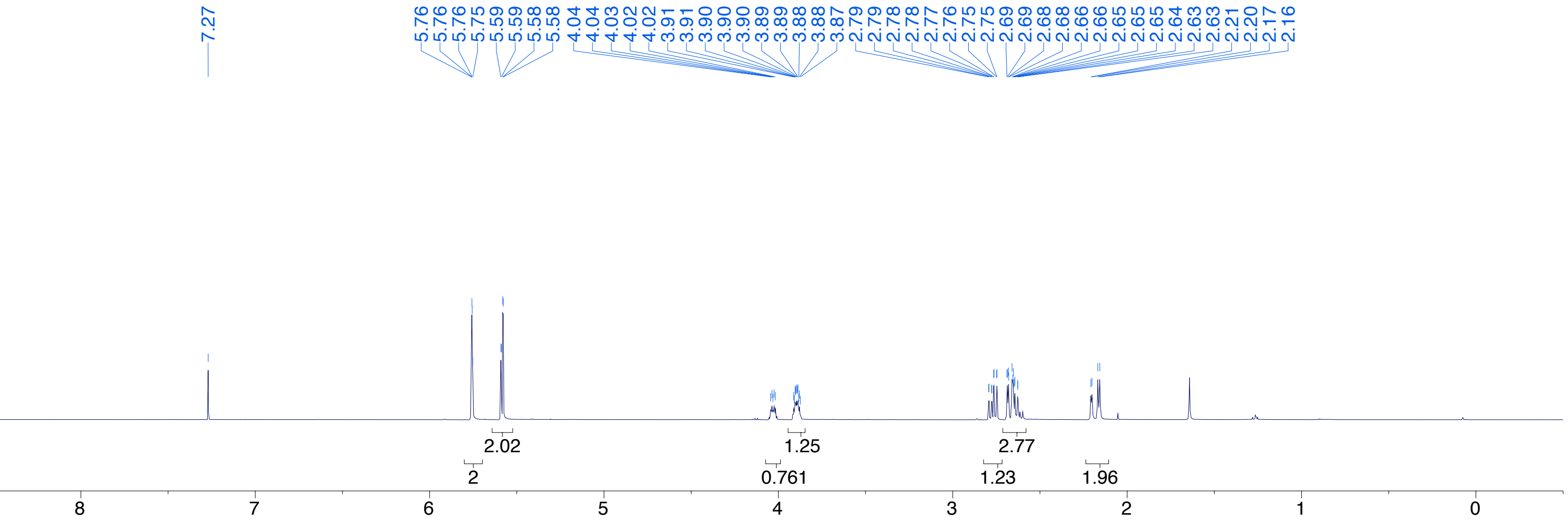

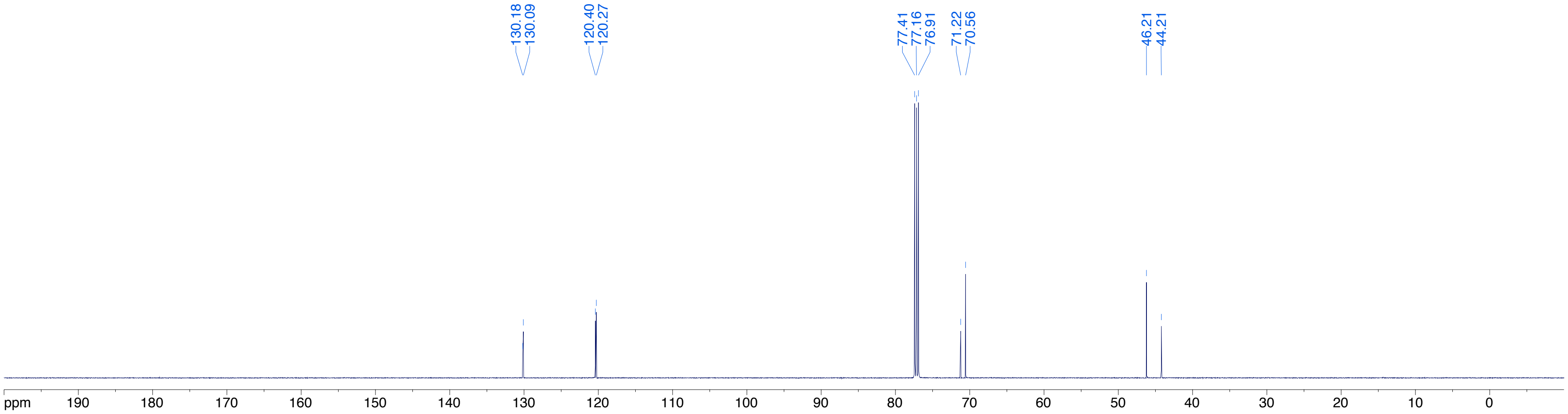

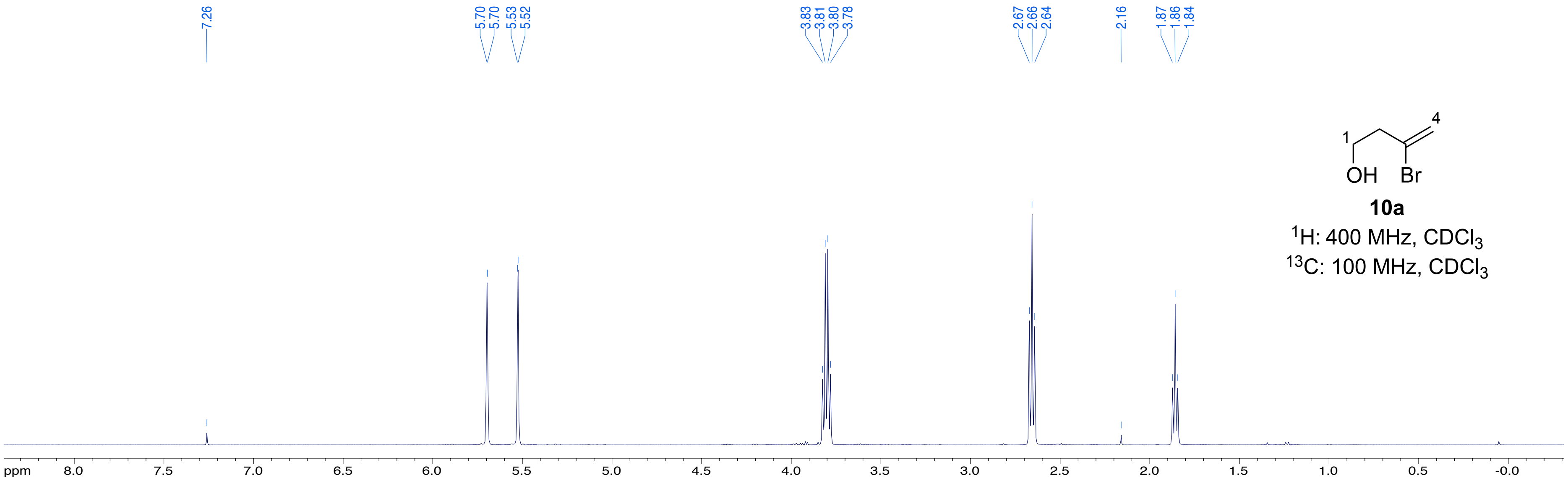

10a

^{1}H: 400 MHz, $CDCl_3$

^{13}C: 100 MHz, $CDCl_3$

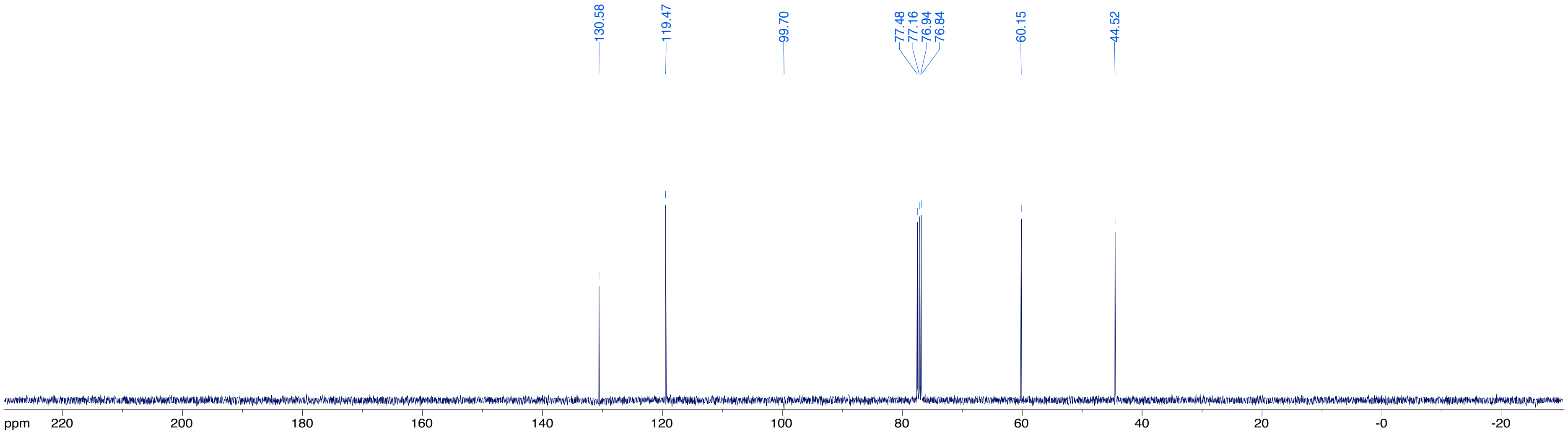

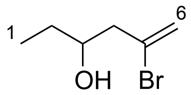

10b

^{1}H: 400 MHz, $CDCl_3$

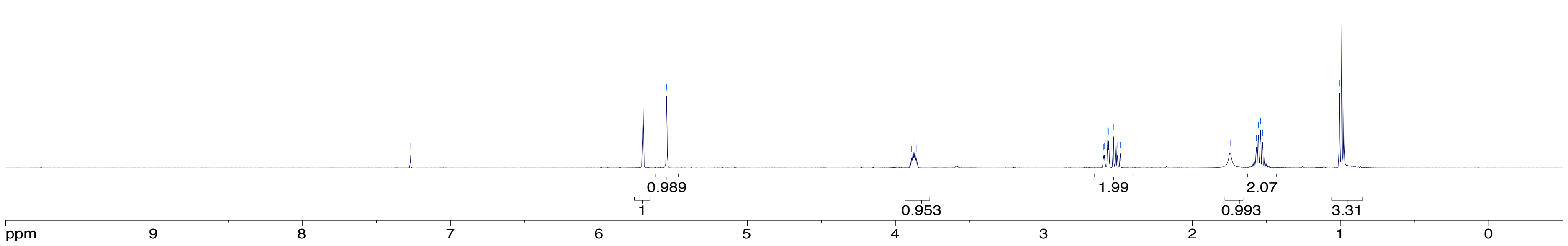

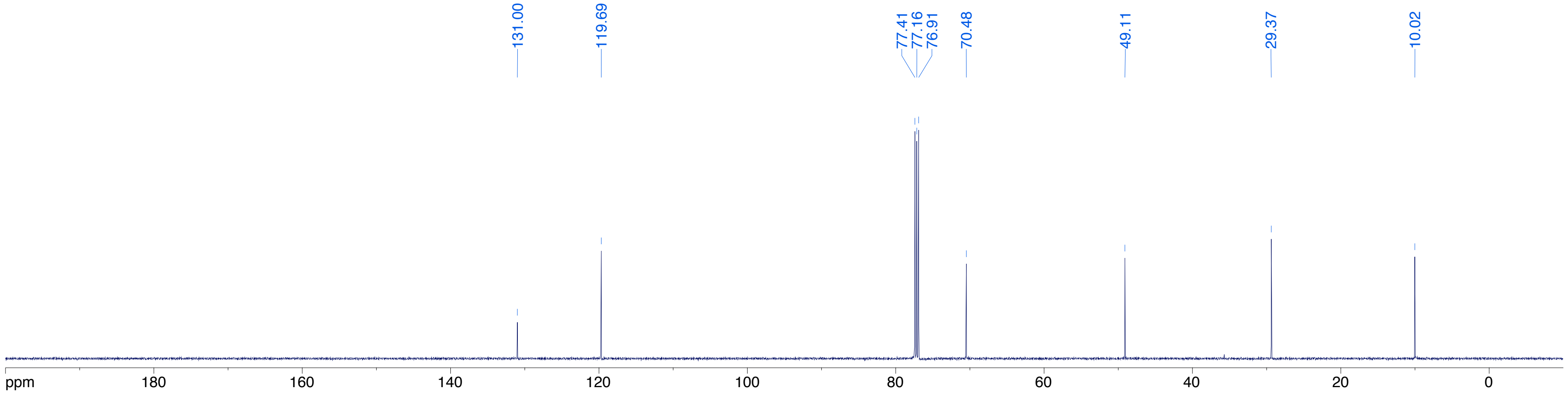

Data according to literature values. [1]

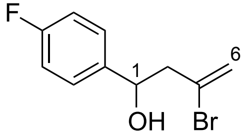

10c

^{1}H: 400 MHz, $CDCl_3$

^{13}C: 100 MHz, $CDCl_3$

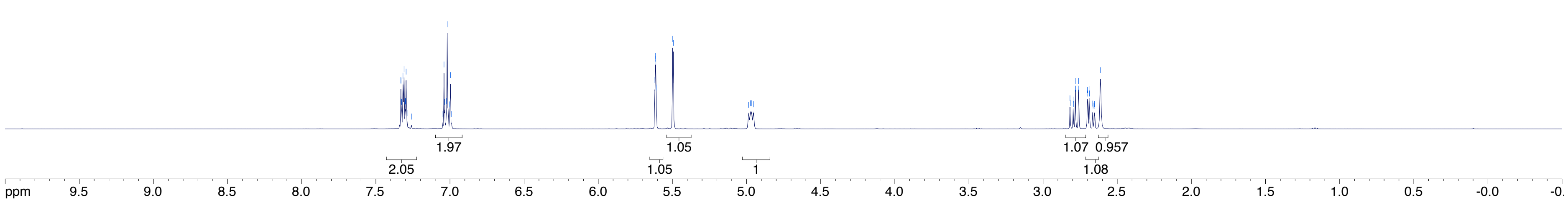

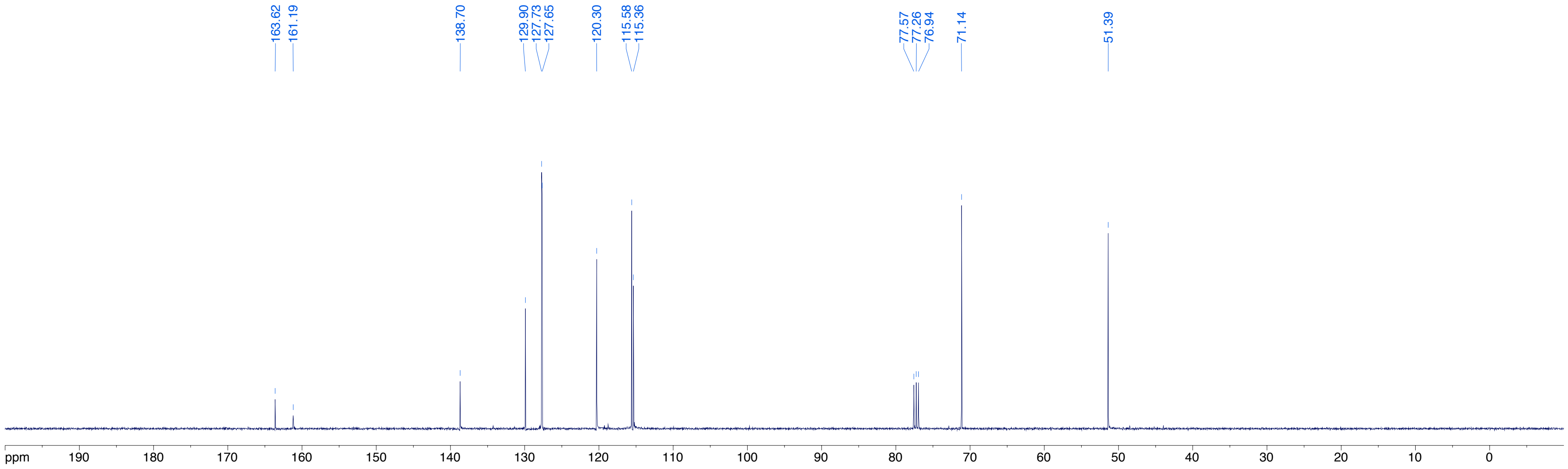

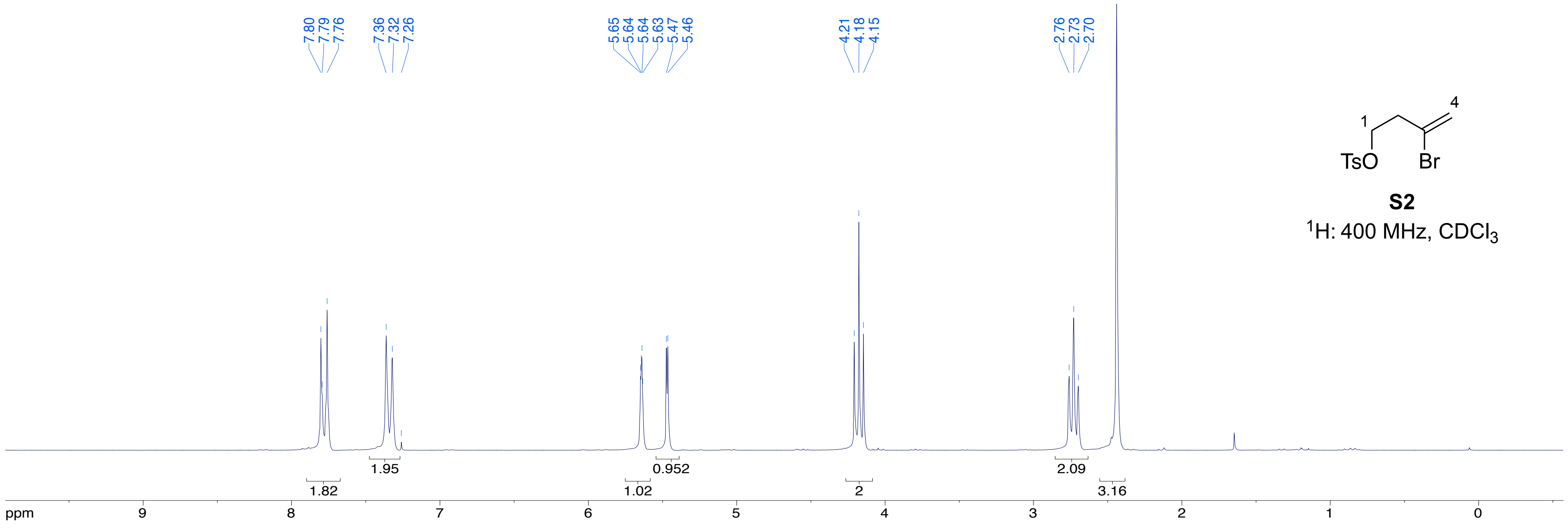

Data in accordance with literature values.[2],[3]

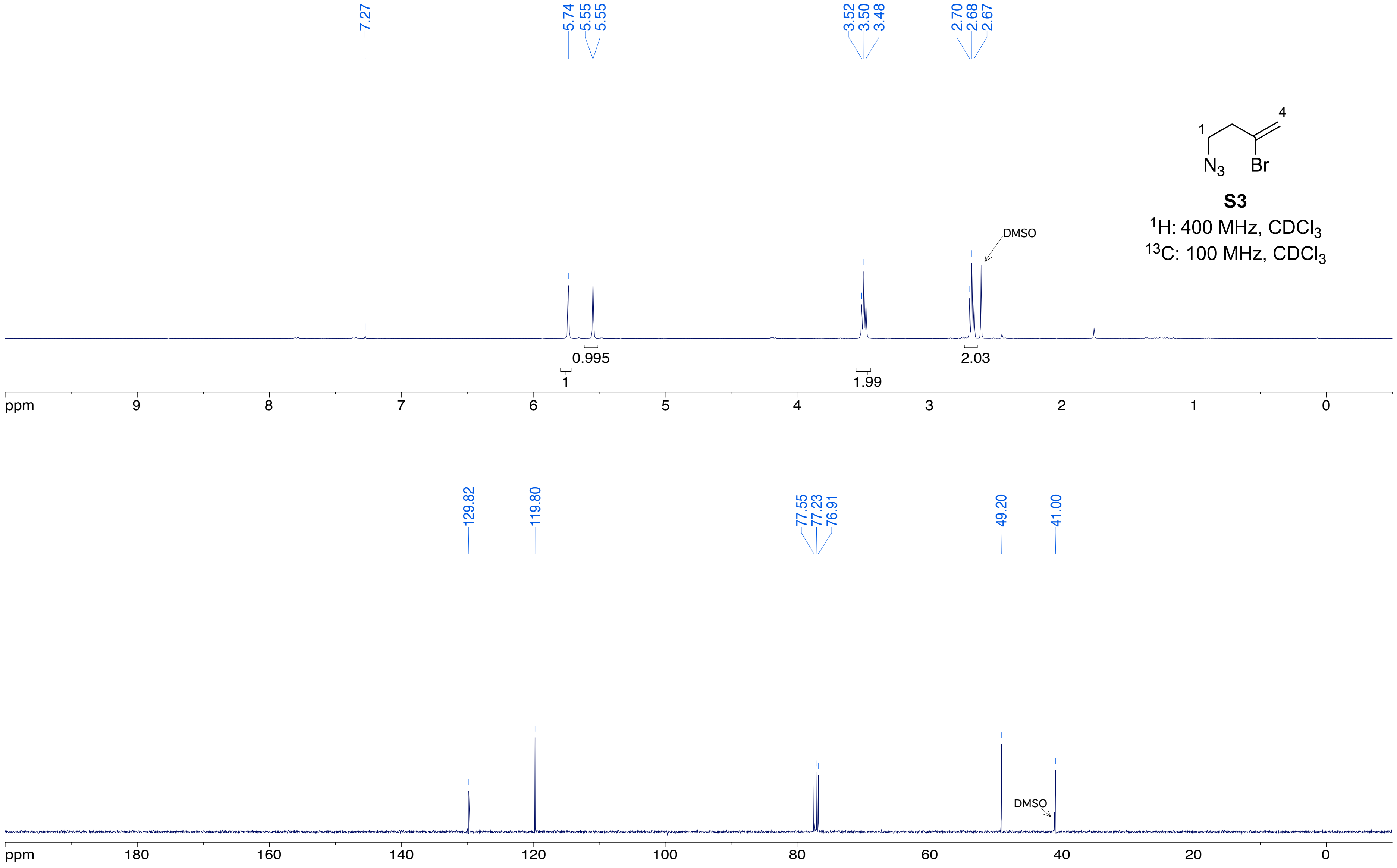

7.27
5.74
5.55
5.55
3.52
3.50
3.48
2.70
2.68
2.67
1
4
N3
Br
S3
1H: 400 MHz, CDCl3
13C: 100 MHz, CDCl3
DMSO
0.995
1
1.99
2.03
ppm
9
8
7
6
5
4
3
2
1
0
129.82
119.80
77.55
77.23
76.91
49.20
41.00
DMSO
ppm
180
160
140
120
100
80
60
40
20
0

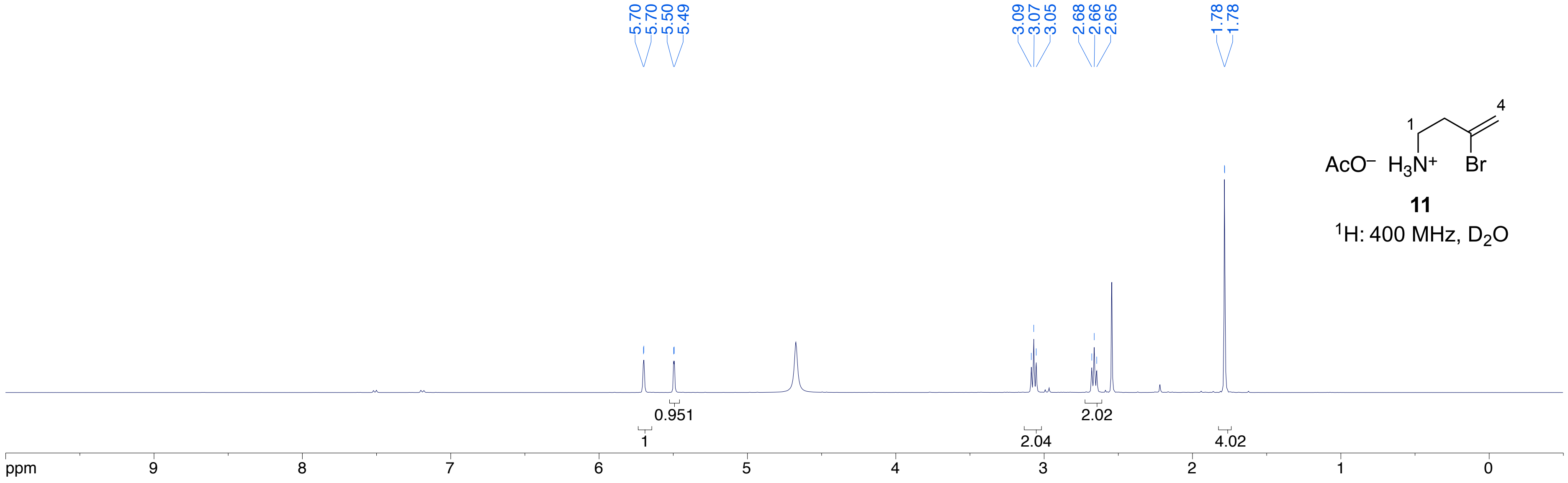

Data in accordance with literature values.[2]

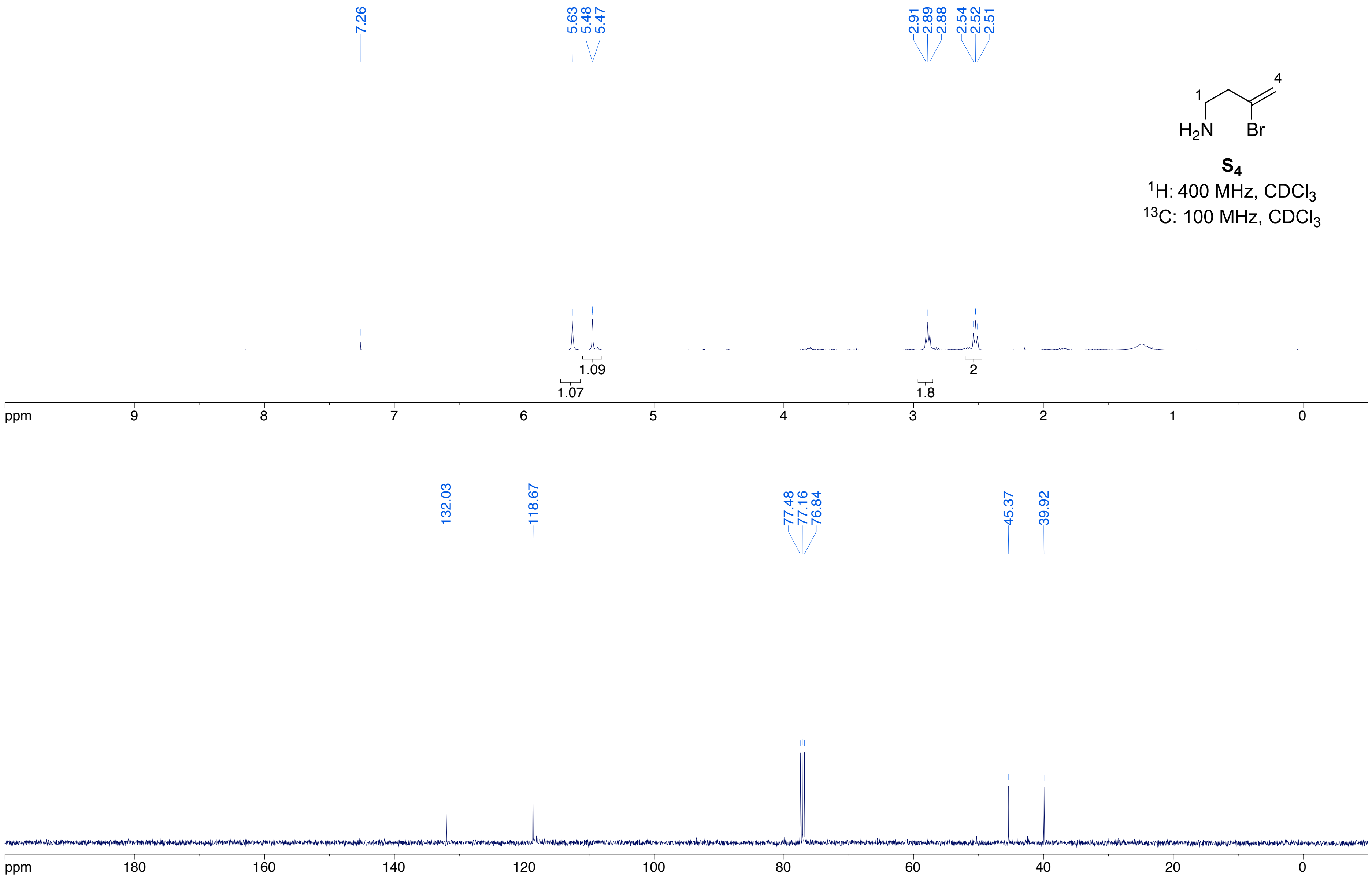

Data identical to literature values.[4]

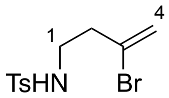

9a

^{1}H: 500 MHz, $CDCl_3$

^{13}C: 125 MHz, $CDCl_3$

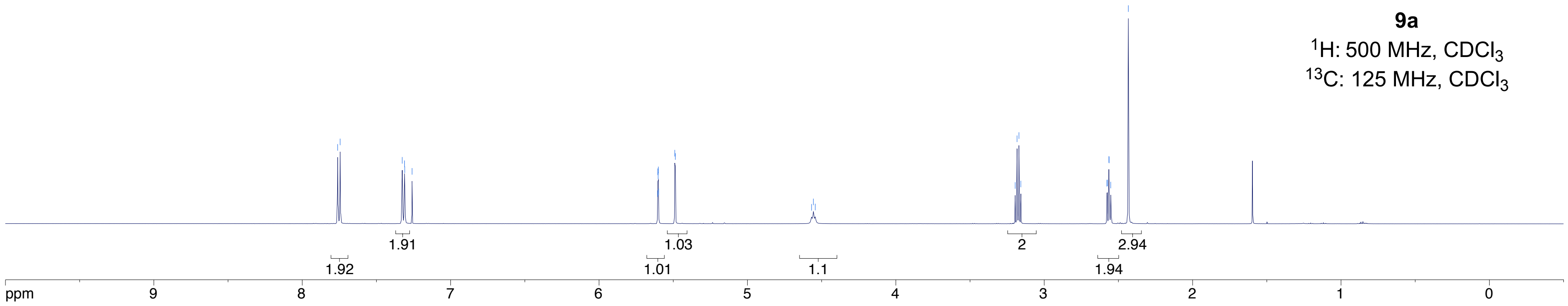

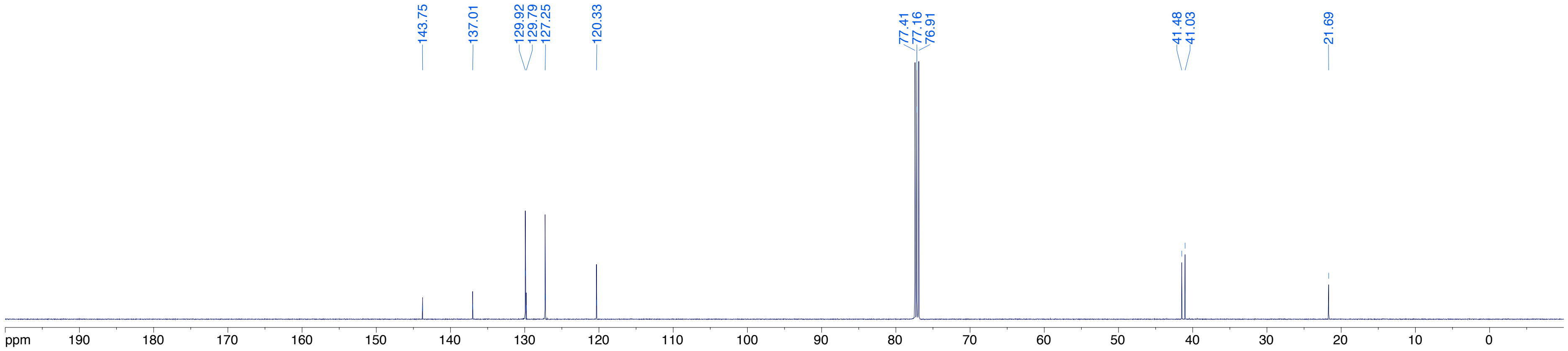

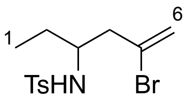

9b

^{1}H: 500 MHz, $CDCl_3$

^{13}C: 125 MHz, $CDCl_3$

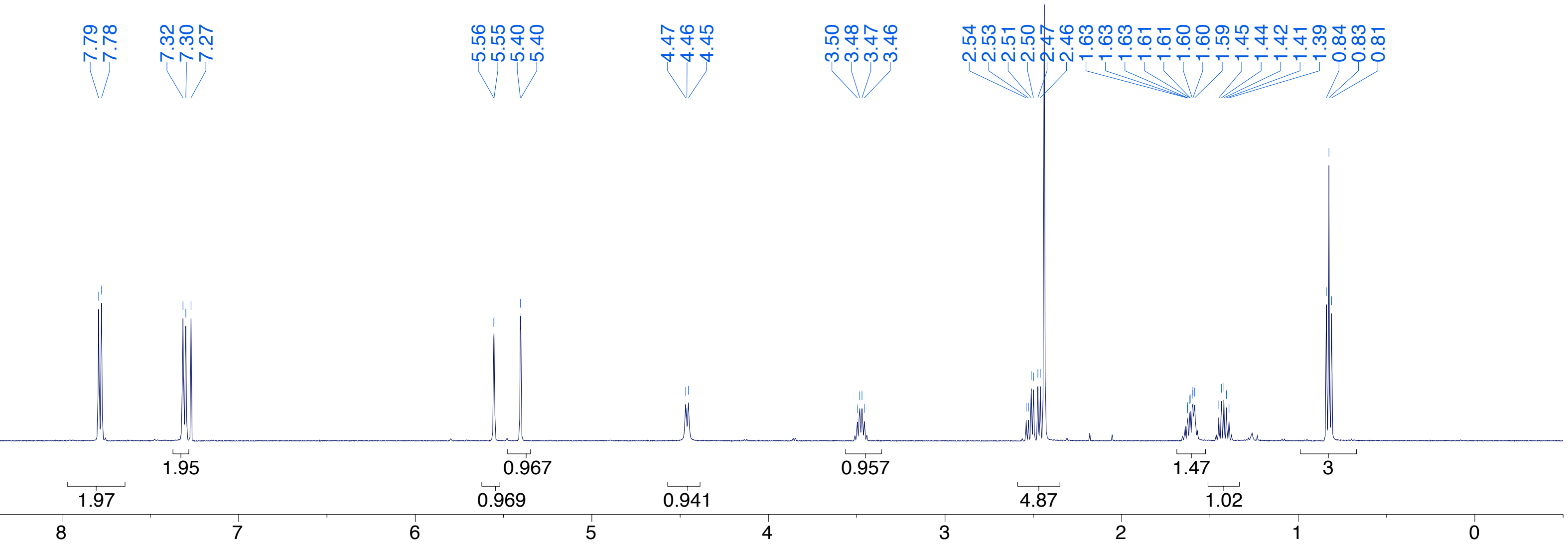

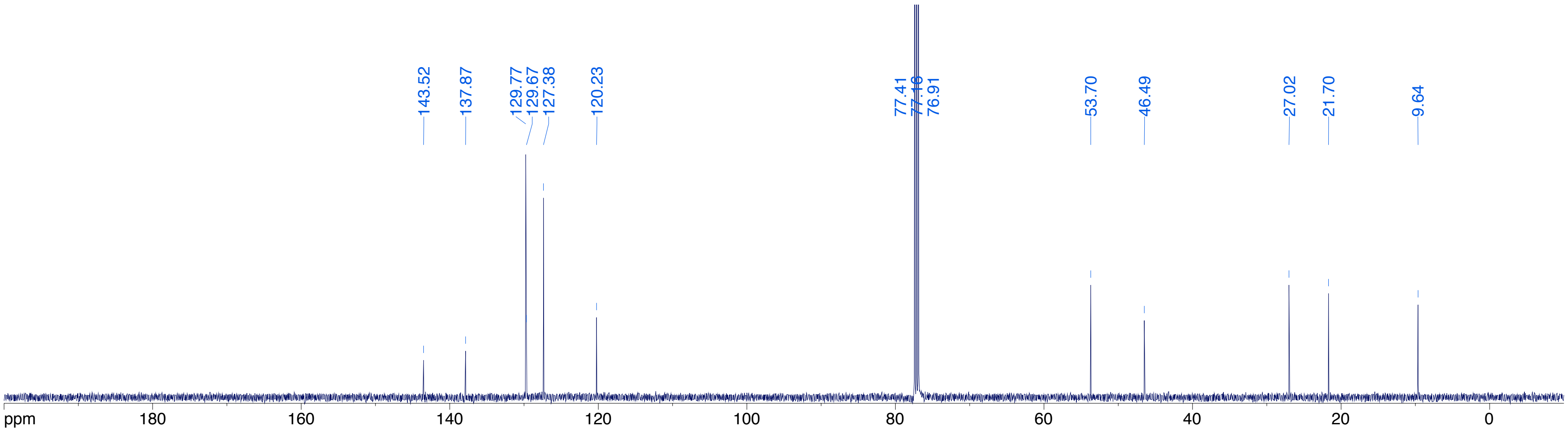

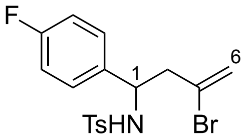

10c

^{1}H: 500 MHz, $CDCl_3$

^{13}C: 125 MHz, $CDCl_3$

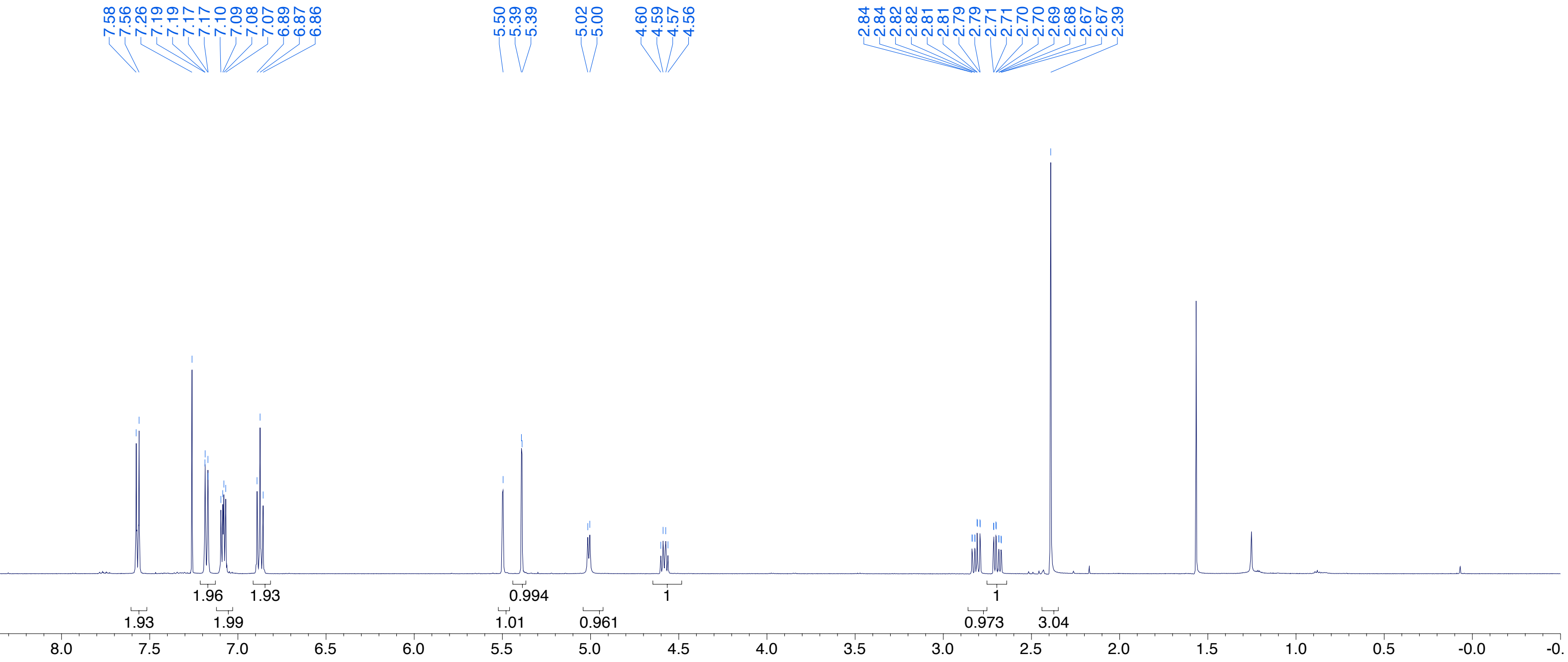

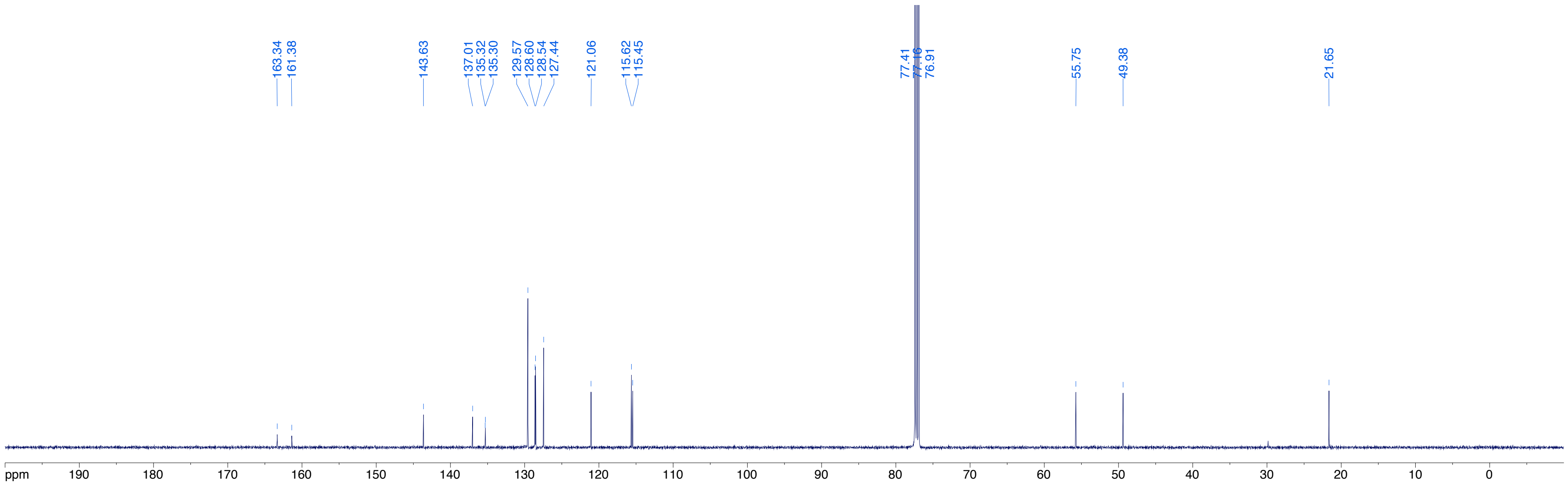

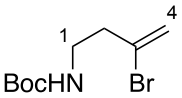

9d

^{1}H: 400 MHz, $CDCl_3$

^{13}C: 100 MHz, $CDCl_3$

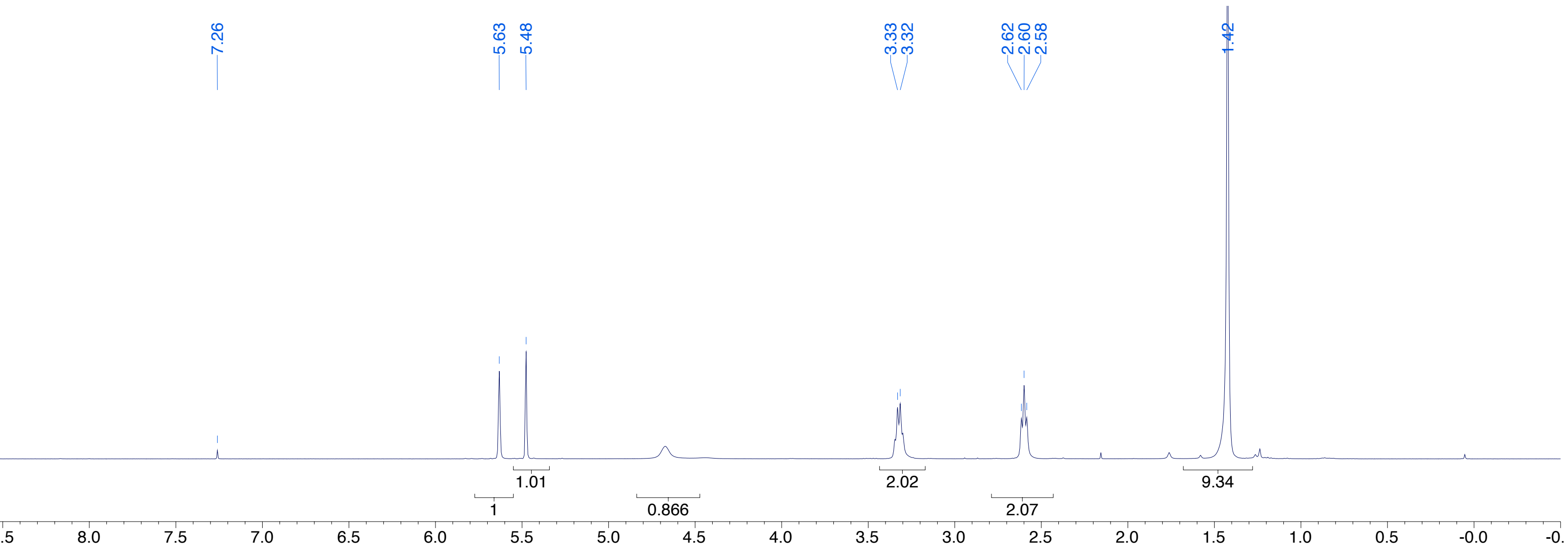

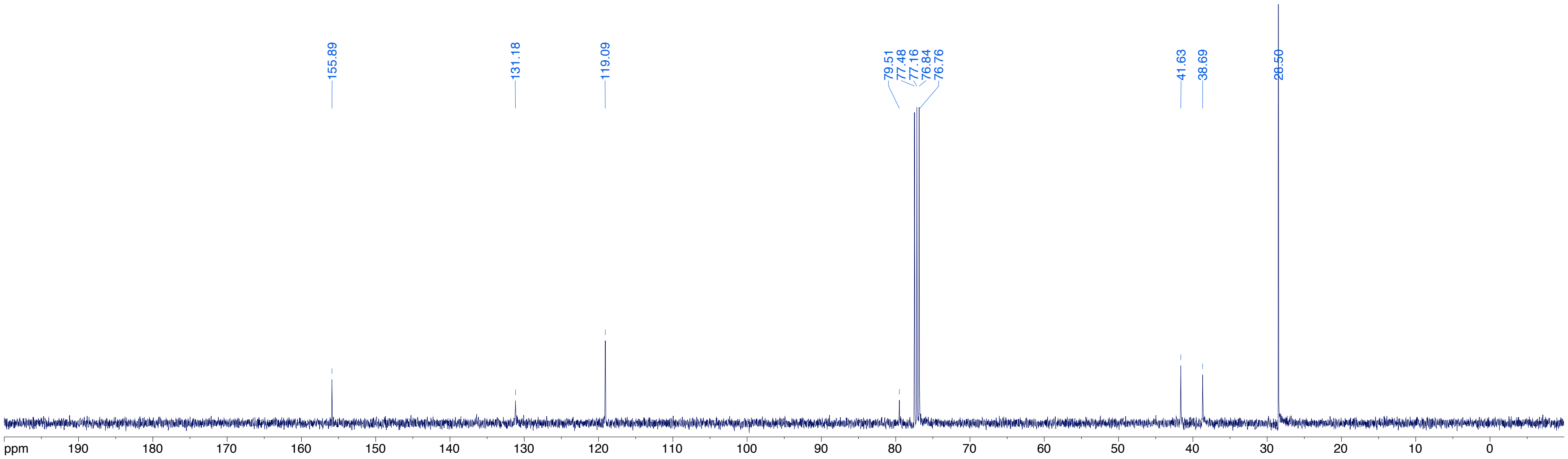

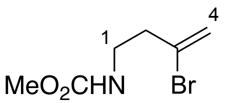

9e

^{1}H: 400 MHz, $CDCl_3$

^{13}C: 100 MHz, $CDCl_3$

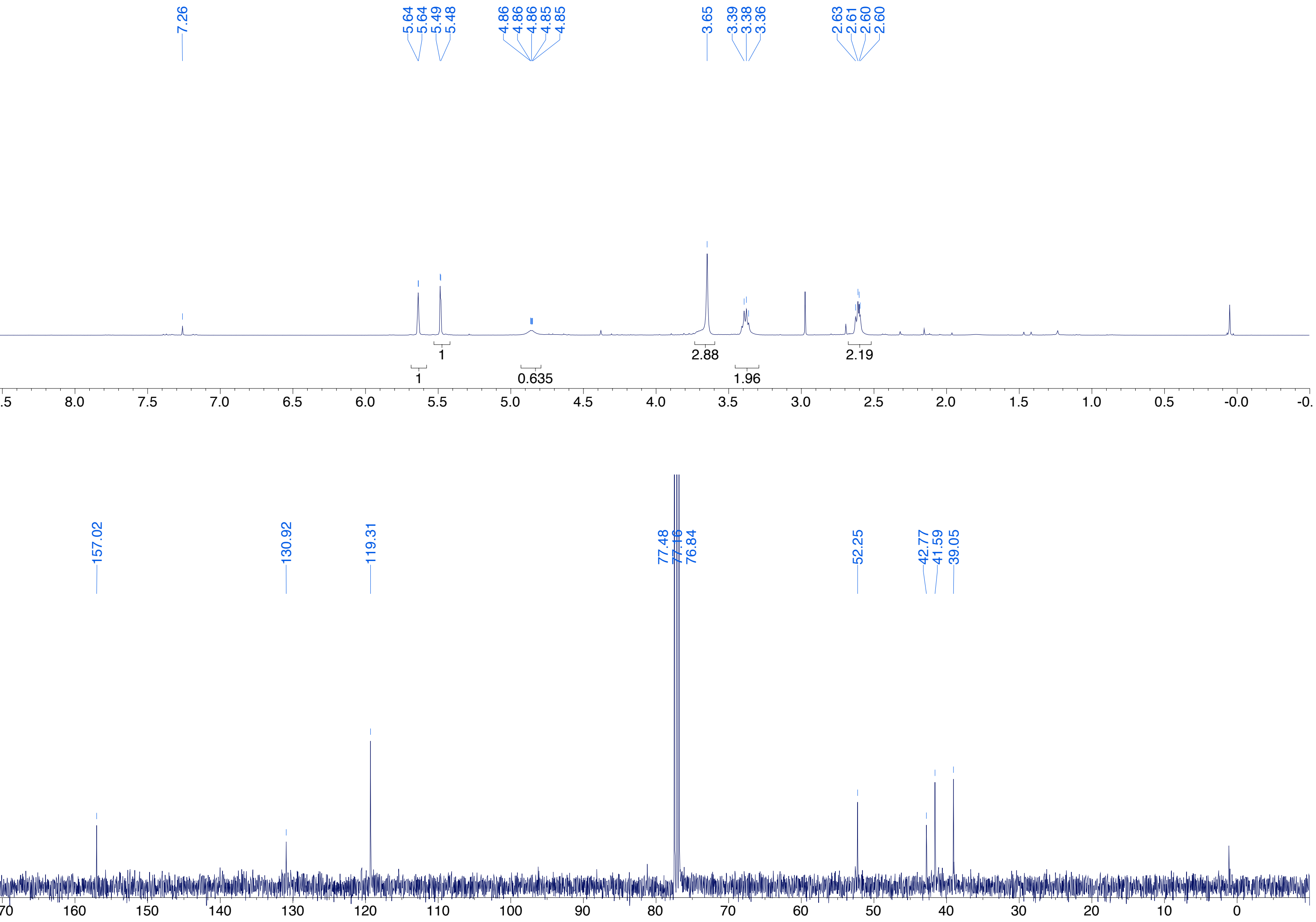

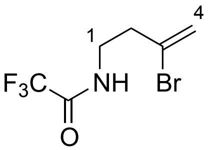

9g

^{1}H: 400 MHz, $CDCl_3$

^{13}C: 100 MHz, $CDCl_3$

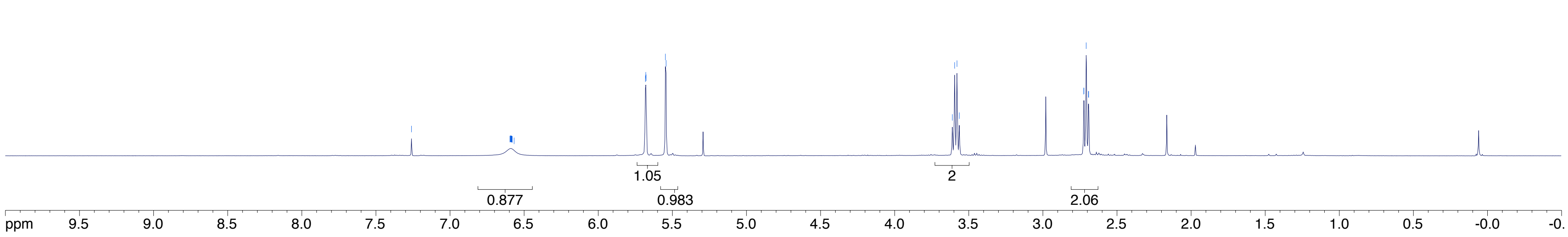

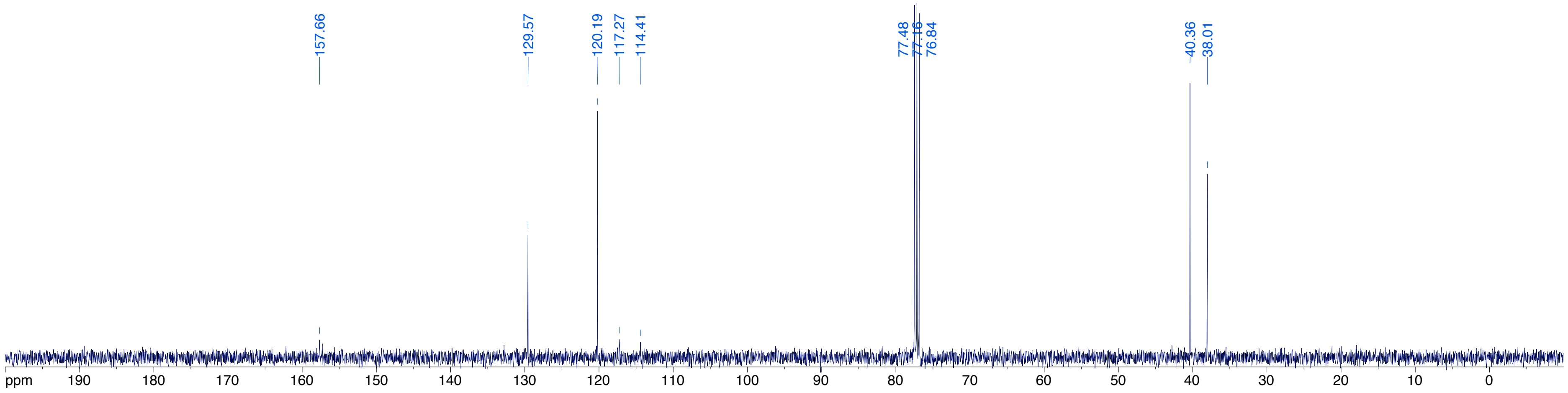

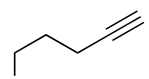

S5

^{1}H: 400 MHz, $CDCl_3$

^{13}C: 100 MHz, $CDCl_3$

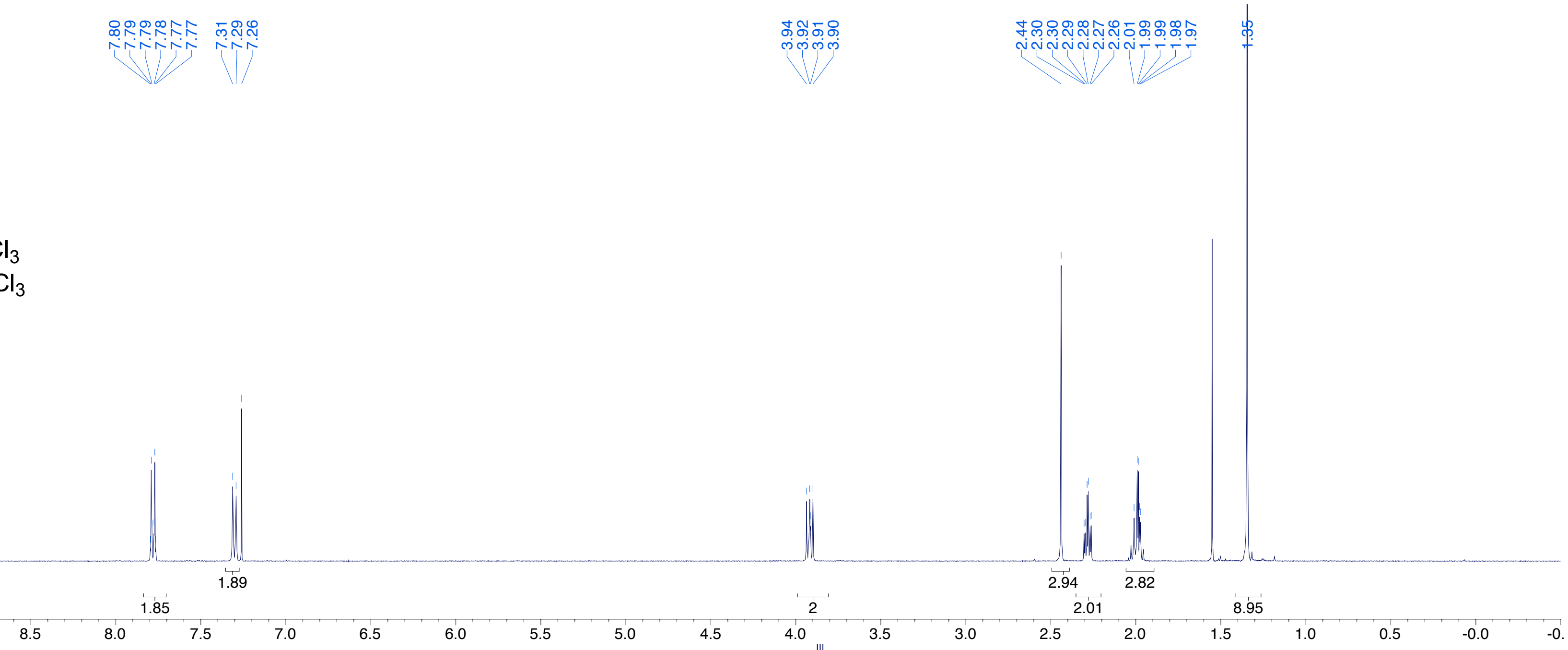

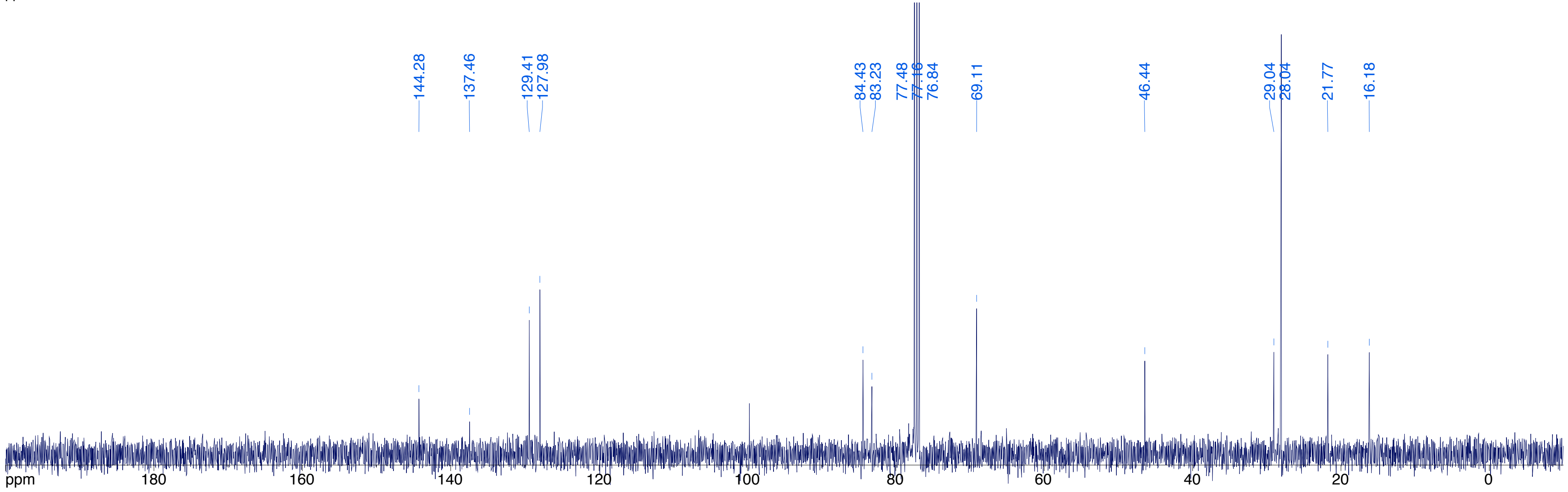

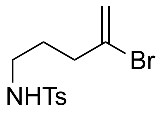

9h

^1H: 200 MHz, $CDCl_3$

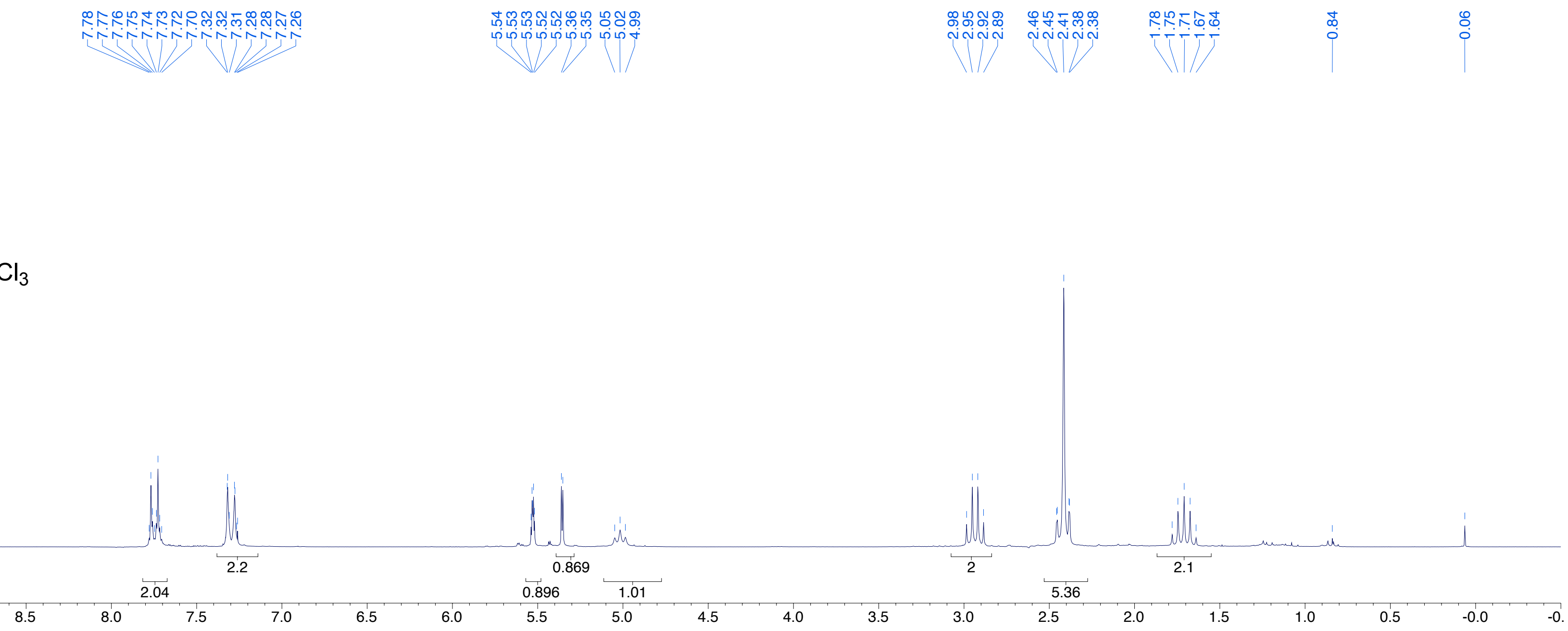

Data in accordance with literature values.[5]

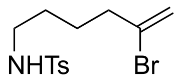

9i

^{1}H: 500 MHz, $CDCl_3$

^{13}C: 125 MHz, $CDCl_3$

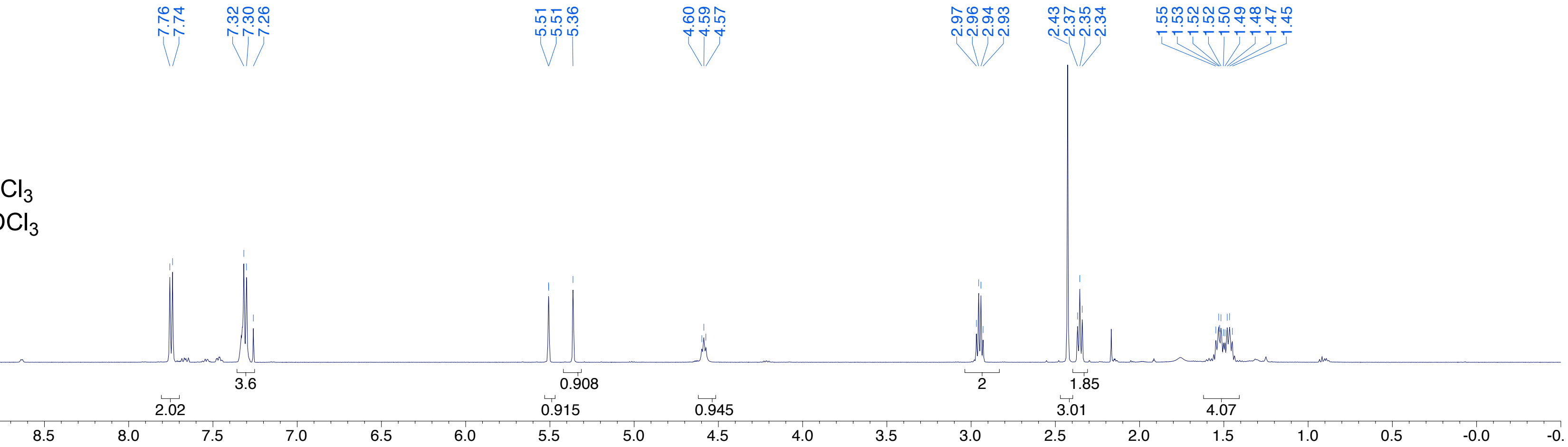

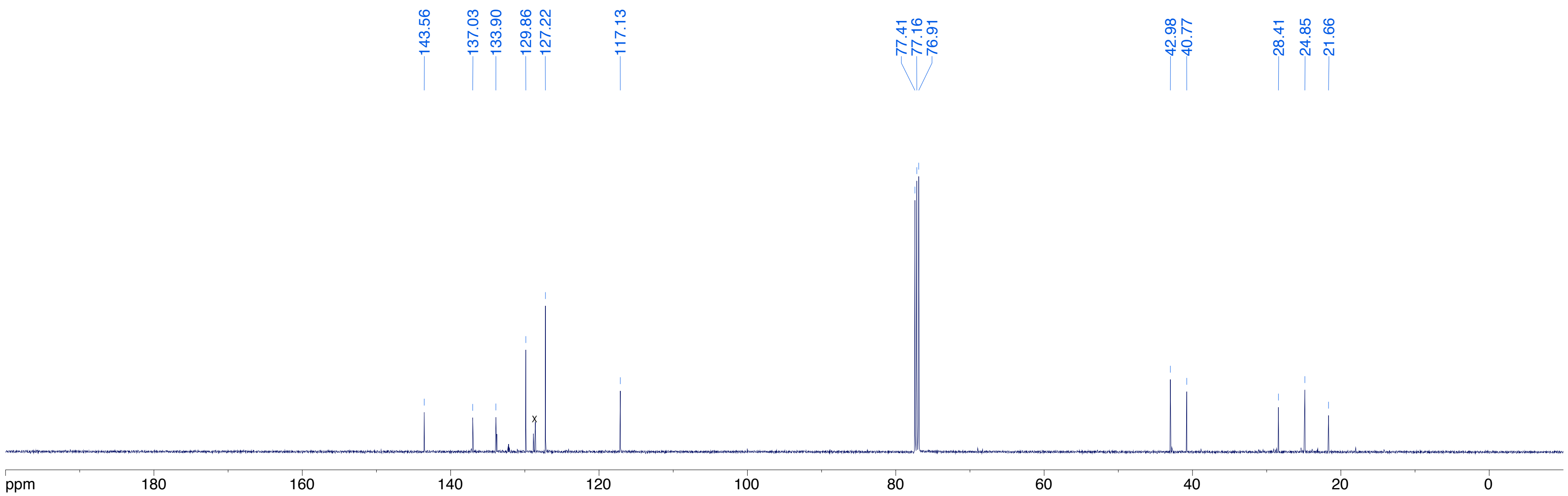

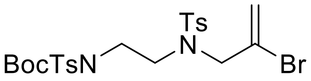

S7

^{1}H: 400 MHz, $CDCl_3$

unstable compond

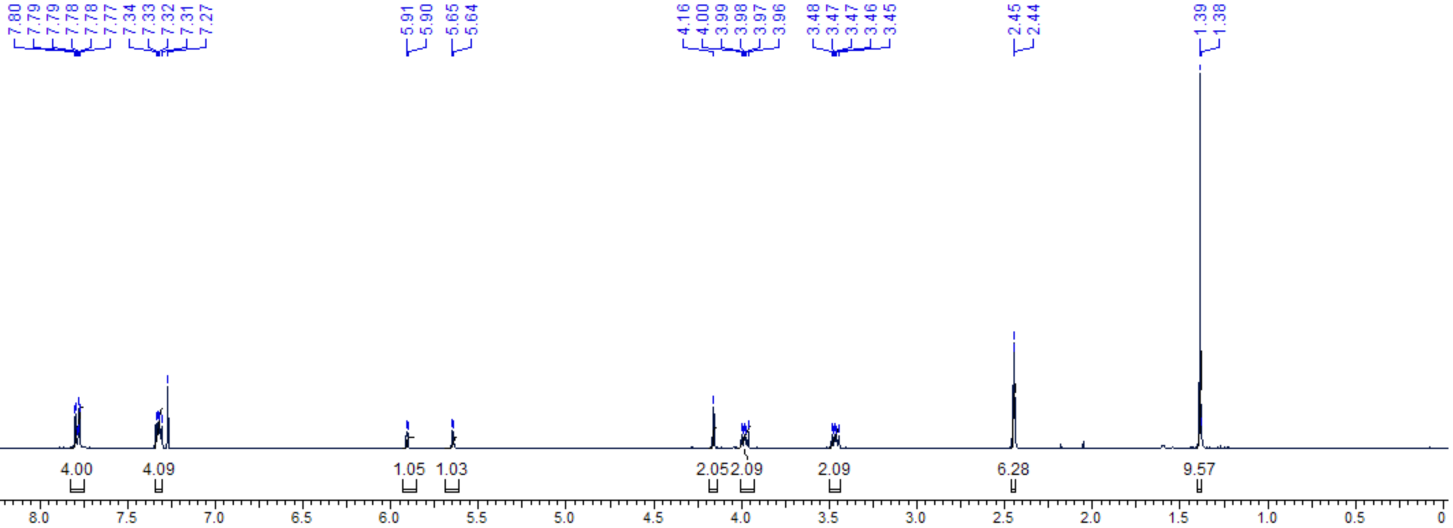

Bromoalkynes

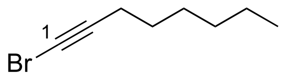

13a

^{1}H NMR (400 MHz, $CDCl_3$)

^{13}C NMR (100 MHz, $CDCl_3$)

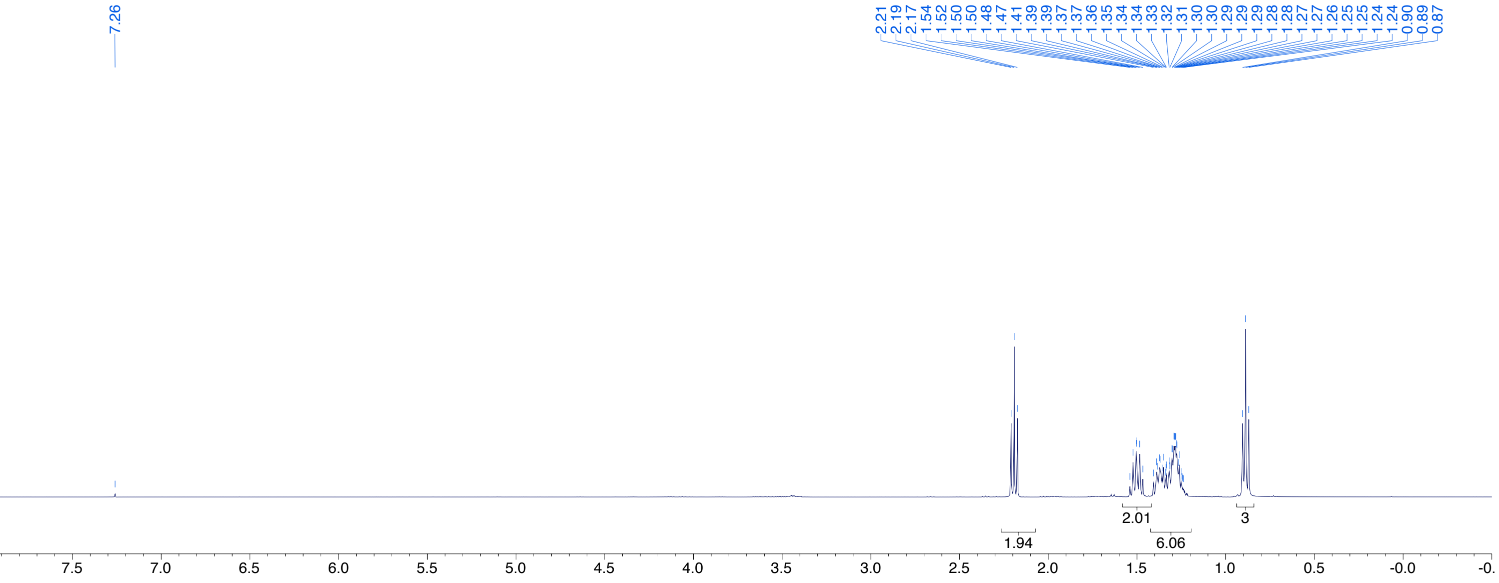

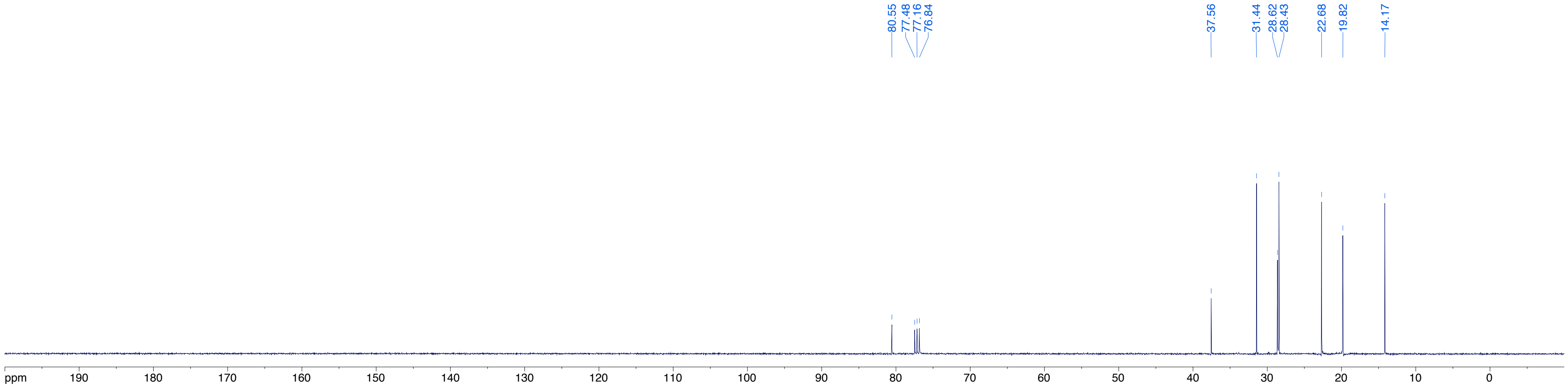

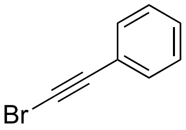

13b

^{1}H NMR (400 MHz, $CDCl_3$)

^{13}C NMR (100 MHz, $CDCl_3$)

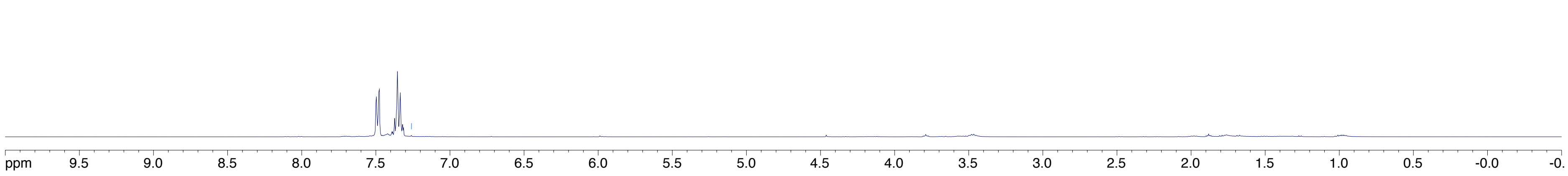

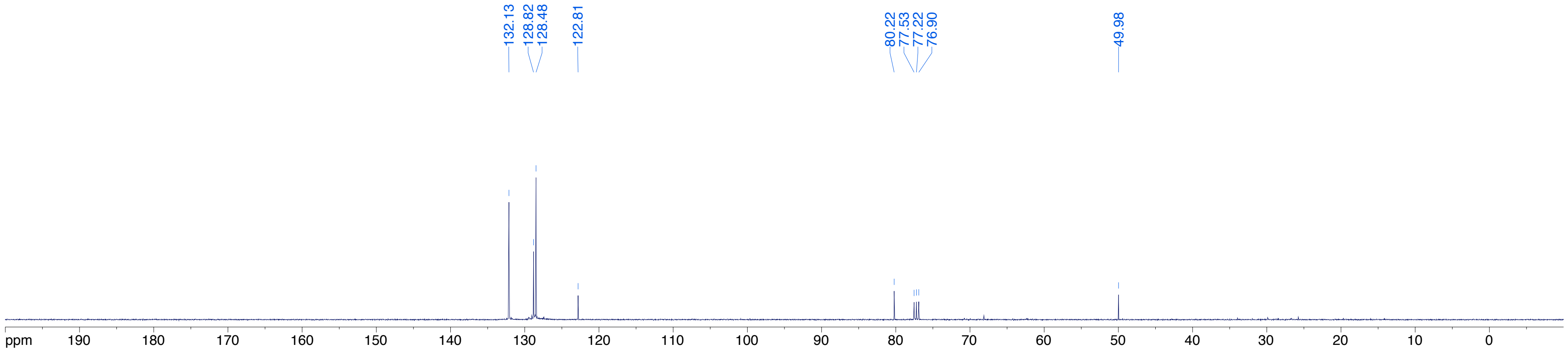

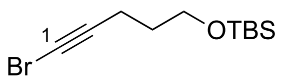

13c

^{1}H NMR (400 MHz, $CDCl_3$)

^{13}C NMR (100 MHz, $CDCl_3$)

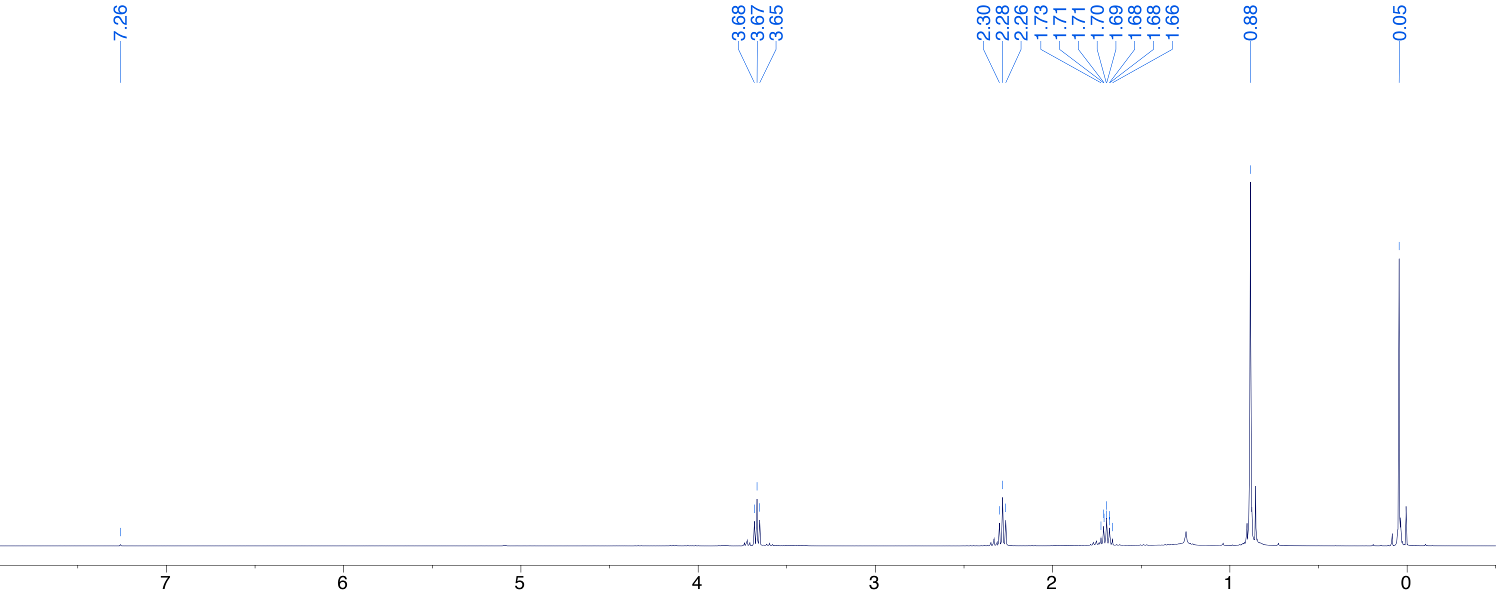

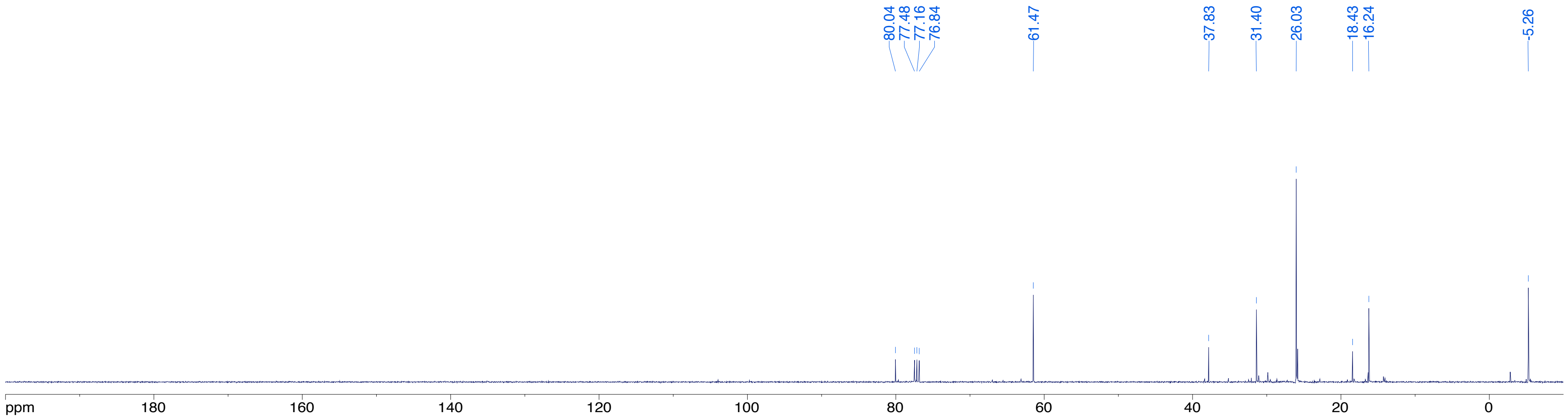

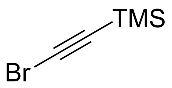

13d

^{1}H NMR (400 MHz, $CDCl_3$)

^{13}C NMR (100 MHz, $CDCl_3$)

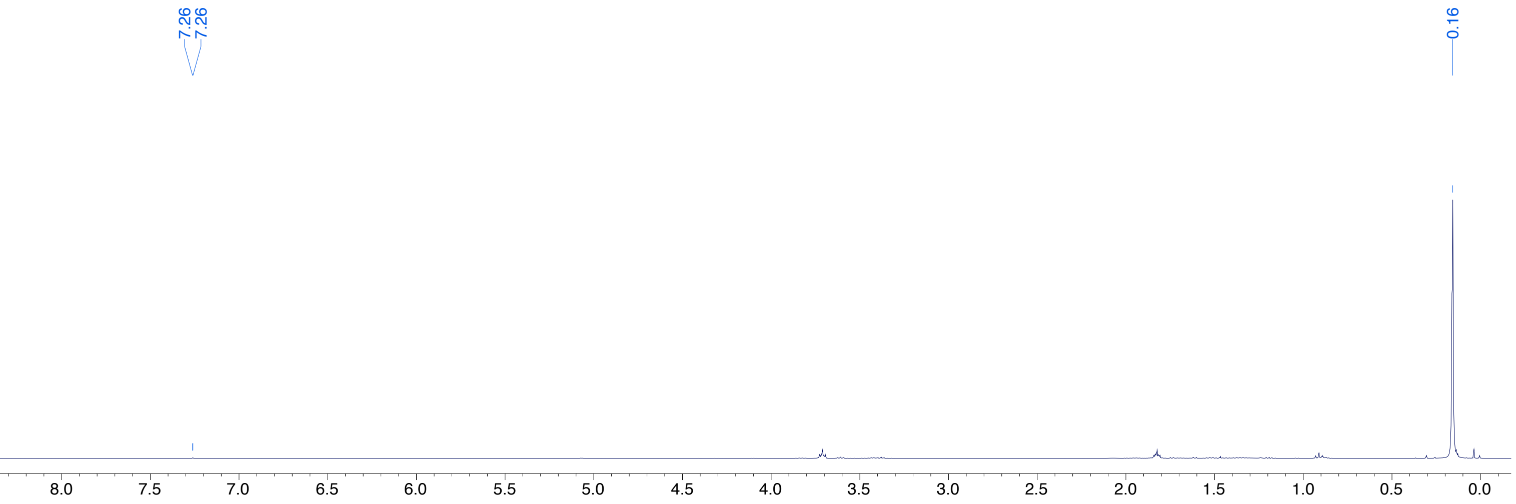

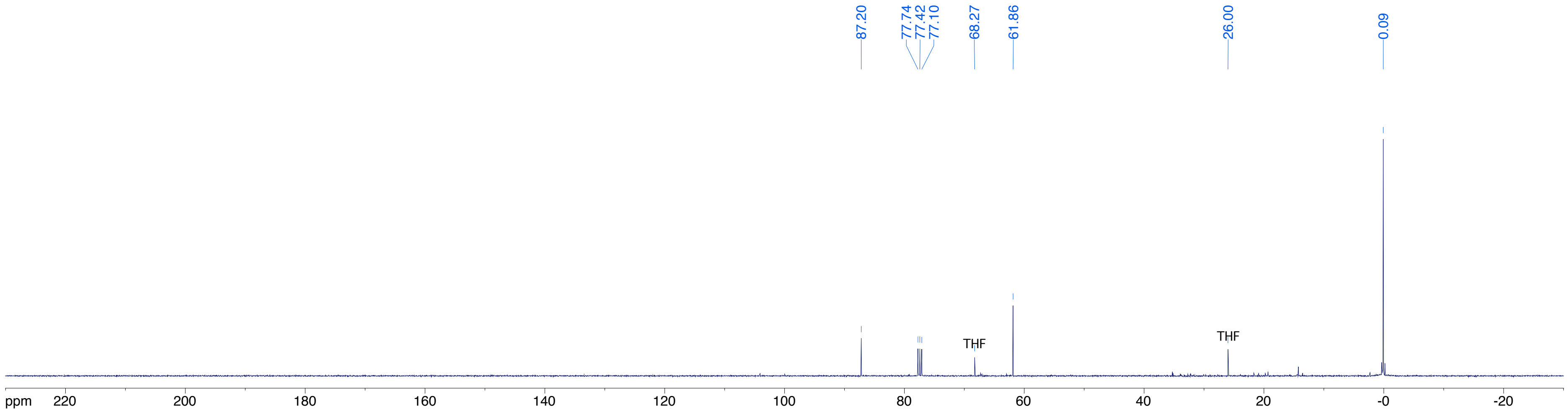

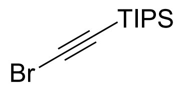

13e

^{1}H NMR (400 MHz, $CDCl_3$)

^{13}C NMR (100 MHz, $CDCl_3$)

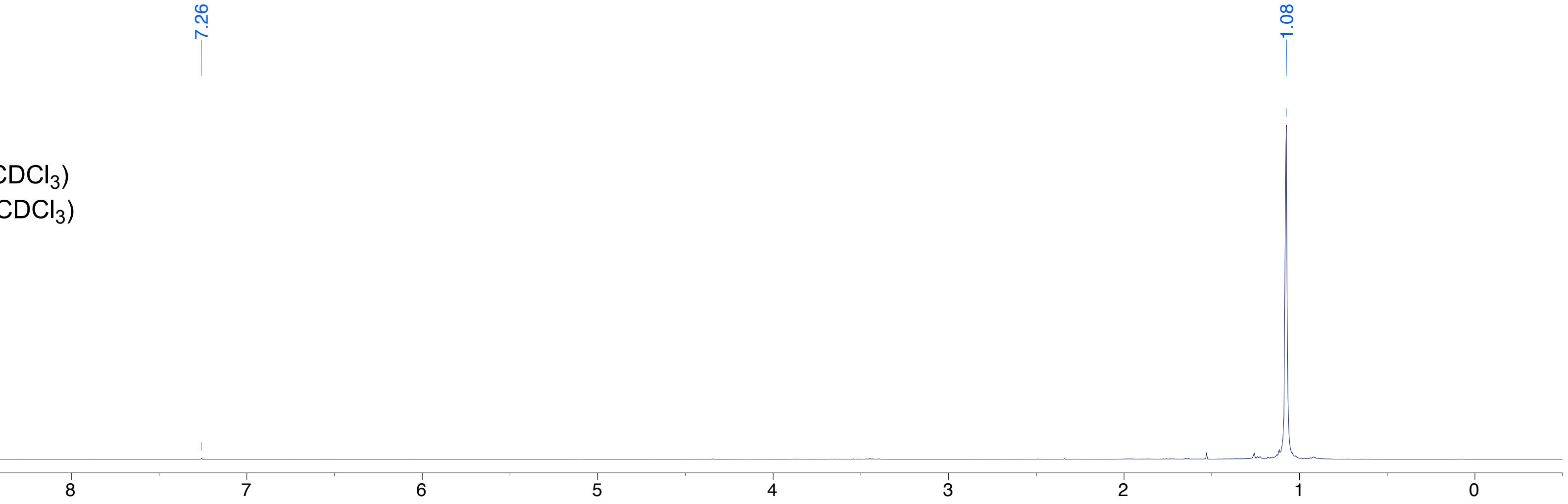

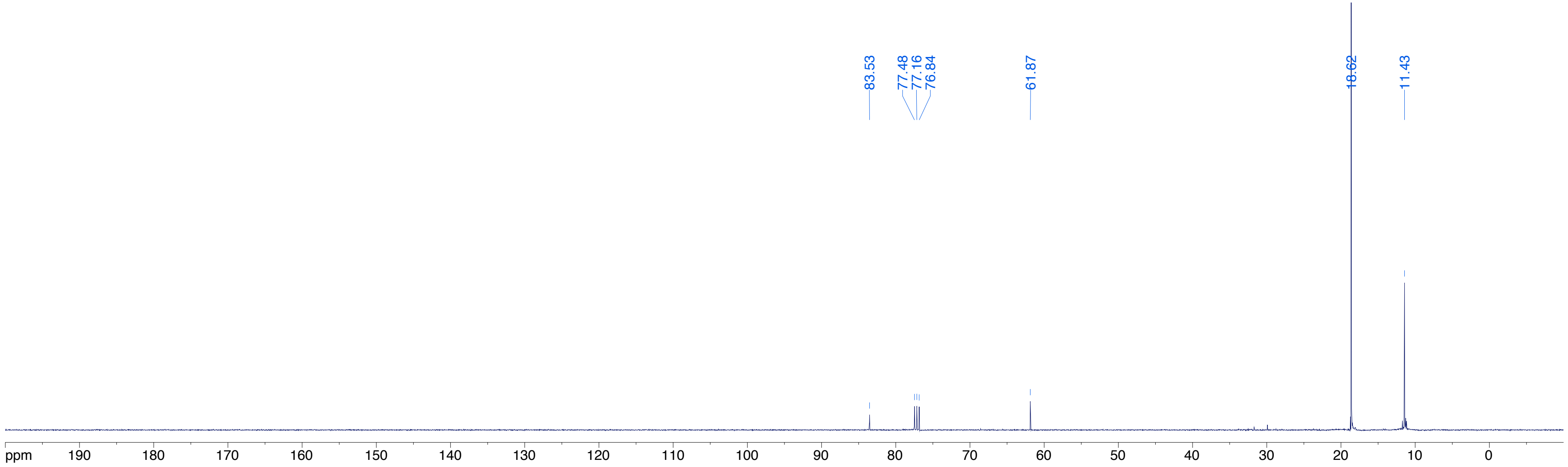

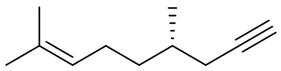

SB

^{1}H: 400 MHz, $CDCl_3$

^{13}C: 101 MHz, $CDCl_3$

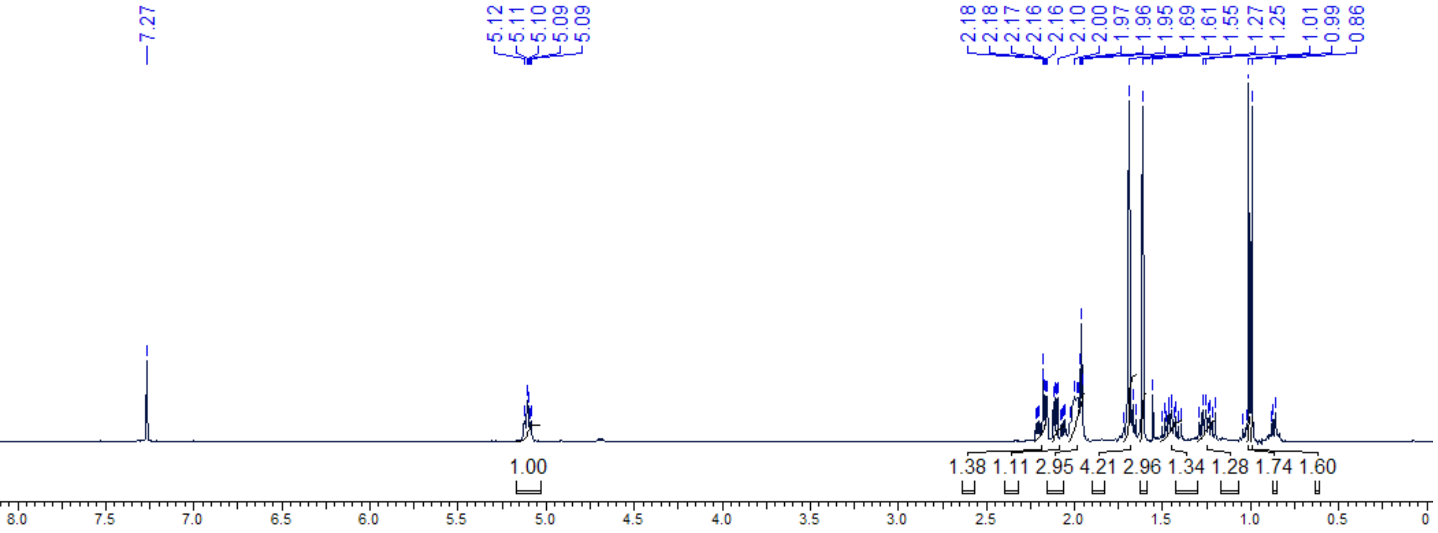

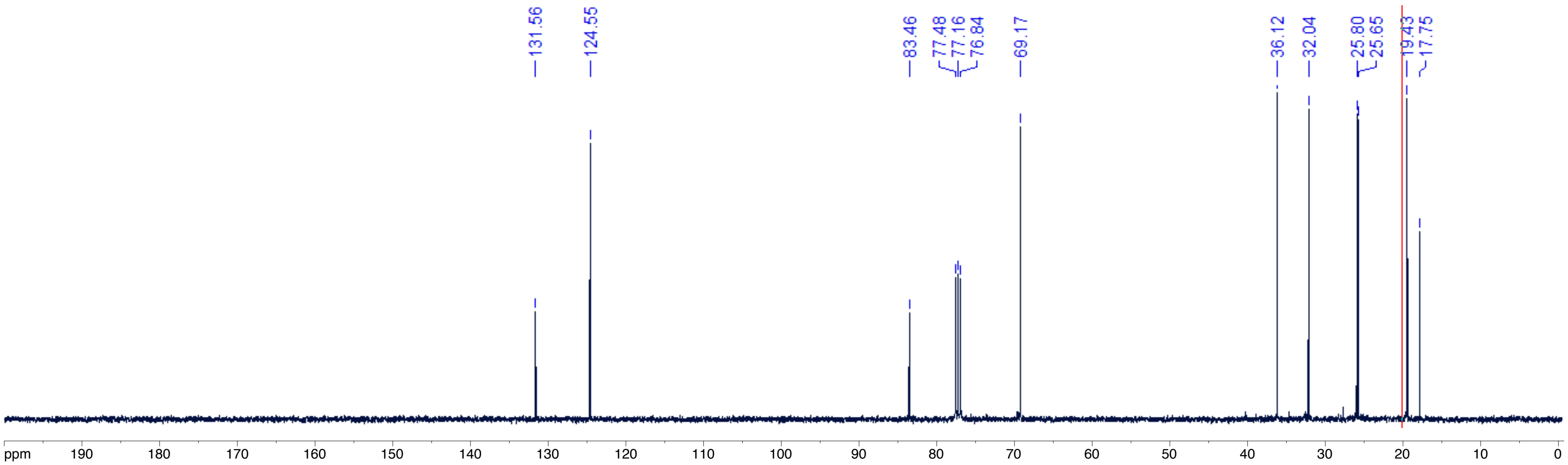

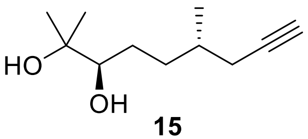

15

^{1}H: 400 MHz, $CDCl_3$

^{13}C: 101 MHz, $CDCl_3$

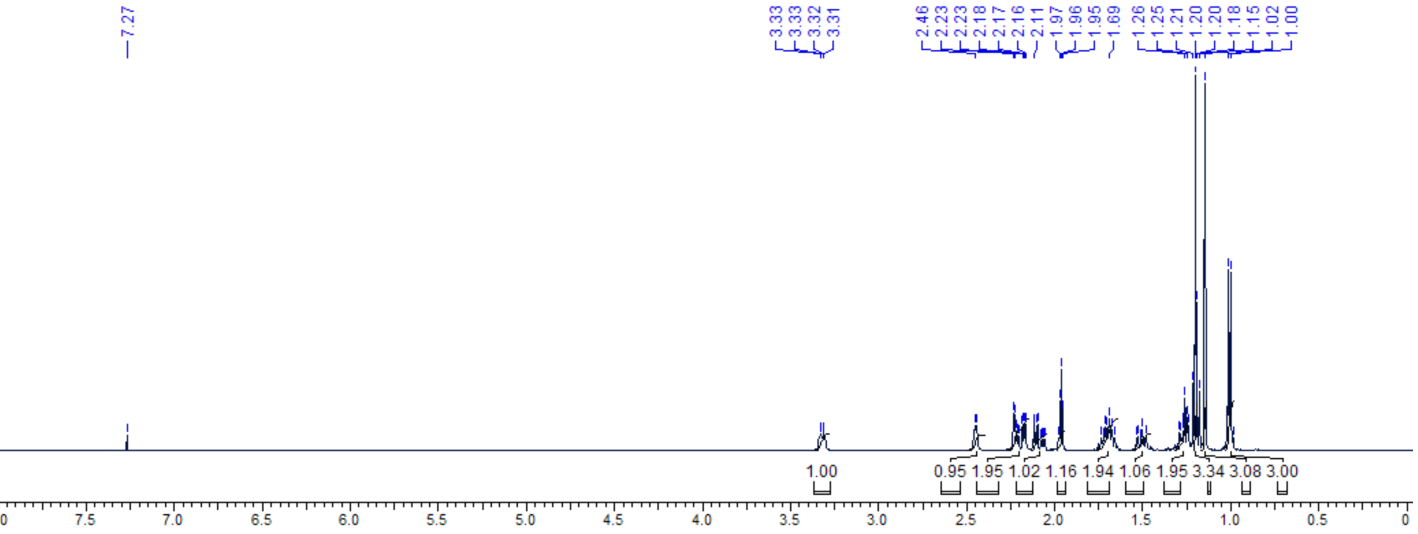

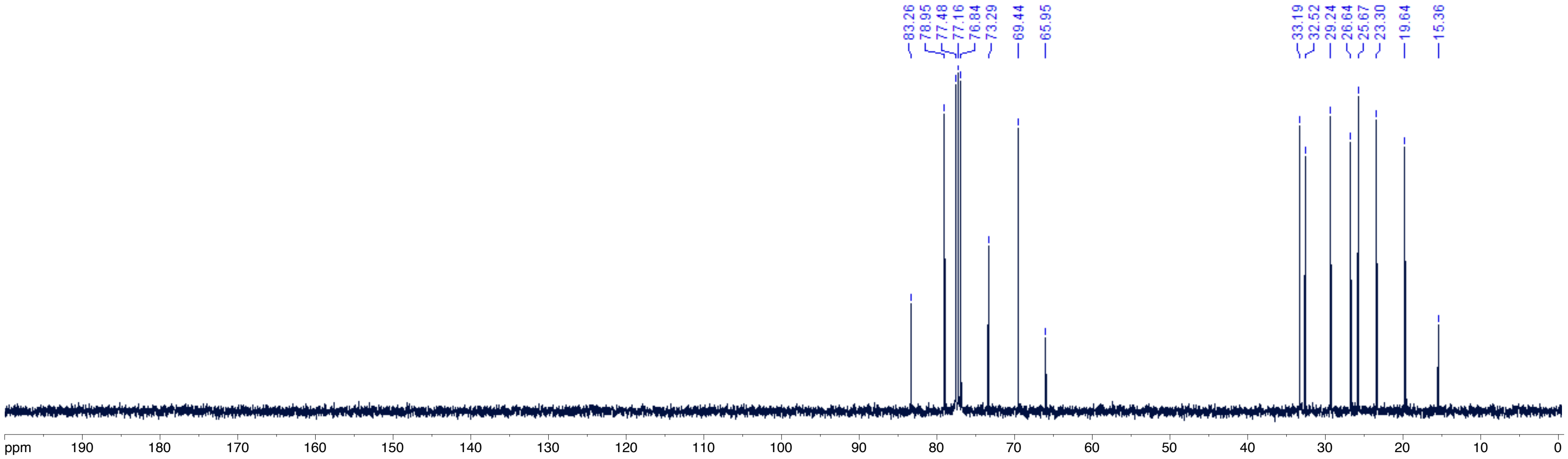

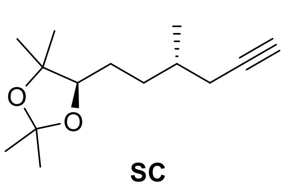

SC

^{1}H: 400 MHz, $CDCl_3$

^{13}C: 101 MHz, $CDCl_3$

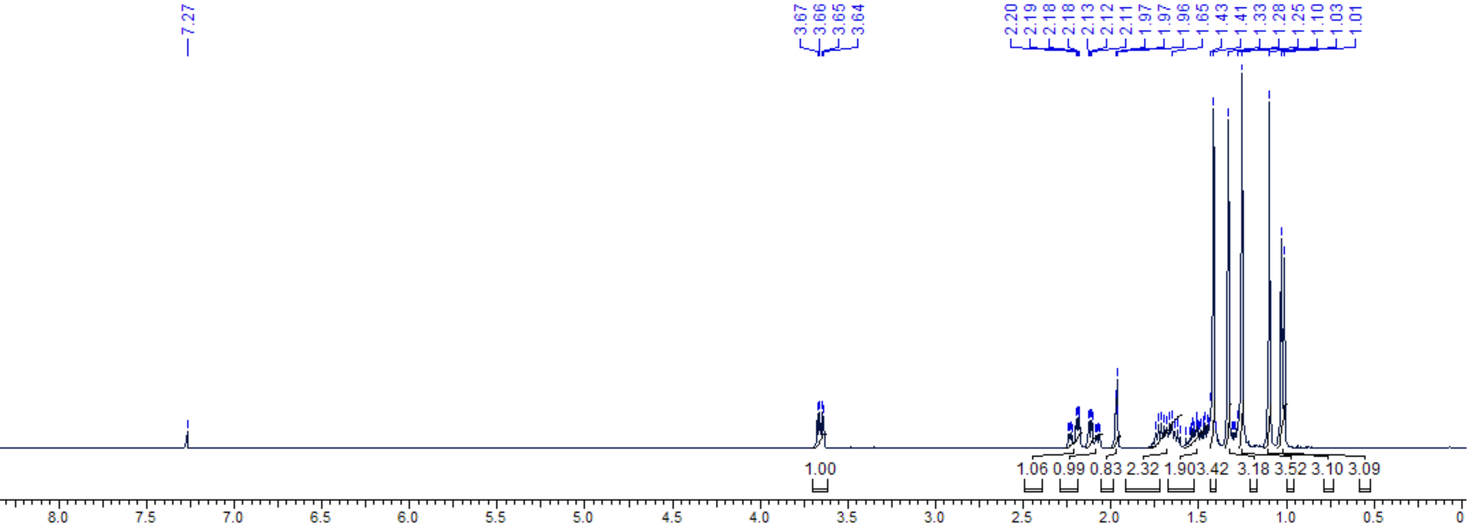

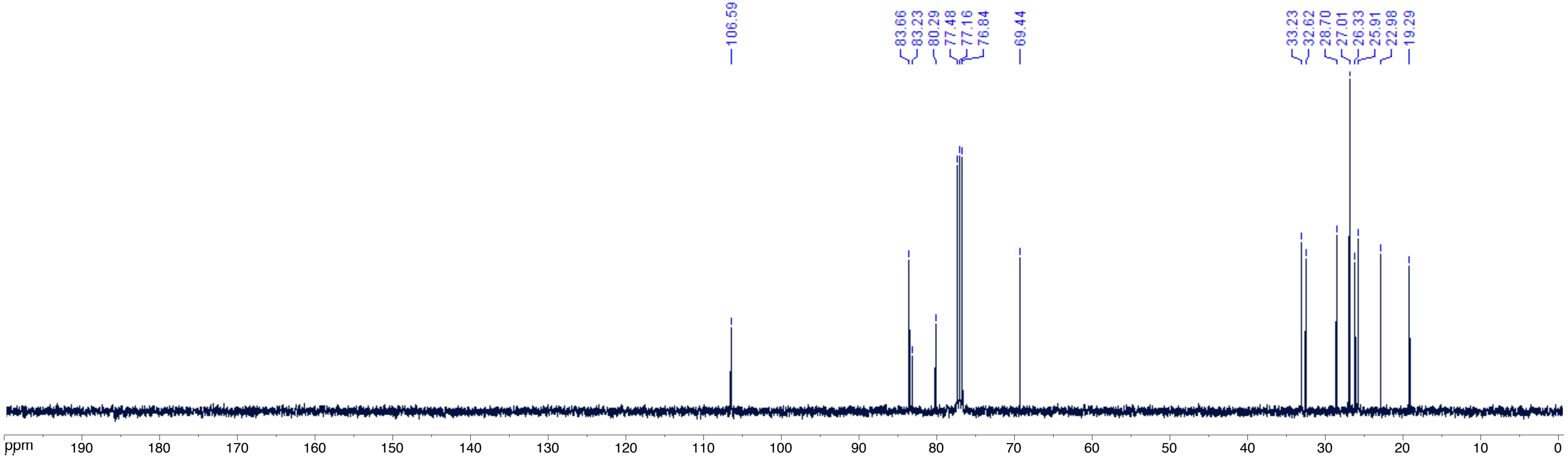

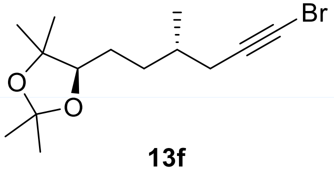

13f

^{1}H: 400 MHz, $CDCl_3$

unstable compound

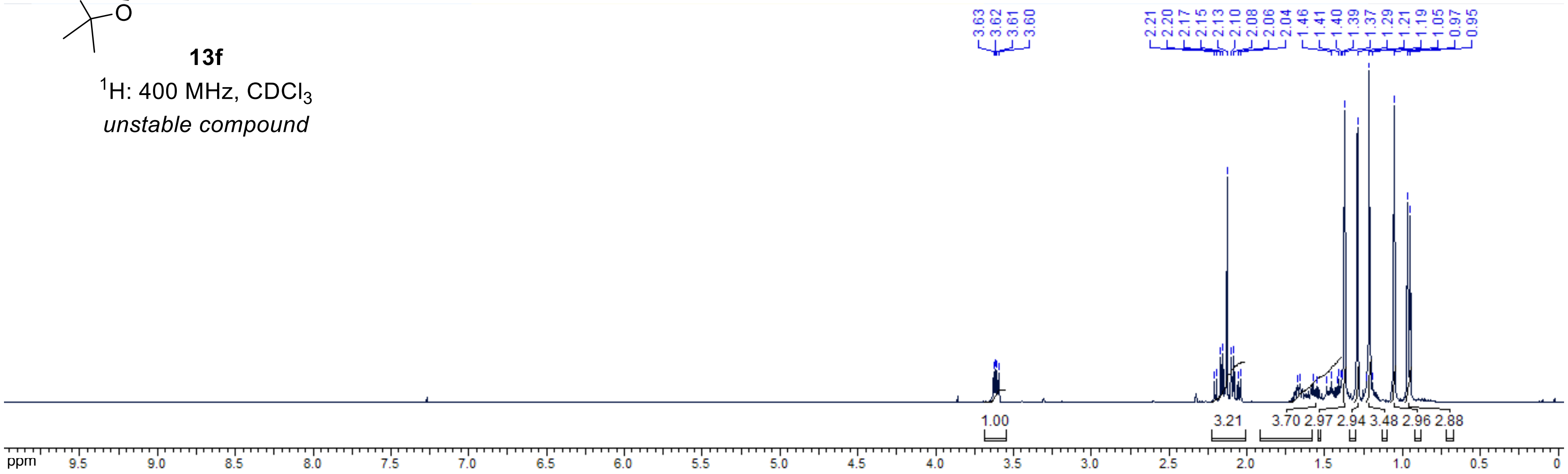

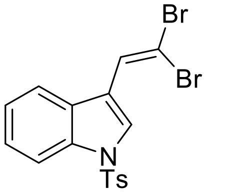

SD

^{1}H: 400 MHz, $CDCl_3$

^{13}C: 101 MHz, $CDCl_3$

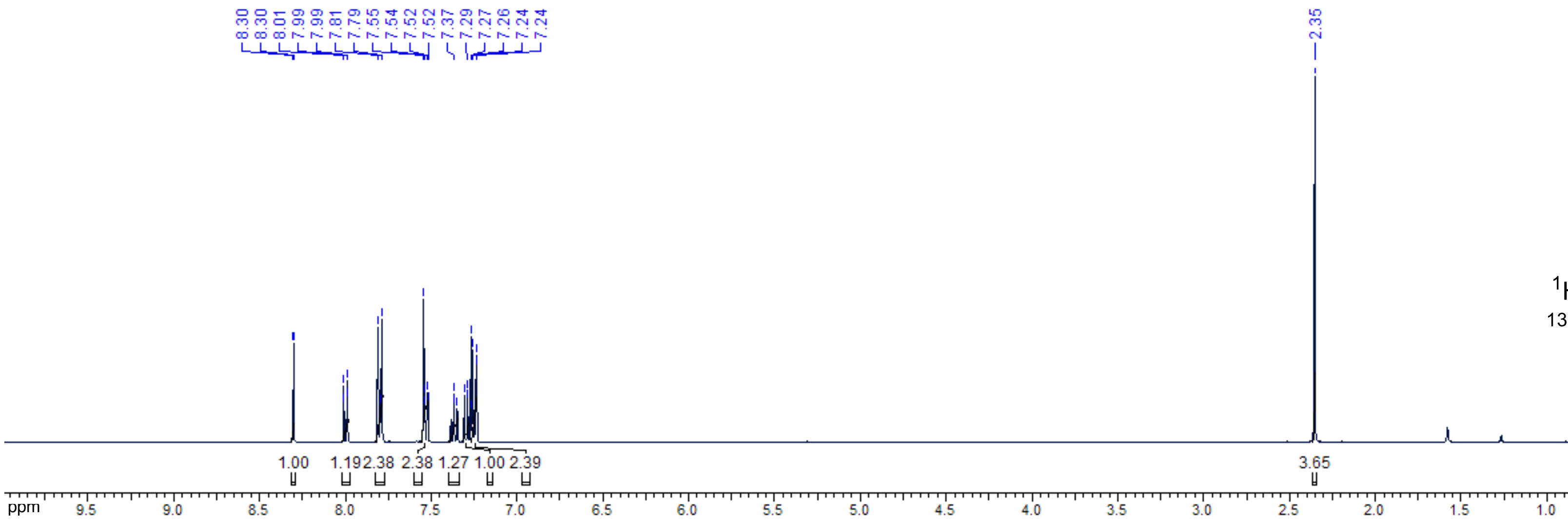

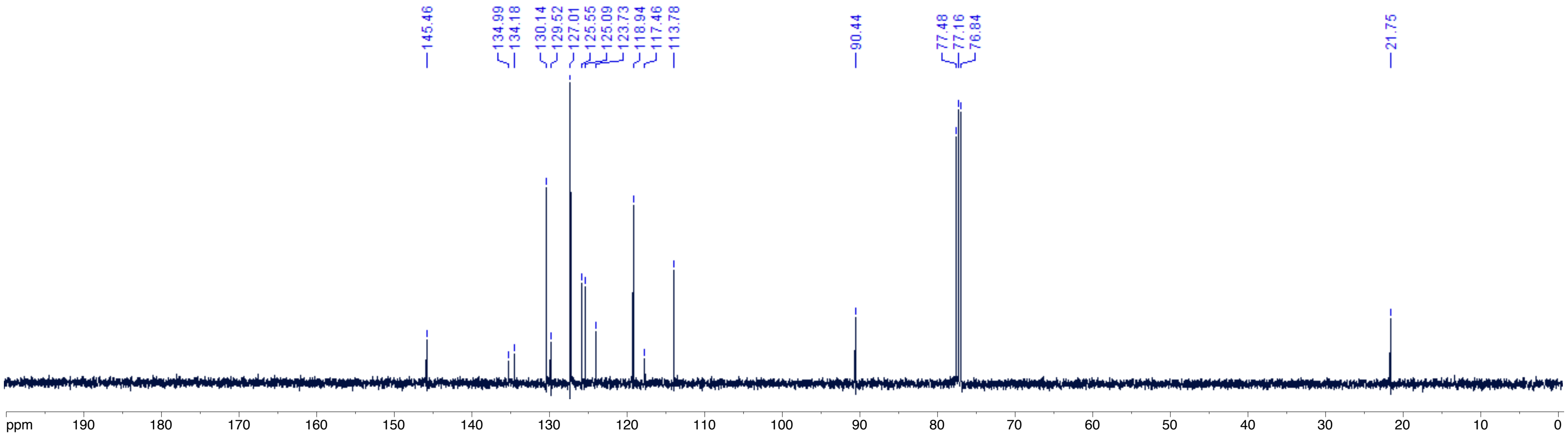

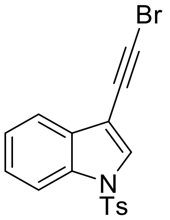

13g

^{1}H: 400 MHz, $CDCl_3$

^{13}C: 101 MHz, $CDCl_3$

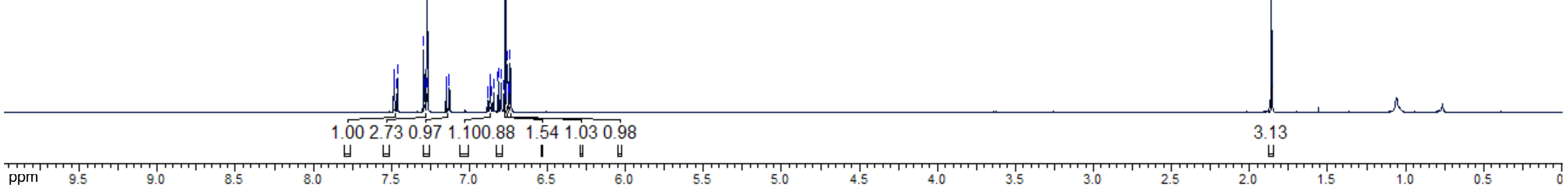

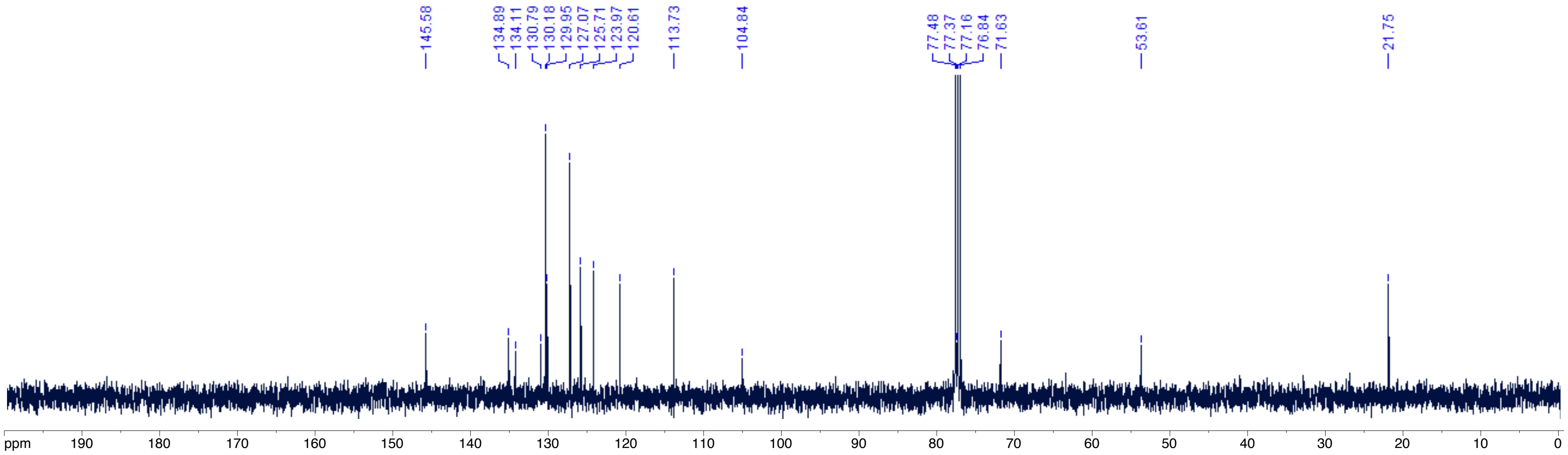

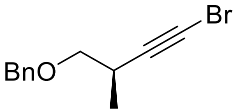

13h

^{1}H: 400 MHz, $CDCl_3$

^{13}C: 101 MHz, $CDCl_3$

unstable compound

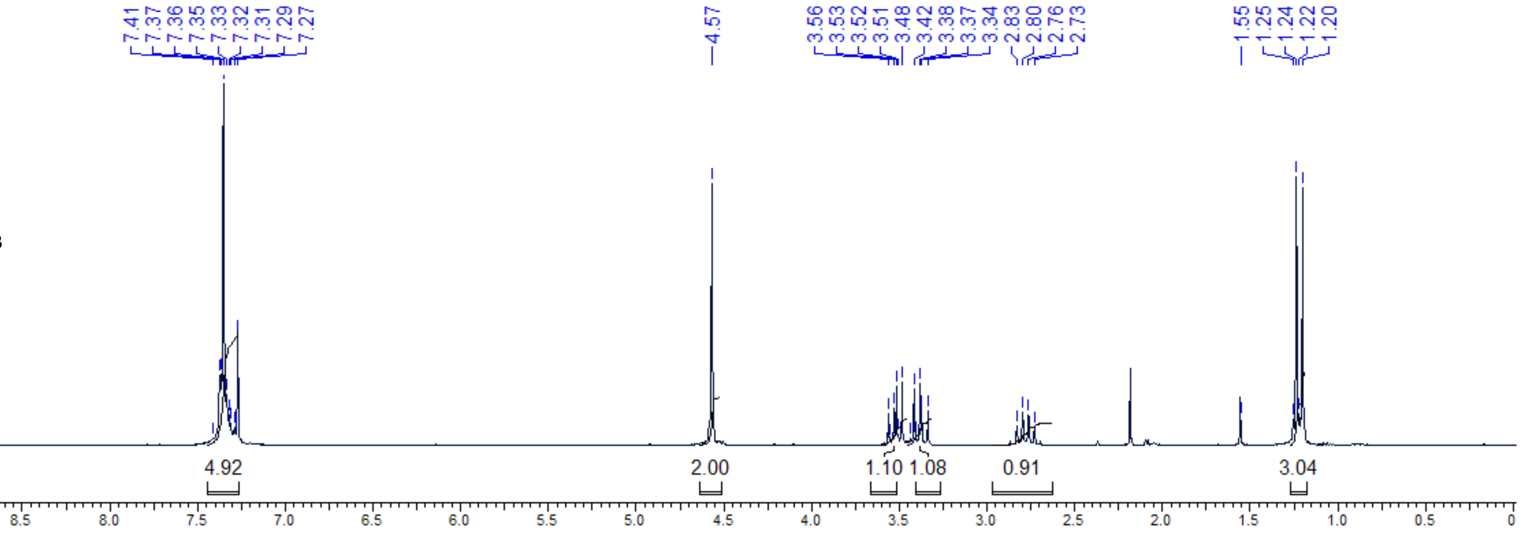

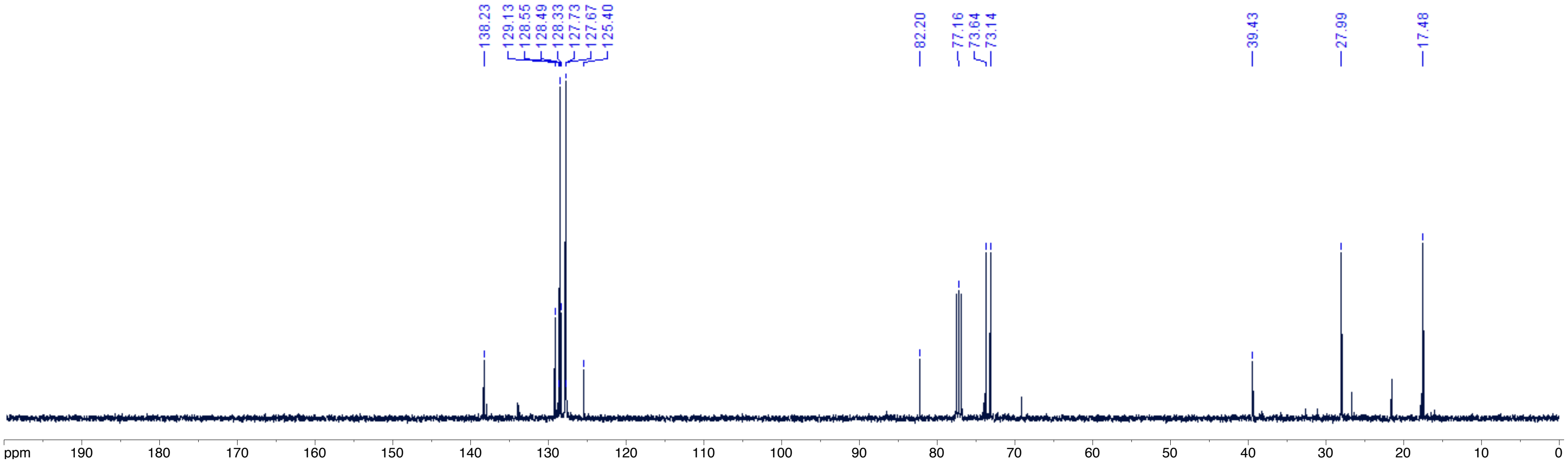

VT NMR – Stille cascade cyclization: isolation of intermediate triene 20 from NMR reaction of 3a and 4a

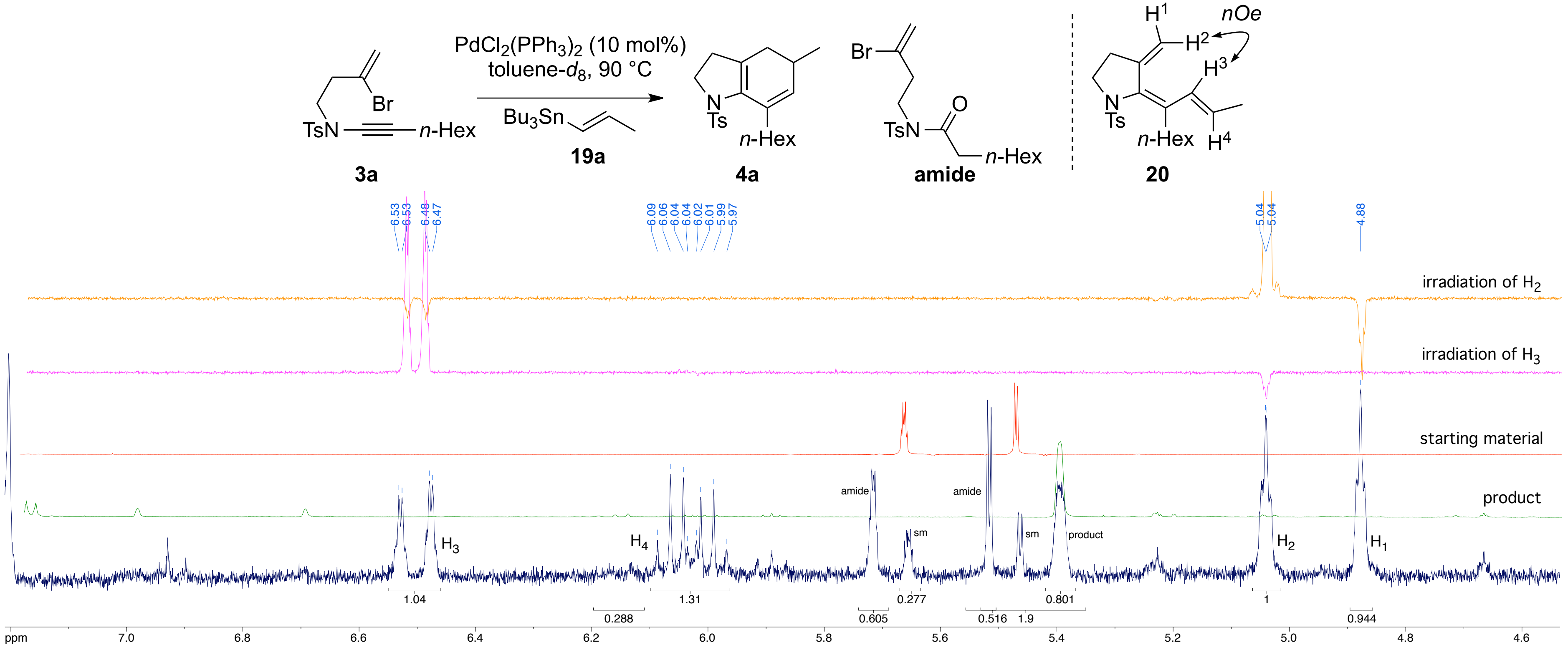

Blue spectrum indicates isolated mixture of triene intermediate (**20**), starting material **3a (red)**, product **4a (green)**, and **amide** (ynamide hydrolysis product) in a **1 : 0.23 : 0.8 : 0.5** ratio.

The assignment of these characteristic peaks is as follows: ^{1}H NMR (300 MHz; $CDCl_3$): δ 6.50 (1H, unresolved dq, J = 15.7 Hz, H3), 6.03 (1H, dq, J = 15.6, 6.8 Hz, H4), 5.04 (d, J = 0.3 Hz, H2), 4.88 (s, H1).

Irradiation of H2 (**orange**) shows enhancement at H3 and H1; Irradiation of H3 (**pink**) shows enhancement at H2.

Stille Cascade

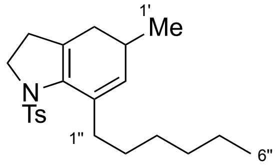

4a

^{1}H NMR (400 MHz, $CDCl_3$)

^{13}C NMR (125 MHz, $CDCl_3$)

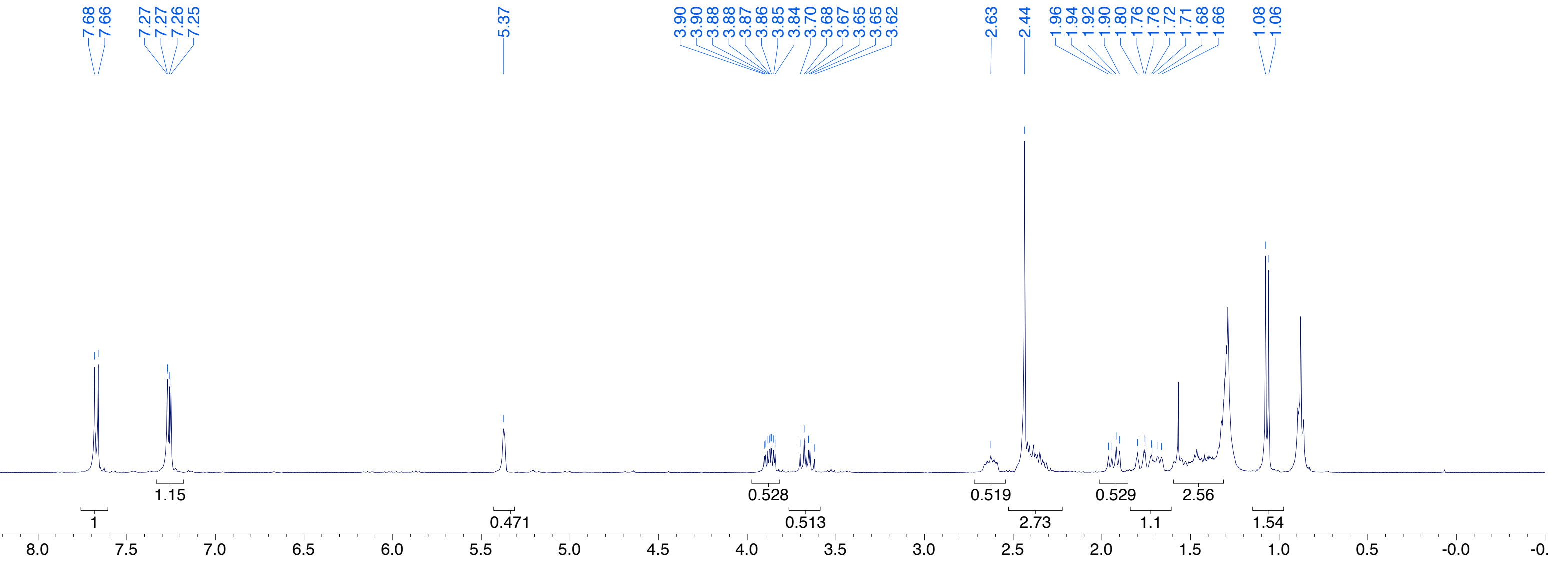

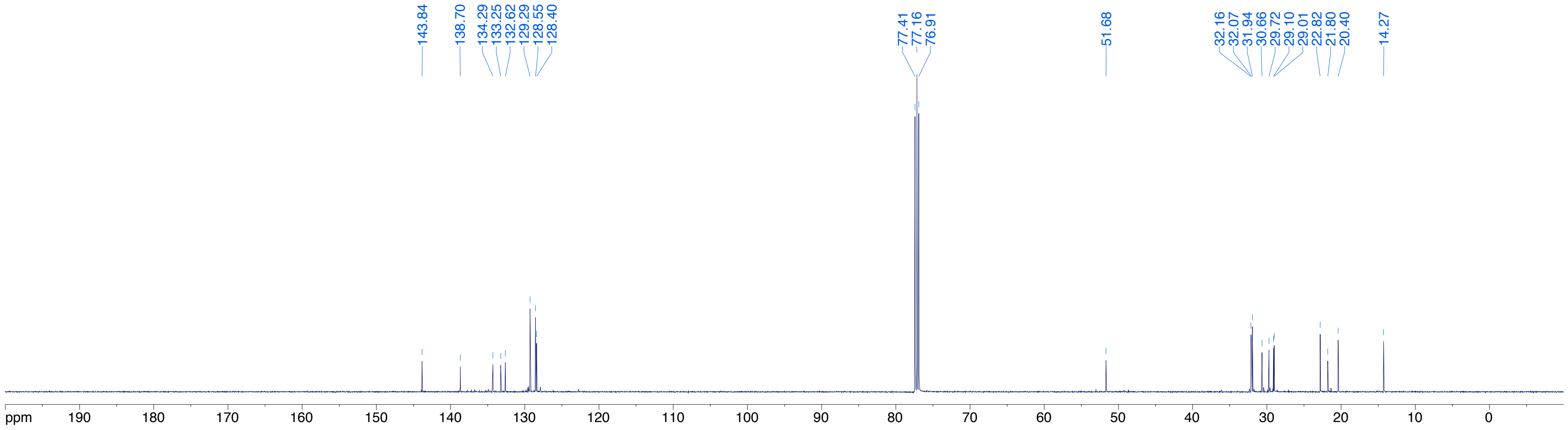

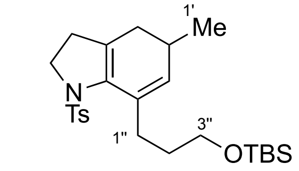

4b

^{1}H NMR (500 MHz, $CDCl_3$)

^{13}C NMR (125 MHz, $CDCl_3$)

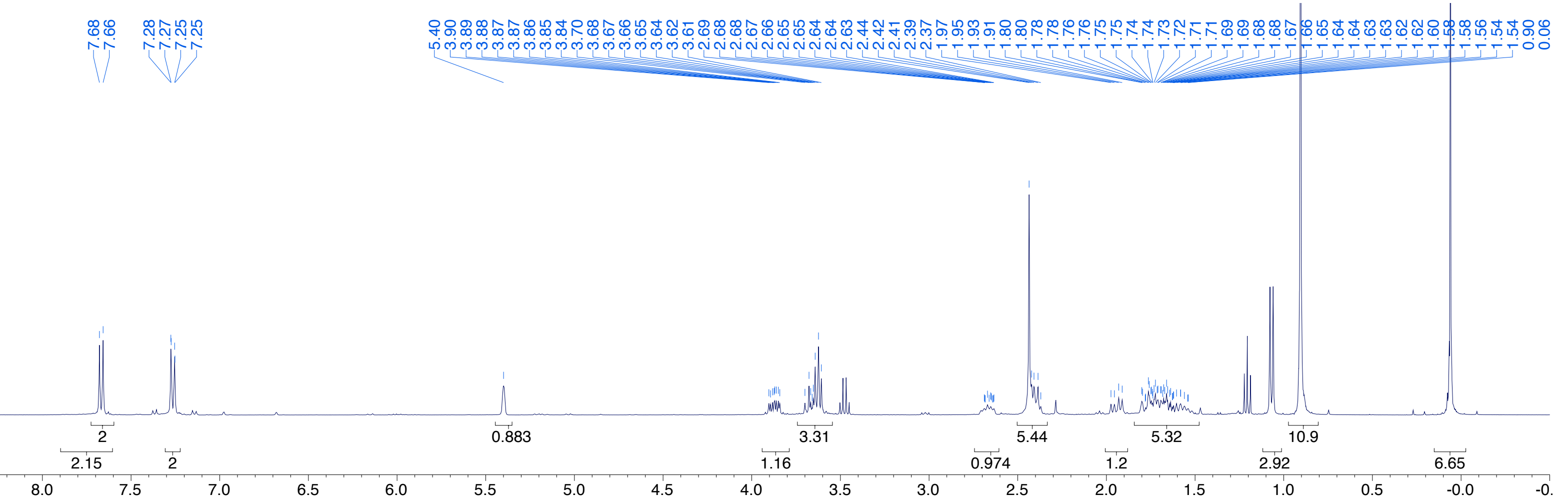

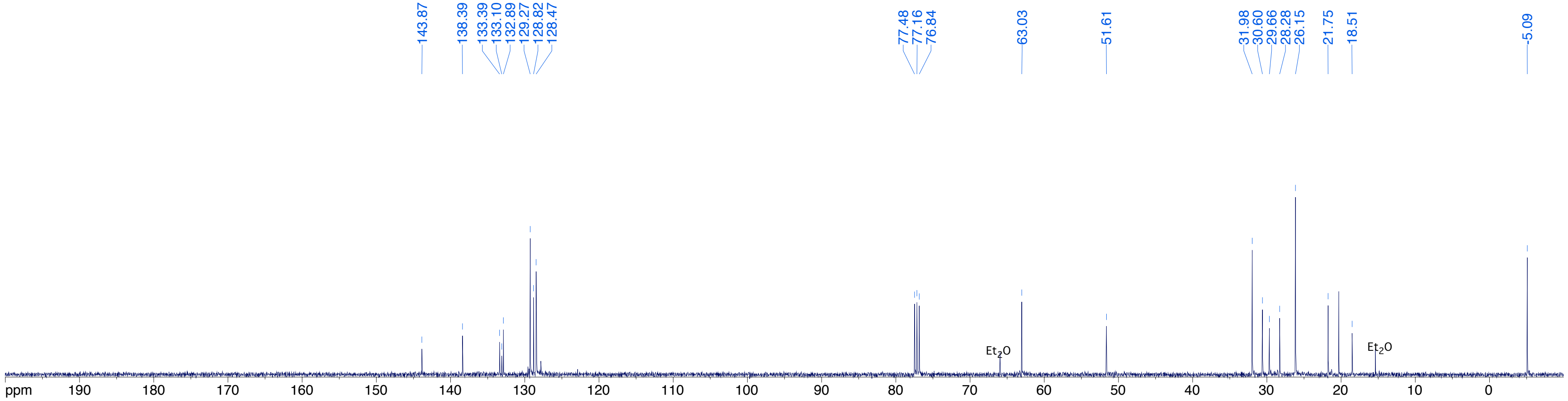

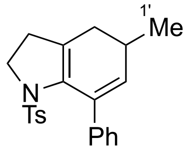

4c

^{1}H NMR (400 MHz, $CDCl_3$)

^{13}C NMR (100 MHz, $CDCl_3$)

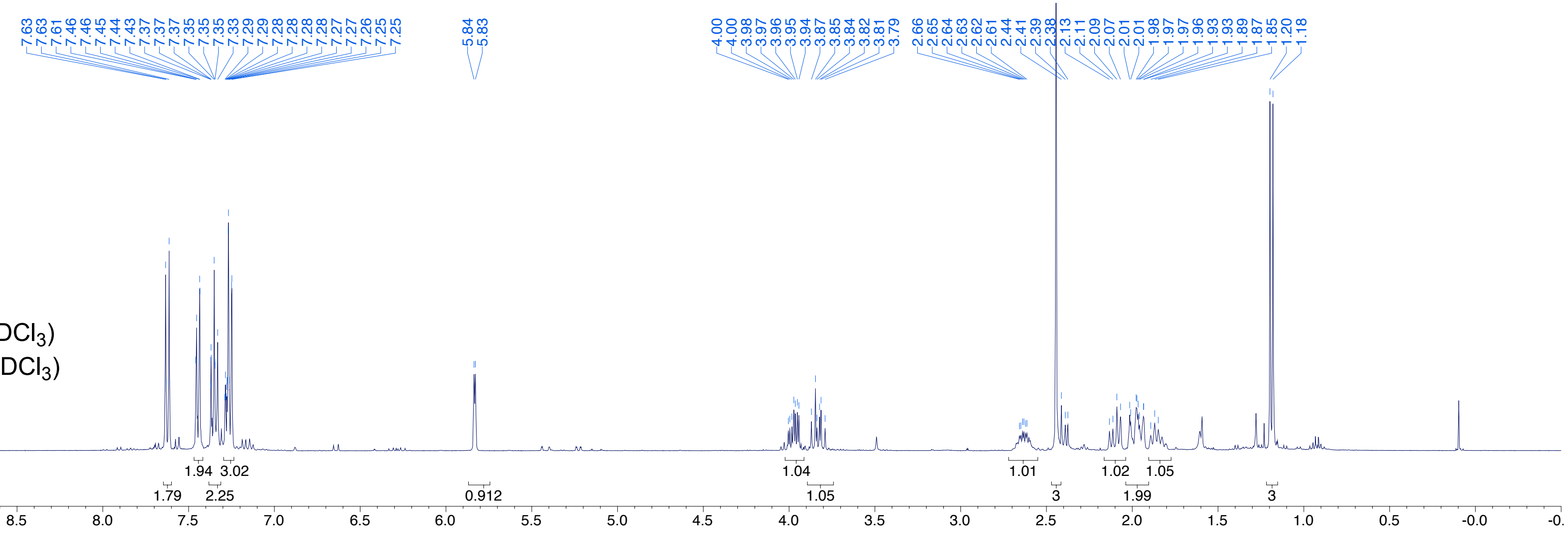

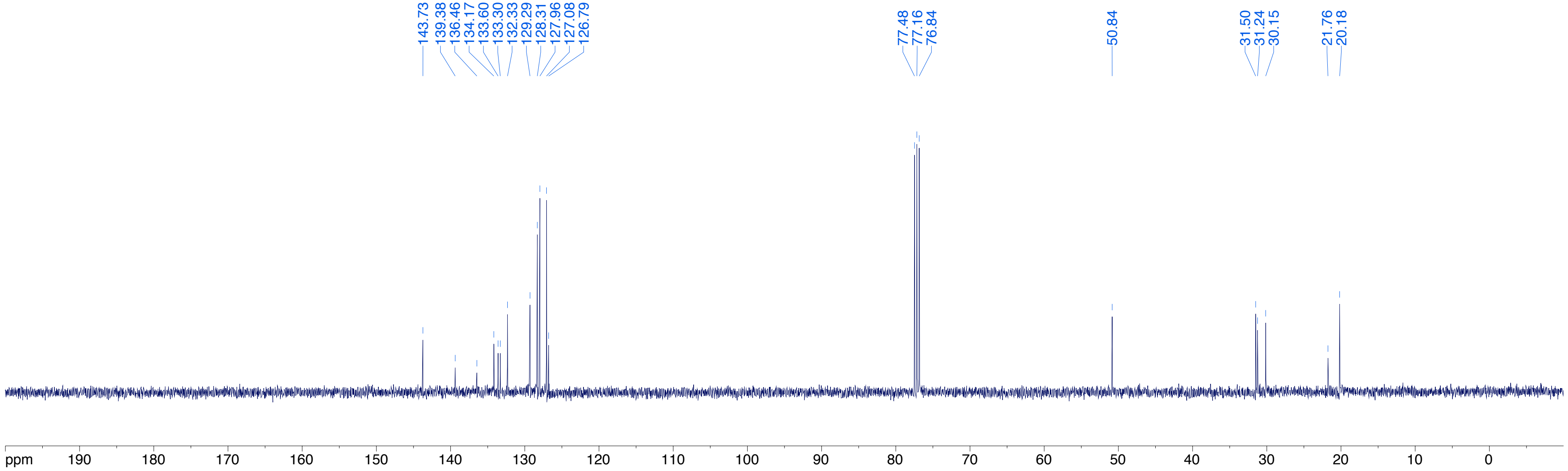

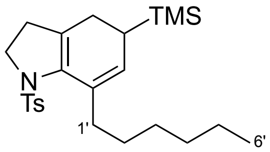

4f

^{1}H NMR (500 MHz, $CDCl_3$)

^{13}C NMR (125 MHz, $CDCl_3$)

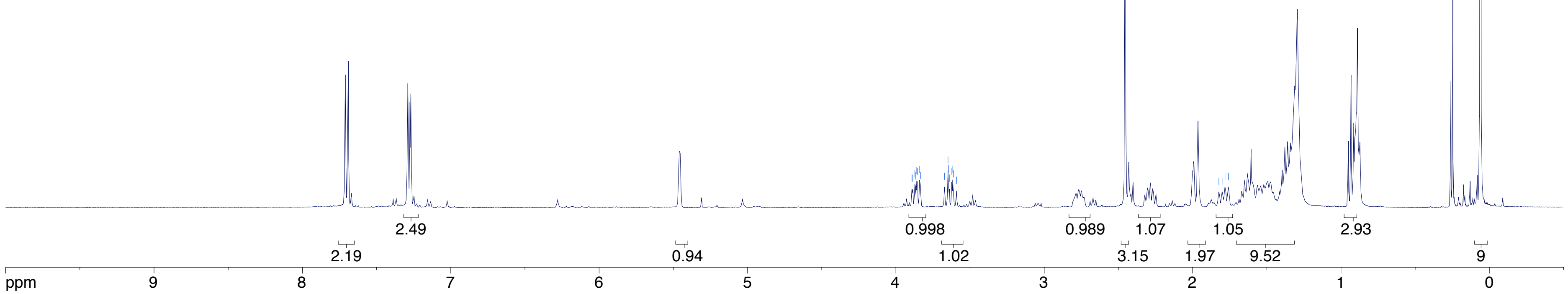

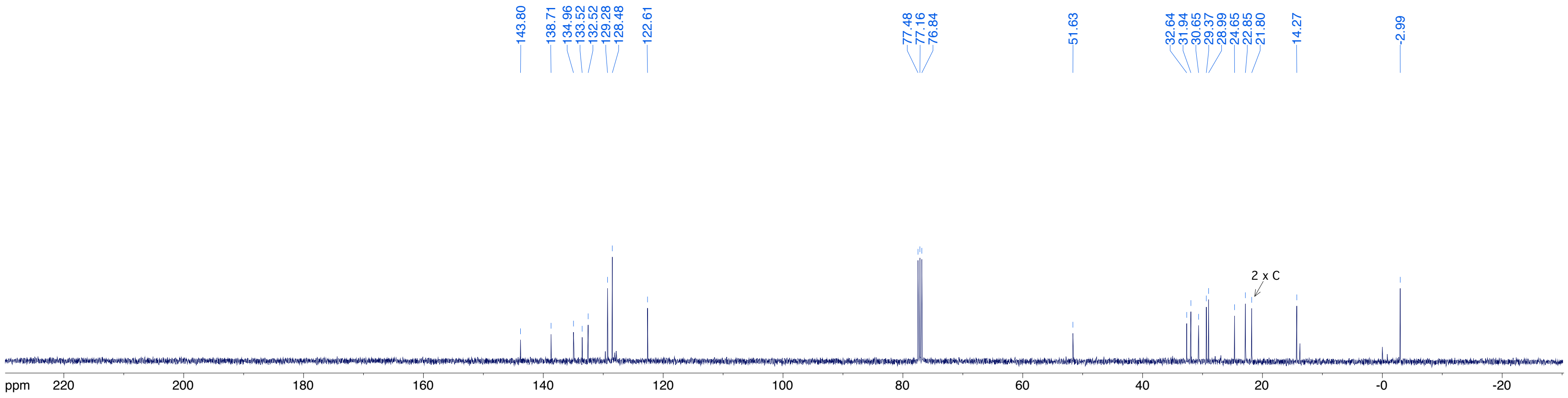

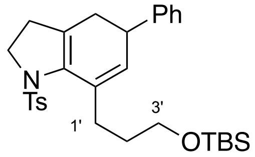

4g

^{1}H NMR (500 MHz, $CDCl_3$)

^{13}C NMR (125 MHz, $CDCl_3$)

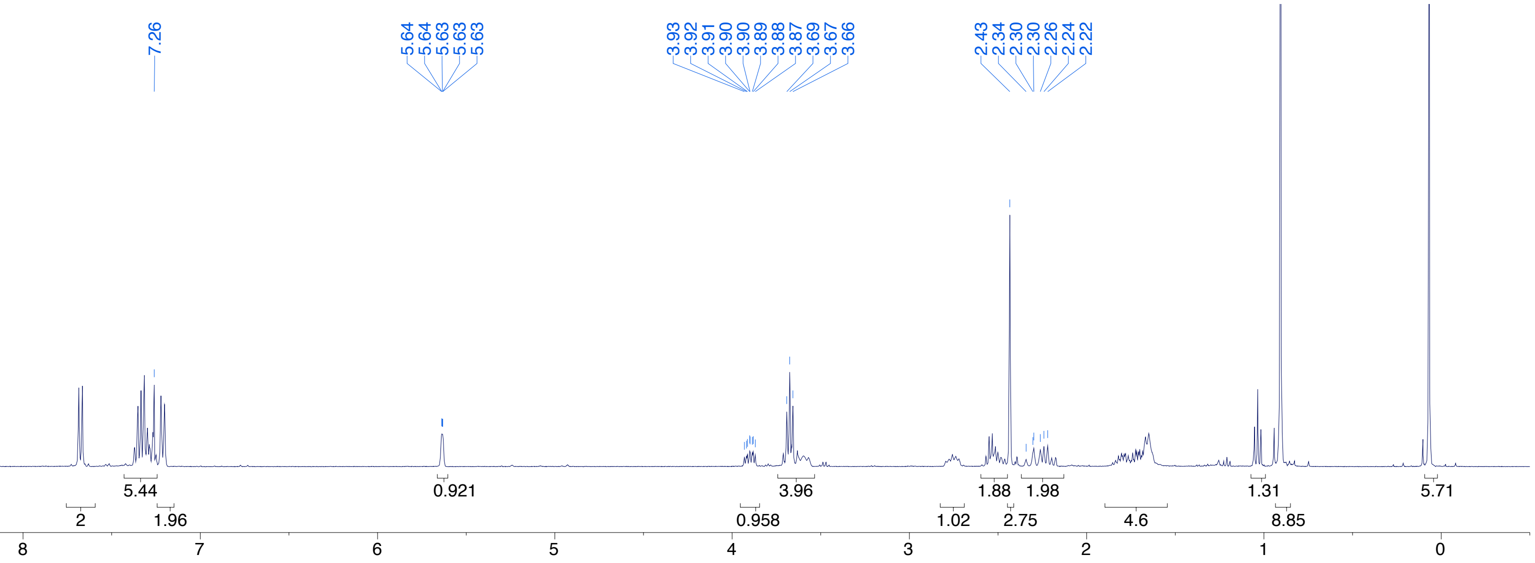

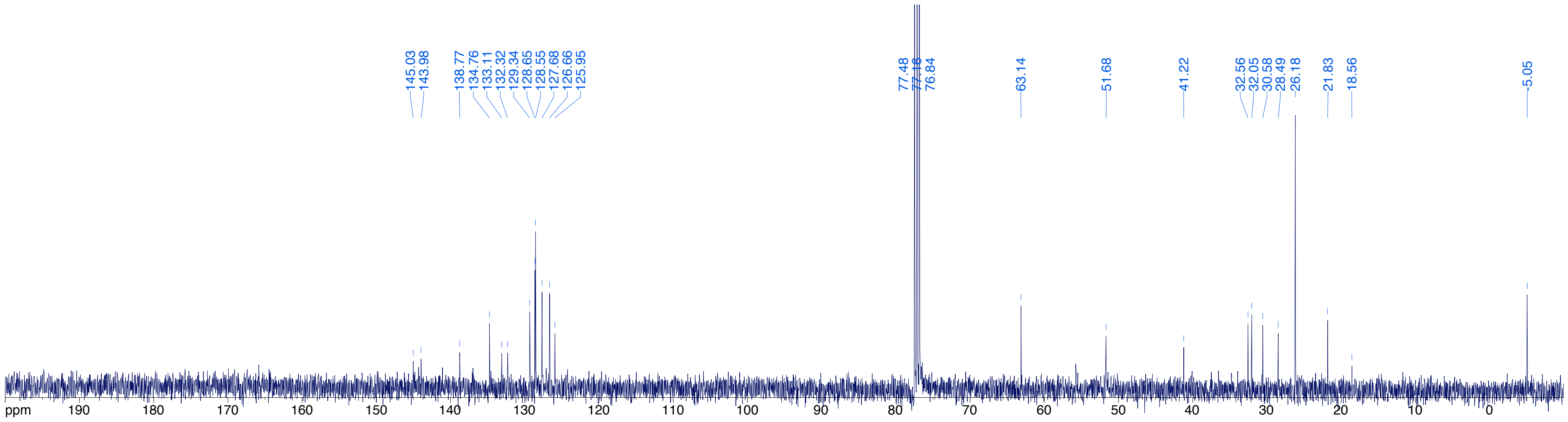

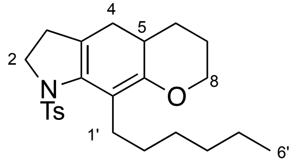

4h

^1H NMR (500 MHz, $CDCl_3$)

^{13}C NMR (125 MHz, $CDCl_3$)

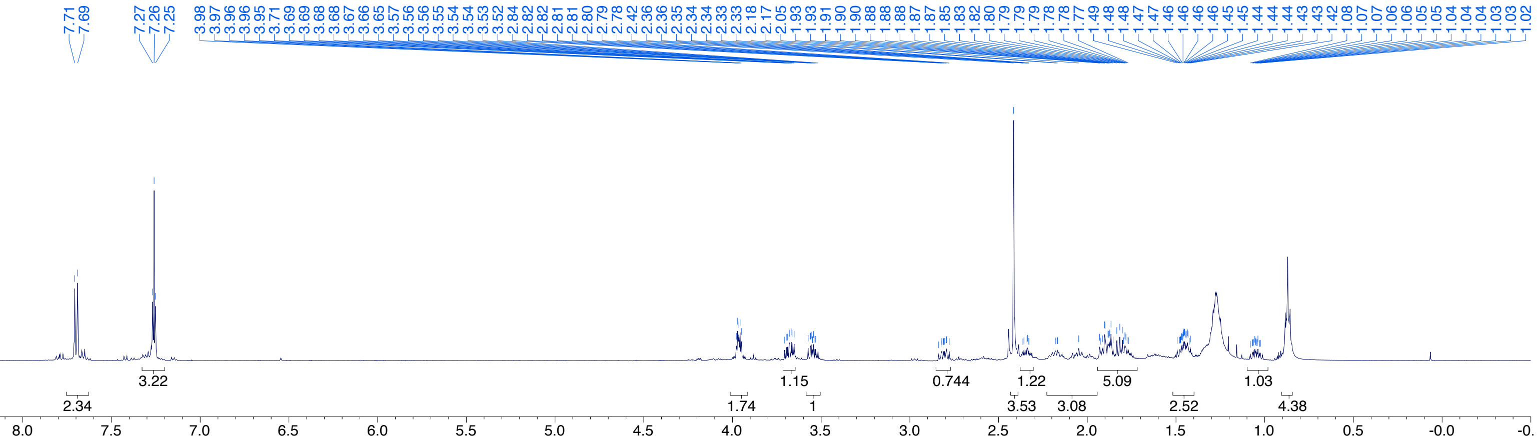

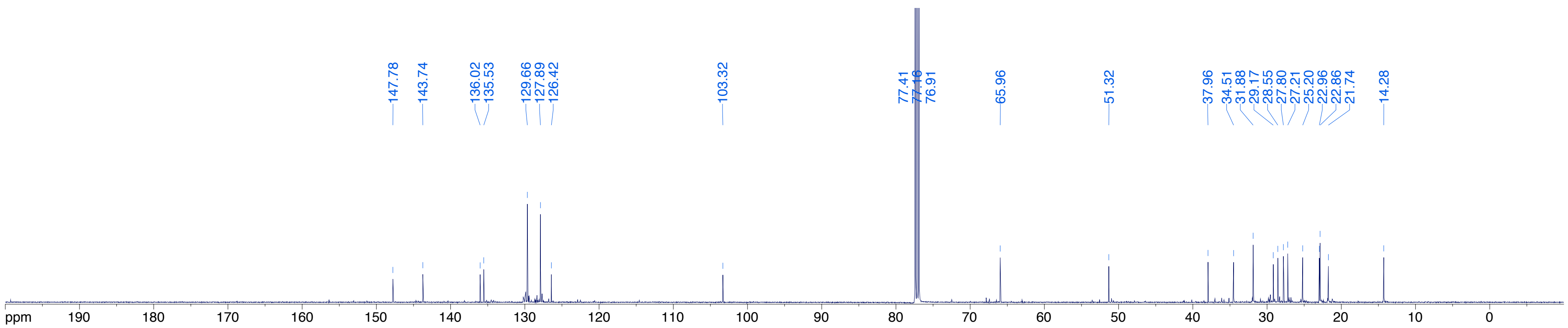

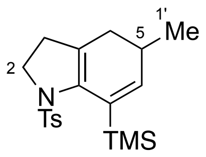

4i

^{1}H NMR (500 MHz, $CDCl_3$)

^{13}C NMR (125 MHz, $CDCl_3$)

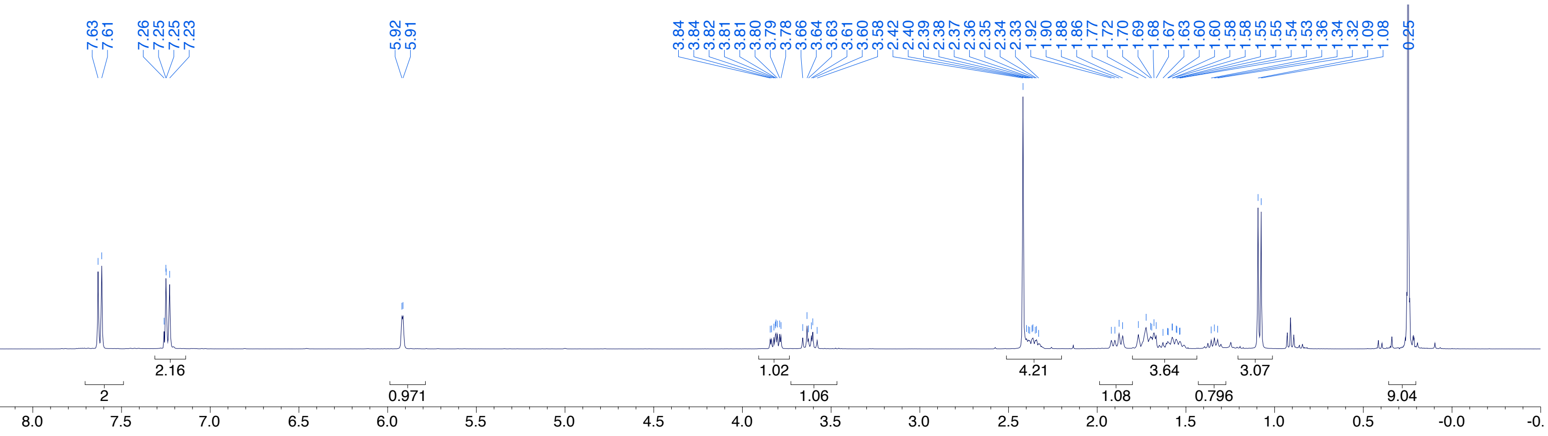

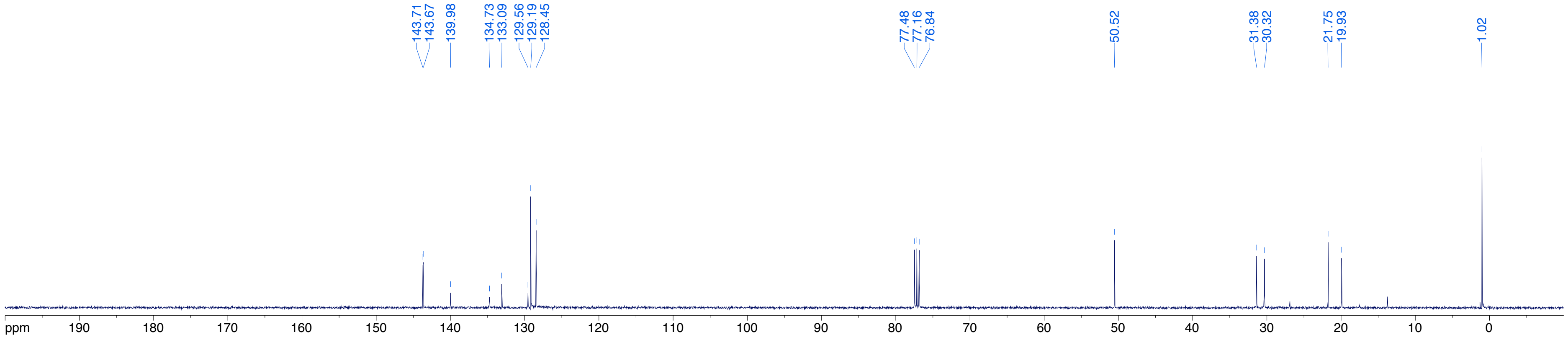

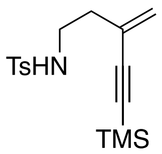

21a

^{1}H NMR (500 MHz, $CDCl_3$)

^{13}C NMR (125 MHz, $CDCl_3$)

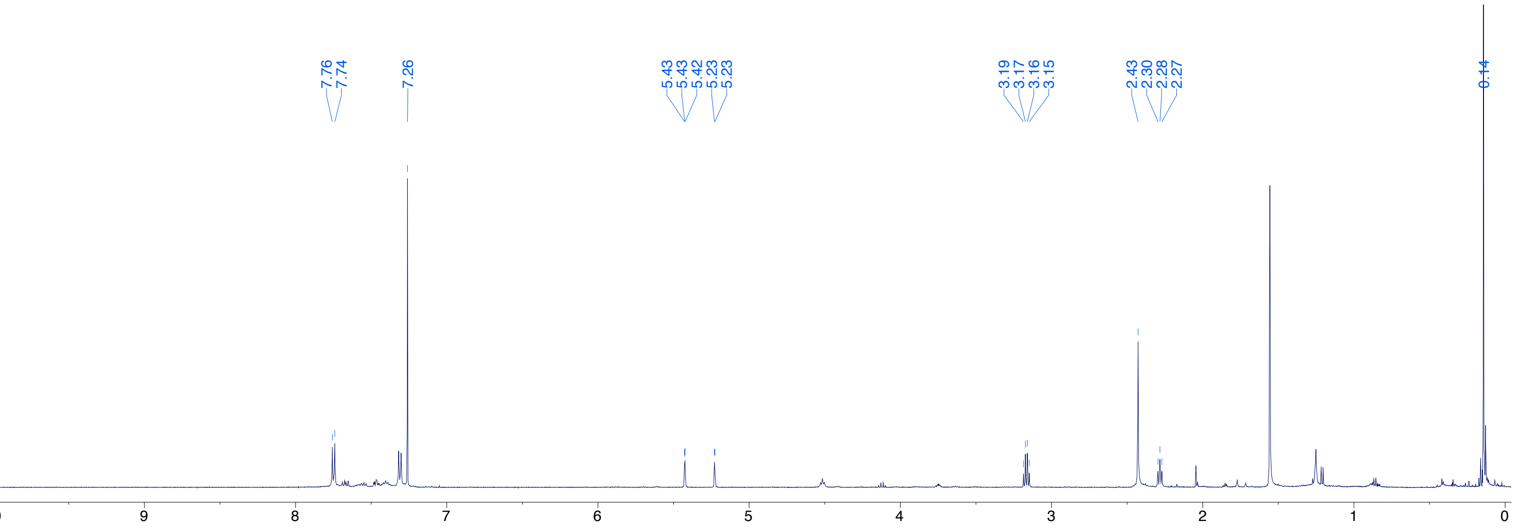

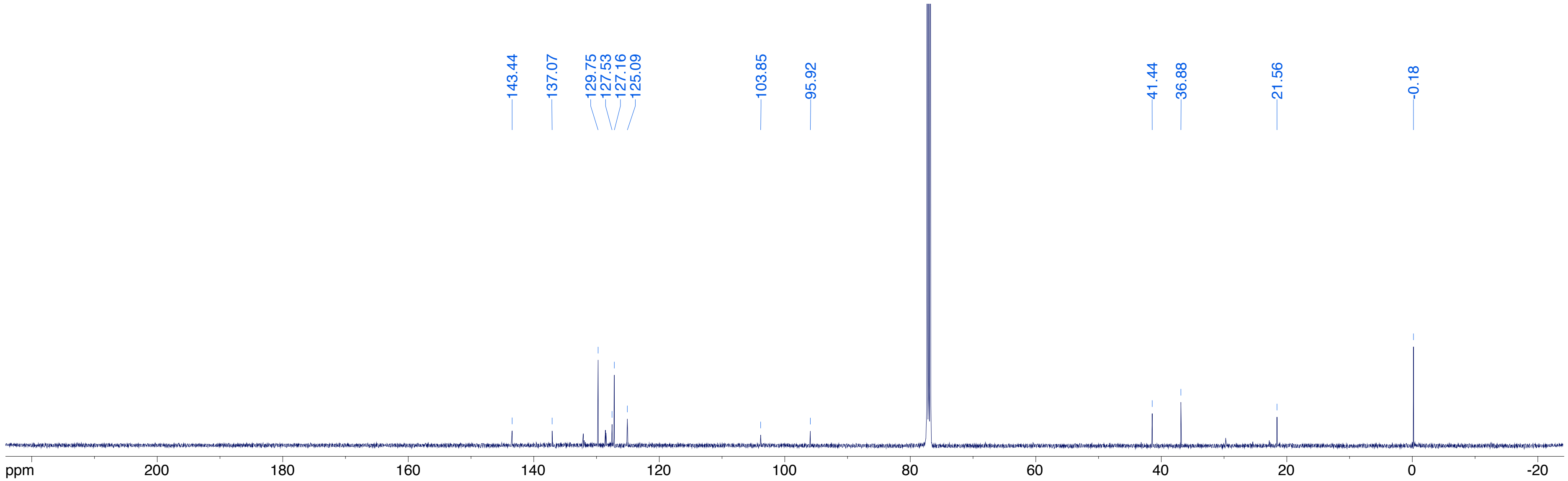

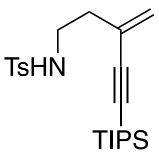

21b

^{1}H NMR (500 MHz, $CDCl_3$)

^{13}C NMR (125 MHz, $CDCl_3$)

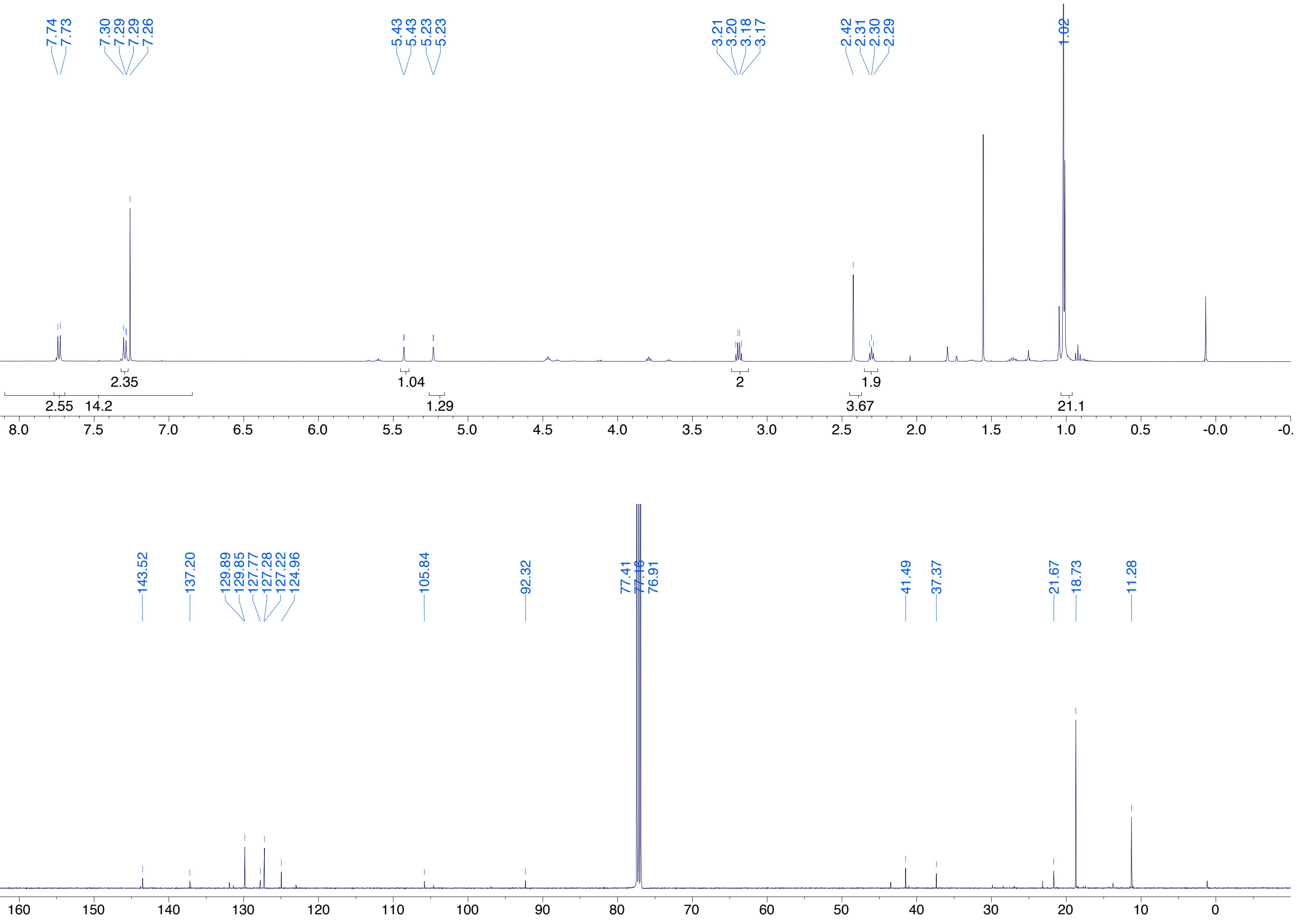

Suzuki-Molander Cascades: Data for compounds **4d**, **4e**, **4j**, **4k**, **4p**, **4ae** has been reported previously.[6]

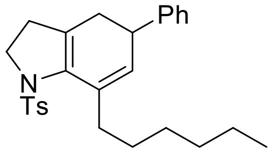

4d

^{1}H: 400 MHz, $CDCl_3$

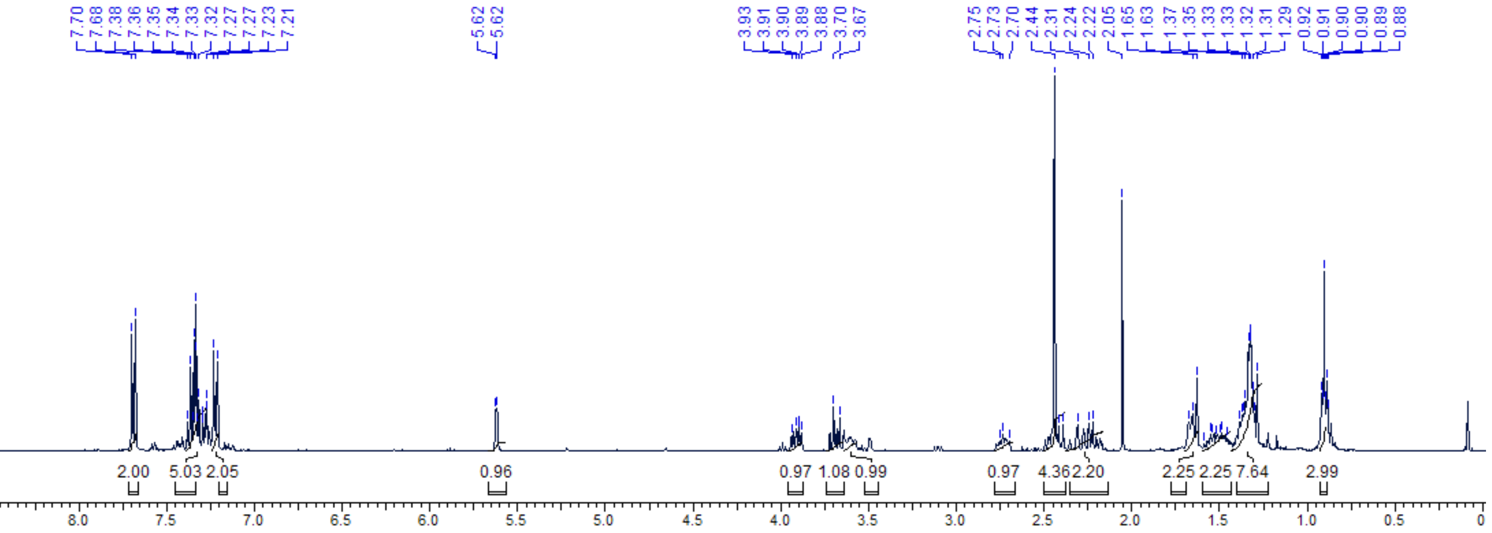

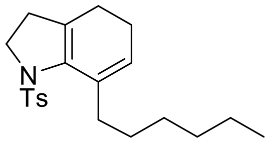

4e

^{1}H: 400 MHz, $CDCl_3$

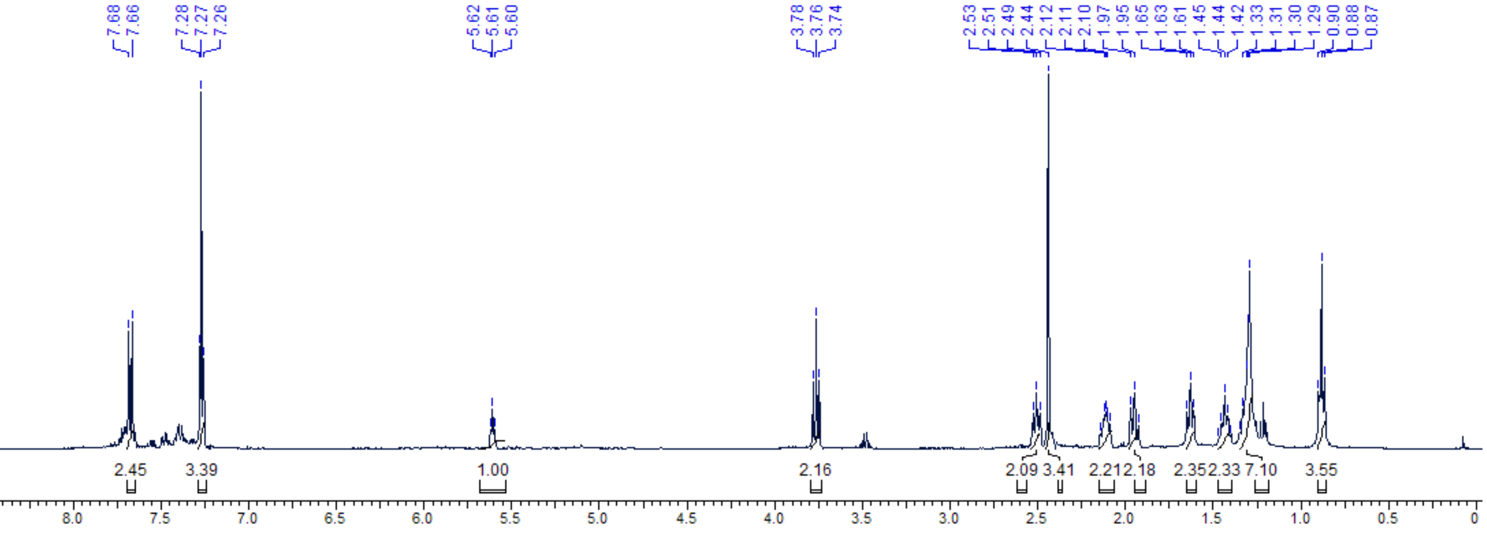

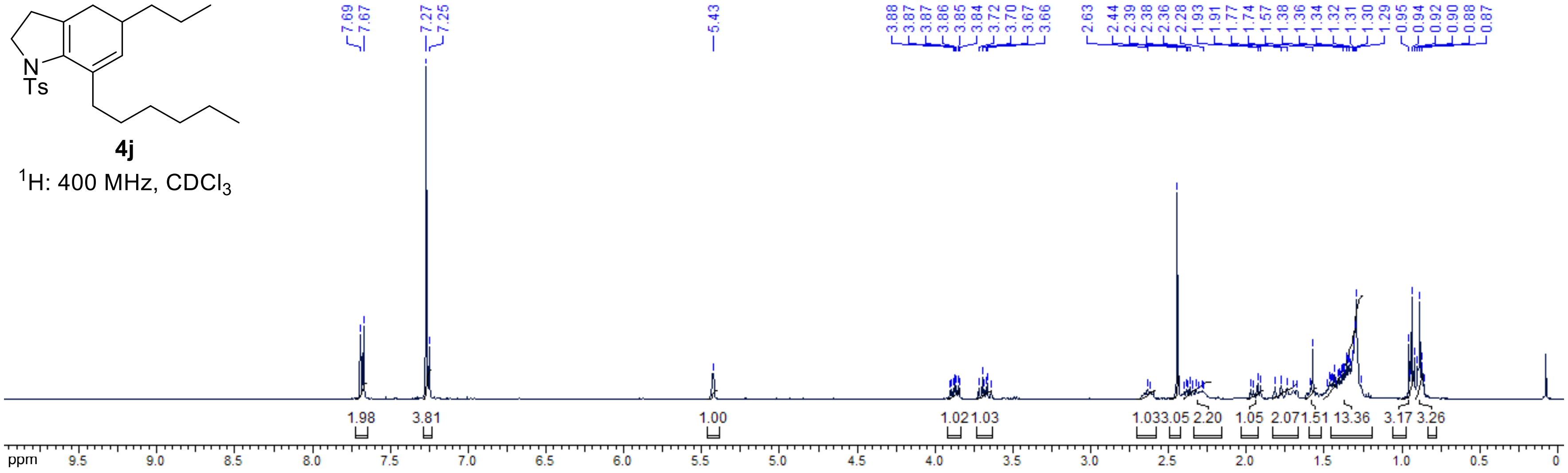

4j

^{1}H: 400 MHz, $CDCl_3$

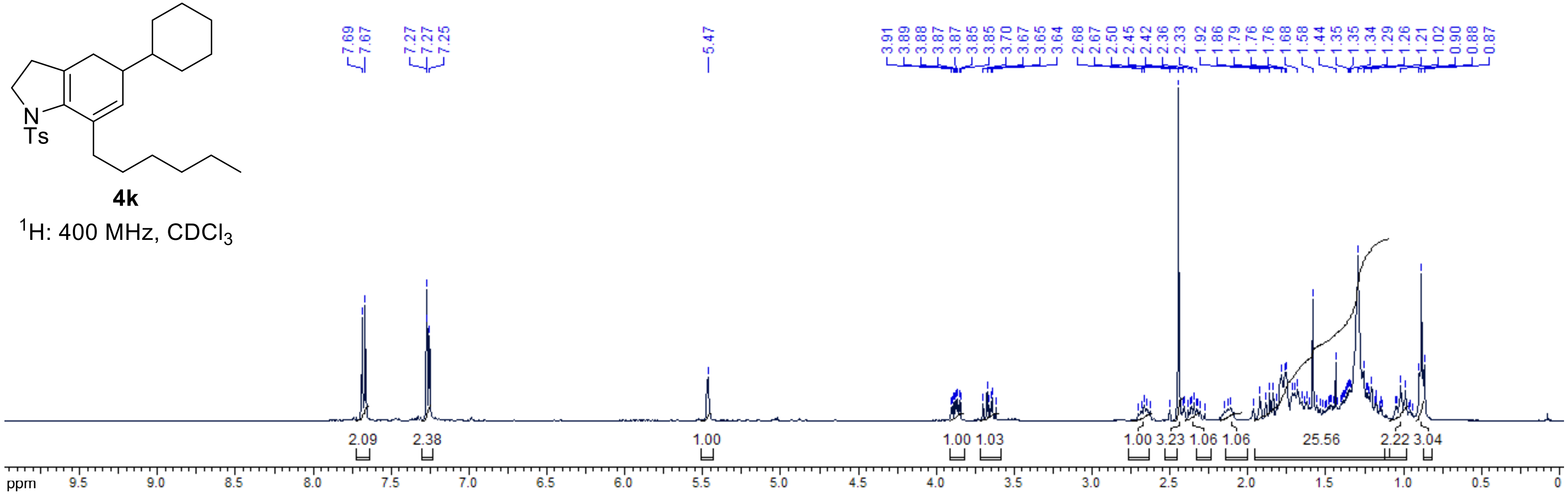

4k

^{1}H: 400 MHz, $CDCl_3$

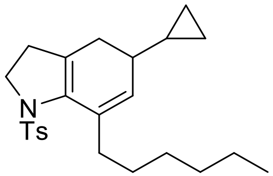

4p

^{1}H: 400 MHz, $CDCl_3$

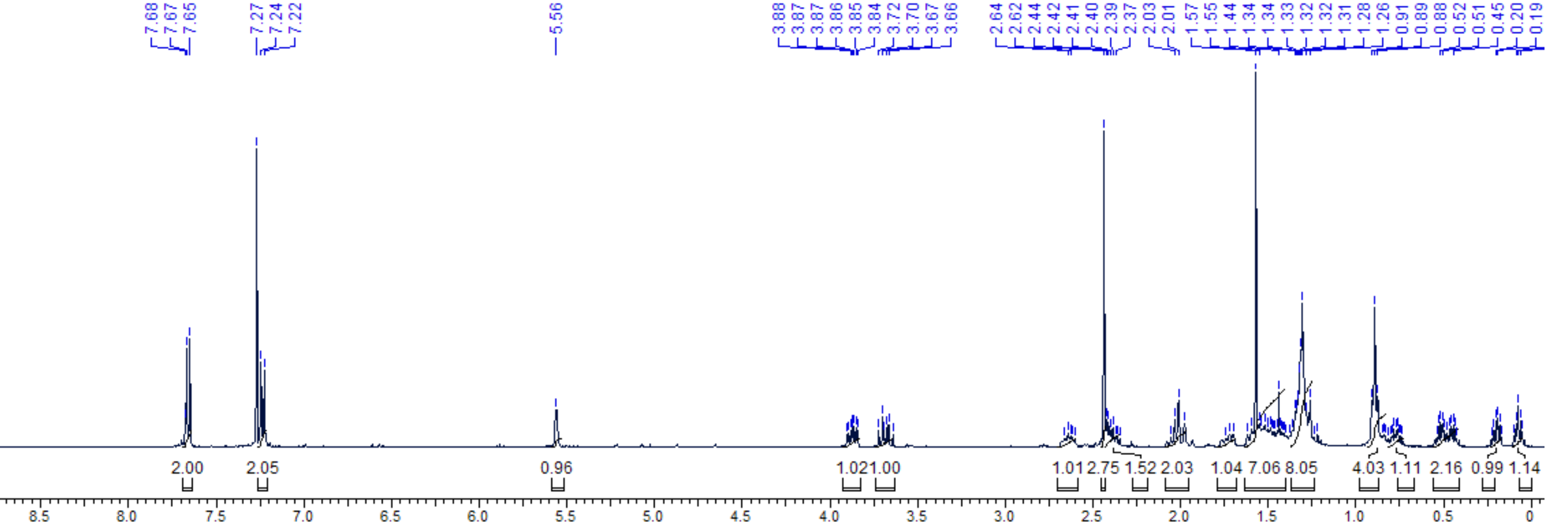

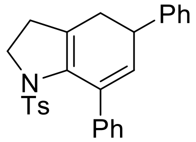

4ae

^{1}H: 400 MHz, $CDCl_3$

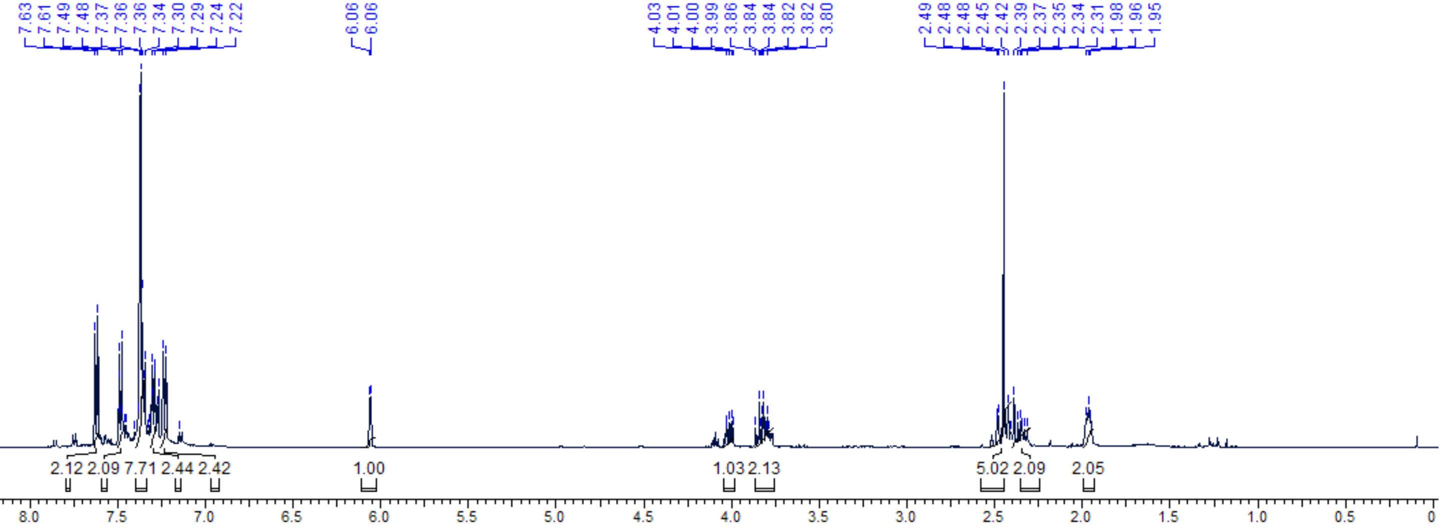

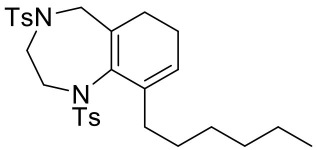

4af

^{1}H: 500 MHz, C_6D_6

^{13}C: 125 MHz, C_6D_6

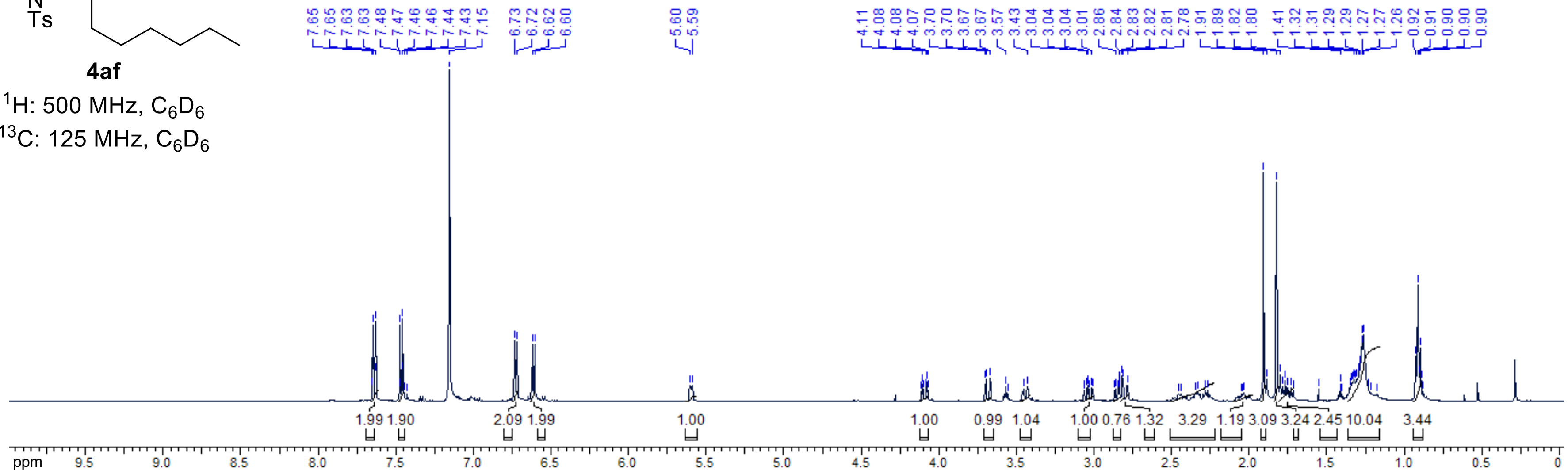

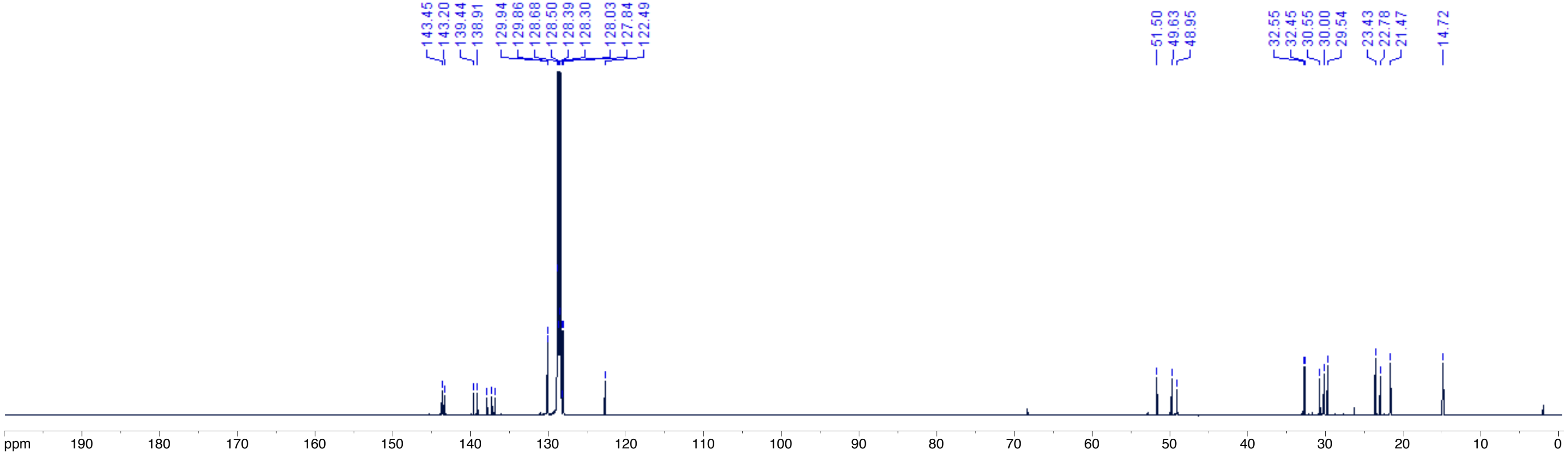

Formal 4-*endo*-trig cyclization product

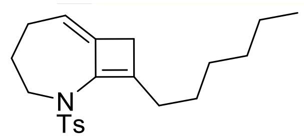

8a

^{1}H: 500 MHz, $CDCl_3$

^{13}C: 125 MHz, $CDCl_2$

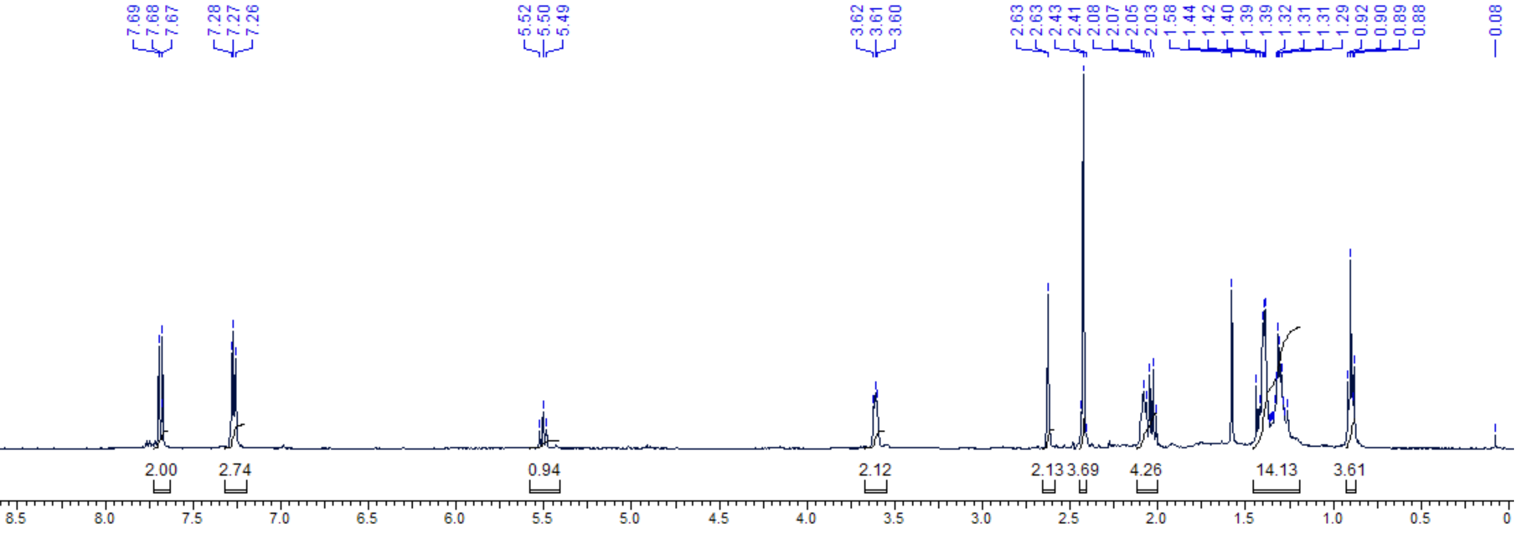

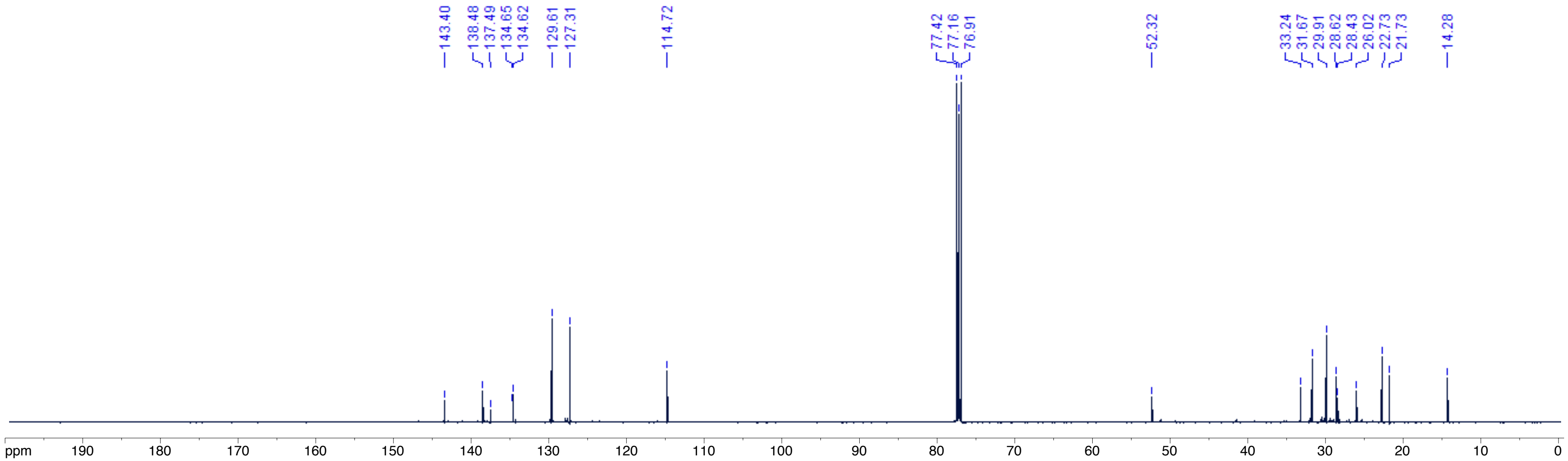

Ynhydrazides

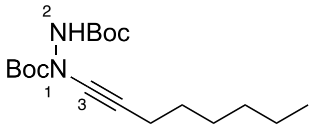

31
^{1}H: 500 MHz, $CDCl_3$
^{13}C: 125 MHz, $CDCl_3$

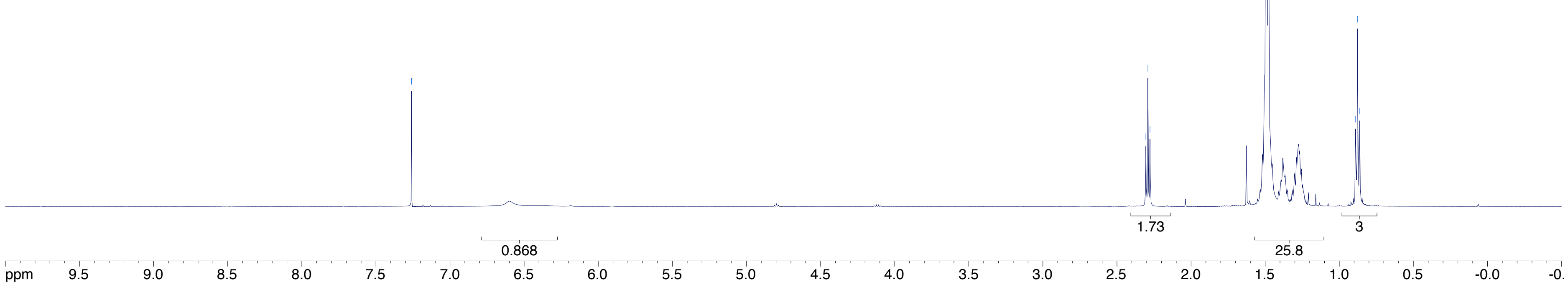

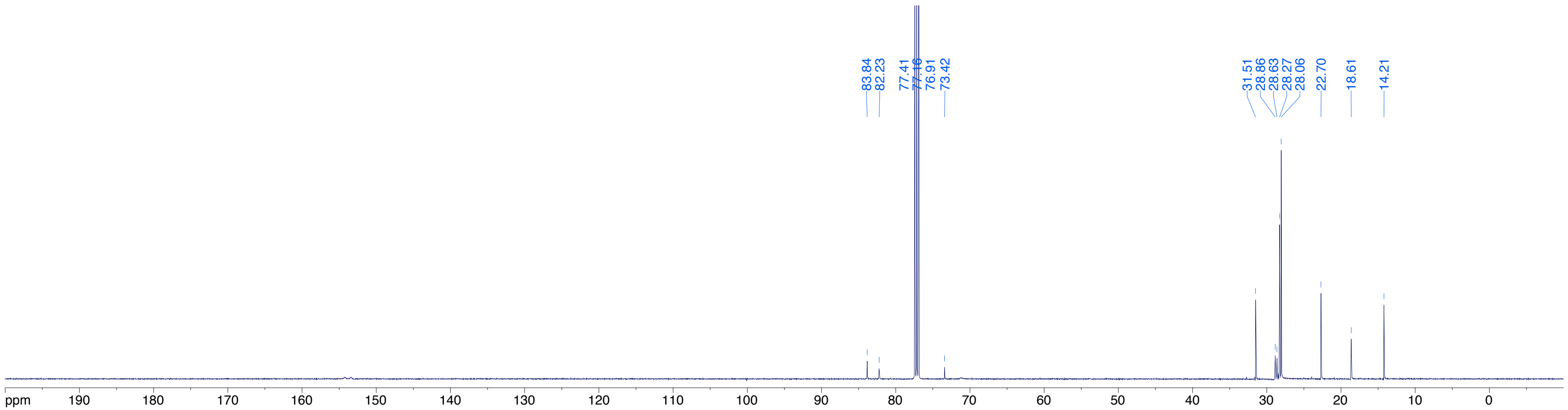

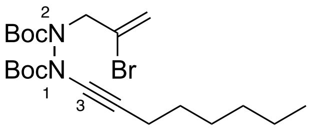

32a

^{1}H: 500 MHz, $CDCl_3$

^{13}C: 125 MHz, $CDCl_3$

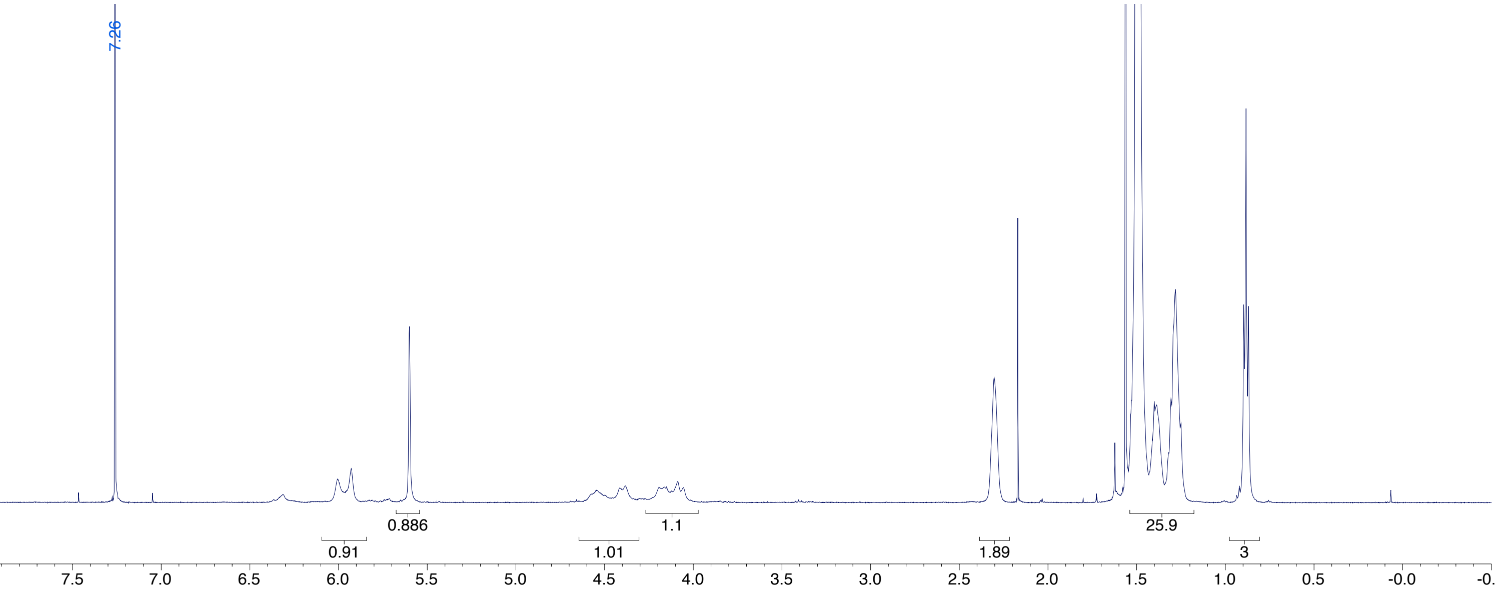

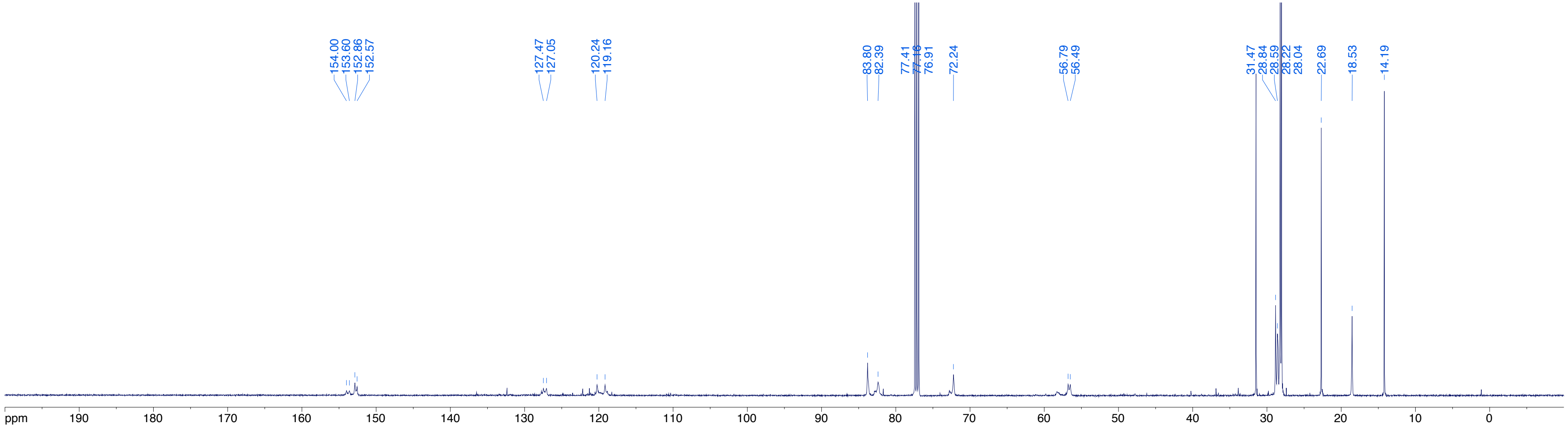

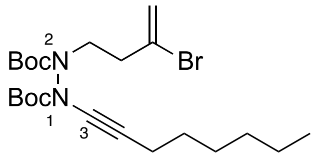

32b

^{1}H: 500 MHz, $CDCl_3$

^{13}C: 125 MHz, $CDCl_3$

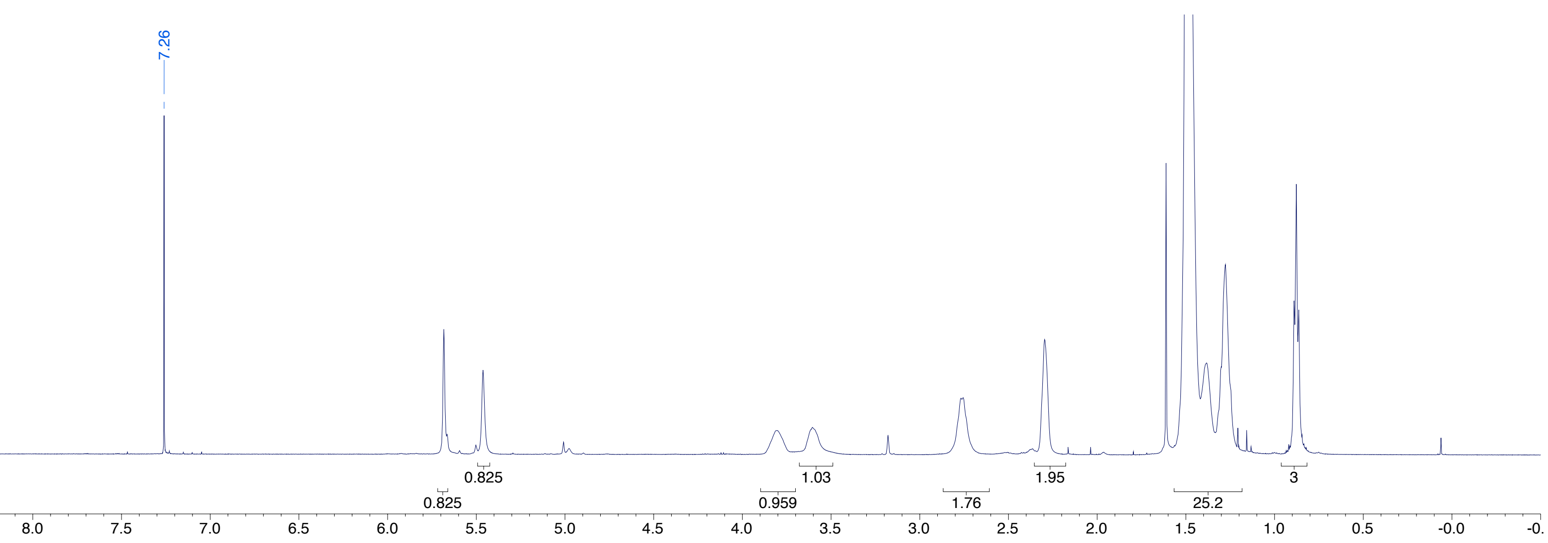

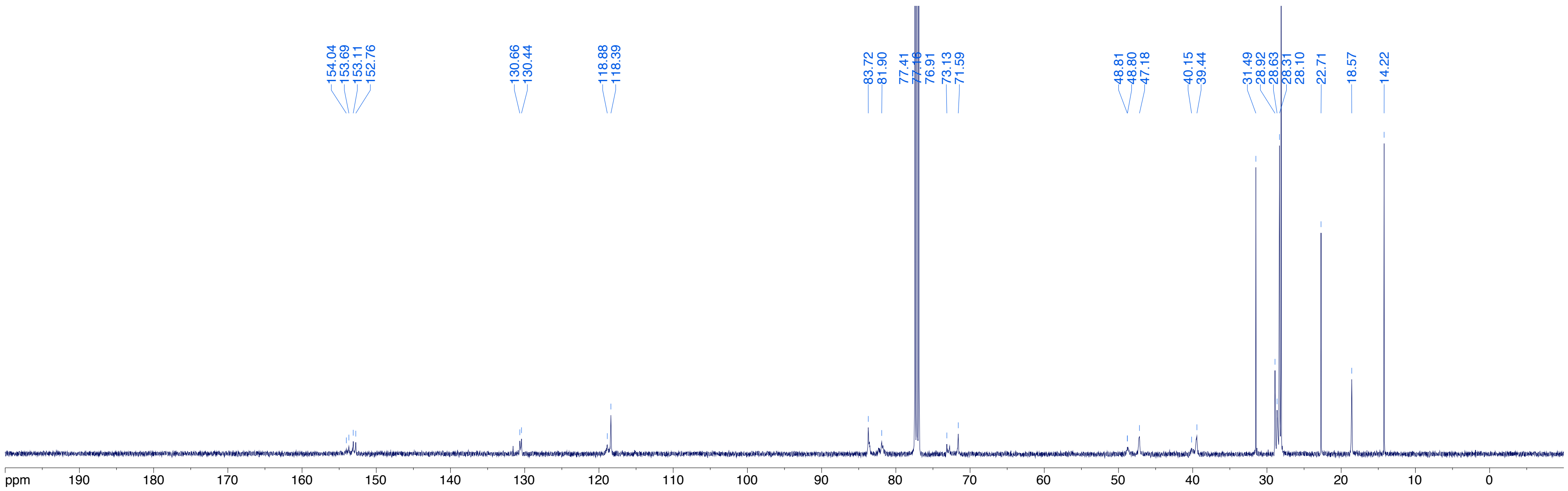

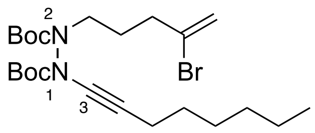

32c

^{1}H: 500 MHz, $CDCl_3$

^{13}C: 125 MHz, $CDCl_3$

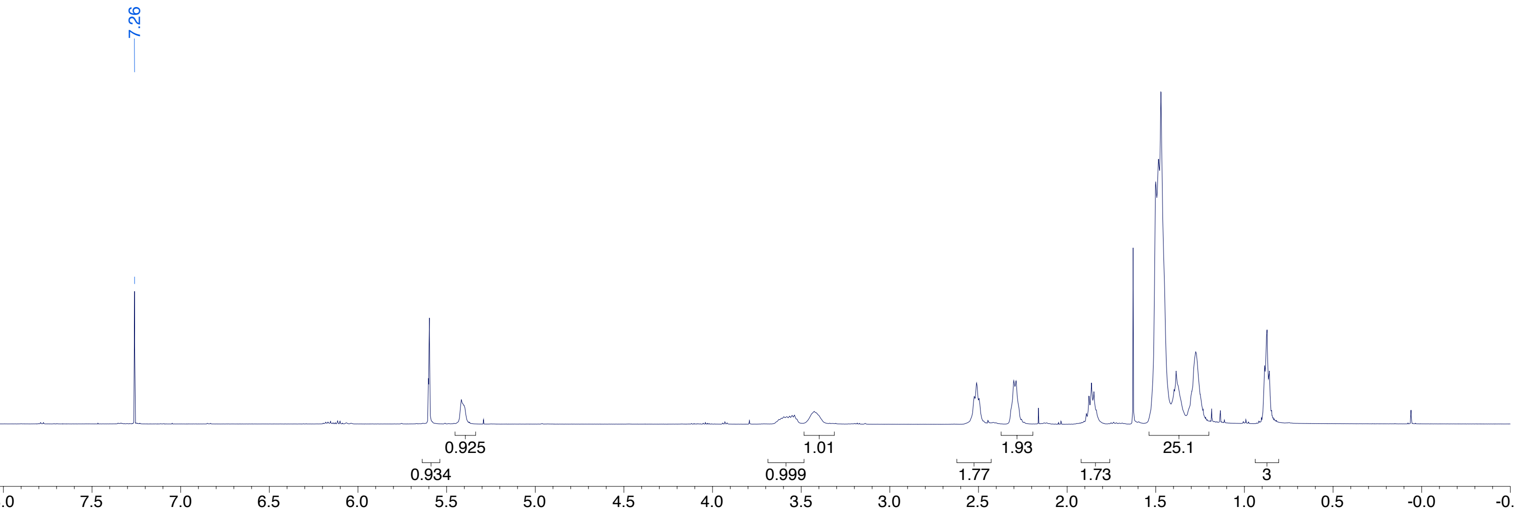

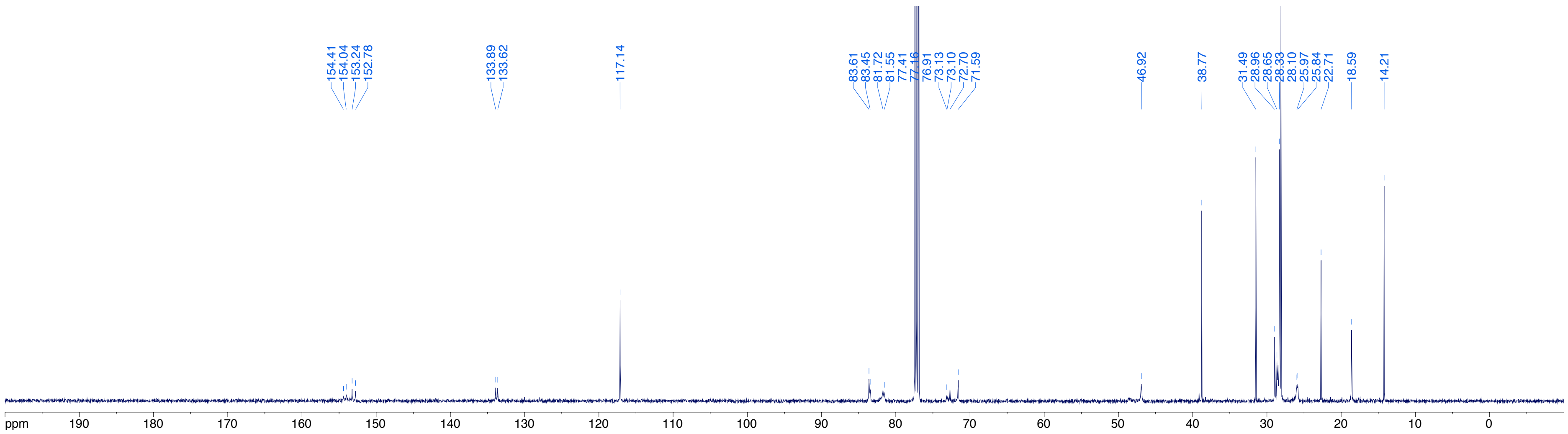

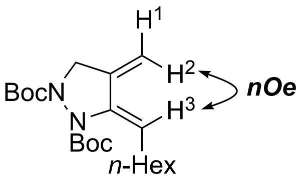

35a

^{1}H: 500 MHz, $CDCl_3$

^{13}C: 125 MHz, $CDCl_3$

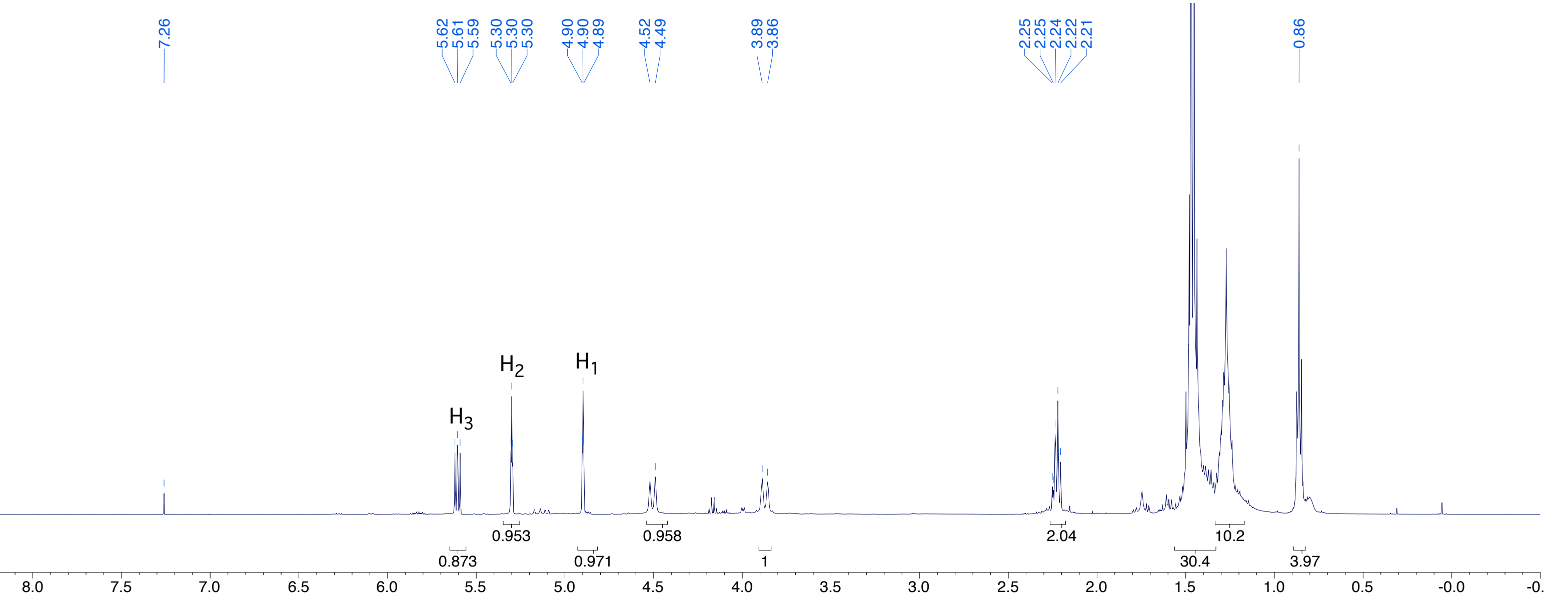

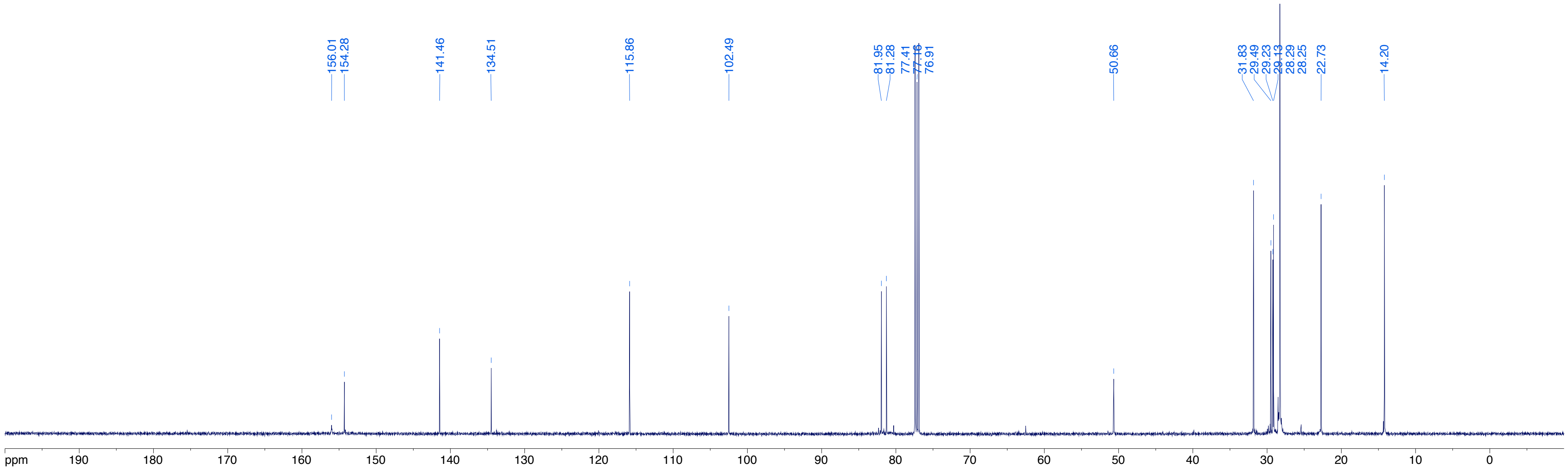

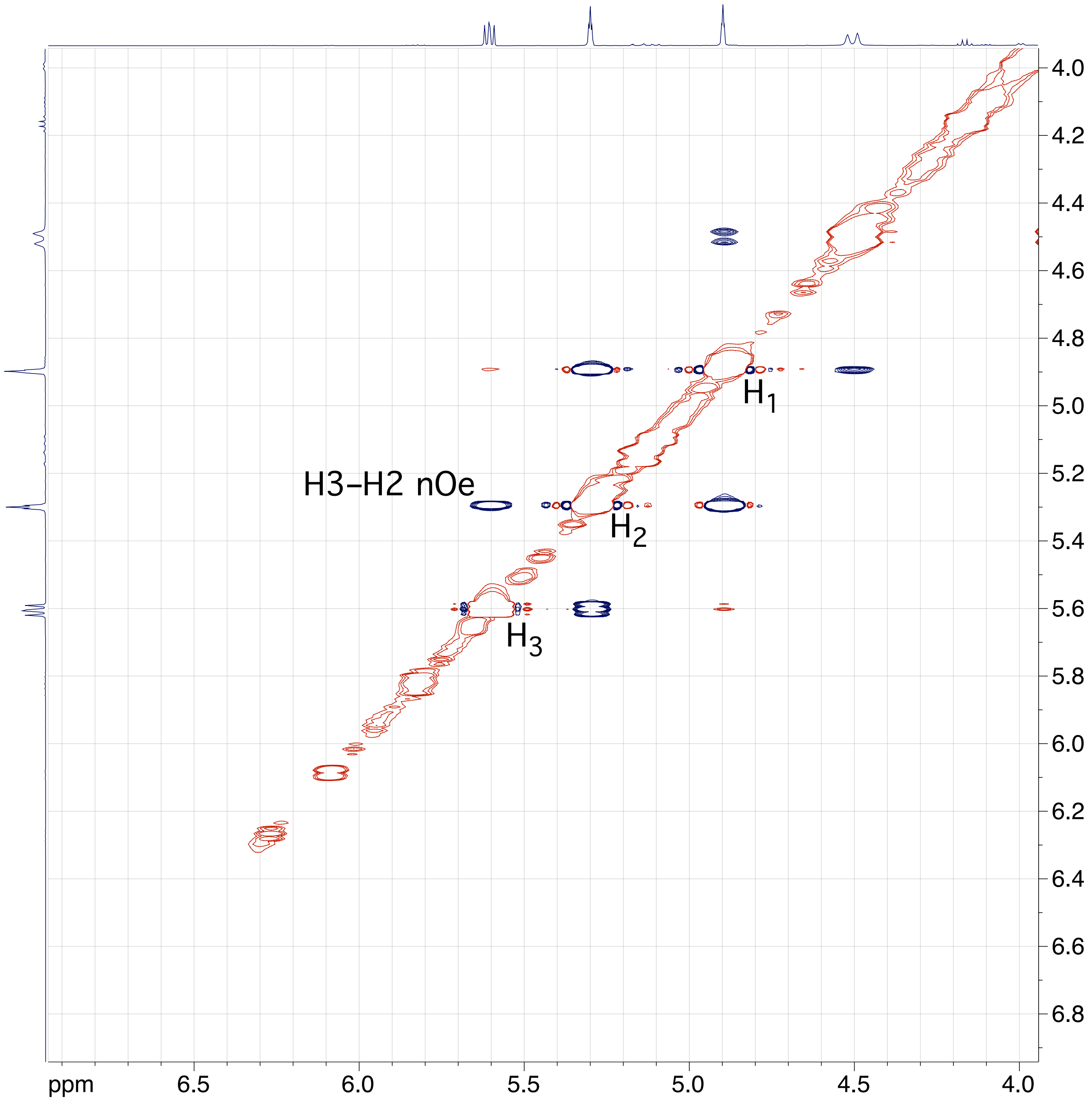

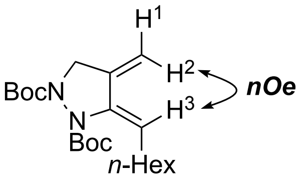

35a

^{1}H: 500 MHz, $CDCl_3$

^{13}C: 125 MHz, $CDCl_3$

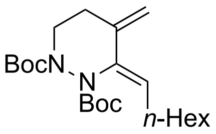

35b

^{1}H: 500 MHz, $CDCl_3$

^{13}C: 125 MHz, $CDCl_3$

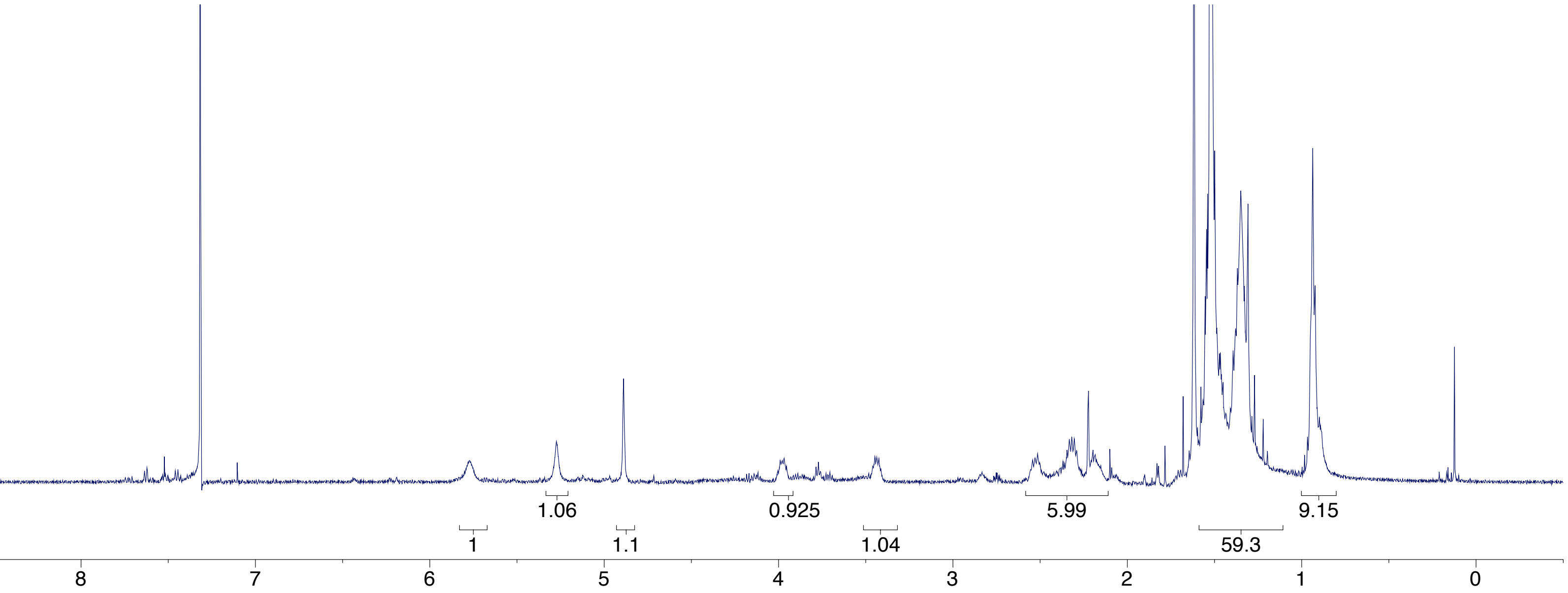

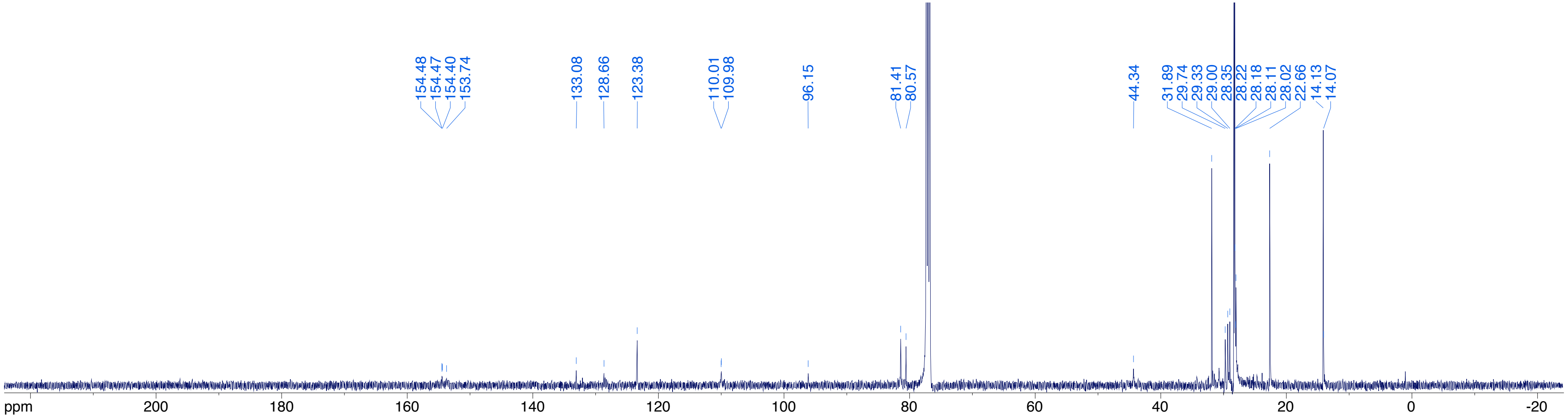

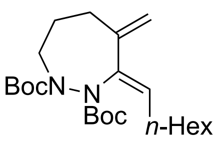

35c

^{1}H: 500 MHz, $CDCl_3$

^{13}C: 125 MHz, $CDCl_3$

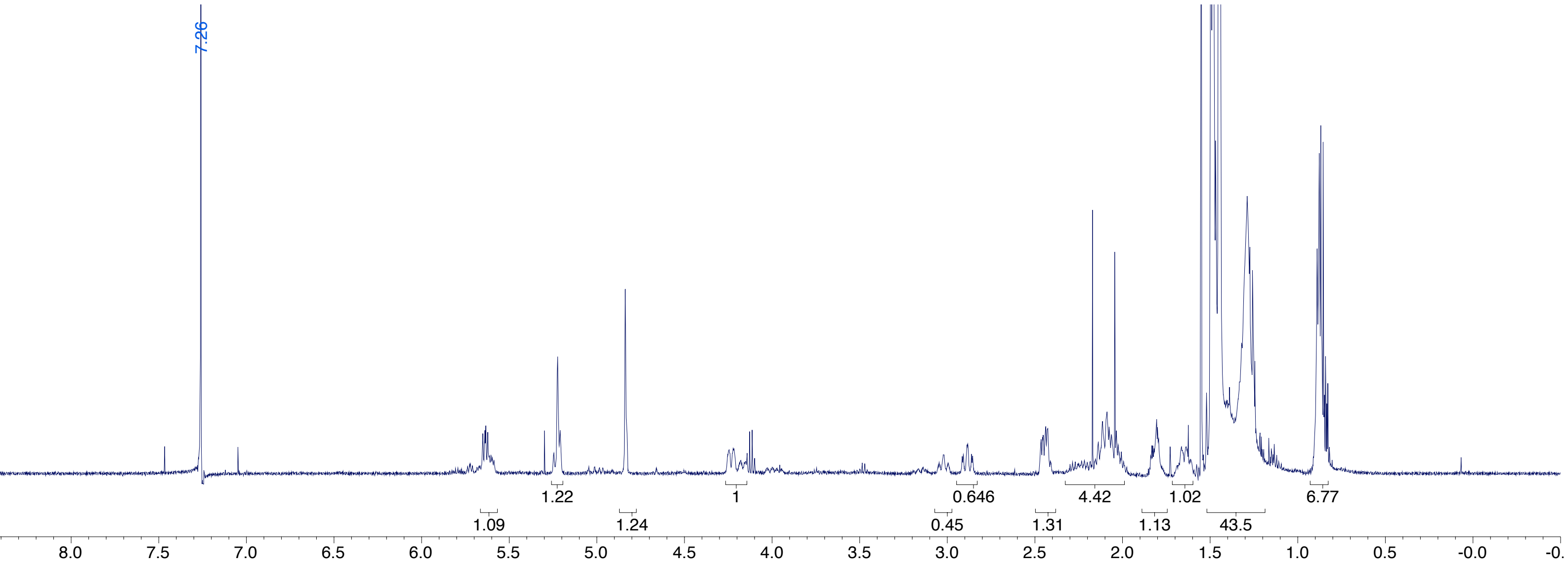

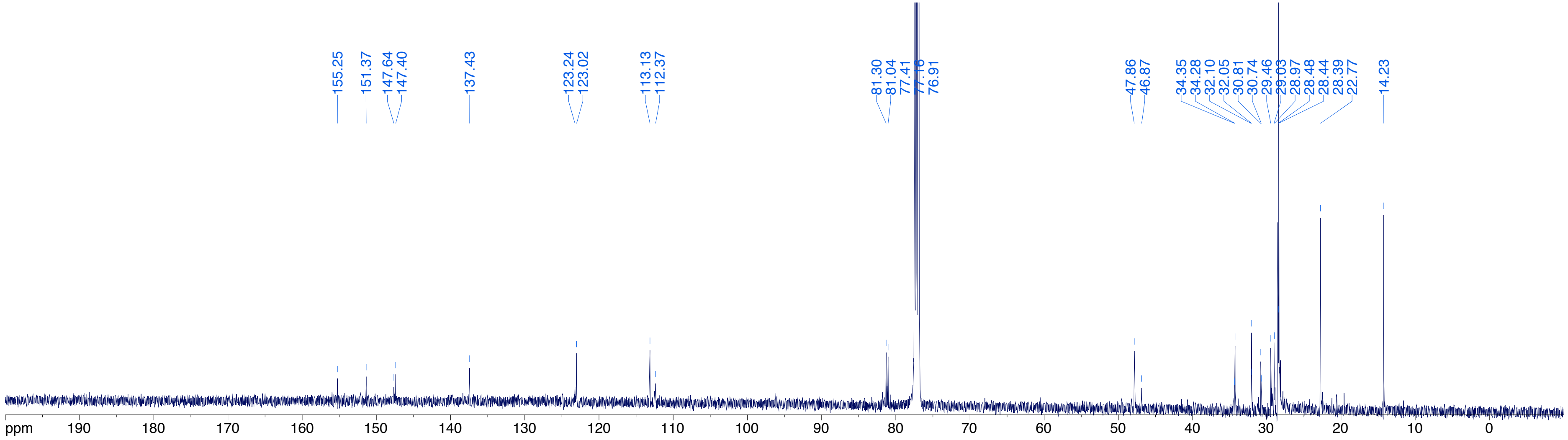

Note: rotameric forms of Boc groups lead to doubling and broadening of signals

(Hetero-) Diels-Alder Cycloadditions of Amidodienes 5a, 4c and 4d

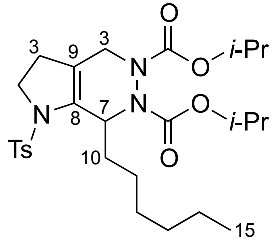

36a

^{1}H: 500 MHz, d_8-tol, 363 K

^{13}C: 125 MHz, d_8-tol, 363 K

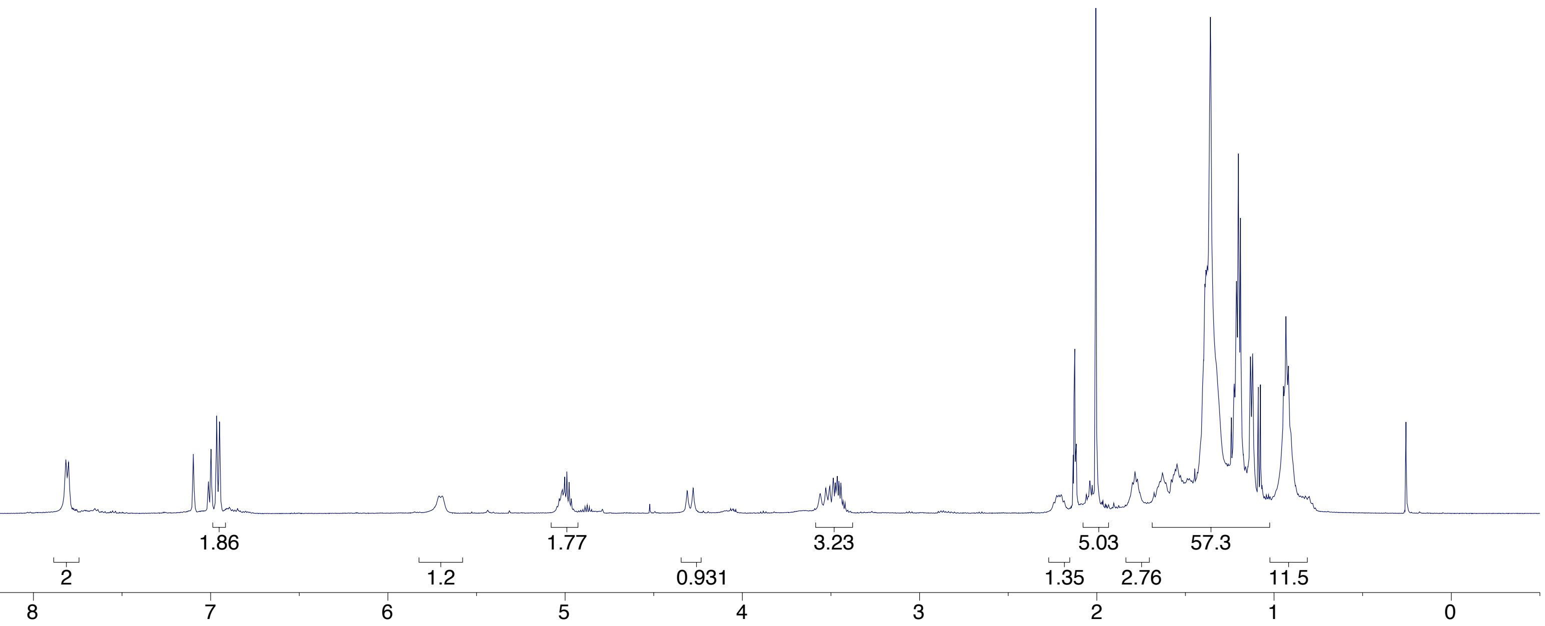

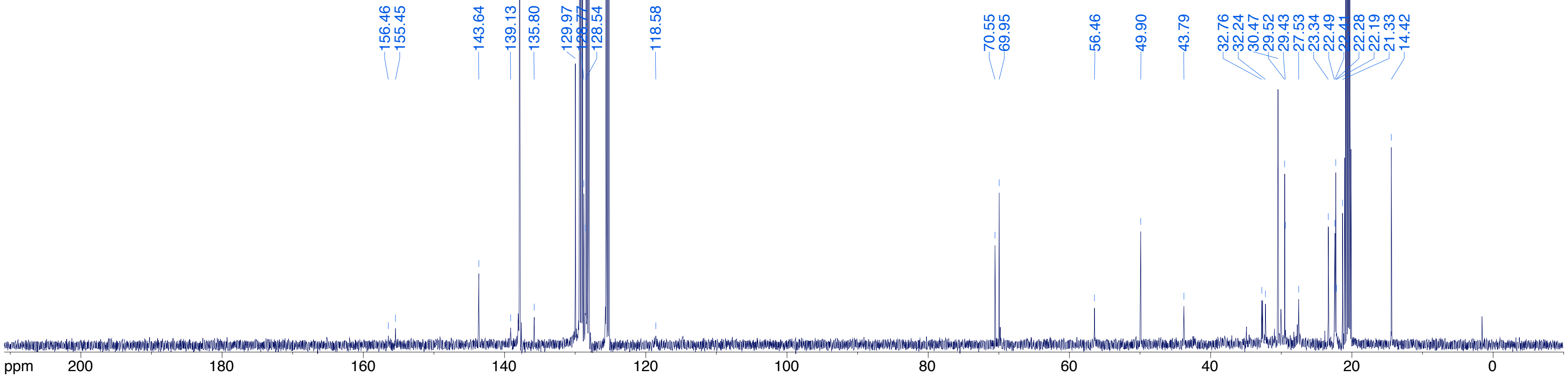

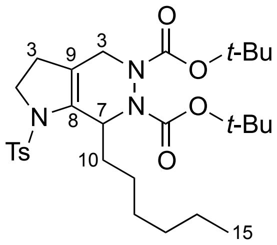

36b

^1H: 500 MHz, $CDCl_3$

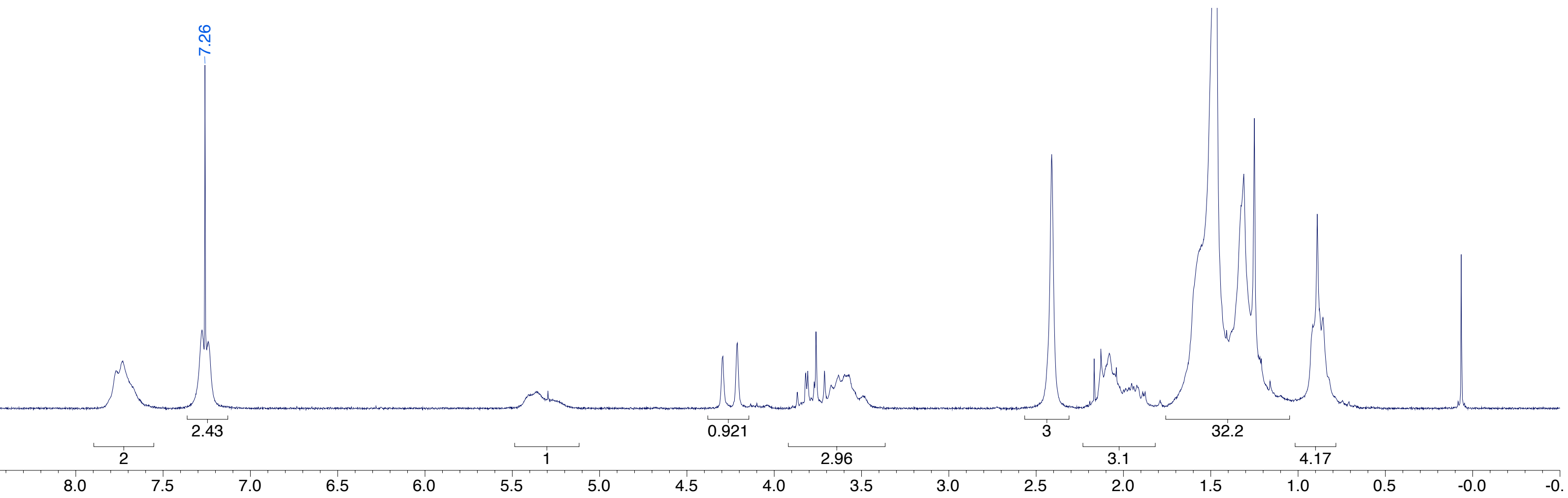

A ^{13}C NMR could not be obtained for this compound due to broad / unresolved peaks at RT, and decomposition of the compound at higher temperatures (loss of Boc groups).

Other spectroscopic and physical properties of this cycloadduct closely resemble **36a**, supporting assignment (including HRMS).

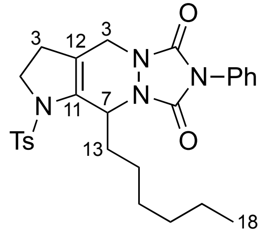

36c

^{1}H: 500 MHz, $CDCl_3$

^{13}C: 125 MHz, $CDCl_3$

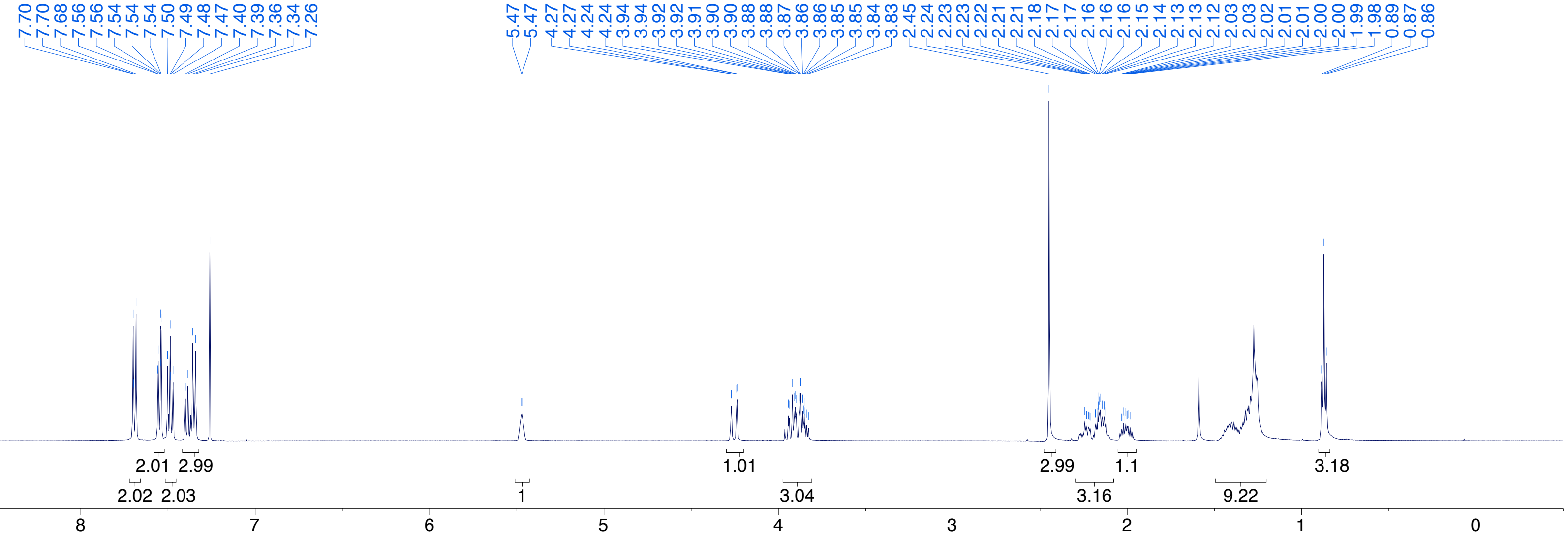

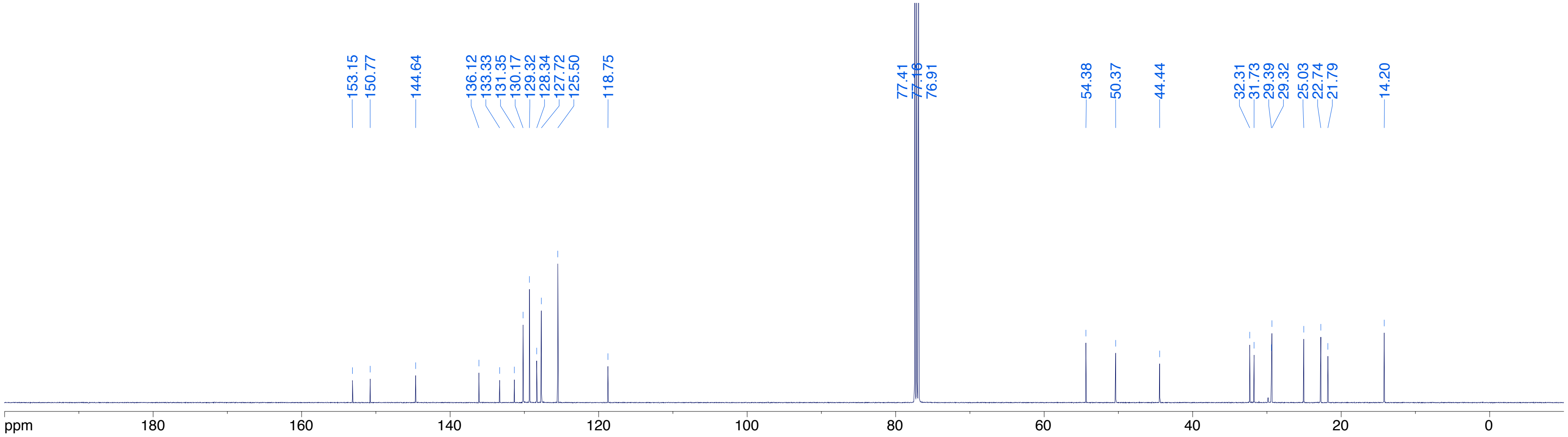

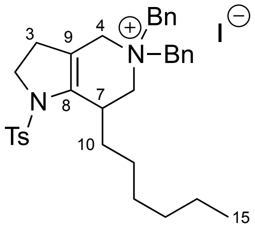

36e

^{1}H: 500 MHz, $CDCl_3$

^{13}C: 125 MHz, $CDCl_3$

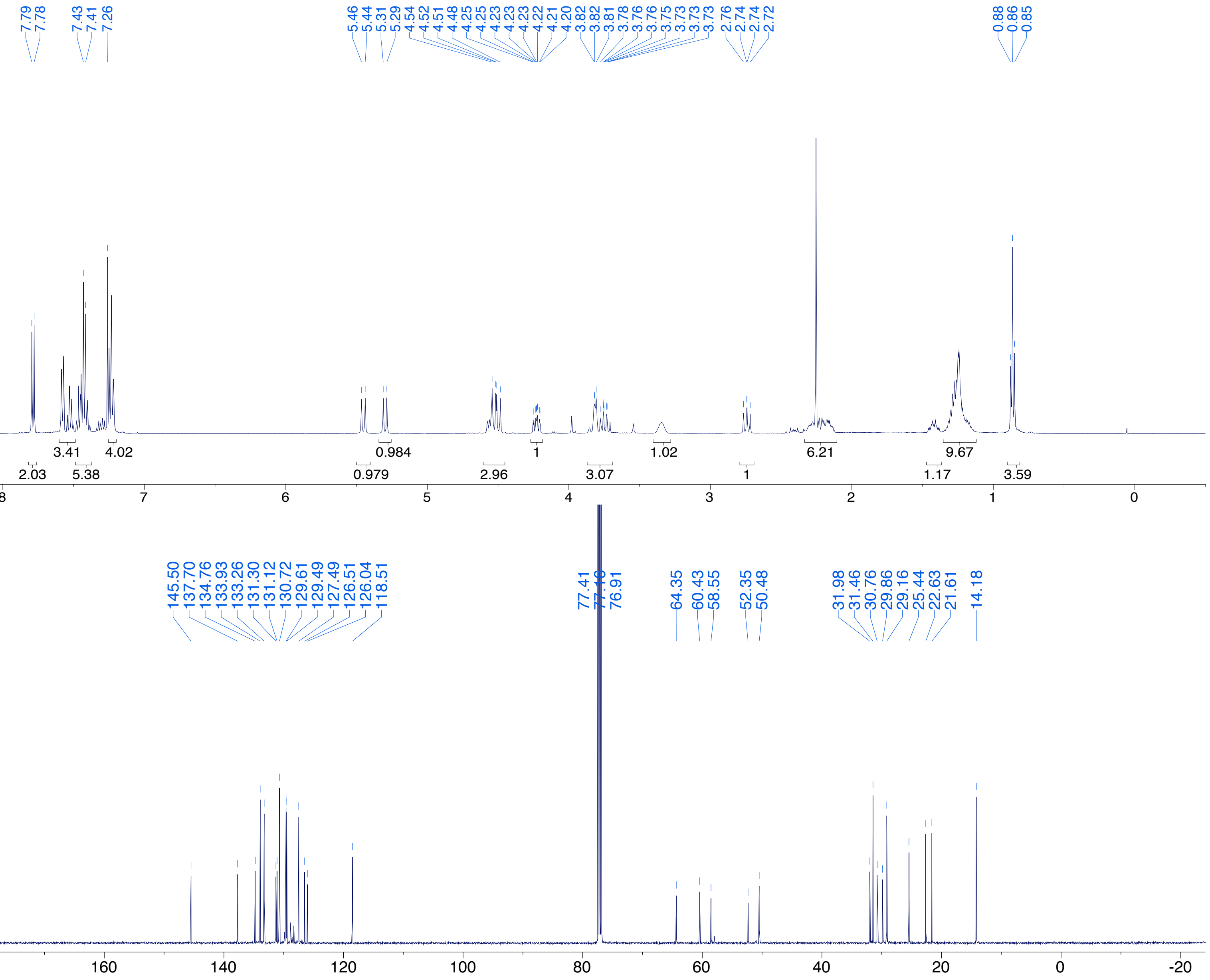

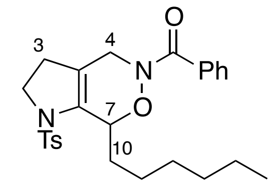

36e

^1H: 500 MHz, $CDCl_3$

^{13}C: 125 MHz, $CDCl_3$

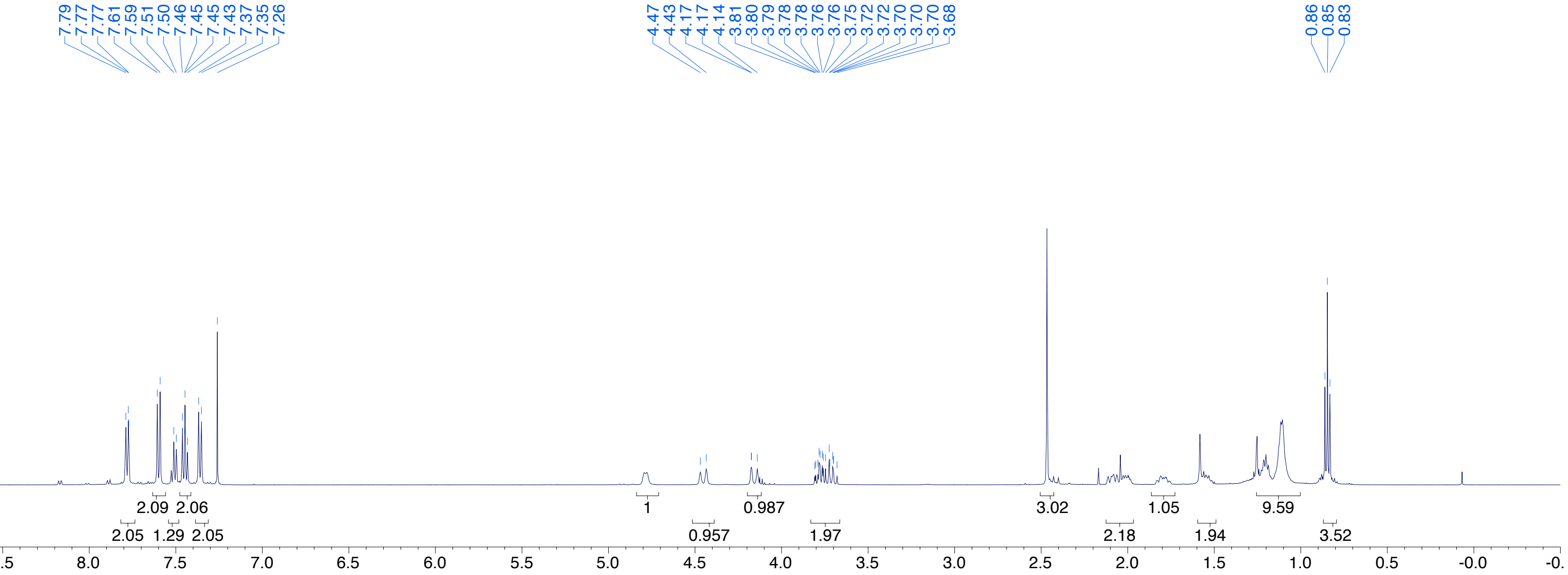

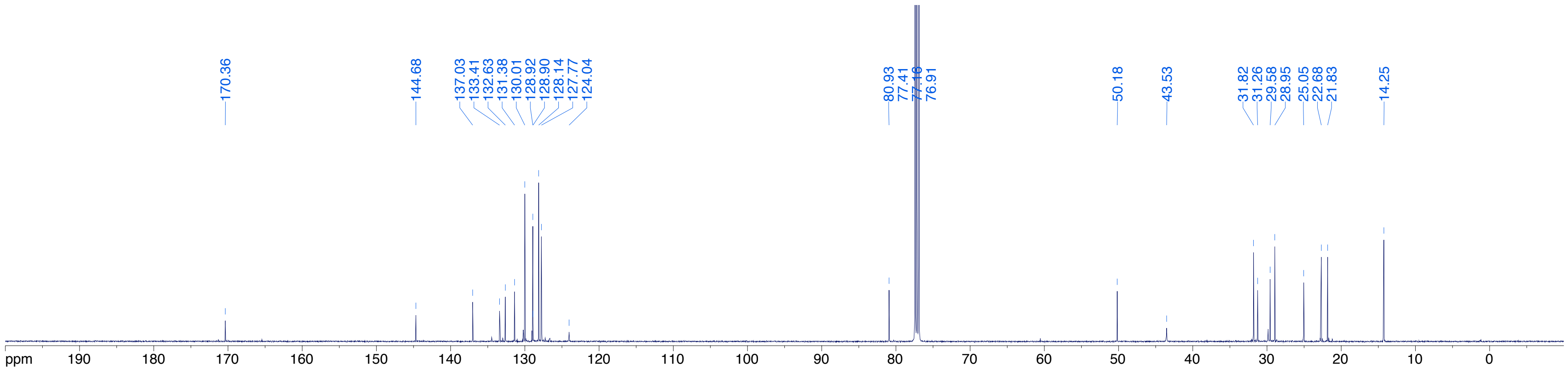

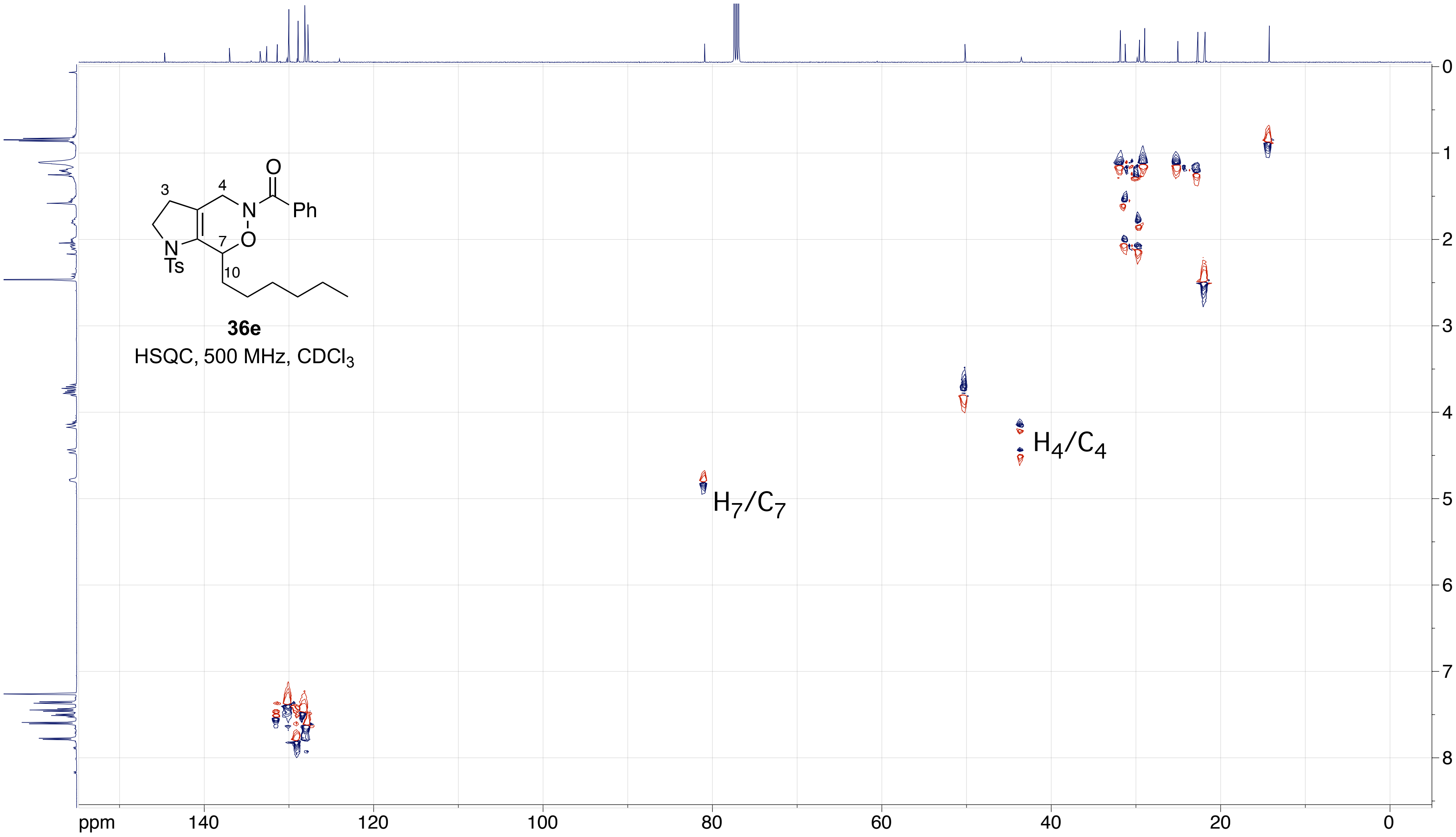
3
4
O
N
Ph
N
O
7
Ts
10
36e
HSQC, 500 MHz, CDCl3
H7/C7
H4/C4
ppm
140
120
100
80
60
40
20
0
1
2
3
4
5
6
7
8

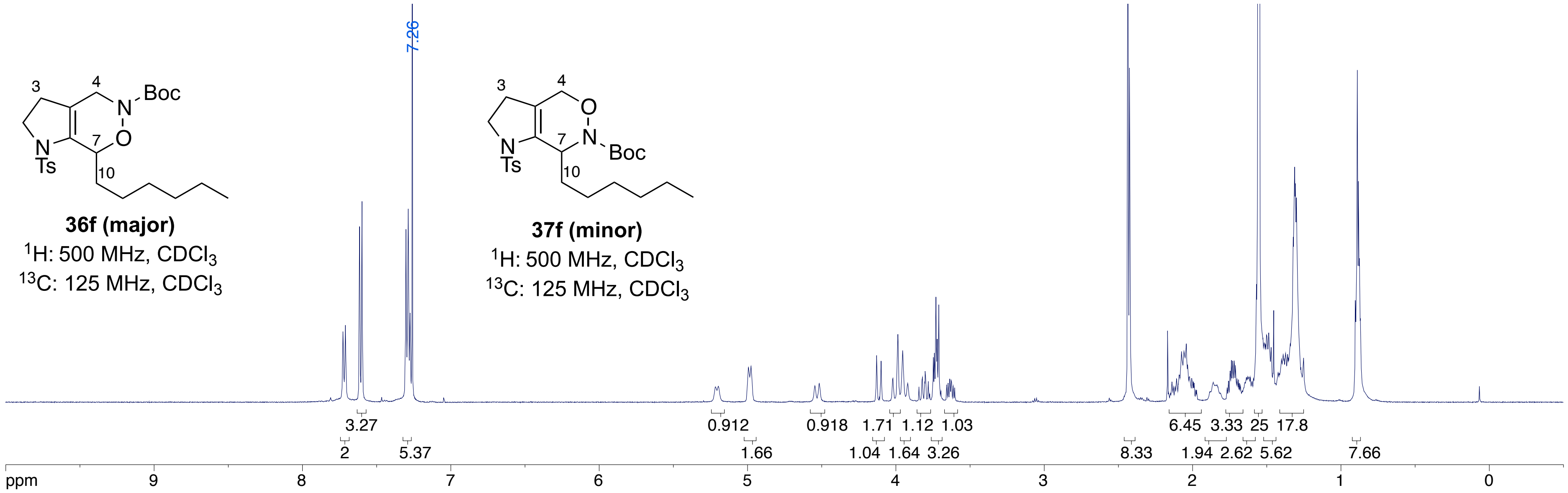
3
4
N
Boc
O
N
Ts
7
10
36f (major)
1H: 500 MHz, CDCl3
13C: 125 MHz, CDCl3
3
4
O
N
Boc
N
Ts
7
10
37f (minor)
1H: 500 MHz, CDCl3
13C: 125 MHz, CDCl3
7.26
3.27
2
5.37
0.912
1.66
0.918
1.71
1.04
1.12
1.64
1.03
3.26
8.33
6.45
1.94
3.33
2.62
25
5.62
17.8
7.66
ppm
9
8
7
6
5
4
3
2
1
0

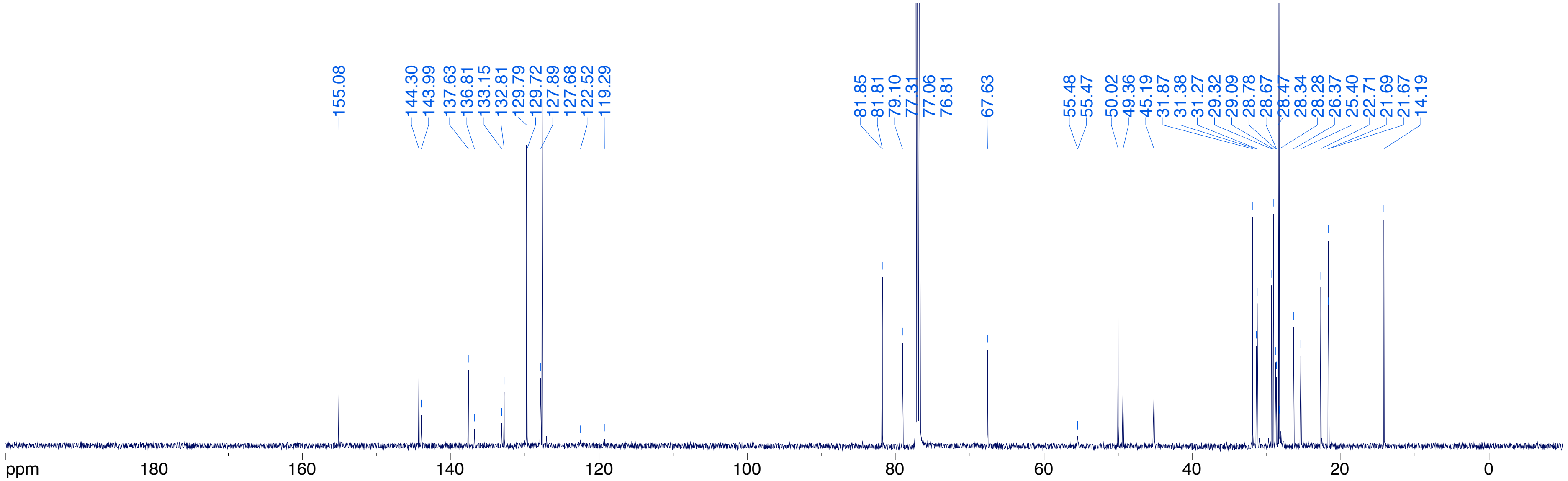
155.08
144.30
143.99
137.63
136.81
133.15
132.81
129.79
129.72
127.89
127.68
122.52
119.29
81.85
81.81
79.10
77.31
77.06
76.81
67.63
55.48
55.47
50.02
49.36
45.19
31.87
31.38
31.27
29.32
29.09
28.78
28.67
28.47
28.34
28.28
26.37
25.40
22.71
21.69
21.67
14.19
ppm
180
160
140
120
100
80
60
40
20
0

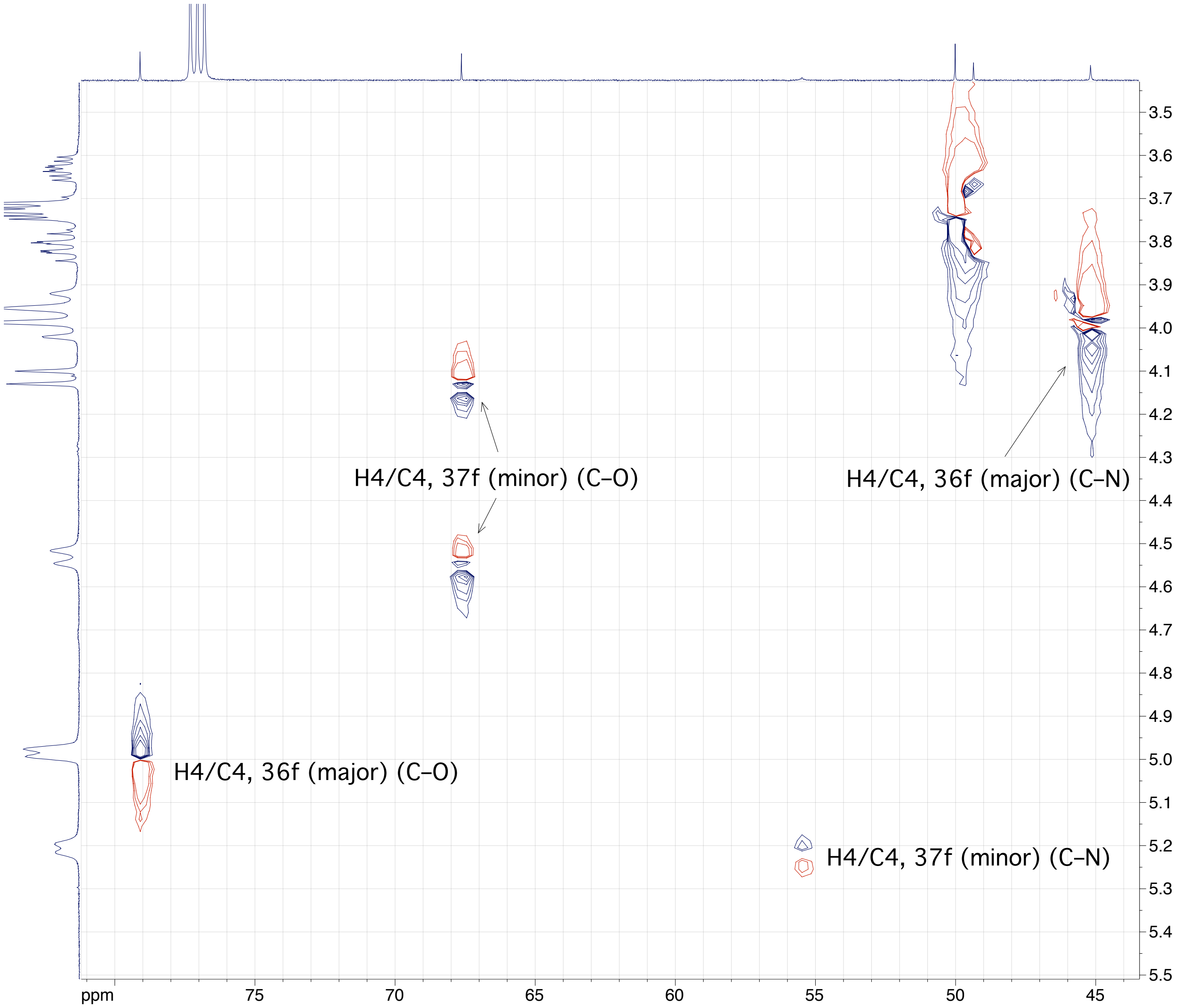

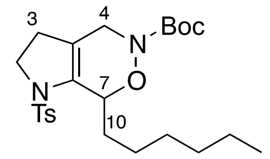

36f (major)
HSQC, 500 MHz, $CDCl_3$

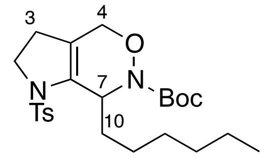

37f (minor)
HSQC, 500 MHz, $CDCl_3$

HPLC-purified **36f**

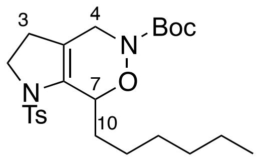

36f (major)

^{1}H: 500 MHz, $CDCl_3$

^{13}C: 125 MHz, $CDCl_3$

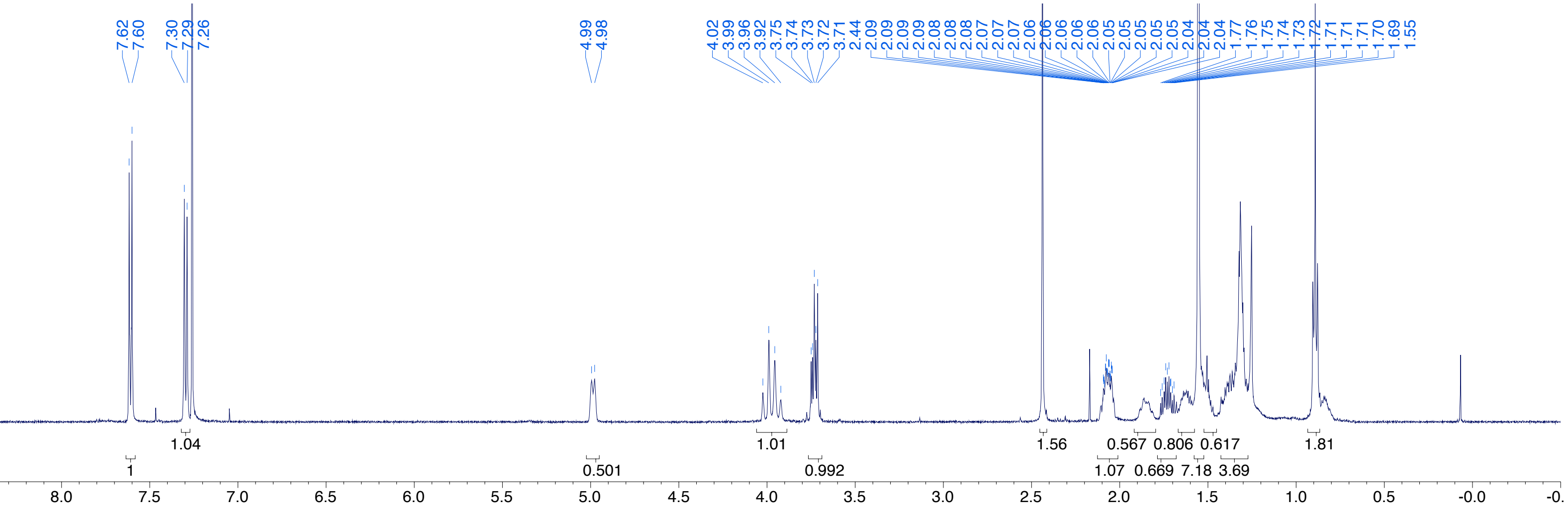

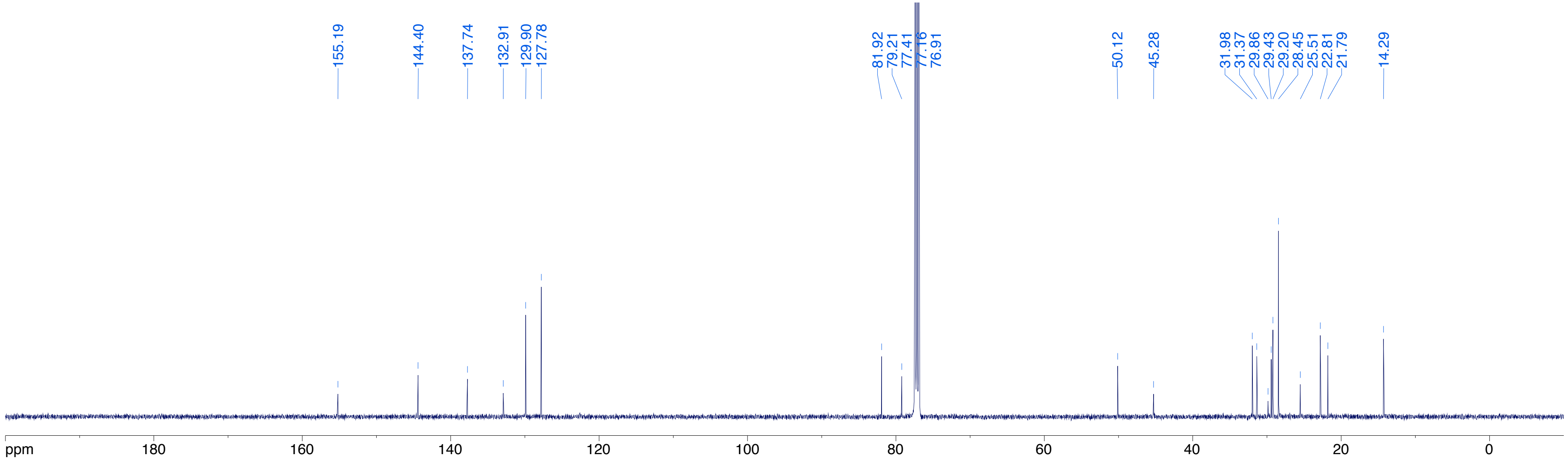

HPLC-purified **37f**

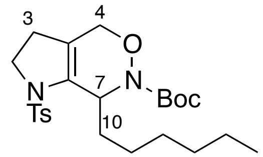

37f (minor)

^{1}H: 500 MHz, $CDCl_3$

^{13}C: 125 MHz, $CDCl_3$

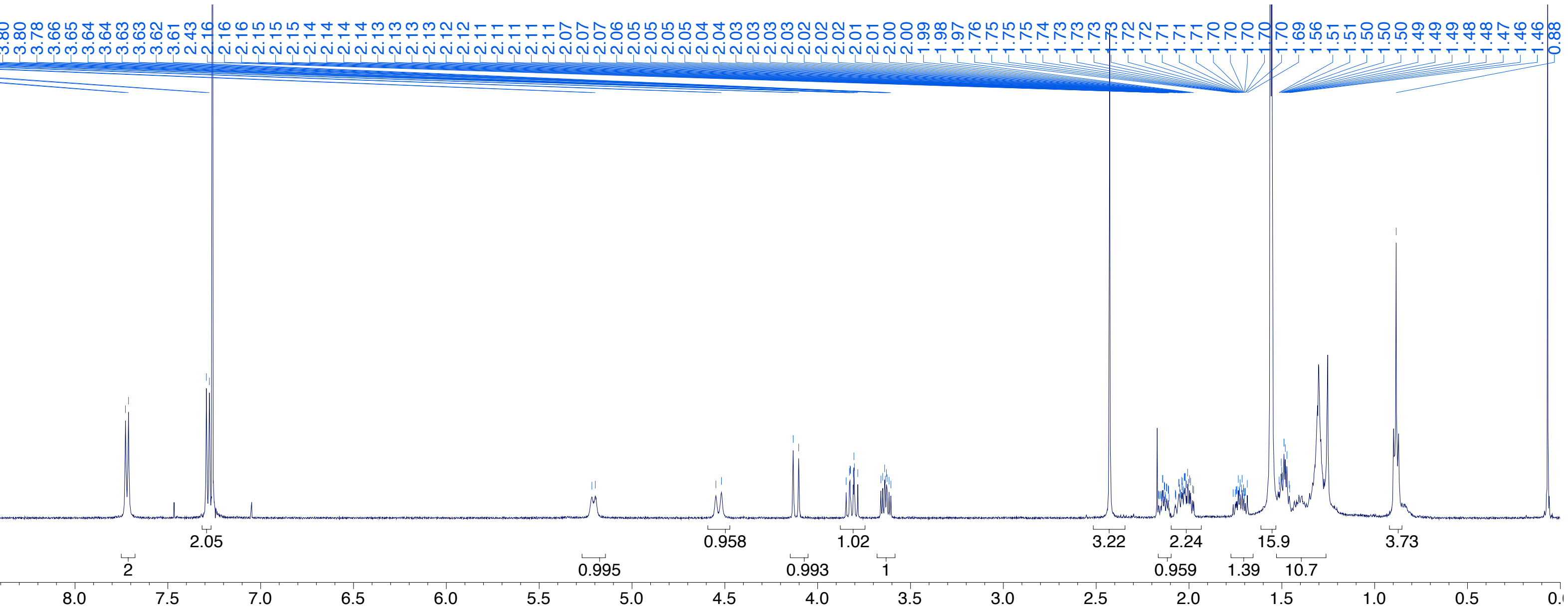

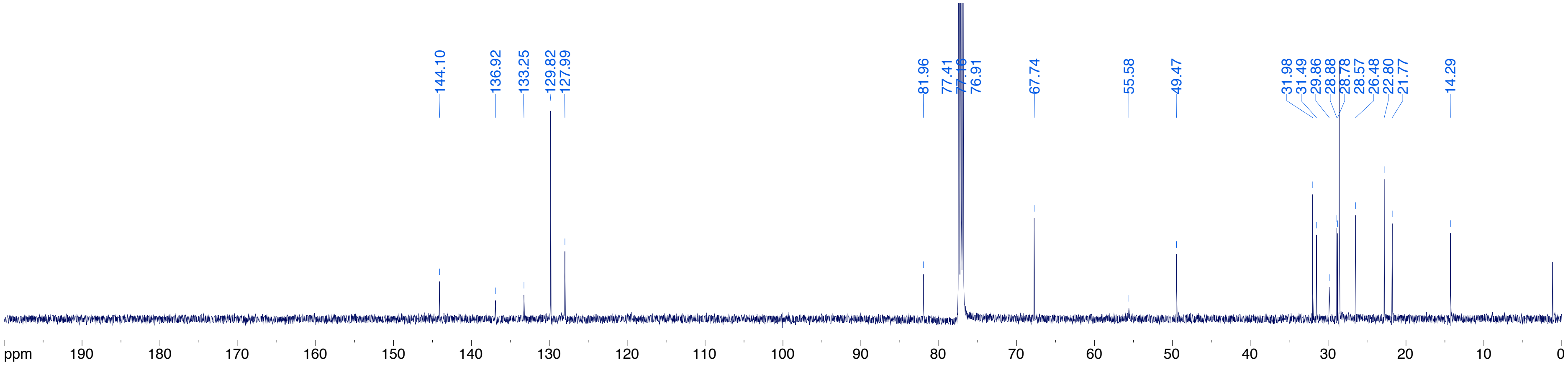

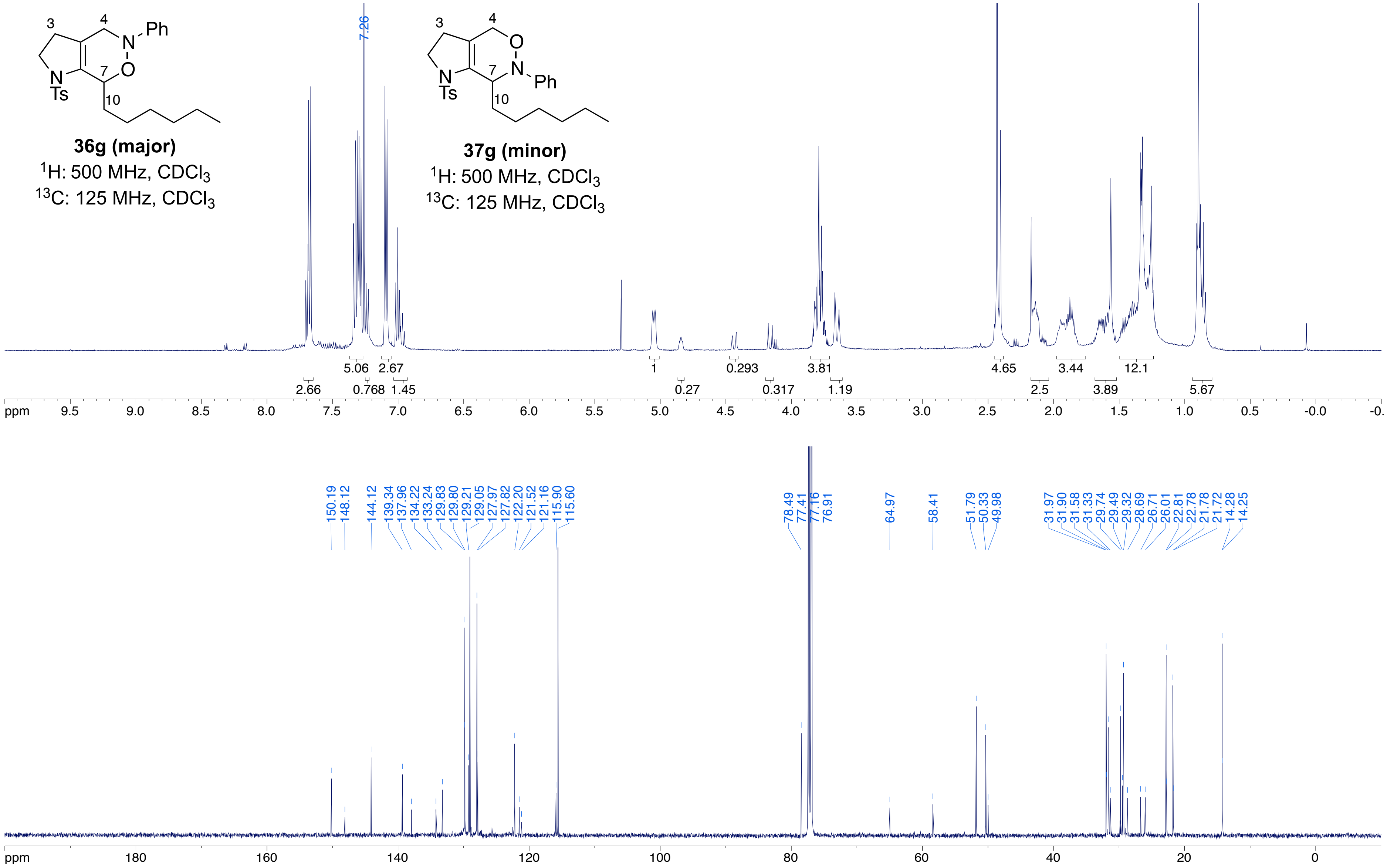

These spectra are of a mixture of the cycloadducts, 3.7:1 ratio. For the purposes of additional characterization, small amounts of each could be separated by HPLC. The amount isolated was only sufficient to obtain a 700 MHz ^{1}H NMR spectrum (see below).

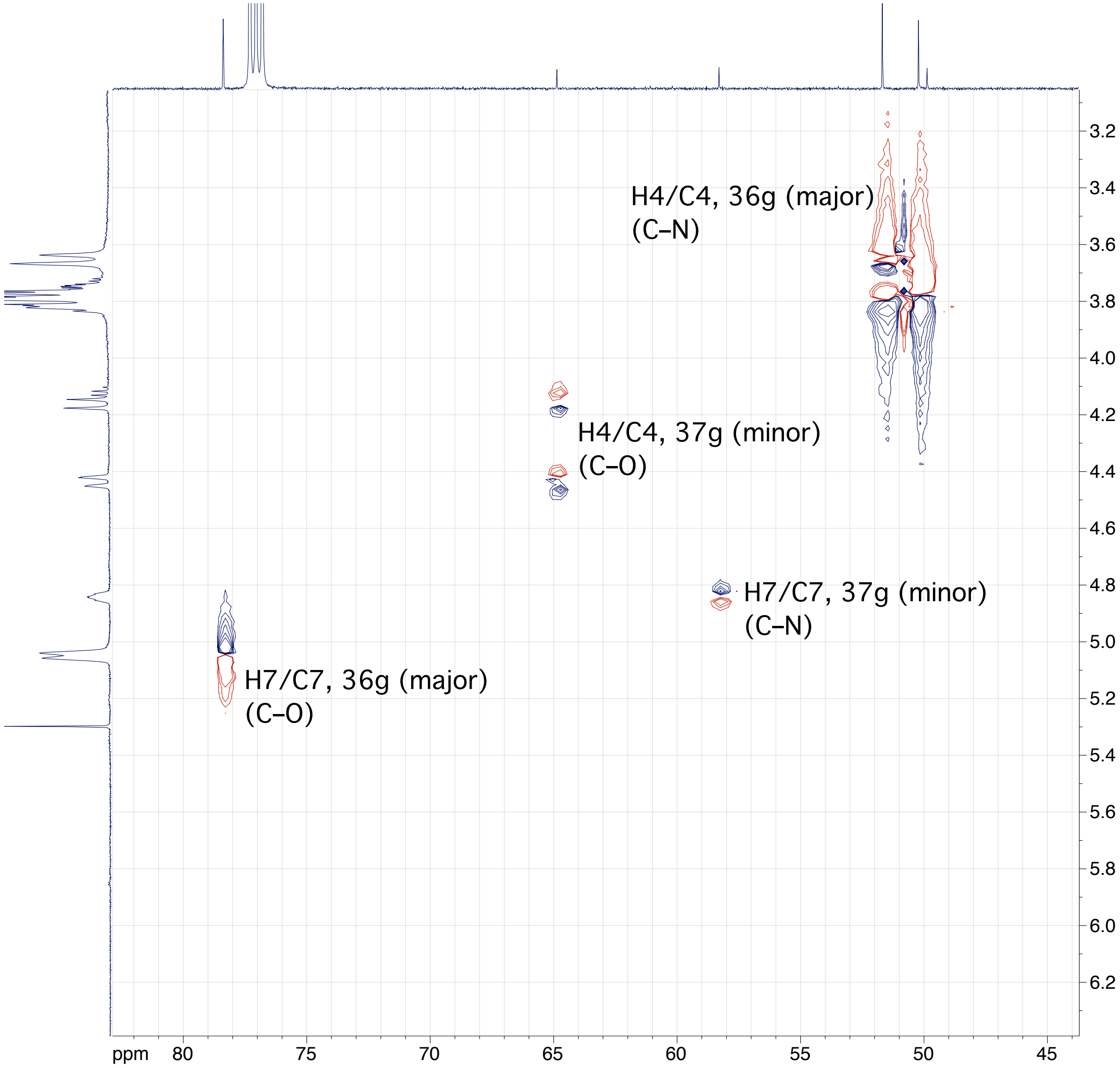

HPLC-purified **36g/37g**

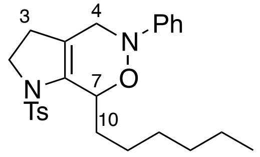

36g (major)

HSQC, 500 MHz, $CDCl_3$

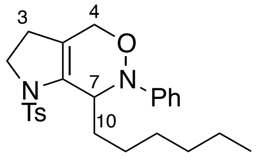

37g (minor)

HSQC, 500 MHz, $CDCl_3$

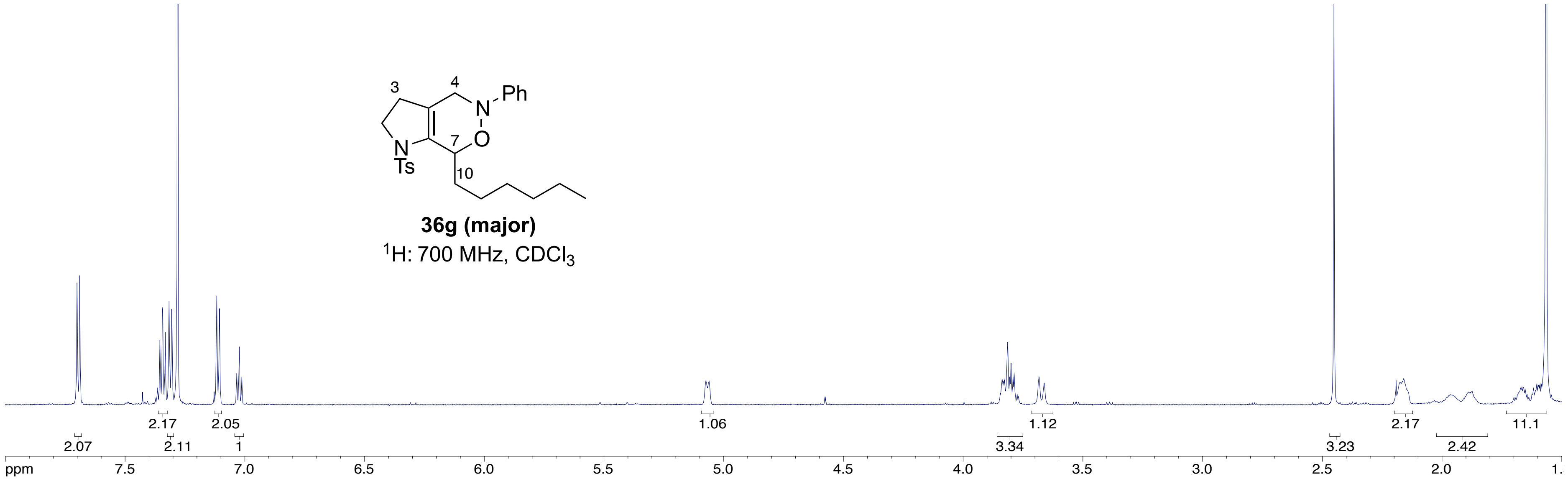

3
4
N
Ph
N
O
7
Ts
10
36g (major)
^{1}H: 700 MHz, CDCl$_3$
2.17
2.05
1.06
1.12
2.17
11.1
2.07
2.11
1
3.34
3.23
2.42
ppm 7.5 7.0 6.5 6.0 5.5 5.0 4.5 4.0 3.5 3.0 2.5 2.0

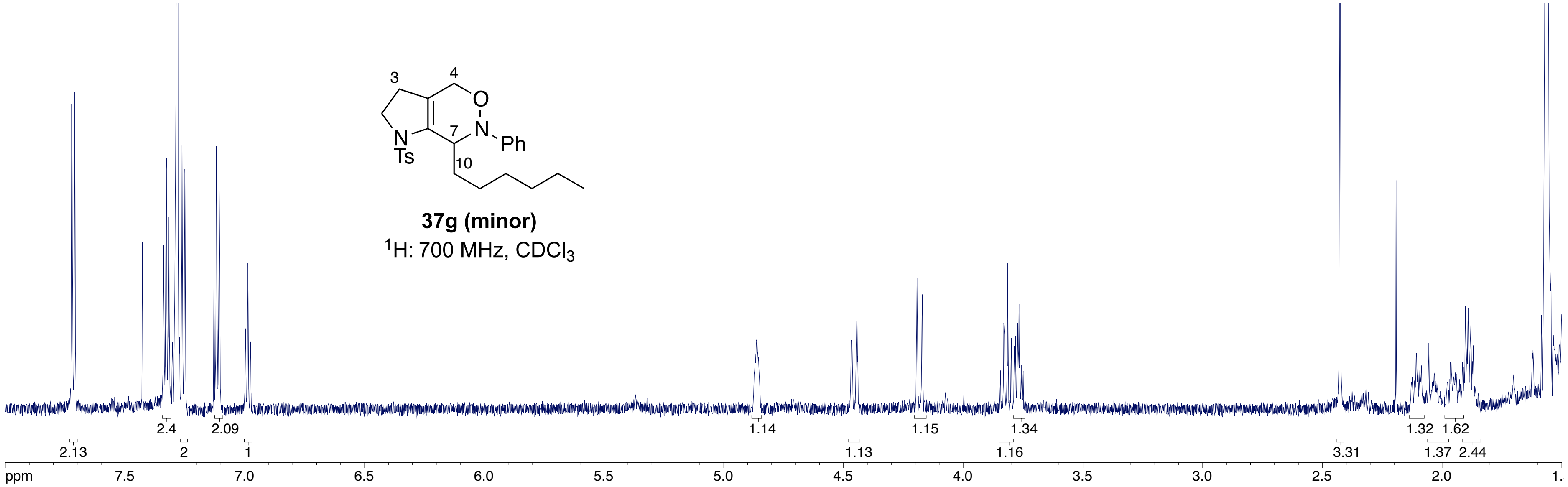

3
4
O
N
7
N
Ph
Ts
10
37g (minor)
^{1}H: 700 MHz, CDCl$_3$
2.4
2.09
1.14
1.15
1.34
1.32
1.62
2.13
2
1
1.13
1.16
3.31
1.37
2.44
ppm 7.5 7.0 6.5 6.0 5.5 5.0 4.5 4.0 3.5 3.0 2.5 2.0

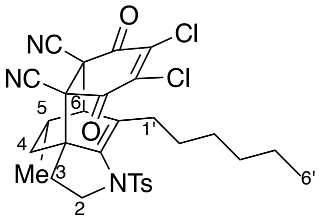

38

^{1}H: 500 MHz, $CDCl_3$

^{13}C: 125 MHz, $CDCl_3$

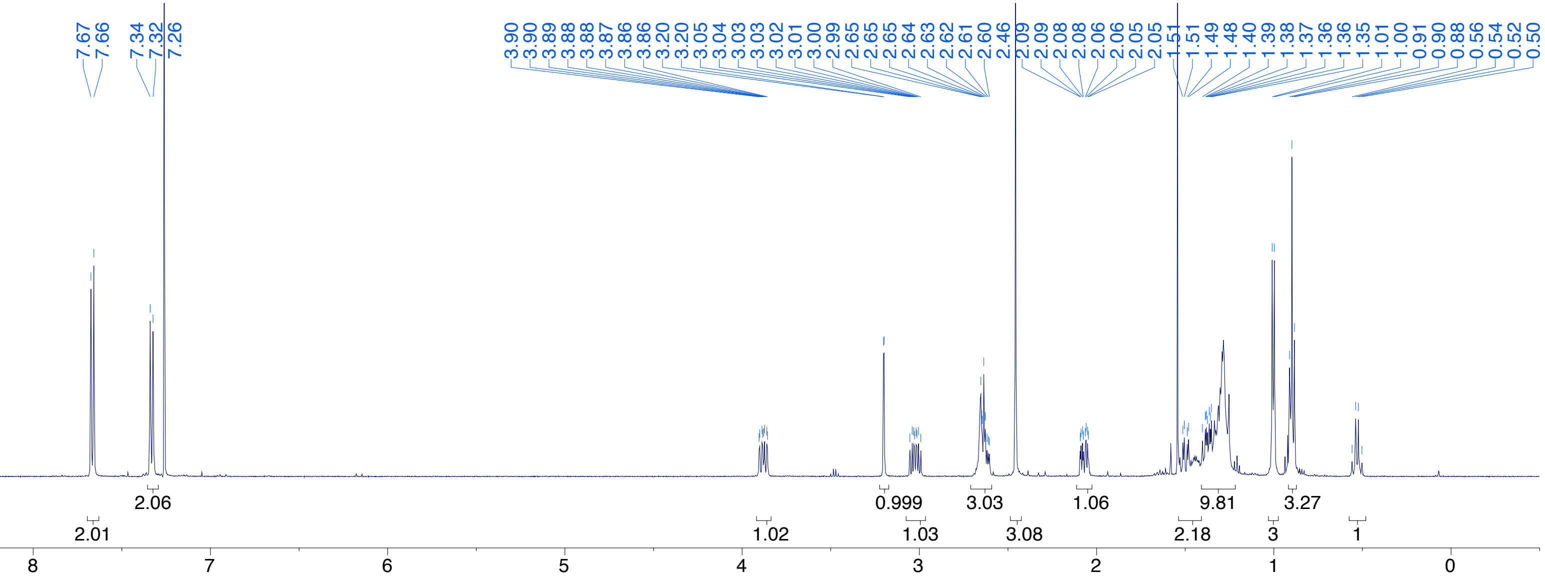

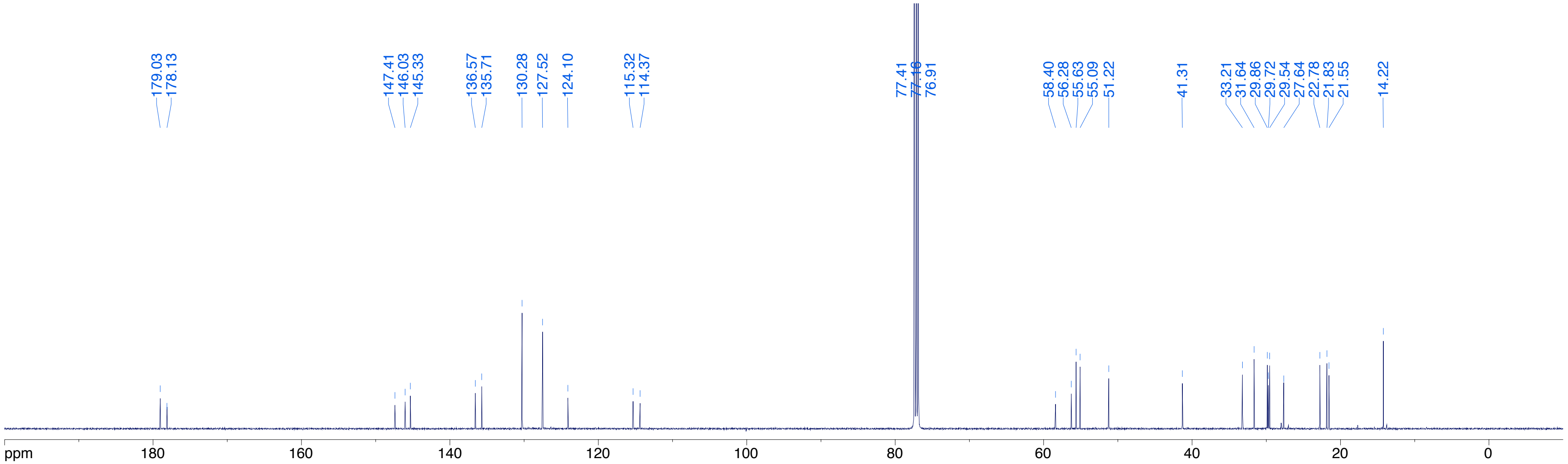

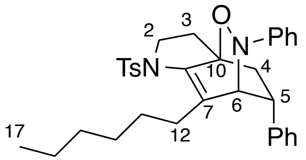

39

^{1}H: 500 MHz, CDCl$_3$

^{13}C: 125 MHz, CDCl$_3$

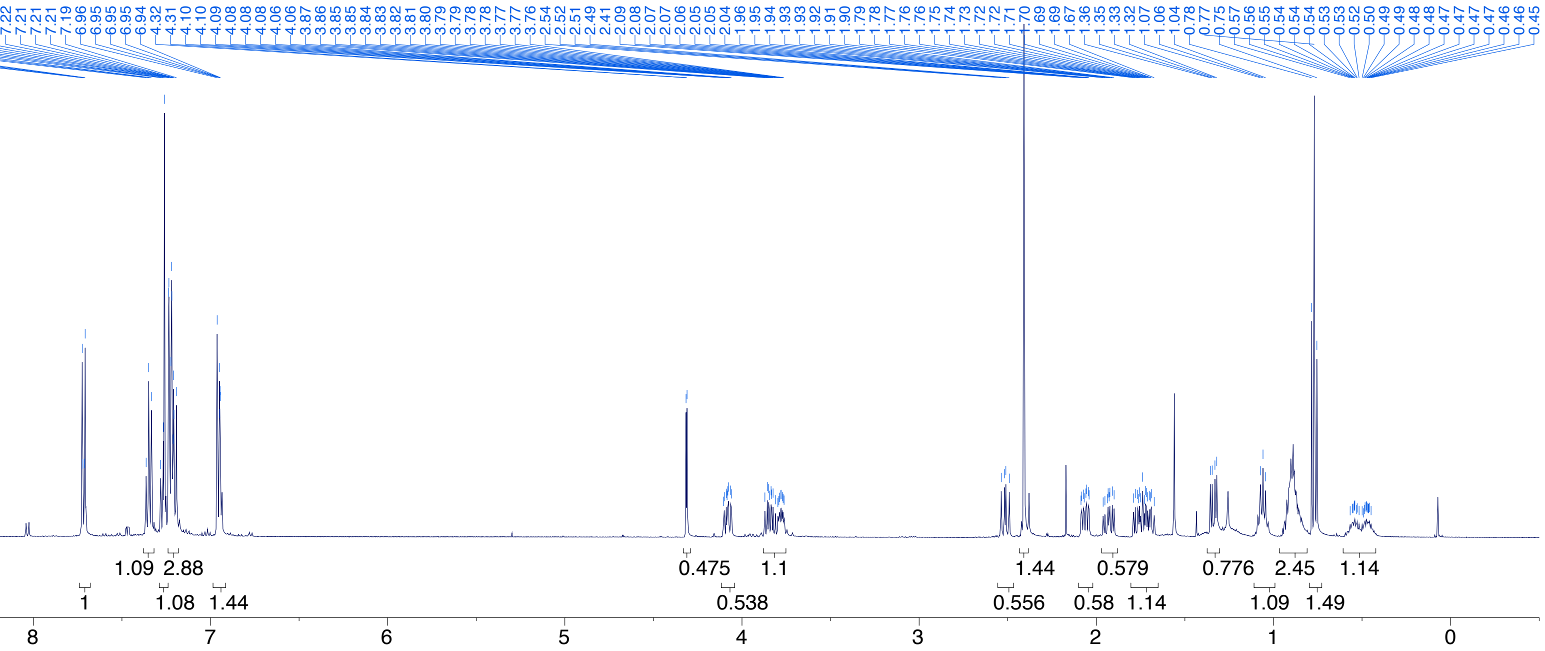

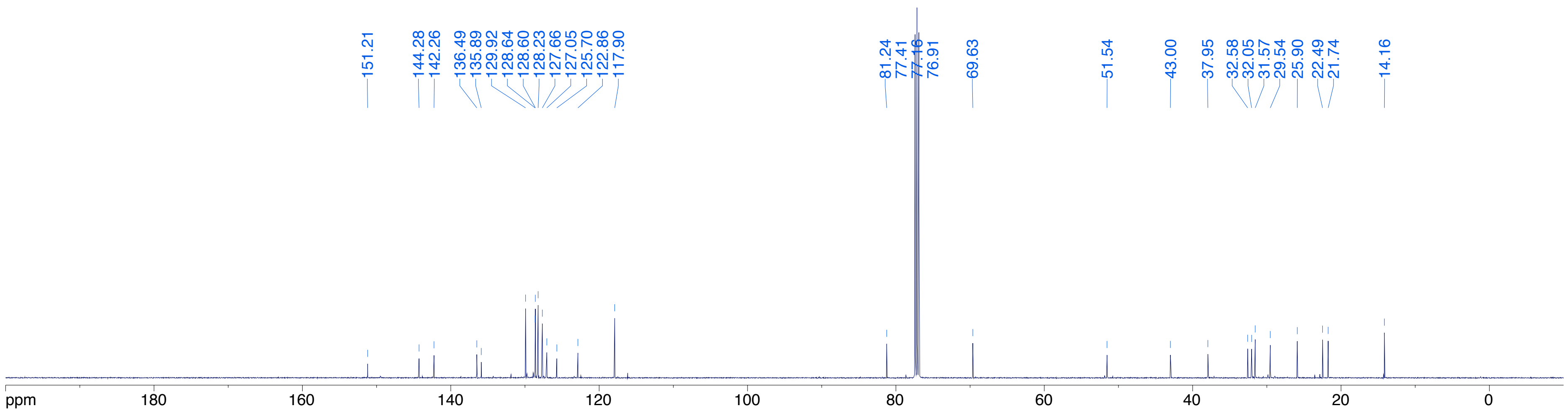

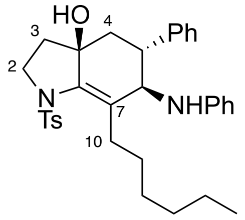

41

^{1}H: 500 MHz, $CDCl_3$

^{13}C: 125 MHz, $CDCl_3$

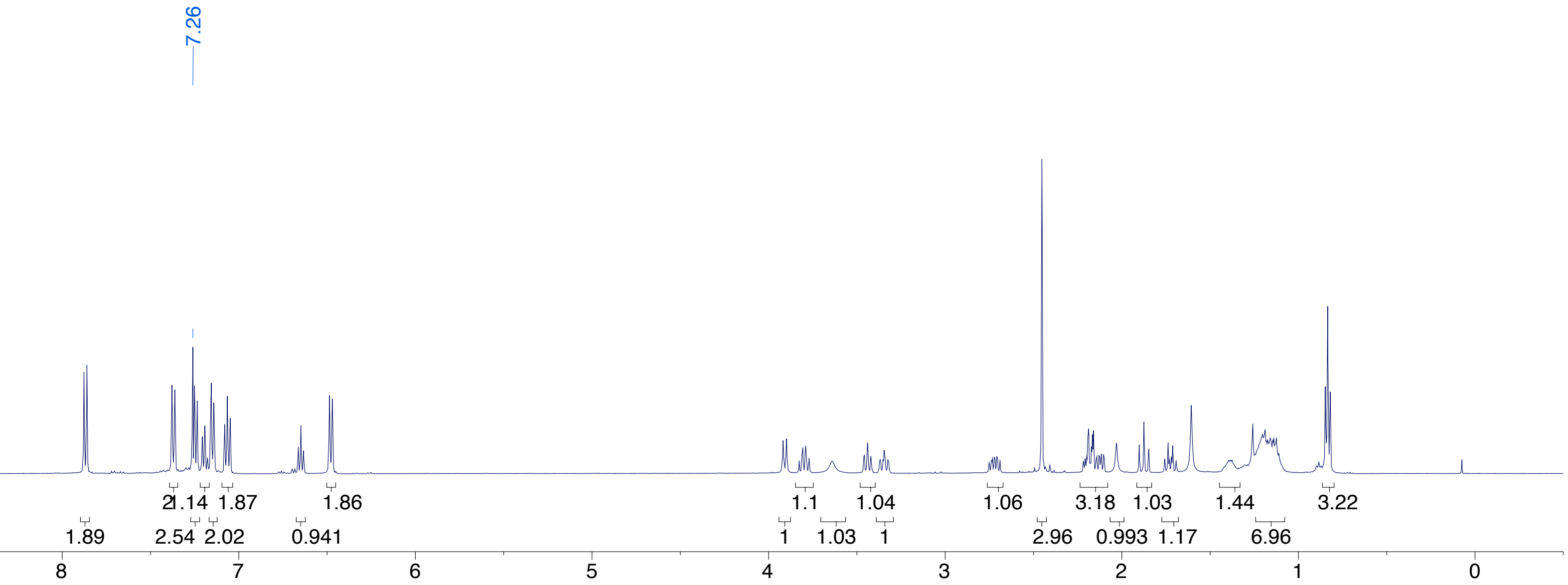

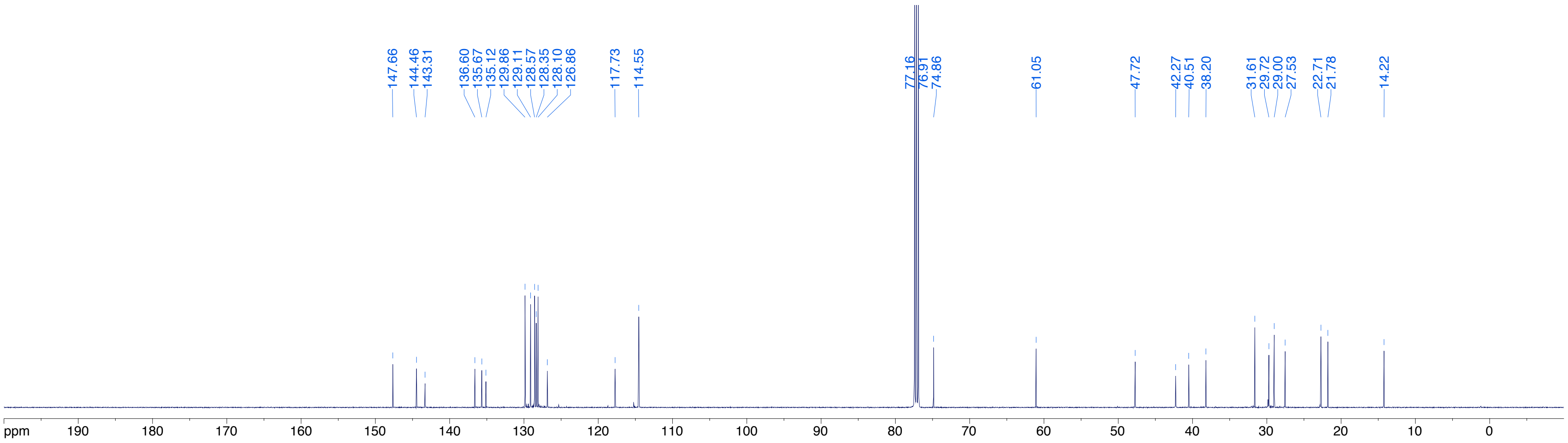

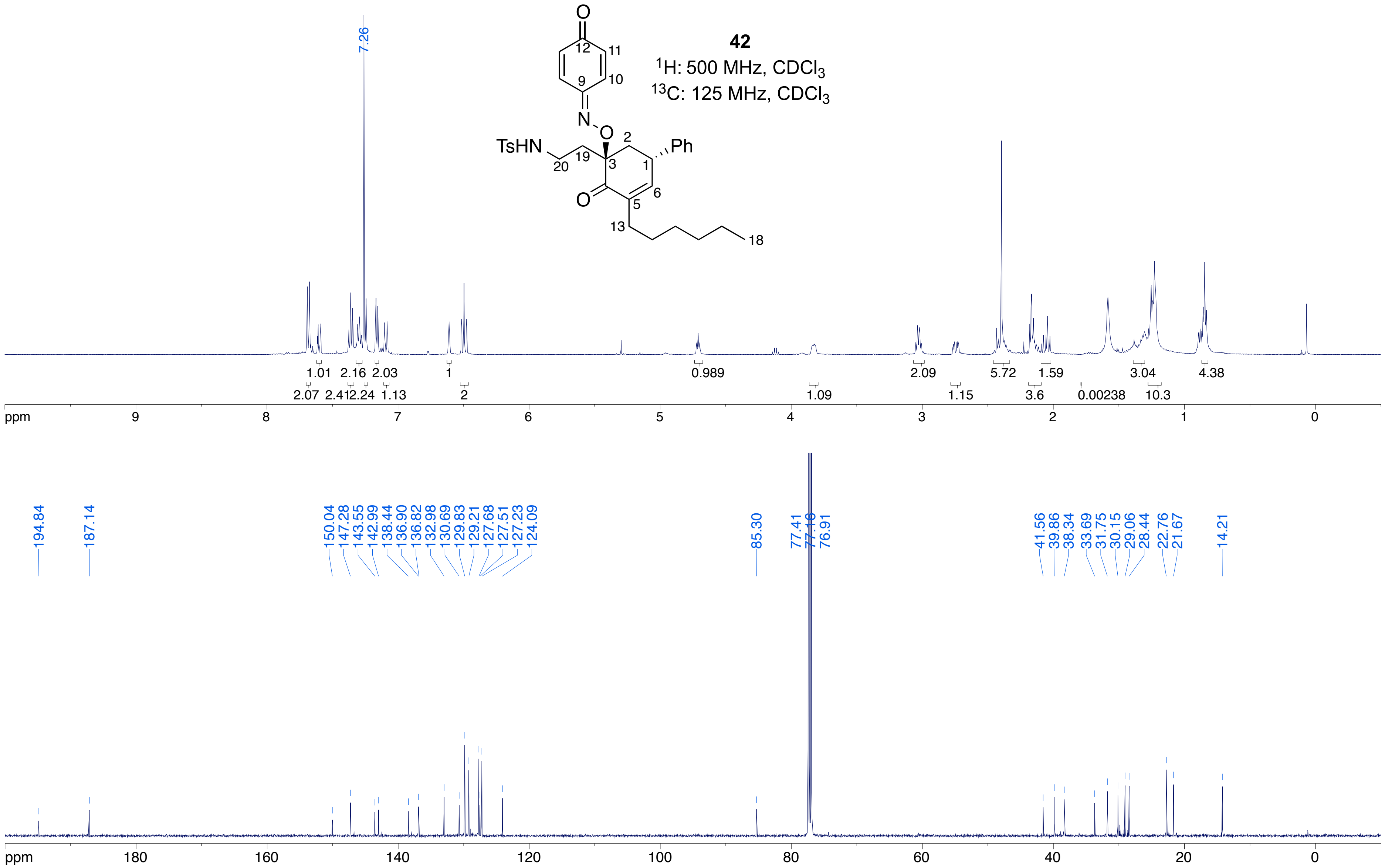

42

^{1}H: 500 MHz, $CDCl_3$

^{13}C: 125 MHz, $CDCl_3$

[1] T. Kauffmann, D. Stach, *Chem. Ber.* **1992**, *125*, 913.
[2] J. Pillai, *D. Phil. Thesis, University of Oxford*, **1998**.
[3] J. P. Gesson, J. C. Jacquesy, D. Rambaud, *Bull. Soc. Chim. Fr.* **1992**, *129*, 227.
[4] A. Padwa, A. G. Waterson, *Tetrahedron* **2000**, *56*, 10159.
[5] H. Lu, Q. Chen, C. Li, *J. Org. Chem.* **2007**, *72*, 2564.
[6] R. L. Greenaway, C. D. Campbell, O. T. Holton, C. A. Russell, E. A. Anderson, *Chem. Eur. J.* **2011**, *17*, 14366.
